# Supplementary material for: Dopamine neurons do not constitute an obligatory stage in the final common path for the evaluation and pursuit of brain stimulation reward
Source: PLoS One. 2020 Jun 5;15(6):e0226722. doi: 10.1371/journal.pone.0226722 (PMC7274413; doi:10.1371/journal.pone.0226722)
Supplement: S1 File — (PDF) [file pone.0226722.s001.pdf]

Dopamine neurons do not constitute an obligatory stage in the final common path for the evaluation and pursuit of brain stimulation reward

Ivan Trujillo-Pisanty[1,¤a], Kent Conover[1], Pavel Solis[1], Daniel Palacios[1], Peter Shizgal[1,*],

1 Centre for Studies in Behavioural Neurobiology, Concordia University, Montreal, Québec, H4B 1R6, Canada

¤a Current Address: Center for the Neurobiology of Addiction, Pain, and Emotion, Department of Anesthesiology and Pain Medicine, Department of Pharmacology, University of Washington, Seattle, WA, 98195, USA
* peter.shizgal@concordia.ca

Supporting information

Power-frequency trade-off

Fig S1. Response-rate versus pulse-frequency graph for rat Bechr14. The number of responses emitted per 2-min trial by an exemplar rat (Bechr29) is plotted as a function of pulse frequency and optical power.

Fig S2. Response-rate versus pulse-frequency graph for rat Bechr19.

Fig S3. Response-rate versus pulse-frequency graph for rat Bechr21.

Fig S4. Response-rate versus pulse-frequency graph for rat Bechr26.

Fig S5. Response-rate versus pulse-frequency graph for rat Bechr27.

Fig S6. Response-rate versus pulse-frequency graph for rat Bechr28.

The data for rat Bechr29 are shown in Fig 4 in the main text.

Time allocation as a function of reward strength and cost

Fig S7. Time allocation as a function of reward strength and cost for rat Bechr14. A: Time allocation as a function of pulse frequency (reward strength) in the vehicle (upright triangles) and drug (inverted triangles) conditions. **B:** Time allocation as a function of price (opportunity cost) in the vehicle (squares) and drug (diamonds) conditions. In the radial-sweep condition, the pulse frequency was decreased and the price decreased concurrently, in stepwise fashion, over consecutive trials. Time allocation is plotted as a function of pulse frequency in panel **C:** and as a function of price in panel **D:**. Data from the vehicle condition are represented by circles, whereas data from the drug condition are represented by Stars of David. The error bars represent 95% confidence intervals.

Fig S8. Time allocation as a function of reward strength and cost for rat Bechr19.

Fig S9. Time allocation as a function of reward strength and cost for rat Bechr21.

Fig S10. Time allocation as a function of reward strength and cost for rat Bechr26.

Fig S11. Time allocation as a function of reward strength and cost for rat Bechr27.

Fig S12. Time allocation as a function of reward strength and cost for rat Bechr28.

The data for rat Bechr29 are shown in Fig 5 in the main text.

Derivation of the reward-mountain model

Acronyms and symbols employed in this supporting-information file are defined in Tab S1.

Table S1. Definition of acronyms and symbols

Acronym or Symbol	Definition
a	price-sensitivity exponent
BSR	brain stimulation reward
c	chronaxie of the strength-duration function for pulses (pulse duration at which the threshold current is twice rheobase)
C	chronaxie of the strength-duration function for trains (train duration at which $F_{firing_{hm}}$ is twice ρ_{Π})
C_r	Conditioned reward value
ChR2	channelrhodopsin-2
d	pulse duration
D_{burst}	duration of stimulation-induced burst of firing in the directly activated neurons
D_{train}	train duration (leading edge of first pulse to leading edge of final pulse)
DAPI	4′,6-diamidino-2-phenylindole
eICSS	electrical intracranial self-stimulation
f_a	function that determines the average reward rate derived from performance of alternate activities
f_D	function that relates the duration of the stimulation-induced burst of firing to the train duration
f_F	frequency-following function that relates the induced firing frequency to the pulse frequency
f_N	function that translates the pulse duration and current into the number of electrically recruited, directly stimulated neurons
f_p	subjective-probability function
f_P	subjective-price function
f_R	reward-growth function for eICSS or oICSS
f_T	behavioral-allocation function
f_U	function that translates the reward rate and subjective rate of exertion into a payoff
f_ϕ	subjective-effort function
F_{bend}	parameter governing the abruptness of the roll-off in frequency following
F_{firing}	firing frequency induced by electrical or optical stimulation
$F_{firing_{hm}}$	firing frequency that generates a reward of half-maximal intensity
$F_{firing_{max}}$	maximum firing frequency induced by electrical or optical stimulation
F_{hm}	notation for $F_{pulse_{hm}}$ used in previous eICSS papers
F^*_{hm}	shorthand notation for $F^*_{pulse_{hm}}$
F_{pulse}	pulse frequency in an electrical or optical pulse train

Table S1. Definition of acronyms and symbols

Acronym or Symbol	Definition
$F_{pulse_{hm}}$	pulse frequency required to drive reward intensity to half its maximum value
$F^*_{pulse_{hm}}$	estimated pulse frequency required to drive reward intensity to half its maximum value if frequency-following fidelity were perfect
F_{ro}	pulse frequency in the center of the roll-off region of the frequency-following function
g	exponent governing the steepness of reward-intensity growth
ICSS	intracranial self-stimulation
I	eICSS current
K_{aa}	constant scaling the value of alternate activities
K_{da}	constant representing the scaling of dopamine release
K_{ec_L}	effort-cost scalar for leisure activities
K_{ec_W}	effort-cost scalar for work
K_F	unit-translation constant to convert pulses s^{-1} into firings s^{-1} neuron^{-1}
K_{IS}	current-distance constant
K_{NS}	neuron-distance constant
K_{rg}	reward-intensity scalar
N	number of directly activated neurons
MFB	medial forebrain bundle
oICSS	optical intracranial self-stimulation
p_{obj}	objective probability that a reward will be delivered upon satisfaction of the response requirement
p_{sub}	subjective probability that a reward will be delivered upon satisfaction of the response requirement
P_e	notation for P_{obj_e} used in previous eICSS papers
P^*_e	shorhand notation for $P^*_{obj_e}$
P_{obj}	objective opportunity cost ("price") of a stimulation train
P_{obj_e}	objective price at which $\widehat{T} = 0.5$ when $\widehat{R} = \widehat{R}_{max}$
$P^*_{obj_e}$	estimated objective price at which $\widehat{T} = 0.5$ and $\widehat{R} = \widehat{R}_{max}$ if frequency-following fidelity were perfect
P_{sub}	subjective opportunity cost ("price") of a stimulation train
$\vec{P}_{sub_{\widehat{T}=0.5}}$	vector of subjective prices that hold time allocation midway between its minimum and maximum values
$P_{sub_{bend}}$	parameter controlling the abruptness of the transition from the "blade" to the "handle" of the subjective-price function
$P_{sub_{min}}$	minimal subjective price
$\dot{R}_{aa}$	average rate of reward from performance of alternate (leisure) activities
$\widehat{\dot{R}}_{aa}$	$\dot{R}_{aa}$ normalized to vary between 0 and 1
R_{bsr}	peak reward intensity achieved over the course of a stimulation train
$\widehat{R}_{bsr}$	R_{bsr} normalized to vary between 0 and 1 as R_{bsr} rises from 0 to $R_{bsr_{max}}$
$\widehat{R}_{bsr_{max}}$	maximum normalized reward intensity
$\vec{\widehat{R}}_{bsr_{\widehat{T}=0.5}}$	vector of normalized reward intensities that hold time allocation midway between its minimum and maximum values
$\dot{R}_{bsr}$	rate of brain-stimulation reward; reward intensity divided by the subjective price paid to procure the pulse train
T	time allocation
$\widehat{T}$	time allocation normalized to rise between 0 and 1 as T rises from T_{min} to T_{max}

Table S1. Definition of acronyms and symbols

Acronym or Symbol	Definition
T_{max}	maximal time allocation
T_{mid}	value of T midway between T_{min} and T_{max}; $T_{mid} = \widehat{T}_{0.5}$
T_{min}	minimal time allocation
TH	tyrosine hydroxylase
U_L	payoff from alternate ("leisure") activities (a.k.a "everything else")
U_W	payoff from brain stimulation reward
$\widehat{U}_W$	U_W normalized to vary between 0 and 1
YFP	(enhanced) yellow fluorescent protein
ρ_I	rheobase of the strength-duration function for pulses
ρ_Π	rheobase of the strength-duration function for trains
$\dot{\phi}_{obj_L}$	average work rate entailed in performing leisure activities
$\dot{\phi}_{sub_L}$	average rate of subjective exertion entailed in performing leisure activities
$\widehat{\dot{\phi}}_{sub_L}$	$\dot{\phi}_{sub_L}$ normalized to vary between 0 and 1
$\dot{\phi}_{obj_W}$	work rate entailed in holding down the lever
$\dot{\phi}_{sub_W}$	subjective rate of exertion entailed in holding down the lever

The reward-mountain model provides a framework for integrating the frequency-sweep, pulse-sweep, and radial-sweep data (e.g., Figure 5) in a unified 3D space and for interpreting the drug-induced changes in the position of the resulting 3D structure (the reward mountain). The following derivation of the model first extends earlier depictions that were developed in the context of eICSS studies [1–3] and then adapts the model to accommodate oICSS of midbrain dopamine neurons.

The reward-mountain model predicts time allocation given experimenter-controlled variables that determine the strength and cost of the rewarding stimulation. In most experiments carried out to date, the strength variable is the pulse frequency within a fixed-duration stimulation train, and the cost variable is the work time (opportunity cost, price) required to procure a stimulation train. The functional machinery that generates time-allocation values from these inputs is summarized in Tab S2.

Table S2. The functions composing the reward-mountain model. The shell → core functions (left column) map the values of variables that are manipulated or controlled into inputs to the core of the model. The core functions (middle column) map these inputs into the payoffs from work and leisure activities, whereas the core → shell function map the payoffs into the observed dependent variable: time allocation. The accompanying Matlab® Live Script illustrates how the listed functions are implemented. Please see Tab S1 for definitions of the symbols.

shell → core	core	core → shell
$F_{firing} = f_F(F_{pulse})$		
$N = f_N(I, d)$		
$D_{burst} = f_D(D_{train})$	$F_{firing_{hm}} = f_H(D_{burst},\ N)$	
	$R_{bsr} = f_R\left(F_{firing},\ F_{firing_{hm}}\right)$	
$p_{sub} = f_p\,(p_{obj})$		
$P_{sub} = f_P(P_{obj})$		
$\dot{\phi}_{sub_W} = f_\phi\left(\dot{\phi}_{obj_W}\right)$	$U_W = f_U\left(\frac{R_{bsr}}{P_{sub}},\ p_{sub},\ \dot{\phi}_{sub_W}\right)$	
$\_ = f_a\,(\_,\ \_,\ \cdots\ \_)$	$\dot{R}_{aa} = f_R\,(\_)$	
$\dot{\phi}_{sub_L} = f_\phi\left(\dot{\phi}_{obj_L}\right)$	$U_L = f_U\left(\dot{R}_{aa},\ \dot{\phi}_{sub_L}\right)$	$T = f_T\,(U_W,\ U_L)$

As the table implies, we distinguish between the "shell" and "core" of the model. The shell consists of the variables that are observed (time allocation), manipulated (pulse frequency, price), and controlled (stimulation parameters held constant, physical work required to hold down the lever, affordances of the test environment [4]). The shell is displayed within the space defined by the observed and manipulated variables.

The core (middle column of Tab S2) consists of the functions that compute the intensity of the reward produced by the stimulation train and combine this value with the opportunity and effort costs to generate what we call "payoffs." Parallel functions in the core compute the value of the alternate activities that compete with pursuit of the stimulation for the rat's behavior. A set of functions (left column) provides the input to the core by mapping the manipulated and controlled variables into the quantities from which payoffs are derived.

A single function (right column), based on the generalized matching law [5], translates the payoffs generated in the core into the time-allocation values that are manifested in the shell.

Shell variables are objective, whereas core variables are inferred subjective quantities. The core functions are the bridge between the objective inputs that are manipulated or controlled to the observed objective output, time allocation; their form and parameters explain why the manipulated variables cause time allocation to vary in the manner observed in the experiment.

Shell → core functions

The arguments of the first three shell → core functions are the four parameters that define a fixed-frequency pulse train: the pulse frequency (F_{pulse}), current (I), pulse duration (d), and train duration (D_{train}). These functions relay to the core functions the stimulation-induced frequency of firing (F_{firing}), the number of activated neurons (N), and the duration of the burst of increased firing (D_{burst}).

$\boldsymbol{f_F}$: In studies of eICSS, the experimenter typically varies the pulse frequency during a fixed-duration stimulation train in order to control the intensity of the electrically-induced reward. The frequency-following function labeled $\boldsymbol{f_F}$ maps the manipulated shell variable, F_{pulse} into the corresponding core variable: the induced frequency of firing in the directly-stimulated substrate, F_{firing}. In the case of eICSS of the MFB, this function has been estimated by

psychophysical means and shown to be roughly scalar up to very high pulse frequencies [6], well beyond the typical range of the $F_{pulse_{hm}}$ values that locate the reward-mountain shell in the space defined by the two independent variables. Solomon et al. [6] showed that the following function provides a good fit to the frequency-following data for eICSS of the MFB:

$$F_{firing} = f_F(F_{pulse}) = K_F \times F_{bend} \times \left(\ln\left[1 + e^{\frac{F_{ro}}{F_{bend}}}\right] - \ln\left[1 + e^{\frac{F_{ro} - F_{pulse}}{F_{bend}}}\right] \right) \quad \text{(S1)}$$

where

$f_F =$ the frequency-following function

$F_{bend} =$ parameter determining the abruptness of the roll-off in the frequency response; units: $unitless$

$F_{firing} =$ induced firing rate in the first-stage neurons; units: $firings\ s^{-1}\ neuron^{-1}$

$F_{pulse} =$ the pulse frequency; units: $pulses\ s^{-1}$

$F_{ro} =$ the pulse frequency in the center of the roll-off region; units: $pulses\ s^{-1}$

$K_F =$ unit-translation constant; units: $firings\ pulse^{-1}\ neuron^{-1}$

A plot of the frequency-following function is shown in Fig S13. The form of the function is the same as the one described by Solomon et al. [6]. The choice of parameters is described below in section Parameters of the frequency-following function for oICSS.

Fig S13. Assumed frequency-following function for optical stimulation of midbrain dopamine neurons. The induced firing frequency is plotted as a function of the optical pulse frequency. Note that frequency-following fidelity is increasingly poor as the pulse frequency increases. At low values, the firing frequency falls only slightly short of the pulse frequency, but by 40 pulses s^{-1}, the firing frequency is only ~80% of the pulse frequency. The maximum induced firing frequency is 51.6 spikes s^{-1}

f_N: The F_N function translates the pulse duration and current into the number of electrically excited neurons. These two variables determine conjointly the boundary of the region in which the stimulation excites reward-related neurons. Holding these variables constant, as is the case in most eICSS studies entailing measurement of the reward mountain and in prior studies employing the curve-shift method [7–9], circumvents the need to make assumptions about the spatial distribution of the directly-stimulated neurons subserving the rewarding effect and about their excitability to extracellular stimulation.

Hawkins (cited in [10]) proposed that the number of directly-stimulated ("first-stage") neurons subserving the rewarding effect is roughly proportional to the current, when pulse duration is held constant. Emprical studies [11, 12] show that the current required to excite a given first-stage neuron varies roughly as a rectangular, hyperbolic function of the pulse duration. Thus,

$$N = f_N(d, I) = \frac{K_{N_S}}{K_{I_S}} \times \frac{I}{\rho_I \times \left(1 + \frac{c}{d}\right)} \tag{S2}$$

where

$c =$ chronxie; pulse duration (units: ms) for which the threshold current is twice ρ_I

$d =$ pulse duration (units: ms)

$f_N =$ the first-stage recruitment function

$I =$ current (units: μA)

$K_{N_S} =$ neuron-distance constant; units: $neurons\ mm^{-2}$

$K_{I_S} =$ current-distance constant; units: $\mu A\ mm^{-2}$

$N =$ number of activated first-stage neurons; units: $neurons$

$\rho_I =$ threshold current required to excite a first-stage neuron using a pulse of infinite duration; units: μA

(S3)

$\boldsymbol{f_D}$: The third stimulation parameter that has been held constant in most reward-mountain and curve-shift studies is the duration of the stimulation train. The f_D function translates the duration set by the experimenter into the duration of the stimulation-induced increase in the activity of the directly-stimulated substrate. If firing is time-locked to the stimulation pulses, then the duration of this increase, D_{burst}, will equal the train duration, D_{train}. Thus, we assume that

$$D_{burst} = f_D(D_{train}) = D_{train} \tag{S4}$$

where

$D_{burst} =$ duration of the stimulation-induced increase in firing above baseline in the directly stimulated neurons subserving the reward effect

$D_{train} =$ time interval between the leading edges of the first and last pulse in a stimulation train

$f_D =$ the duration-mapping function

$\boldsymbol{f_p}$: Psychophysical methods have been used to describe the subjective-probability function for BSR, f_p (lower-case subscript) [13]. This function returns the subjective probability that a reward will be delivered upon payment of the price set by the experimenter. Over the range, 0.5 - 1.0, this function was determined to be roughly scalar. In the present study and in all other studies that have entailed measurement of the reward mountain, delivery of the reward upon satisfaction of the response requirement is certain ($p_{obj} = 1$).

$$p_{sub} = f_p(p_{obj}) = p_{obj} \tag{S5}$$

for $0.5 \leq p_{obj} \leq 1$

where

$f_p =$ the subjective-probability function

$p_{obj} =$ objective probability that reward will be delivered upon satisfaction of the response requirement

$p_{sub} =$ subjective probability that reward will be delivered upon satisfaction of the response requirement

$\boldsymbol{f_P}$: Past measurements of the reward-mountain in eICSS studies employed the work time required to trigger delivery

of a stimulation train (the price) as the cost variable, and that practice is continued here. The function labeled f_P (upper-case subscript) maps the required work time, P_{obj}, into the corresponding subjective variable, P_{sub}. The form and parameters of this function for eICSS of the MFB have been estimated by psychophysical means [14]. We showed that the following psychophysical function more accurately describes the opportunity cost of rewarding brain stimulation than the identity function or functions based either on hyperbolic or exponential discounting:

$$P_{sub} = f_P\left(P_{obj}\right) = P_{sub_{min}} + P_{sub_{bend}} \times \ln\left(1 + \mathrm{e}^{\left[\frac{P_{obj} - P_{sub_{min}}}{P_{sub_{bend}}}\right]}\right) \tag{S6}$$

where

$f_P =$ the subjective-price function

$P_{obj} =$ the "price" of a stimulation train: the cumulative time the lever must be depressed to trigger reward delivery; units: s

$P_{sub} =$ the subjective price of a stimulation train; units: s

$P_{sub_{min}} =$ the minimum subjective price; units: s

$P_{sub_{bend}} =$ a constant that controls the abruptness of the transition. from "blade" to "handle;" unitless

For a different view of the subjective-price function, please see [15].

At higher prices, the output of the subjective-price function defined by Eq S6 converges on its input: the subjective price becomes indistinguishable from the objective one. However, as the objective price is reduced below ∼3 s, the subjective price deviates from the objective price and eventually approaches an asymptotic value: $P_{sub_{min}}$. This asymptote has been interpreted to arise from the reduction and eventual disappearance of competition between lever depression and competing activities, such as grooming, resting, and exploring; performance of these competing activities is no longer perceived as beneficial once the available time for their execution becomes sufficiently short.

A plot of the subjective-price function is shown in Fig S14. The parameters employed are the mean values determined by Solomon et al. [14].

Fig S14. Subjective opportunity-cost ("price") function. The function maps the objective opportunity cost (cumulative lever-depression time required to trigger reward delivery), P_{obj}),into its subjective equivalent P_{sub}. The form and parameters of this function are based on measurements by Solomon et al. [6].

The arguments of the first five shell → core functions are all variables controlled directly by the experimenter: $\{F_{pulse}, d, I, D, p, P\}$. These six variables are transformed by the first five shell → core functions into inputs to the core. The arguments of the remaining two shell → core functions listed in Tab S2 are variables arising from features of the test environment that the experimenter attempts to hold constant: the rate of physical work entailed in holding down the lever or in performing activities that compete with pursuit of the rewarding stimulation.

$\boldsymbol{f_\phi}$: The effort cost of the reward is the subjective rate of exertion entailed in holding down the lever. We know of no psychophysical studies that reveal the form of this function. That said, we can assume that it accelerates steeply as the effort cost approaches the physical capabilities of the rat. The objective effort cost has been held constant in this and in prior studies carried out in the reward-mountain paradigm. We assume that the subjective effort cost did not co-vary with the manipulated variables. To help ensure this in the current study, the lever was withdrawn for 2 s following the triggering of a stimulation train so as to provide time for any interfering motoric consequences of the stimulation to dissipate.

We treat the subjective rate of exertion required to depress the lever as a constant defined by:

$$\dot{\phi}_{sub_W} = f_\phi\left(\dot{\phi}_{obj_W}, K_\phi\right) \tag{S7}$$

where

f_ϕ = subjective-effort function (form unknown)

$\dot{\phi}_{obj_W}$ = rate of physical work required to hold down the lever; units: Js^{-1}

$\dot{\phi}_{sub_W}$ = subjective rate of exertion required to hold down the lever in units we call "*oomphs*" s^{-1}

K_ϕ = unit-conversion constant; units: *oomphs* J^{-1}

The dots over $\dot{\phi}_{obj_W}$ and $\dot{\phi}_{sub_W}$ signify that we define these quantities as rates. (No dots are placed over F_{firing} and F_{pulse} because doing so would be superfluous and potentially misleading. Frequencies are inherently rates over time (the first time derivative of the pulse or spike number). By omitting the dots, we wish to avoid confusion between these rates and their changes over time (the second derivative of the pulse or spike number).)

The effect of the drug, if any, on the rate of subjective exertion is defined as:

$$\dot{\phi}_{sub_{W_{drug}}} = \dot{\phi}_{sub_{W_{vehicle}}} \times K_{ec_W} \tag{S8}$$

where

K_{ec_W} = proportional drug-induced change in the subjective rate of exertion required to hold down the lever; unitless

In the vehicle condition, K_{ec_W} assumes an implicit value of one.

Activities such as grooming and exploring also entail performance of physical work. Thus, f_ϕ is also applied to these activities:

$$\dot{\phi}_{sub_L} = f_\phi\left(\dot{\phi}_{obj_L}, K_\phi\right) \tag{S9}$$

where

$\dot{\phi}_{obj_L}$ = Average rate of physical work required to perform alternate ("leisure") activities; units: Js^{-1}.

$\dot{\phi}_{sub_L}$ = average subjective rate of exertion entailed in performance of leisure activities in *oomphs* s^{-1} .

K_ϕ = unit-conversion constant; units: *oomphs* J^{-1}

We assume that $\dot{\phi}_{sub_L}$ does not covary systematically with the independent variables.

As in the case of the subjective rate of exertion entailed in work, we allow for drug-induced modulation of the subjective rate of exertion entailed in performance of leisure activities.

$$\dot{\phi}_{sub_{L_{drug}}} = \dot{\phi}_{sub_{L_{vehicle}}} \times K_{ec_L} \tag{S10}$$

where

K_{ec_L} = proportional drug-induced change in the subjective rate of exertion required to hold down the lever; unitless

In the vehicle condition, K_{ec_L} assumes an implicit value of one.

$\boldsymbol{f_a}$: We do not know which aspects of the leisure activities that compete with pursuit of BSR give rise to reward signals in the brain nor how these signals are encoded. That is why both the arguments and output of the seventh

shell → core function (f_a) have been left blank. However, we do know that valuation of these activities influences the allocation of time to pursuit of experimenter-controlled rewards: enrichment of the test environment shifts allocation towards alternate activities and away from the experimenter-controlled reward [16]. Thus a function such as f_a must exist. We include f_a in the list of shell → core functions for completeness and in recognition of this requirement.

Core functions

Core functions determine the reward rates produced by work (lever depression) and leisure (alternate) activities. These are combined with the associated effort costs to yield a pair of payoffs, which are then passed to the core → shell function for translation into time allocation.

$\boldsymbol{f_R}$: The core receives a set of spike trains as a result of the combined action of the first three shell → core functions $\{f_F, f_N, f_D\}$, one spike train from every activated first-stage neuron. According to the counter model [10, 11, 17], the effects of the spike trains delivered by the individual first-stage neurons are summed, and thus, the intensity of the rewarding effect is determined by the product of the number of activated first-stage neurons and the rate at which they are fired by the stimulation train. This is why a Π symbol is used in the flow diagrams to represent the drive produced by a pulse train of fixed duration on the scalar at the input of the reward-growth function (Fig 3).

The logistic form of the reward-growth function was described originally in operant-matching studies carried out by Gallistel's group [18–20]. Shizgal [21] proposed the following expression for this function:

$$R_{bsr} = f_R\left[f_F(F_{pulse})\right] = \widehat{R}_{bsr} \times K_{rg} = \left(\frac{{F_{firing}}^g}{{F_{firing}}^g + {F_{firing_{hm}}}^g}\right) \times K_{rg} \tag{S11}$$

where

$f_R =$ the reward-growth function

$F_{firing_{hm}} =$ firing rate required to drive reward intensity to half its maximum value; units: $firings\ neuron^{-1}\ s^{-1}$
$F_{firing_{hm}} = f_F\left(F_{pulse_{hm}}\right)$

$F_{pulse_{hm}} =$ pulse frequency required to drive reward intensity to half its maximum value; units: $pulses\ s^{-1}$

$g =$ the exponent that determines the steepness of reward-intensity growth as a function of pulse frequency

$K_{rg} =$ reward-growth scalar; units: *hedons*

$R_{bsr} =$ reward intensity produced by F_{firing}; units: *hedons*

$\widehat{R}_{bsr} =$ normalized reward intensity produced by a pulse frequency of F_{pulse}, which, in turn, produces a firing frequency of F_{firing} in each first-stage neuron; unitless. $0 \le \widehat{R}_{bsr} \le 1$

According to Eq S11,

$$\widehat{R}_{bsr} = \frac{{F_{firing}}^g}{{F_{firing}}^g + {F_{firing_{hm}}}^g} \tag{S12}$$

When frequency-following fidelity is sufficiently high, the normalized reward intensity $\left(\widehat{R}_{bsr}\right)$ will approach one at high pulse frequencies. The lower the value of the location parameter $\left(F_{firing_{hm}}\right)$ and the higher the value of the reward-growth exponent (g), the easier this will be to achieve.

To accommodate the predicted rescaling of the input to the reward-growth function for oICSS by dopamine-transporter blockade, a scalar, K_{da}, is added to Eq S11 in the drug condition of the experiment:

$$
\begin{aligned}
R_{bsr_{drug}} &= \left[\frac{\left(F_{firing_{drug}} \times K_{da}\right)^{g}}{\left(F_{firing_{drug}} \times K_{da}\right)^{g} + \left(F_{firing_{hm_{vehicle}}}\right)^{g}} \right] \times K_{rg} \\
&= \left[\frac{\left(F_{firing_{drug}}\right)^{g}}{\left(F_{firing_{drug}}\right)^{g} + \left(\dfrac{F_{firing_{hm_{vehicle}}}}{K_{da}}\right)^{g}} \right] \times K_{rg}
\end{aligned}
\tag{S13}
$$

where

K_{da} = scalar representing the boost in dopamine release due to transporter blockade

Thus,

$$
F_{firing_{hm_{drug}}} = \frac{F_{firing_{hm_{vehicle}}}}{K_{da}} \tag{S14}
$$

Dopamine-transporter blockade boosts dopamine release, thereby increasing the impact of each firing. The increased value of the scalar (K_{da}) captures this augmented impact, reducing the value of the location parameter of the reward-growth function in the drug condition. Fewer pulses per train are required to produce a reward of a given intensity when K_{da} increases. Consequently, the reward-growth function shifts leftwards along the pulse-frequency axis. In the simulations of the vehicle condition, K_{da} is assigned an implicit value of one.

Division by the subjective price transforms the reward intensity into a reward rate:

$$
\dot{R}_{bsr} = \frac{R_{bsr}}{P_{sub}} \tag{S15}
$$

where

$\dot{R}_{bsr}$ = experienced rate of brain stimulation reward; units: $hedons\ s^{-1}$

$\boldsymbol{f_H}$: The location parameter of the reward-growth function is the firing rate that drives reward intensity to half its maximal value. The value of this parameter depends on the number of stimulated first-stage neurons, N and the interval during which the stimulation train elevates their firing rate, D_{burst}. A prior study of temporal integration in the neural circuitry responsible for eICSS of the MFB [21] implies the following form for the function that determines $F_{firing_{hm}}$:

$$
F_{firing_{hm}} = f_H\left(D_{burst},\ N\right) = \frac{\rho_{\Pi} \times \left(1 + \dfrac{C}{D_{burst}}\right)}{N} \tag{S16}
$$

where

C = chronaxie: train duration at which $F_{firing_{hm}}$ is twice the value of ρ_{Π}; units: s

ρ_{Π} = aggregate rate of firing required to produce a reward of half-maximal intensity when the train duration is infinite; units: $firings\ s^{-1}$

$\boldsymbol{f_U}$: In keeping with the generalized matching law [22], the benefit from work and its costs are combined in scalar fashion to yield a net payoff. We can format the expression for the payoff as a ratio of two rates: the

probability-weighted rate of brain-stimulation reward and the subjective rate of exertion required to hold down the lever (see [23]):

$$U_W = f_U\left(\frac{R_{bsr}}{P_{sub}},\ p_{sub},\ \dot{\phi}_{sub_W}\right) = \frac{\frac{R_{bsr}}{P_{sub}} \times p_{sub}}{\dot{\phi}_{sub_W}} = \frac{\dot{R}_{bsr} \times p_{sub}}{\dot{\phi}_{sub_W}} \tag{S17}$$

where

$f_U =$ utility function

$U_W =$ payoff from a train of rewarding stimulation; units: *hedons oomph*$^{-1}$

or as a benefit/cost ratio:

$$U_W = f_U\left(\frac{R_{bsr}}{P_{sub}},\ p_{sub},\ \dot{\phi}_{sub_W}\right) = \frac{R_{bsr} \times p_{sub}}{P_{sub} \times \dot{\phi}_{sub_W}} \tag{S18}$$

In the case of BSR, there is solid evidence that aggregate impulse flow in the first-stage neurons encodes the signal that will be translated into the intensity of the reward [17,20]. Although the identity of the first-stage neurons subserving eICSS of the MFB (or any other brain site) remains unknown, directly-driven MFB neurons with properties that match the psychophysically derived portrait of the first-stage fibers have been observed by means of electrophysiological recording [24–26].

The argument of the reward-growth function for leisure activities is left blank, thus signifying our ignorance of how pursuit of these activities is encoded by the brain and translated into a reward rate. That said, the considerable evidence that the value of leisure activities competes effectively with experimenter-controlled rewards [5,16,27–29] implies that the payoffs from work and leisure are commensurable. Accordingly, we define a reward rate for the leisure activities that compete with pursuit of BSR:

$$\dot{R}_{aa} = f_R\left(\_\right) = \dot{\hat{R}}_{aa} \times K_{aa} \tag{S19}$$

where

$K_{aa} =$ alternate-activity scalar; units: *hedons* s^{-1}

$\dot{R}_{aa} =$ average rate of reward from alternate (leisure) activities that compete with pursuit of BSR; units: *hedons* s^{-1}

$\dot{\hat{R}}_{aa} =$ normalized rate of reward from alternate (leisure) activities; unitless. $0 \leq \dot{\hat{R}}_{aa} \leq 1$

$(\_) =$ the unknown variables that give rise to $\dot{R}_{aa}$

The payoff from these alternate activities is computed in a manner analogous to the computation of the payoff from BSR, as a ratio of reward and subjective-exertion rates:

$$U_L = f_U\left(\dot{R}_{aa},\ \dot{\phi}_{sub_L}\right) = \frac{\dot{\hat{R}}_{aa} \times K_{aa}}{\dot{\phi}_{sub_L}} \tag{S20}$$

where

$U_L =$ payoff from leisure activities; units: *hedons oomph*$^{-1}$

The core → shell function

***f*$_T$**: The payoffs from pursuit of BSR (U_W) and engagement in leisure activities (U_L) are used by the sole core → shell function to compute the allocation of time to pursuit of BSR. This behavioral-allocation function is derived from the single-operant matching law [5, 30, 31]:

$$T = T_{min} + \left[(T_{max} - T_{min}) \times \frac{(U_W)^a}{(U_W)^a + (U_L)^a} \right] \tag{S21}$$

where

a = price-sensitivity exponent; unitless

T = time allocation; unitless

T_{max} = maximum time allocation; unitless

T_{min} = minimum time allocation; unitless

U_W = payoff from pursuit of rewarding brain stimulation ("work"); units: *hedons oomph*$^{-1}$

U_L = payoff from pursuit of alternate ("leisure") activities; units: *hedons oomph*$^{-1}$

Even when the rat is working maximally, latency to depress the lever is typically greater than zero, and T_{max} is thus typically less than one. The rat tends to sample the lever at trial onset, even when the payoff from brain stimulation is low. Thus, T_{min} is typically greater than zero.

The single-operant matching law was formulated initially to account for the behavior of subjects working on variable-interval schedules of reinforcement. The cumulative handling-time schedule in force in the present study [32] is more akin to a fixed-ratio schedule in that the number of rewards earned is strictly proportional to time worked. On such schedules, time allocation shifts more abruptly than on variable-interval schedules as the value of the experimenter-controlled reward is varied. In the original version of the single-operant matching law [30, 31], the payoff terms are not exponentiated. In contrast, the price-sensitivity exponent (a) in Eq S21 allows the reward-mountain model to account for time-allocation shifts of varying abruptness.

To simplify the remaining derivation of the reward-mountain model, we define a normalized measure of time allocation:

$$\widehat{T} = \frac{(U_W)^a}{(U_W)^a + (U_L)^a} \tag{S22}$$

$$\text{where} \quad 0 \le \widehat{T} \le 1$$

Substituting from Eq S22 in Eq S21, we obtain

$$T = T_{min} + \left[(T_{max} - T_{min}) \times \widehat{T} \right] \tag{S23}$$

$$\widehat{T} = \frac{T - T_{min}}{T_{max} - T_{min}} \tag{S24}$$

Time allocation as a function of reward strength and cost

We are now in a position to tie the dependent variable, time allocation (T), to the two independent variables, pulse frequency (F_{pulse}) and price (P_{obj}). Substitution for U_W and U_L from Eqs S17 and S20, respectively, in Eq S22 yields:

$$\widehat{T} = \frac{\left(\dfrac{[\widehat{R}_{bsr} \times K_{rg}] \times p_{sub}}{[\dot{\widehat{\phi}}_{sub_W} \times K_{ec_W}] \times P_{sub}}\right)^a}{\left(\dfrac{[\widehat{R}_{bsr} \times K_{rg}] \times p_{sub}}{[\dot{\widehat{\phi}}_{sub_W} \times K_{ec_W}] \times P_{sub}}\right)^a + (U_L)^a} \tag{S25}$$

An initial step towards simplifying Eq S25 is to multiply each of the terms on the right by

$$\left(\frac{K_{ec_W}}{K_{rg}}\right) \times \left(\frac{\dot{\widehat{\phi}}_{sub_W} \times P_{sub}}{p_{sub}}\right)$$

This yields:

$$\widehat{T} = \frac{(\widehat{R}_{bsr})^a}{(\widehat{R}_{bsr})^a + \left[\left(\dfrac{K_{ec_W} \times K_{aa}}{K_{rg}}\right) \times \left(\dfrac{\dot{\widehat{\phi}}_{sub_W} \times (U_L)^a}{p_{sub}}\right) \times P_{sub}\right]^a} \tag{S26}$$

To simplify Eq S25 further, we first define T_{mid} as the time-allocation value midway between maximal and minimal time allocation:

$$T_{mid} = T_{min} + \left(\frac{T_{max} - T_{min}}{2}\right) \tag{S27}$$

According to Eqs S22 and S23,

$$\begin{gathered}\text{when } T = T_{mid}\\ \widehat{T} = 0.5\\ \text{and}\\ U_W = U_L\end{gathered} \tag{S28}$$

We now hold time allocation at T_{mid} and drive reward intensity to its maximum value $\left(\widehat{R}_{bsr} = \widehat{R}_{bsr_{max}}\right)$. Substituting for U_W from Eq S17 and reversing Eq S28, we obtain

$$U_L = \frac{[\widehat{R}_{bsr_{max}} \times K_{rg}] \times p_{sub}}{[\dot{\widehat{\phi}}_{sub_W} \times K_{ec_W}] \times P_{sub_e}(P_{obj_e})} \tag{S29}$$

where

P_{obj_e} = objective price at which $T = T_{mid}$ when $\widehat{R}_{bsr} = \widehat{R}_{bsr_{max}}$

P_{sub_e} = subective price at which $T = T_{mid}$ when $\widehat{R}_{bsr} = \widehat{R}_{bsr_{max}}$

Rearranging the terms yields

$$P_{sub_e} = \frac{K_{rg}}{K_{aa} \times K_{ec_W}} \times \frac{p_{sub} \times \widehat{R}_{bsr_{max}}}{\dot{\widehat{\phi}}_{sub_W} \times U_L} \tag{S30}$$

For consistency with previous papers, we use the subscript, "e" in the symbol P_{sub_e} to refer to the fact that the payoff from brain stimulation equals the payoff from alternate activities ("everything else") when the normalized reward intensity is maximal ($\widehat{R}_{bsr} = \widehat{R}_{bsr_{max}}$) and the subjective price (P_{sub}) equals P_{sub_e}. This equivalence between the two competing payoffs is what drives time allocation to the half-way point (T_{mid}) between its minimal (T_{min}) and maximal (T_{max}) values.

Rearranging Eq S30, we obtain:

$$\frac{\widehat{R}_{bsr_{max}}}{P_{sub_e}} = \left[\left(\frac{K_{aa} \times K_{ec_W}}{K_{rg}} \right) \times \left(\frac{\dot{\widehat{\phi}}_{sub_W} \times U_L}{p_{sub}} \right) \right] \tag{S31}$$

Substituting in Eq S26 from Eqs S11, S24, and S31, we obtain

$$\widehat{T} = \frac{(\widehat{R}_{bsr})^a}{(\widehat{R}_{bsr})^a + \left[\widehat{R}_{bsr_{max}} \times \left(\frac{P_{sub}}{P_{sub_e}} \right) \right]^a} \tag{S32}$$

By setting $\widehat{R}_{bsr} = \widehat{R}_{bsr_{max}}$ and $P_{sub} = P_{sub_e}$ in Eq S32, it can be seen readily that $\widehat{T} = 0.5$, thus satisfying the definition of P_{sub_e} as the price at which time allocation to pursuit of a maximally intense reward is halfway between T_{min} and T_{max}.

P_{sub} and $\widehat{R}_{bsr}$ must trade off to hold $\widehat{T}$ at a given level. The lowest attainable subjective price is the value corresponding to an objective price of zero, which we will call P_{sub_0}. (Negative values of P_{obj} may be required to drive P_{sub} to $P_{sub_{min}}$.) Consider a vector of subjective prices, $\vec{P}_{sub_{\widehat{T}}}$, that extends from P_{sub_0} to the highest tested value of P_{sub} and a corresponding vector of normalized reward intensities, $\vec{\widehat{R}}_{bsr_{\widehat{T}}}$, that hold normalized time allocation ($\widehat{T}$) constant over $\vec{P}_{sub}$. If follows from Eq S32 that

$$\frac{\vec{\widehat{R}}_{bsr_{\widehat{T}}}}{\vec{P}_{sub_{\widehat{T}}}} = \left(\frac{\widehat{T}}{1-\widehat{T}}\right)^{\frac{1}{a}} \times \left(\frac{\widehat{R}_{bsr_{max}}}{P_{sub_e}}\right) \tag{S33}$$

$$0 \leq \vec{\widehat{R}}_{bsr_{\widehat{T}}} \leq \widehat{R}_{bsr_{max}} \leq 1$$

$$0 < P_{sub_0} \leq P_{sub_{\widehat{T}}}$$

where

$P_{sub_0} =$ the subjective price corresponding to an objective price of zero

The higher the subjective price, the higher the normalized reward intensity required to hold time allocation constant. When $\widehat{T} = 0.5$, Eq S33 reduces to:

$$\frac{\vec{\widehat{R}}_{bsr_{\widehat{T}=0.5}}}{\vec{P}_{sub_{\widehat{T}=0.5}}} = \frac{\widehat{R}_{bsr_{max}}}{P_{sub_e}} \tag{S34}$$

$$0 \leq \widehat{R}_{bsr_{\widehat{T}=0.5}} \leq \widehat{R}_{bsr_{max}} \leq 1$$

$$0 < P_{sub_0} \leq P_{sub_{\widehat{T}=0.5}} \leq P_{sub_e}$$

Below (see: Contour lines: the trade-off between pulse frequency and price to hold time allocation constant), we use Eqs S33 and S34 to obtain the equation for the contour lines that provide a two-dimensional description of the reward-mountain surface.

To complete the derivation of the reward-mountain model, we now substitute for $\widehat{T}$ in Eq S23 and expand Eq S32 so that time allocation is expressed in terms of the independent variables: price (P_{obj}) and pulse frequency (F_{pulse}), which appear as the arguments of the subjective-price and frequency-following functions, respectively:

$$T = T_{min} + (T_{max} - T_{min}) \times \frac{\left(\frac{f_F\,(F_{pulse})^{\,g}}{f_F(F_{pulse})^g + f_F(F_{pulse_{hm}})^g}\right)^a}{\left(\frac{f_F(F_{pulse})^g}{f_F(F_{pulse})^g + f_F(F_{pulse_{hm}})^g}\right)^a + \left(\widehat{R}_{bsr_{max}} \times \left[\frac{f_P(P_{obj})}{f_P(P_{obj_e})}\right]\right)^a} \tag{S35}$$

The conditioned-reward variant of the reward-mountain model

The six-parameter version of the reward-mountain model incorporates Eqs S1, S6, and S35. The fitted parameters are a (the price-sensitivity exponent), $F_{pulse_{hm}}$ (the pulse frequency at which reward intensity is half maximal), g (the reward-growth exponent) P_{obje} (the price at which time allocation to pursuit of a maximal reward falls midway between its minimal and maximal values), T_{min} (minimum time allocation), and T_{max} (maximal time allocation). The seven-parameter version includes an additional parameter, C_r, to reflect conditioned reward. This seventh parameter reflects a learned value, above and beyond the payoff from the stimulation train, associated with the lever and/or the act of holding it down. The paper that introduced this parameter [3] incorporated it into the reward-growth function as follows:

$$\widehat{R}_{bsr} = \frac{{F_{pulse}}^{g} + Cr^{g}}{{F_{pulse}}^{g} + {F_{pulse_{hm}}}^{g} + Cr^{g}} \tag{S36}$$

where

C_r = the conditioned reward, expressed in terms of the equivalent pulse frequency

Note that the way the C_r parameter was incorporated into Eq S36 causes this parameter to interact with both the a (Eq S35) and g (Eq S36) parameters. That form of the model failed to yield consistently converging fits when applied to the current dataset. To address this problem, we altered the way that the C_r parameter is incorporated into the reward-growth function (Eq S11) so as to reduce its interaction with the other parameters:

$$\widehat{R}_{bsr} = C_r + \left[\left(1 - \frac{C_r}{\widehat{R}_{bsr_{max}}}\right) \times \left(\frac{f_F(F_{pulse})^g}{f_F(F_{pulse})^g + f_F(F_{pulse_{hm}})^g}\right)\right] \tag{S37}$$

The resulting reward-mountain surface is produced by substituting the expression for $\widehat{R}_{bsr}$ from Eq S37 in Eq S32, as follows:

$$T = T_{min} + (T_{max} - T_{min}) \times \frac{\left(C_r + \left[\left(1 - \frac{C_r}{\widehat{R}_{bsr_{max}}}\right) \times \left(\frac{f_F(F_{pulse})^g}{f_F(F_{pulse})^g + f_F(F_{pulse_{hm}})^g}\right)\right]\right)^a}{\left(C_r + \left[\left(1 - \frac{C_r}{\widehat{R}_{bsr_{max}}}\right) \times \left(\frac{f_F(F_{pulse})^g}{f_F(F_{pulse})^g + f_F(F_{pulse_{hm}})^g}\right)\right]\right)^a + \left(\widehat{R}_{bsr_{max}} \times \left[\frac{f_P(P_{obj})}{f_P(P_{obj_e})}\right]\right)^a} \tag{S38}$$

As shown in Fig S15, Eq S38 yields a reward-mountain surface that is all but indistinguishable from the surface generated by the equation in the 2010 paper. Unlike the equation in the 2010 paper, Eq S38 produced well-behaved, converging fits.

Fig S15. Surface and contour plots of two seven-parameter reward-mountain models. The new version of the model produces a surface that is nearly identical to the one generated by the 2010 version of this model [3]. Fits of the 2010 model to the current datasets failed to converge, whereas fits of the new version of the model converged in all cases.

Adaptation of the reward-mountain model for oICSS

Fig S16. The reward-mountain model for oICSS. A graphical summary of the reward-mountain model, as adapted for oICSS of midbrain dopamine neurons. The symbols are defined in Tab S1.

The reward-mountain surface shows the observed behavioral output (time allocation, T) as a function of the two independent variables, the price (P_{obj}) and pulse frequency (F_{pulse}). These three variables constitute the shell of the reward-mountain model, together with the controlled variables: the work rate required to hold down the lever ($\dot{\phi}_{obj_W}$), the leisure activities afforded by the test environment, and the average work rate required to perform these activities ($\dot{\phi}_{obj_L}$). The shell → core functions, $\{f_F, f_p, f_P, f_\phi, f_a\}$, map the manipulated and controlled variables into the core quantities that determine the payoffs from work and leisure. These mapping functions are shown at the left of the figure. The core functions that compute the reward intensities and corresponding payoffs $\{f_R, f_U\}$ are shown in the center. (f_H, the function that determines the location parameter of the reward-growth function, is not shown, nor are f_D, the function that determines the duration of the stimulation-induced burst of firing and f_N, the

function that determines the number of recruited neurons (N).) The core → shell function, (f_T), translates the payoffs from work and leisure into time allocation. It is shown on the right.

Minimal changes were made to adapt the model prior to fitting the reward-mountain surface to the data:

1. New values were used for the parameters of the frequency-following function (Eq S1) so as to accommodate the known properties of midbrain dopamine neurons and the optical-power versus pulse-frequency trade-off data reported here.
2. In eICSS, the current and pulse duration conjointly determine the number of directly stimulated neurons. In oICSS, optical power plays the role assumed by current in eICSS. However, there is insufficient information available to model f_N, the function that determines the number of neurons directly activated by a given optical power and pulse duration. Instead, we simply chose values of N and ρ_Π that produce simulated reward-mountain surfaces similar to the ones returned by the fits of the model to the data. (See the accompanying Matlab® Live Script.)
3. $\widehat{R}_{bsr_{max}}$ was included explicitly in the reward-mountain model, thus expanding the model to accommodate imperfect frequency-following fidelity.
4. Explicit inclusion of $\widehat{R}_{bsr_{max}}$ required correction of the location parameters and a more nuanced treatment of drug-induced shifts in the location of the mountain. (See: Displacement of the shell: distinguishing two sources.)
5. The new conditioned-reward variant described above (Eq S38) was used in lieu of the version introduced in our prior studies of the effect of cocaine and GBR-12909 on reward mountains obtained in the eICSS paradigm [3, 33].

Parameters of the frequency-following function for oICSS

The form of the frequency-following function (f_F) for channelrhodopsin-2-mediated excitation of midbrain dopamine neurons has yet to be determined. Here, we used a function of the same form as the one we had determined previously for eICSS of the MFB [6], but we substituted new parameter values. Dopamine neurons cannot fire nearly as fast as the directly stimulated neurons subserving the rewarding effect of electrical MFB stimulation [6, 34, 35], and the kinetics of channelrhodopsin-2 are slow in comparison to those of the voltage-gated channels responsible for electrically induced neural firing [36]. Thus, frequency-following parameters determined for eICSS of the MFB cannot be used to account for the frequency response of the dopamine neurons subserving oICSS of the ventral midbrain.

Although two studies found that frequency-following fidelity in optically stimulated midbrain dopamine neurons fell to only 40-50% at optical pulse frequencies of 40-50 pulses s^{-1} [34, 35], results of two other electrophysiological studies show very good firing fidelity at 50 pulses s^{-1} [37, 38]. Moreover, results of a recent electrochemical study [39] show that optically induced dopamine release in the nucleus accumbens continued to rise as the pulse frequency was increased from 40-50 pulses s^{-1}. Poor frequency-following fidelity was found by Lohani and colleagues in midbrain dopamine neurons optically stimulated at 100 pulses s^{-1} [40], suggesting that the upper limit on the induced firing rate lies at a significantly lower pulse frequency.

Figs 4 and S5 show two cases (data from rats BeChR29 and 27) in which the behavioral effectiveness of the stimulation continues to rise at pulse frequencies up to, or beyond, 60 pulses s^{-1}. The curves for the remaining rats approach asymptote earlier, but this does not necessarily reflect failure the higher pulse frequencies to increase firing: saturation of reward-intensity growth [20] could be responsible instead.

In view of the power-frequency trade-off data reported here and the results reported in the studies cited above, we set the middle of the roll-off region of the frequency-following function (F_{ro}) to 50 pulses s^{-1} and the parameter governing the abruptness of the roll-off (F_{bend}) to 20. The resulting frequency-following function is shown in Fig S13. It continues to climb up to optical pulse frequencies of ∼100 pulses s^{-1} to attain a maximum induced firing rate of 51.6 spikes s^{-1}.

Displacement of the shell: distinguishing two sources

The purpose of the experiment is to draw inferences about brain-reward circuitry from the effect of dopamine-transporter blockade on the location of the shell of the reward mountain within the space defined by the two independent variables, the **objective** opportunity cost of the reward, P_{obj}, and the ***pulse*** frequency, F_{pulse}. However, the core variables about which the inferences are to be drawn operate in spaces defined by the ***subjective*** opportunity cost, P_{sub}, and the induced frequency of ***firing***, F_{firing} (Tab S2). As we explain below, the core → shell function, f_F, which maps the pulse frequency into the evoked frequency of firing, contributes to the estimates of both location parameters of the shell. Its influence must be removed in order to isolate the effects of the drug on $F_{firing_{hm}}$ and P_{sub_e}, the location parameters of the core. In the depiction and interpretation of the fitted surfaces, we will distinguish between

- ***primary*** displacement of the shell of the reward mountain due to drug actions on the core components and
- ***secondary*** (additional) displacement of the shell due to the differential response of the frequency-following function (f_F) in the drug and vehicle conditions

In the following section, we explain how to remove the secondary displacements from the location-parameter estimates, thus isolating the primary displacements that are the focus of the study.

Correction of the location-parameter estimates for changes in frequency-following fidelity

Fitting the mountain surface to time-allocation data returns the two location parameters of the reward-mountain shell: P_{obj_e}, which positions the shell along the price axis, and $F_{pulse_{hm}}$, which positions the shell along the pulse-frequency axis. Whereas the value of $F_{pulse_{hm}}$ is independent of the value of P_{obj_e}, the reverse does not hold when frequency-following fidelity is imperfect. Changes in frequency-following fidelity alter the maximum reward intensity ($\widehat{R}_{bsr_{max}}$) , which contributes to the value of both P_{obj_e} and its subjective equivalent, P_{sub_e}. The portion of the shifts in P_{obj_e} due to changes in frequency-following fidelity must be removed in order decouple estimates of that parameter from shifts along the pulse-frequency axis. Once P_{sub_e} has been suitably corrected, manipulations that act at, or beyond, the output of the reward-growth function (f_R, Fig S16) shift the mountain core uniquely along the price axis, whereas manipulations that alter the input to the reward-growth function shift the mountain core uniquely along the frequency axis [2, 3, 41].

Imperfect frequency-following fidelity can also alter the extent to which the surface of the mountain shell shifts along the pulse-frequency axis. This problem will arise if frequency-following fidelity differs in the drug and vehicle conditions. Correction is required in order to estimate the shift of fundamental interest, which is the displacement of the reward-growth function (a core component) along its firing-frequency axis.

The frequency-following function. The correction of the location-parameter estimates arises from the form of the frequency-following function (f_F). The form and parameters of this function for eICSS of the MFB were described by Solomon et al. [14]. In the absence of analogous data for oICSS, we have assumed the same functional form but have tuned the parameters to accommodate the power-frequency trade-off data reported here and results of prior studies [37–40].

In double-logarithmic coordinates, the frequency-following function (Fig S13) has the form of an inverted hockey stick, with a straight handle that transitions into a flat blade [6]. Pulse frequency is represented along the abscissa of this function, and firing frequency is represented along the ordinate. The induced firing frequency follows the pulse frequency perfectly over the portion of the handle that is truly straight, grows more slowly over a transition zone, and levels off over the blade portion. Thus, within the transition zone, a given drug-induced decrement in firing frequency (a core variable) will correspond to a larger decrement in pulse frequency (a shell variable). Drug-induced displacement of the reward-growth function (f_R) towards lower values of $F_{firing_{hm}}$ improves frequency-following fidelity (by moving the pulse frequency toward, or onto the straight "handle"). This causes the displacement of the shell to exceed, and thereby overestimate, the underlying displacement of the reward-growth function.

To correct our estimate of how far the reward-growth function has shifted along the pulse-frequency axis, we need to decouple its value from the maximum normalized reward intensity, $\widehat{R}_{bsr_{max}}$. This is achieved by using the assumed frequency-following function (Eq S1) to estimate $F_{firing_{hm}}$ from $F_{pulse_{hm}}$.

We define the corrected estimate of the location parameter as follows:

$$F^*_{pulse_{hm}} = f_F(F_{pulse_{hm}}) \tag{S39}$$

where

$F^*_{pulse_{hm}}$ = the estimated value of $F_{firing_{hm}}$, which is the value $F_{pulse_{hm}}$ would have attained had frequency-following fidelity been perfect

Eq S39 states that $F^*_{pulse_{hm}}$ and $f_F(F_{pulse_{hm}})$ are one and the same. We will plot the value in question in the coordinate space of the shell. There, the pulse frequency, rather than the firing frequency, serves as the ordinate. Thus, in that context, we use $F^*_{pulse_{hm}}$ in lieu of $f_F(F_{pulse_{hm}})$ as our notation.

$f_F(F_{pulse_{hm}})$ (and thus $F^*_{pulse_{hm}}$) is a value along the ordinate of the frequency-following function (Fig S13), whereas $F_{pulse_{hm}}$ is a value along the abscissa. Given the form of the frequency-following function, $F^*_{pulse_{hm}} < F_{pulse_{hm}}$ once pulse frequency exceeds the capacity of the neurons to fire reliably to each and every pulse.

Several steps are required to correct the the parameter that locates the reward mountain along the price axis so that it too is decoupled from frequency-following fidelity. The step first is analogous to the estimation of $F^*_{pulse_{hm}}$ from $F_{pulse_{hm}}$: We use the subjective-price equation (Eq S6) to transform P_{obj_e} into its subjective equivalent, P_{sub_e}.

The next step is to correct P_{sub_e} for the effect of imperfect frequency-following fidelity. Eq S30 can be rearranged as follows:

$$P_{sub_e} = \left(\frac{p_{sub}}{\left[K_{ec} \times \widehat{\phi}\right] \times \left[K_{aa} \times \widehat{U}_e\right]} \times K_{rg} \right) \times \widehat{R}_{bsr_{max}} \tag{S40}$$

Eq S40 reminds us that P_{sub_e} is proportional to the maximum normalized reward intensity that can be attained, $\widehat{R}_{bsr_{max}}$. Eq S12 defines $\widehat{R}_{bsr_{max}}$ in terms of the maximal attainable firing frequency, $F_{firing_{max}}$, the firing frequency corresponding to $F_{pulse_{hm}}$, and the reward-growth exponent, g. To estimate $F_{firing_{max}}$, we solve Eq S1 for a pulse frequency more than high enough to drive firing frequency to its maximum ($F_{pulse_{max}} = 1000$ pulses s^{-1}). We then use the resulting estimate of $\widehat{R}_{bsr_{max}}$ to produce a revised estimate of P_{sub_e}:

$$P^*_{sub_e} = \frac{P_{sub_e}}{\widehat{R}_{bsr_{max}}} \tag{S41}$$

where

$P^*_{sub_e}$ = estimated value that P_{sub_e} would have attained had frequency-following fidelity been perfect

Last, we transform $P^*_{sub_e}$ into its objective-price counterpart, $P^*_{obj_e}$ by passing $P^*_{sub_e}$ through the back-solution of the subjective-price equation [6]:

$$P^*_{obje} = P_{sub_{min}} + P_{sub_{bend}} \times \ln \left[-1 + e^{\left(\frac{P^*_{sub_e} - P_{sub_{min}}}{P_{sub_{bend}}} \right)} \right] \tag{S42}$$

for $P^*_{sub_e} \geq P_{sub_0}$

(S43)

The transformation of $P^*_{sub_e}$ into $P^*_{obj_e}$ is performed so that the corrected location-parameter estimate can be plotted in the space defined by the independent variables, $\{P_{obj}, F_{pulse}\}$. In this space, drug-induced shifts in the position of the reward mountain, corrected for changes in frequency-following fidelity, are depicted as $\left[\log_{10}\left(F^*_{pulse_{hm_{drug}}}\right) - \log_{10}\left(F^*_{pulse_{hm_{vehicle}}}\right)\right]$ and $\left[\log_{10}\left(P^*_{obj_{e_{drug}}}\right) - \log_{10}\left(P^*_{obj_{e_{vehicle}}}\right)\right]$.

Model fitting and selection

Model selection

The 12 candidate models fit to the data are described in Tab S3. The models differ in the total number of parameters as well as in the number of parameters free to vary across the vehicle and drug conditions. Models 2, 5, 8, and 11 are based on the six-parameter version of the reward-mountain model (Eq S35), whereas the remaining models are based on the seven-parameter version (Eq S38). The fits of all of the candidate models to the reward-mountain data from all seven rats converged successfully.

Table S3. The 12 candidate models fit to each dataset. Values of the "_free" parameters were free to differ between the vehicle and drug conditions, whereas a single value was fitted to the data from both conditions in the case of "_com" (common) parameters. Additional columns list the total number of "Common" and "Free" parameters along with their "Totals." Models 2, 5, 8, and 11 are based on the six-parameter version of the reward-mountain model (Eq S35), whereas the remaining models are based on the seven-parameter version (Eq S38).

Num	a_free	g_free	CR_free	CR_com	Common	Free	Total
1	0	0	1	0	4	6	10
2	0	0	0	0	4	4	8
3	0	0	0	1	5	4	9
4	1	0	1	0	3	8	11
5	1	0	0	0	3	6	9
6	1	0	0	1	4	6	10
7	0	1	1	0	3	8	11
8	0	1	0	0	3	6	9
9	0	1	0	1	4	6	10
10	1	1	1	0	2	10	12
11	1	1	0	0	2	8	10
12	1	1	0	1	3	8	11

Tab S4 ranks the fits of the 12 candidate models for one rat (Bechr29) by their evidence ratios (the relative likelihood that a candidate model is true in comparison to the best-fitting model). Note that the residual sum of squares for the worst-fitting model (model 10) is slightly lower than in the case of the best-fitting model (model 2). This is not surprising given that the worst-fitting model comprises 12 parameters whereas the best-fitting model comprises only eight. The Akaike Information Criterion (AIC) [42] implements a trade-off between goodness of fit and simplicity. Thus, the AIC penalizes models with large number of parameters in comparison to simpler ones. On the basis of the AIC, model 10 is over 5×10^5 times less likely than model 2 and is ranked accordingly. Summary statistics for the best-fitting model for each rat are listed in Tab S5.

Table S4. Model-evaluation statistics for the fit of the 12 candidate models to the data from rat Bechr29. The models are described in Tab S3. "AIC" stands for the Akaike Information Criterion [42]. "Likelihood" refers to the ratio: $\left[\frac{\text{Likelihood(candidate model)}}{\text{Likelihood(best-fitting model)}}\right]$, which expresses the relative likelihood that a candidate model is true in comparison to the best-fitting model. The Evidence Ratio ("Ev_ratio") is the inverse of the likelihood ratio. The total number of parameters is listed under "prms," the residual sum of squares under "RSS," the total sum of squares under "TSS," and the adjusted R^2 under "Adj R^2."

Model	AIC	Likelihood	Ev_ratio	prms	RSS	TSS	Adj R^2
2	-1,922.58	1.000000	1.00	8	13.904	12,647.57	0.998886
8	-1,916.12	0.039636	25.23	9	13.897	12,647.57	0.998885
5	-1,915.86	0.034860	28.69	9	13.904	12,647.57	0.998884
3	-1,915.86	0.034846	28.70	9	13.904	12,647.57	0.998884
11	-1,909.46	0.001421	703.74	10	13.896	12,647.57	0.998883
9	-1,909.40	0.001377	726.48	10	13.897	12,647.57	0.998883
6	-1,909.14	0.001211	825.83	10	13.904	12,647.57	0.998882
1	-1,909.14	0.001210	826.19	10	13.904	12,647.57	0.998882
12	-1,902.74	0.000049	20,330.45	11	13.896	12,647.57	0.998881
7	-1,902.69	0.000048	20,826.76	11	13.897	12,647.57	0.998880
4	-1,902.42	0.000042	23,852.26	11	13.904	12,647.57	0.998880
10	-1,896.02	0.000002	585,023.15	12	13.895	12,647.57	0.998878

Table S5. Summary statistics for the model that provided the best fit (highest evidence ratio) to the data for each rat. The models in the "Model Num" column are defined in Tab S3. The residual sum of squares is listed in column "RSS," the total sum of squares in column "TSS," and the adjusted R^2 in column "Adj R^2."

Rat	Model Num	RSS	TSS	Adj R^2
Bechr14	2	23.23	14,413.79	0.9984
Bechr19	3	28.68	4,554.59	0.9936
Bechr21	11	19.52	14,177.29	0.9986
Bechr26	3	8.52	9,017.52	0.9990
Bechr27	12	4.55	11,171.77	0.9996
Bechr28	9	20.45	8,986.80	0.9977
Bechr29	2	13.90	12,647.57	0.9989

Different variants of the reward-mountain model provided the best fit to the data from different rats. Tab S6 shows the best-fitting model for all rats, as determined by the AIC-based evidence ratio. In four cases (Bechr14,19,26,29), the best-fitting model was one in which common values of the a and g parameters were fit to the data from both the vehicle and drug conditions; in the remaining three cases, the best-fitting model was one in which the values of a, g, or both were free to vary across the vehicle and drug conditions.

In four cases (Bechr19, 26,27,28), the C_r parameter was included in the best-fitting model, whereas in the three remaining cases, it was not. As Fig S15 illustrates, the C_r parameter will be advantageous when time allocation in low-payoff trials is higher along pulse-frequency sweeps than along price or radial sweeps. In no case was the value of this parameter free to vary across the vehicle and drug conditions in the best-fitting model. Thus, there is no evidence that the advantage conferred by addition of the C_r parameter was due to dopamine-transporter blockade.

Table S6. Best-fitting models for all rats. The models are described in Tab S3. Values of the "_free" parameters were free to differ between the vehicle and drug conditions, whereas a single value was fitted to the data from both conditions in the case of "_com" (common) parameters. Additional columns list the total number of common and free parameters and their totals

Rat	Model_num	a_free	g_free	CR_free	CR_com	Common	Free	Total
Bechr14	2	0	0	0	0	4	4	8
Bechr19	3	0	0	0	1	5	4	9
Bechr21	11	1	1	0	0	2	8	10
Bechr26	3	0	0	0	1	5	4	9
Bechr27	12	1	1	0	1	3	8	11
Bechr28	9	0	1	0	1	4	6	10
Bechr29	2	0	0	0	0	4	4	8

Fitted reward-mountain surfaces

Fig S17. Reward-mountain surfaces fit to the vehicle and drug data from rat Bechr14. The surfaces of the reward-mountain shell are shown in gray. The thick black line represents the contour mid-way between the minimal and maximal estimates of time allocation (the estimated altitudes of the valley floor and summit). Mean time-allocation values for the pulse frequency, price, and radial sweeps are denoted by red pyramids, blue squares, and green polyhedrons, respectively.

Fig S18. Reward-mountain surfaces fit to the vehicle and drug data from rat Bechr19. See caption for Fig S17.

Fig S19. Reward-mountain surfaces fit to the vehicle and drug data from rat Bechr21. See caption for Fig S17.

Fig S20. Reward-mountain surfaces fit to the vehicle and drug data from rat Bechr26. See caption for Fig S17.

Fig S21. Reward-mountain surfaces fit to the vehicle and drug data from rat Bechr27. See caption for Fig S17.

Fig S22. Reward-mountain surfaces fit to the vehicle and drug data from rat Bechr28. See caption for Fig S17.

The corresponding graph for rat Bechr29 is shown in Fig 6 in the main text along with the caption.

Contour and bar graphs

Fig S23. Contour graphs of the surfaces fit to the vehicle and drug data and bar graphs of the shifts in the location parameters for rat Bechr14. The values of the independent variables along frequency sweeps are designated by red triangles, along price sweeps by blue squares, and along radial sweeps by green circles. The values of the location parameters, $F_{pulse_{hm}}$ and P_{obj_e}, and are indicated by red horizontal lines with right-facing triangular end points and blue vertical lines with diamond end points, respectively. The shaded regions surrounding the lines denote 95% confidence intervals. The vehicle data are shown twice, once in the upper-left quadrant and once in the lower right. The dotted lines connecting the panels designate the shifts in the common-logarithmic values of the location parameters of the mountain, which are designated as $\{\Delta P_{obj_e}, \Delta F_{hm}\}$ and plotted in the bar graph in the upper-right panel. The dot-dash cyan lines superimposed on the bars show location-parameter estimates corrected for changes in frequency-following fidelity due to the displacement of the mountain along the pulse-frequency axis. (See section ***Correction of the location-parameter estimates for changes in frequency-following fidelity***.) The 95% confidence intervals are shown in the bar graphs as vertical lines.

Fig S24. Contour and bar graphs for rat Bechr19. See caption for Fig S23.

Fig S25. Contour and bar graphs for rat Bechr21. See caption for Fig S23.

Fig S26. Contour and bar graphs for rat Bechr26. See caption for Fig S23.

Fig S27. Contour and bar graphs for rat Bechr27. See caption for Fig S23.

Fig S28. Contour and bar graphs for rat Bechr28. See caption for Fig S23.

The corresponding graphs for rat Bechr29 are shown in Fig 7 in the main text.

Location-parameter estimates

$\mathbf{F_{hm}}$: Tab S7 shows the estimates of $F_{pulse_{hm}}$ (uncorrected) and $F^*_{pulse_{hm}}$ (corrected) for the drug and vehicle conditions. In six of seven cases, a lower pulse frequency sufficed to produce a reward of half-maximal intensity under the influence of dopamine-transporter blockade than in the vehicle condition.

The $F_{pulse_{hm}}$ estimates vary over more than a doubling range in both the drug and vehicle conditions. Particularly in the vehicle condition, the higher values fall within a range over which the assumed frequency-following function (**??**) rolls off, thus preventing the normalized reward-growth function from approaching a value of one at the highest pulse frequencies tested. This is why the uncorrected ($F_{pulse_{hm}}$) and corrected ($F^*_{pulse_{hm}}$) values in Tab S7) differ. For example, the ∼31 pulses s^{-1} that produced a reward of half-maximal intensity in rat Bechr29 in the vehicle condition are estimated to have generated only ∼26 firings s^{-1}.

Tab 2 in the main text shows the estimated drug-induced ***shifts*** in the location of the reward-mountain core along the frequency axis.

Eqs S13,S14 express the effect of the drug on the location parameter of the reward-growth function as a divisor: the more the drug boosts dopamine release, the lower the value of the location parameter for the drug condition and thus the farther the reward-growth function is shifted to the left. The rightmost column in Tab 2 lists the values of this divisor implied by the drug-induced shifts in the position of the reward mountain along the pulse-frequency axis. By analogy to Eq S14,

Table S7. The position of the reward mountain along the pulse-frequency axis. The $F_{pulse_{hm}}$ parameter sets the location of the shell of the reward-mountain along the pulse-frequency axis. If frequency-following fidelity were perfect, a pulse frequency of $F_{pulse_{hm}}$ would have induced an identical firing frequency in the optically activated dopamine neurons. Otherwise, the values corrected for imperfect frequency following, which are shown in the "$F^*_{pulse_{hm}}$" columns, will be lower than the uncorrected values, which are shown in the "$F_{pulse_{hm}}$" columns. The corrected values in the "$F^*_{pulse_{hm}}$" columns are the estimated firing frequencies induced by the pulse frequencies in the "$F_{pulse_{hm}}$" columns, derived from the frequency-following function described in section **??**. The values listed in the "Veh" columns are from the fits to the data acquired in the vehicle condition, whereas the values in the "Drg" columns are from the fits to the data acquired under the influence of GBR-12909.

Rat	$F_{pulse_{hm}}$ Drg	$F_{pulse_{hm}}$ Veh	$F^*_{pulse_{hm}}$ Drg	$F^*_{pulse_{hm}}$ Veh
Bechr14	19.734	27.094	17.341	23.164
Bechr19	11.502	16.265	10.372	14.457
Bechr21	10.072	35.836	9.103	29.453
Bechr26	17.718	25.359	15.668	21.822
Bechr27	23.395	18.892	20.286	16.648
Bechr28	14.728	32.350	13.142	27.007
Bechr29	20.776	31.347	18.184	26.293

$$F^*_{pulse_{hm_{drug}}} = \frac{F^*_{pulse_{hm_{vehicle}}}}{K_{da_{drug}}} \quad \text{(S44)}$$

where

$F^*_{pulse_{hm_{drug}}}$ = location parameter of the reward-growth function for the drug condition

$F^*_{pulse_{hm_{vehicle}}}$ = location parameter of the reward-growth function for the vehicle condition

$K_{da_{drug}}$ = proportional reduction in the value of the location parameter of the reward-growth function due to dopamine-transporter blockade

It follows that

$$K_{da_{drug}} = 10^{-\text{diff}}$$

where

$$\text{diff} = \log\left(F^*_{pulse_{hm_{drug}}}\right) - \log\left(F^*_{pulse_{hm_{vehicle}}}\right)$$

$\mathbf{P_e}$: In previous work employing the reward-mountain model [3, 33, 43, 44], changes in the location of the fitted surface along the price axis have been attributed to variables acting at, or beyond, the ***output*** of the reward-growth function, whereas changes in the location of the fitted surface along the pulse-frequency axis have been attributed to variables acting at, or prior to, the ***input*** to the reward-growth function. The reward-mountain model treats these two sets of changes as independent, a postulate that is largely supported by empirial findings [1, 2, 13]. This interpretation is valid as long as the induced firing frequency can be driven high enough to maximize reward intensity. Tab S8 shows that this assumption does not hold in several of the datasets from the present study: The maximum normalized reward intensity, $\widehat{R}_{bsr_{max}}$, is substantially less than one in these cases.

The deviation of $\widehat{R}_{bsr_{max}}$ from one is generally greater in the vehicle data than in the drug data. In such cases, a

Table S8. Estimates of the maximum normalized reward intensities in the vehicle and drug conditions. The values in "$\widehat{R}_{bsr_{max}}$ Drg" and "$\widehat{R}_{bsr_{max}}$ Veh" columns are the maximum normalized reward intensities in the vehicle and drug conditions, respectively, based on the frequency-following function described in section ??. The ratios of these values (Drg / Veh) are listed in the "Ratio" column, and the common logarithms of the ratios in the "log(Ratio)" column.

Rat	$\widehat{R}_{bsr_{max}}$ Drg	$\widehat{R}_{bsr_{max}}$ Veh	Ratio	log(Ratio)
Bechr14	0.986	0.958	1.029	0.013
Bechr19	1.000	0.999	1.001	0.000
Bechr21	1.000	0.849	1.179	0.071
Bechr26	0.998	0.989	1.009	0.004
Bechr27	0.964	0.997	0.967	-0.015
Bechr28	1.000	0.952	1.051	0.022
Bechr29	0.964	0.894	1.079	0.033

portion of the change in the value of the P_{obj_e} parameter is due to fact that the drug displaced the rising portion of the reward-mountain surface into a range of pulse frequencies over which the fidelity of frequency following is better than in the vehicle condition. That contribution to the change in the value of the P_{obj_e} parameter reflects mitigation at the ***input*** to the reward-growth function (Eq S11), thus undermining the independence of the changes in the parameters that locate the reward mountain along the price and frequency axes. That is why we computed corrected estimates ($P^*_{obj_e}$), as described in section ***Correction of the location-parameter estimates for changes in frequency-following fidelity*** thus compensating for the differences in the value of $\widehat{R}_{bsr_{max}}$ across the vehicle and drug conditions. The estimates of P_{obj_e} and $P^*_{obj_e}$ are shown in Tab S9.

Table S9. The position of the reward mountain along the price axis. The P_{obj_e} parameter determines the position of the reward mountain along the price axis. When the maximum normalized reward intensity differs between the drug and vehicle conditions due to differences in frequency-following fidelity, the value of the P_{obj_e} parameter is affected. The values listed in the "$P^*_{obj_e}$" columns have been corrected to remove this effect. They show the estimated value that the P_{obj_e} parameter would have attained had frequency-following fidelity been perfect.

Rat	P_{obj_e} Drg	P_{obj_e} Veh	$P^*_{obj_e}$ Drg	$P^*_{obj_e}$ Veh
Bechr14	9.572	6.127	9.414	6.252
Bechr19	11.755	5.369	11.658	5.363
Bechr21	25.102	13.662	25.096	15.933
Bechr26	14.051	11.625	14.347	11.843
Bechr27	22.434	19.475	23.066	19.592
Bechr28	75.392	51.225	76.366	56.401
Bechr29	10.041	6.295	10.480	7.375

Tab 3 in the main text lists the drug-induced shifts in the common logarithms of $P^*_{obj_e}$.

The drug-induced shifts in the location-parameter values are uncorrelated

Parameter values for best-fitting model for all rats

The values of the location parameters in these tables are uncorrected. Corrected values are listed in Tabs 2 and 3 in the main text.

Fig S29. Scatter plot of drug-induced shifts in the location parameters. The corrected estimates of displacement along the price and pulse-frequency axes are shown on the abscissa and ordinate, respectively. F^{*}_{hm} is shorthand for $F^{*}_{pulse_{hm}}$

Table S10. Parameter values from the best-fitting model for the data from Rat Bechr14. Columns CB_{low} and CB_{high} list the upper and lower bounds of the 95.2% confidence intervals.

	Vehicle			Drug		
Parameter	Estimate	CB_{low}	CB_{high}	Estimate	CB_{low}	CB_{high}
a	1.064	0.922	1.233	1.064	0.922	1.233
g	3.915	3.450	4.423	3.915	3.450	4.423
$Log_{10}(F_{hm})$	1.433	1.390	1.478	1.295	1.262	1.331
$Log_{10}(P_e)$	0.787	0.694	0.869	0.981	0.889	1.052
T_{max}	0.821	0.771	0.881	0.821	0.771	0.881
T_{min}	0.144	0.127	0.159	0.144	0.127	0.159

Table S11. Parameter values from the best-fitting model for the data from Rat Bechr19. Columns CB_{low} and CB_{high} list the upper and lower bounds of the 95.2% confidence intervals.

	Vehicle			Drug		
Parameter	Estimate	CB_{low}	CB_{high}	Estimate	CB_{low}	CB_{high}
a	1.840	1.729	1.972	1.840	1.729	1.972
C_r	0.214	0.191	0.239	0.214	0.191	0.239
g	5.862	3.837	13.690	5.862	3.837	13.690
$Log_{10}(F_{hm})$	1.211	1.172	1.247	1.061	0.996	1.100
$Log_{10}(P_e)$	0.730	0.699	0.762	1.070	1.051	1.089
T_{max}	0.796	0.777	0.816	0.796	0.777	0.816
T_{min}	0.005	0.000	0.009	0.005	0.000	0.009

Table S12. Parameter values from the best-fitting model for the data from Rat Bechr21. Columns CB_{low} and CB_{high} list the upper and lower bounds of the 95.2% confidence intervals.

	Vehicle			Drug		
Parameter	Estimate	CB_{low}	CB_{high}	Estimate	CB_{low}	CB_{high}
a	1.442	1.368	1.523	3.436	3.222	3.674
g	3.109	2.719	3.593	10.992	10.503	11.432
$Log_{10}(F_{hm})$	1.554	1.480	1.649	1.003	1.000	1.007
$Log_{10}(P_e)$	1.136	1.115	1.154	1.400	1.388	1.410
T_{max}	0.836	0.829	0.843	0.836	0.829	0.843
T_{min}	0.097	0.091	0.103	0.097	0.091	0.103

Table S13. Parameter values from the best-fitting model for the data from Rat Bechr26. Columns CB_{low} and CB_{high} list the upper and lower bounds of the 95.2% confidence intervals.

	Vehicle			Drug		
Parameter	Estimate	CB_{low}	CB_{high}	Estimate	CB_{low}	CB_{high}
a	1.998	1.887	2.106	1.998	1.887	2.106
C_r	0.071	0.062	0.080	0.071	0.062	0.080
g	5.233	4.788	5.804	5.233	4.788	5.804
$Log_{10}(F_{hm})$	1.404	1.384	1.427	1.248	1.232	1.268
$Log_{10}(P_e)$	1.048	1.080	1.148	1.134	1.164	
T_{max}	0.882	0.870	0.895	0.882	0.870	0.895
T_{min}	0.057	0.051	0.063	0.057	0.051	0.063

Table S14. Parameter values from the best-fitting model for the data from Rat Bechr27. Columns CB_{low} and CB_{high} list the upper and lower bounds of the 95.2% confidence intervals.

	Vehicle			Drug		
Parameter	Estimate	CB_{low}	CB_{high}	Estimate	CB_{low}	CB_{high}
a	1.454	1.381	1.525	2.015	1.924	2.135
C_r	0.037	0.035	0.039	0.037	0.035	0.039
g	5.050	4.561	5.684	3.524	3.360	3.699
$Log_{10}(F_{hm})$	1.276	1.248	1.307	1.369	1.342	1.394
$Log_{10}(P_e)$	1.289	1.272	1.303	1.351	1.337	1.364
T_{max}	0.888	0.881	0.895	0.888	0.881	0.895
T_{min}	0.009	0.000	0.028	0.009	0.000	0.028

Table S15. Parameter values from the best-fitting model for the data from Rat Bechr28. Columns CB_{low} and CB_{high} list the upper and lower bounds of the 95.2% confidence intervals.

	Vehicle			Drug		
Parameter	Estimate	CB_{low}	CB_{high}	Estimate	CB_{low}	CB_{high}
a	2.432	2.304	2.595	2.432	2.304	2.595
C_r	0.022	0.021	0.024	0.022	0.021	0.024
g	4.609	4.281	5.077	17.305	16.182	18.765
$Log_{10}(F_{hm})$	1.510	1.489	1.532	1.168	1.163	1.173
$Log_{10}(P_e)$	1.709	1.687	1.730	1.877	1.860	1.893
T_{max}	0.915	0.910	0.921	0.915	0.910	0.921
T_{min}	0.143	0.135	0.151	0.143	0.135	0.151

Table S16. Parameter values from the best-fitting model for the data from Rat Bechr29. Columns CB_{low} and CB_{high} list the upper and lower bounds of the 95.2% confidence intervals.

	Vehicle			Drug		
Parameter	Estimate	CB_{low}	CB_{high}	Estimate	CB_{low}	CB_{high}
a	2.312	2.172	2.462	2.312	2.172	2.462
g	3.161	2.980	3.354	3.161	2.980	3.354
$Log_{10}(F_{hm})$	1.496	1.476	1.518	1.318	1.304	1.335
$Log_{10}(P_e)$	0.799	0.783	0.814	1.002	0.990	1.015
T_{max}	0.896	0.883	0.910	0.896	0.883	0.910
T_{min}	0.112	0.107	0.118	0.112	0.107	0.118

The reward-growth function for oICSS

Fig S30. Changing the value of the input-scaling parameter, F_{hm}, shifts the mountain along the pulse-frequency axis. F_{hm} is shorthand for $F_{pulse_{hm}}$

Fig S31. Changing the value of the output-scaling parameter, K_{rg}, shifts the mountain along the price axis. P_e is shorthand for P_{obj_e}

Illustration of the correction for imperfect frequency-following fidelity

When the parameter that locates the reward mountain along the pulse-frequency axis ($F_{pulse_{hm}}$) falls within the range over which the induced firing frequency diverges substantially from the pulse frequency, then the magnitude of a drug-induced shift along the pulse-frequency axis is exaggerated, and a fictive shift is produced along the price axis. Fig S32 illustrates this effect and its removal by means of the correction procedure. The dot-dash cyan contour line shows the objective-price and pulse-frequency values that would have driven time allocation halfway between its minimal and maximal values had frequency-following fidelity been perfect. In contrast, the solid, wide, black contour line shows the equivalent objective-price and pulse-frequency values given the assumed frequency-following function. Note that the dot-dash cyan line deviates more from the solid, wide, black line in the simulated vehicle data in the upper left and lower right quadrants than in the simulated drug data in the lower-left quadrant. This is so because the uncorrected location-parameter value (vehicle: ~ 39 pulses s^{-1}; drug: ~ 18 pulses s^{-1}) is much closer to the estimated maximum attainable firing frequency (51.67 firings s^{-1}) in the vehicle condition than in the drug condition. As a result, the simulated firing rate falls further below the pulse frequency in the vehicle condition than in the drug condition. The dot-dash lines superimposed on the bar graphs show the result of correcting the location-parameter shifts for this effect.

Fig S32. Correction for imperfect frequency-following fidelity. Simulated data, with $F_{pulse_{hm}}$ placed well within the region over which frequency-following fidelity falls off substantially. The simualated vehicle data are shown twice, once in the upper-left quadrant and once in the lower right. The dotted lines connecting the panels designate the shifts in the common-logarithmic values of the location parameters of the mountain, which are designated as $\{\Delta P_{obj_e}, \Delta F_{hm}\}$ and plotted in the bar graph in the upper-right panel. The dot-dash cyan lines superimposed on the bars show location-parameter estimates corrected for changes in frequency-following fidelity due to the displacement of the mountain along the pulse-frequency axis. F_{hm} is shorthand for $F_{pulse_{hm}}$

Comparison of logistic and power growth of reward intensity

Fig S33. The input-scaling parameter of the power reward-growth function locates the reward mountain along the price axis. Contour- and bar-graph representation of the simulated reward mountains produced by the magenta and green power-reward-growth functions (Eq 2) in the lower-left panel of Fig 10. In contrast to the effect of varying the value of the input-scaling parameter on the location of reward mountains based on logistic reward growth (Figs S30, S31), changing the value of the input-scaling parameter of the power-reward-growth function shifts the mountain along the price axis and not along the pulse-frequency axis.

Fig S34. The output-scaling parameter of the power reward-growth function also locates the reward mountain along the price axis. Contour- and bar-graph representation of the simulated reward mountains produced by the magenta and green power-reward-growth functions (Eq 2) in the lower-right panel of Fig 10. Changing the value of the output-scaling parameter of the power-reward-growth function shifts the mountain along the price axis just like the effect of changing the value of the input-scaling parameter shown in Fig S33.

Toward a new model of brain-reward circuitry

Fig S35. Contour graphs of reward mountains simulated by the convergence model. Dopamine-transporter blockade shifts the simulated reward mountain (almost) exclusively along the pulse-frequency axis, as in the behavioral data. The simulated MFB drive on the dopamine neurons is equivalent to an optical pulse frequency of 40 pulses s^{-1}.

Fig S36. Contour graphs of reward mountains simulated by the convergence model given very strong MFB input. The simulated MFB drive on the dopamine neurons is now equivalent to an optical pulse frequency of 80 pulses s^{-1}.

Training in preparation for measurement of the reward mountain

Fig S37. Graphical summary of the experimental procedure. A: TH::Cre +/- rats received bilateral VTA injections of an AAV5 virus bearing a Cre-dependent, ChR2-YFP transcript. Optical fibers were bilaterally aimed at the VTA. B: Rats were trained to hold down a lever for a specified cumulative amount of time to deliver trains of optical stimulation to the VTA. The red curve represents the proportion of trial time the rat spent working for the optical reward as the optical pulse frequency (the reward-strength variable) was systematically manipulated. The blue curve shows the proportion of trial time the rat spent working for a maximal optical reward as the cumulative amount of time required to harvest the reward ("price") was manipulated systematically. The green curves show proportion of trial time the rat spent working for the optical reward as the strength and price of the reward were simultaneously manipulated. C: The reward-mountain model was fit independently to the data from each rat following injections of GBR-12909 or vehicle. Within subject comparisons were performed.

Contour lines: the trade-off between pulse frequency and price to hold time allocation constant

Contour graphs provide a compact summary of the reward-mountain surface in a format that facilitates visualization of the direction(s) in which the mountain has been shifted by a manipulation such as administration of a drug. The changes in the values of the location parameters become visually apparent in this format.

Here, we derive the equation for the contour lines, thus updating an earlier derivation [14] in which it had been assumed that the higher pulse frequencies tested drive reward intensity to its maximum attainable value ($\widehat{R}_{max} \to 1$). This will indeed be so if the pulse frequencies in question are substantially lower than the frequency-following limit in the directly stimulated neurons. That assumption was usually justified in previous eICSS studies in which the reward mountain was measured [2, 3, 6, 13, 14, 33, 43, 44]. Highly excitably MFB neurons served as the directly activated substrate for the rewarding effect in those studies. In contrast, midbrain dopamine neurons are directly activated substrate in the current study. Not only do these neurons have more limited frequency-following abilities than their MFB counterparts [38, 40], their activation is due to optical excitation of a relatively slow opsin [36] rather than to electrical excitation of voltage-sensitive membrane channels. As we show below, it is likely that the highest pulse frequencies employed in the present study did not always succeed in driving reward intensity to its maximum, particularly in the vehicle condition. To accommodate such cases, we now generalize the previously published expression for the contour lines [14].

We begin by reformatting Eq 33 from the main text as follows:

$$\vec{\widehat{R}}_{cont} = \left(\frac{\widehat{T}_{cont}}{1-\widehat{T}_{cont}}\right)^{\dfrac{1}{a}} \times \left[\widehat{R}_{max} \times \left(\frac{f_P(\vec{P}_{obj_{cont}})}{f_P(P_{obj_e})}\right)\right] \tag{S45}$$

where

$$\begin{aligned}
a &= \text{price-sensitivity exponent} \\
\vec{P}_{obj_{cont}} &= \text{vector of prices for which } \widehat{T} = \widehat{T}_{cont} \text{ when } \widehat{R} \text{ is a corresponding element of } \widehat{R}_{cont} \qquad \text{(S46)} \\
P_{obj_e} &= \text{objective price at which } T = T_{mid} \text{ when } \widehat{R} = \widehat{R}_{max} \\
f_P(P_{obj_{cont}}) &= \text{subjective equivalent of } P_{obj_{cont}} \\
f_P(P_{obj_e}) &= \text{subective price at which } T = T_{mid} \text{ when } \widehat{R} = \widehat{R}_{max} \\
\vec{\widehat{R}}_{cont} &= \text{vector of normalized reward intensities for which } \widehat{T} = \widehat{T}_{cont} \text{ when } P_{obj} \text{ is a corresponding} \\
&\quad\ \text{element of } P_{obj_{cont}};\ 0 \leq \widehat{R} \leq 1 \\
\widehat{R}_{max} &= \text{maximum normalized reward intensity} \\
\widehat{T}_{cont} &= \text{time allocation represented by the contour line} \\
\widehat{T}_{cont} &= \text{normalized time allocation represented by the contour line;} \\
&\quad\ \widehat{T}_{cont} = (T_{cont} - T_{min}) \div (T_{max} - T_{min});\ 0 \leq \widehat{T}_{cont} \leq 1;
\end{aligned}$$

Substituting for $\widehat{R}$ from Eq 12 in the main text, we obtain:

$$\frac{f_F(\vec{F}_{pulse_{cont}})^g}{f_F(\vec{F}_{pulse_{cont}})^g + f_F(F_{pulse_{hm}})^g} = \left(\frac{\widehat{T}_{cont}}{1-\widehat{T}_{cont}}\right)^{\dfrac{1}{a}} \times \left[\widehat{R}_{max} \times \left(\frac{f_P(\vec{P}_{obj_{cont}})}{f_P(P_{obj_e})}\right)\right] \tag{S47}$$

We now multiply both sides by $f_F(\vec{F}_{pulse_{cont}})^g + f_F(F_{pulse_{hm}})^g$, yielding

$$f_F(\vec{F}_{pulse_{cont}})^g =$$
$$\left[f_F(\vec{F}_{pulse_{cont}})^g + f_F(F_{pulse_{hm}})^g\right] \times \left\{\left(\frac{\widehat{T}_{cont}}{1-\widehat{T}_{cont}}\right)^{\frac{1}{a}} \times \left[\widehat{R}_{max} \times \left(\frac{f_P(\vec{P}_{obj_{cont}})}{f_P(P_{obj_e})}\right)\right]\right\} \tag{S48}$$

and we then expand the right side to yield:

$$\begin{aligned}
f_F(\vec{F}_{pulse_{cont}})^g = &\left\{f_F(\vec{F}_{pulse_{cont}})^g \times \left(\frac{\widehat{T}_{cont}}{1-\widehat{T}_{cont}}\right)^{\dfrac{1}{a}} \times \left[\widehat{R}_{max} \times \left(\frac{f_P(\vec{P}_{obj_{cont}})}{f_P(P_{obj_e})}\right)\right]\right\} + \\
&\left\{f_F(\vec{F}_{pulse_{hm}})^g \times \left(\frac{\widehat{T}_{cont}}{1-\widehat{T}_{cont}}\right)^{\dfrac{1}{a}} \times \left[\widehat{R}_{max} \times \left(\frac{f_P(\vec{P}_{obj_{cont}})}{f_P(P_{obj_e})}\right)\right]\right\}
\end{aligned} \tag{S49}$$

The terms that include $f_F(\vec{F}_{pulse_{cont}})^g$ are collected on the left side

$$f_F(\vec{F}_{pulse_{cont}})^g - \left\{ f_F(\vec{F}_{pulse_{cont}})^g \times \left(\frac{\widehat{T}_{cont}}{1-\widehat{T}_{cont}}\right)^{\frac{1}{a}} \times \left[\widehat{R}_{max} \times \left(\frac{f_P(\vec{P}_{obj_{cont}})}{f_P(P_{obj_e})}\right)\right]\right\} = f_F(\vec{F}_{pulse_{hm}})^g \times \left(\frac{\widehat{T}_{cont}}{1-\widehat{T}_{cont}}\right)^{\frac{1}{a}} \times \left[\widehat{R}_{max} \times \left(\frac{f_P(\vec{P}_{obj_{cont}})}{f_P(P_{obj_e})}\right)\right] \quad \text{(S50)}$$

and the left side is factored to yield:

$$f_F(\vec{F}_{pulse_{cont}})^g \times \left(1 - \left\{\left(\frac{\widehat{T}_{cont}}{1-\widehat{T}_{cont}}\right)^{\frac{1}{a}} \times \left[\widehat{R}_{max} \times \left(\frac{f_P(\vec{P}_{obj_{cont}})}{f_P(P_{obj_e})}\right)\right]\right\}\right) = f_F(\vec{F}_{pulse_{hm}})^g \times \left(\frac{\widehat{T}_{cont}}{1-\widehat{T}_{cont}}\right)^{\frac{1}{a}} \times \left[\widehat{R}_{max} \times \left(\frac{f_P(\vec{P}_{obj_{cont}})}{f_P(P_{obj_e})}\right)\right] \quad \text{(S51)}$$

Re-arranging the terms, we obtain:

$$\frac{f_F(\vec{F}_{pulse_{cont}})^g}{f_F(F_{pulse_{hm}})^g} = \frac{\left(\frac{\widehat{T}_{cont}}{1-\widehat{T}_{cont}}\right)^{\frac{1}{a}} \times \left[\widehat{R}_{max} \times \left\{\frac{P_{sub}\left(\vec{P}_{obj_{cont}}\right)}{P_{sub}\left(P_{obj_e}\right)}\right\}\right]}{1 - \left\{\left(\frac{\widehat{T}_{cont}}{1-\widehat{T}_{cont}}\right)^{\frac{1}{a}} \times \left[\widehat{R}_{max} \times \left\{\frac{P_{sub}\left(\vec{P}_{obj_{cont}}\right)}{P_{sub}\left(P_{obj_e}\right)}\right\}\right]\right\}} \quad \text{(S52)}$$

The numerator and denominator of the right side are now multiplied by $f_P(P_{obj_e}) \times \left(\frac{1-\widehat{T}_{cont}}{\widehat{T}_{cont}}\right)^{\frac{1}{a}}$ to yield

$$\frac{f_F(\vec{F}_{pulse_{cont}})^g}{f_F(F_{pulse_{hm}})^g} = \frac{\widehat{R}_{max} \times f_P(\vec{P}_{obj_{cont}})}{\left[\left(\frac{1-\widehat{T}_{cont}}{\widehat{T}_{cont}}\right)^{\frac{1}{a}} \times f_P(P_{obj_e})\right] - \left(\widehat{R}_{max} \times f_P(\vec{P}_{obj_{cont}})\right)} \quad \text{(S53)}$$

The contour graphs will be plotted in double logarithmic coordinates. In that space, Eq S53 becomes

$$\log_{10}\left[f_F(\vec{F}_{pulse_{cont}})\right] - \log_{10}\left(f_F(F_{pulse_{hm}})\right) =$$

$$\left(\frac{1}{g}\right) \times \log_{10}\left\{\frac{\widehat{R}_{max} \times f_P(\vec{P}_{obj_{cont}})}{\left[\left(\frac{1-\widehat{T}_{cont}}{\widehat{T}_{cont}}\right)^{\frac{1}{a}} \times f_P(P_{obj_e})\right] - \left[\widehat{R}_{max} \times f_P(\vec{P}_{obj_{cont}})\right]}\right\} \tag{S54}$$

When time allocation falls halfway between T_{min} and T_{max} ($T = T_{mid}$; $\widehat{T} = 0.5$), Eq S54 reduces to:

$$\log_{10}\left[f_F(\vec{F}_{pulse_{\widehat{T}=0.5}})\right] - \log_{10}\left[f_F(F_{pulse_{hm}})\right] =$$

$$\left(\frac{1}{g}\right) \times \log_{10}\left\{\frac{\widehat{R}_{max} \times f_P(\vec{P}_{obj_{\widehat{T}=0.5}})}{f_P(P_{obj_e}) - \left[\widehat{R}_{max} \times f_P(\vec{P}_{obj_{\widehat{T}=0.5}})\right]}\right\} \tag{S55}$$

Expanding the right side, we obtain

$$\log_{10}\left[f_F(\vec{F}_{pulse_{T=0.5}})\right] - \log_{10}\left[f_F(F_{pulse_{hm}})\right] =$$

$$\left(\frac{1}{g}\right) \times \left\{\log_{10}\left\langle \widehat{R}_{max} \times f_P(\vec{P}_{obj_{T=0.5}})\right\rangle - \log_{10}\left\langle f_P(P_{obj_e}) - \left[\widehat{R}_{max} \times f_P(\vec{P}_{obj_{T=0.5}})\right]\right\rangle\right\} \tag{S56}$$

When $\widehat{R}_{max} = 1$, Eq S56 reduces to

$$\log_{10}\left[f_F(\vec{F}_{pulse_{T=0.5}})\right] - \log_{10}\left[f_F(F_{pulse_{hm}})\right] =$$

$$\left(\frac{1}{g}\right) \times \left\{\log_{10}\left[f_P(\vec{P}_{obj_{T=0.5}})\right] - \log_{10}\left[f_P(P_{obj_e}) - f_P(\vec{P}_{obj_{T=0.5}})\right]\right\} \tag{S57}$$

References

1. Arvanitogiannis A, Shizgal P. The reinforcement mountain: allocation of behavior as a function of the rate and intensity of rewarding brain stimulation. Behavioral Neuroscience. 2008;122(5):1126–1138. doi:10.1037/a0012679.
2. Breton YA, Mullett A, Conover K, Shizgal P. Validation and extension of the reward-mountain model. Frontiers in Behavioral Neuroscience. 2013;7:125. doi:10.3389/fnbeh.2013.00125.
3. Hernandez G, Breton YA, Conover K, Shizgal P. At what stage of neural processing does cocaine act to boost pursuit of rewards? PLoS ONE. 2010;5(11). doi:10.1371/journal.pone.0015081.
4. Gibson J. The Theory of Affordances. In: Shaw R, Bransford J, editors. Perceiving, Acting, and Knowing: Toward an Ecological Psychology. Hillsdale, N.J.: Lawrence Erlbaum Associates; 1977. p. 67–82.
5. McDowell JJ. On the classic and modern theories of matching. Journal of the Experimental Analysis of Behavior. 2005;84(1):111–127. doi:10.1901/jeab.2005.59-04.

6. Solomon RB, Trujillo-Pisanty I, Conover K, Shizgal P. Psychophysical Inference of Frequency-Following Fidelity in the Neural Substrate for Brain Stimulation Reward. Behavioural Brain Research. 2015;292:327–341. doi:10.1016/j.bbr.2015.06.008.

7. Edmonds DE, Gallistel CR. Reward versus performance in self-stimulation: electrode-specific effects of alpha-methyl-p-tyrosine on reward in the rat. Journal of Comparative and Physiological Psychology. 1977;91(5):962–974.

8. Miliaressis E, Rompré PP, Laviolette P, Philippe L, Coulombe D. The curve-shift paradigm in self-stimulation. Physiology & behavior. 1986;37(1):85–91.

9. Wise RA. Action of Drugs of Abuse on Brain Reward Systems. Pharmacology, biochemistry, and behavior. 1980;13 Suppl 1:213–223.

10. Gallistel CR. Spatial and Temporal Summation in the Neural Circuit Subserving Brain Stimulation Reward. In: Wauquier A, Rolls ET, editors. Brain-Stimulation Reward: A Collection of Papers Prepared for the First International Conference on Brain-Stimulation Reward at Janssen Pharmaceutica, Beerse, Belgium on April 21-24, 1975. Oxford: North-Holland; 1976. p. 97–99.

11. Gallistel CR. Self-Stimulation in the Rat: Quantitative Characteristics of the Reward Pathway. Journal of Comparative and Physiological Psychology. 1978;92(6):977–998.

12. Matthews G. Neural substrate for brain stimulation reward in the rat: cathodal and anodal strength-duration properties. Journal of Comparative and Physiological Psychology. 1977;91(4):858–874.

13. Breton YA, Conover K, Shizgal P. The effect of probability discounting on reward seeking: a three-dimensional perspective. Frontiers in Behavioral Neuroscience. 2014;8:284. doi:10.3389/fnbeh.2014.00284.

14. Solomon RB, Conover K, Shizgal P. Valuation of Opportunity Costs by Rats Working for Rewarding Electrical Brain Stimulation. PLOS ONE. 2017;12(8):e0182120. doi:10.1371/journal.pone.0182120.

15. Niyogi RK, Breton YA, Solomon RB, Conover K, Shizgal P, Dayan P. Optimal indolence: a normative microscopic approach to work and leisure. Journal of The Royal Society Interface. 2013;11(91):20130969–20130969. doi:10.1016/S0166-4328(02)00282-6.

16. Petry N, Heyman G. Rat toys, reinforcers, and response strength: An examination of the Re parameter in Herrnstein's equation. Behavioural Processes. 1997;39:39–52.

17. Gallistel C, Shizgal P, Yeomans J. A Portrait of the Substrate for Self-Stimulation. Psychol Rev. 1981;88(3):228–273.

18. Gallistel CR, Leon M. Measuring the Subjective Magnitude of Brain Stimulation Reward by Titration with Rate of Reward. Behavioral Neuroscience. 1991;105(6):913–925.

19. Leon M, Gallistel CR. The Function Relating the Subjective Magnitude of Brain Stimulation Reward to Stimulation Strength Varies with Site of Stimulation. Behavioural Brain Research. 1992;52(2):183–193.

20. Simmons JM, Gallistel CR. Saturation of Subjective Reward Magnitude as a Function of Current and Pulse Frequency. Behavioral Neuroscience. 1994;108(1):151–160.

21. Sonnenschein B, Conover K, Shizgal P. Growth of Brain Stimulation Reward as a Function of Duration and Stimulation Strength. Behavioral Neuroscience. 2003;117(5):978–994. doi:http://psycnet.apa.org/journals/bne/117/5/978.

22. Killeen P. The Matching Law. Journal of the Experimental Analysis of Behavior. 1972;17(3):489–495.

23. Gallistel CR, Gibbon J. Time, rate, and conditioning. Psychological Review. 2000;107(2):289–344.

24. Kiss I, Shizgal P. Compound action potentials recorded in the ventral tegmental area, substantia nigra, and periaqueductal gray following rewarding stimulation of the lateral hypothalamus in the rat. Psychobiology. 1990;18(2):205–214.

25. Rompré PP, Shizgal P. Electrophysiological characteristics of neurons in forebrain regions implicated in self-stimulation of the medial forebrain bundle in the rat. Brain research. 1986;364(2):338–349.

26. Shizgal P, Schindler D, Rompré PP. Forebrain neurons driven by rewarding stimulation of the medial forebrain bundle in the rat: comparison of psychophysical and electrophysiological estimates of refractory periods. Brain research. 1989;499(2):234–248.

27. Heyman GM, Monaghan MM. Reinforcer magnitude (sucrose concentration) and the matching law theory of response strength. J Exp Anal Behav. 1994;61(3):505–516.

28. Heyman GM, Beer B. A new approach for evaluating the behavioral effects of antipsychotic drugs. Trends in Pharmacological Sciences. 1987;8(10):388–393.

29. Heyman G. How drugs affect cells and reinforcement affects behavior: formal analogies. In: Commons ML, Church RM, Stellar JR, Wagner AR, editors. Biological determinants of reinforcement. vol. 7 of Quantitative analysis of behavior. New York, NY: Lawrence Erlbaum Associates; 1988. p. 157–182.

30. Herrnstein R. On the Law of Effect. J Exp Anal Behav. 1970;13(2):243–266.

31. Herrnstein R. Formal Properties of the Matching Law. J Exp Anal Behav. 1974;21(1):159–164.

32. Breton YA, Marcus JC, Shizgal P. Rattus Psychologicus: construction of preferences by self-stimulating rats. Behavioural Brain Research. 2009;202(1):77–91. doi:10.1016/j.bbr.2009.03.019.

33. Hernandez G, Trujillo-Pisanty I, Cossette MP, Conover K, Shizgal P. Role of dopamine tone in the pursuit of brain stimulation reward. The Journal of neuroscience : the official journal of the Society for Neuroscience. 2012;32(32):11032–11041. doi:10.1523/JNEUROSCI.1051-12.2012.

34. Tsai HC, Zhang F, Adamantidis A, Stuber GD, Bonci A, de Lecea L, et al. Phasic firing in dopaminergic neurons is sufficient for behavioral conditioning. Science (New York, NY). 2009;324(5930):1080–1084. doi:10.1126/science.1168878.

35. Witten IB, Steinberg EE, Davidson TJ, Yizhar O, Ramakrishnan C, Stuber GD, et al. Recombinase-driver rat lines: tools, techniques, and optogenetic application to dopamine-mediated reinforcement. Neuron. 2011;72(5):721–733. doi:10.1016/j.neuron.2011.10.028.

36. Klapoetke NC, Murata Y, Kim SS, Pulver SR, Birdsey-Benson A, Cho YK, et al. Independent optical excitation of distinct neural populations. Nature Methods. 2014;11(3):338–346. doi:10.1038/nmeth.2836.

37. Cohen JY, Haesler S, Vong L, Lowell BB, Uchida N. Neuron-type-specific signals for reward and punishment in the ventral tegmental area. Nature. 2012; p. 1–6. doi:10.1038/nature10754.

38. Ilango A, Kesner AJ, Broker CJ, Wang DV, Ikemoto S. Phasic excitation of ventral tegmental dopamine neurons potentiates the initiation of conditioned approach behavior: parametric and reinforcement-schedule analyses. Frontiers in Behavioral Neuroscience. 2014;8:155. doi:10.3389/fnbeh.2014.00155.

39. Covey DP, Cheer JF. Accumbal Dopamine Release Tracks the Expectation of Dopamine Neuron-Mediated Reinforcement. Cell Reports. 2019;27(2):481–490.e3. doi:10.1016/j.celrep.2019.03.055.

40. Lohani S, Martig AK, Deisseroth K, Witten IB, Moghaddam B. Dopamine Modulation of Prefrontal Cortex Activity Is Manifold and Operates at Multiple Temporal and Spatial Scales. Cell Reports. 2019;27(1):99–114.e6. doi:10.1016/j.celrep.2019.03.012.

41. Shizgal P, Conover K, Trujillo-Pisanty I. Video Introducing the Mountain Model; 2012.

42. Akaike H. A new look at the statistical model identification. IEEE Transactions on Automatic Control. 1974;19(6):716–723. doi:10.1109/TAC.1974.1100705.

43. Trujillo-Pisanty I, Conover K, Shizgal P. A new view of the effect of dopamine receptor antagonism on operant performance for rewarding brain stimulation in the rat. Psychopharmacology. 2014;231(7):1351–1364. doi:10.1007/s00213-013-3328-x.

44. Trujillo-Pisanty I, Hernandez G, Moreau-Debord I, Cossette MP, Conover K, Cheer JF, et al. Cannabinoid receptor blockade reduces the opportunity cost at which rats maintain operant performance for rewarding brain stimulation. The Journal of neuroscience : the official journal of the Society for Neuroscience. 2011;31(14):5426–5435. doi:10.1523/JNEUROSCI.0079-11.2011.

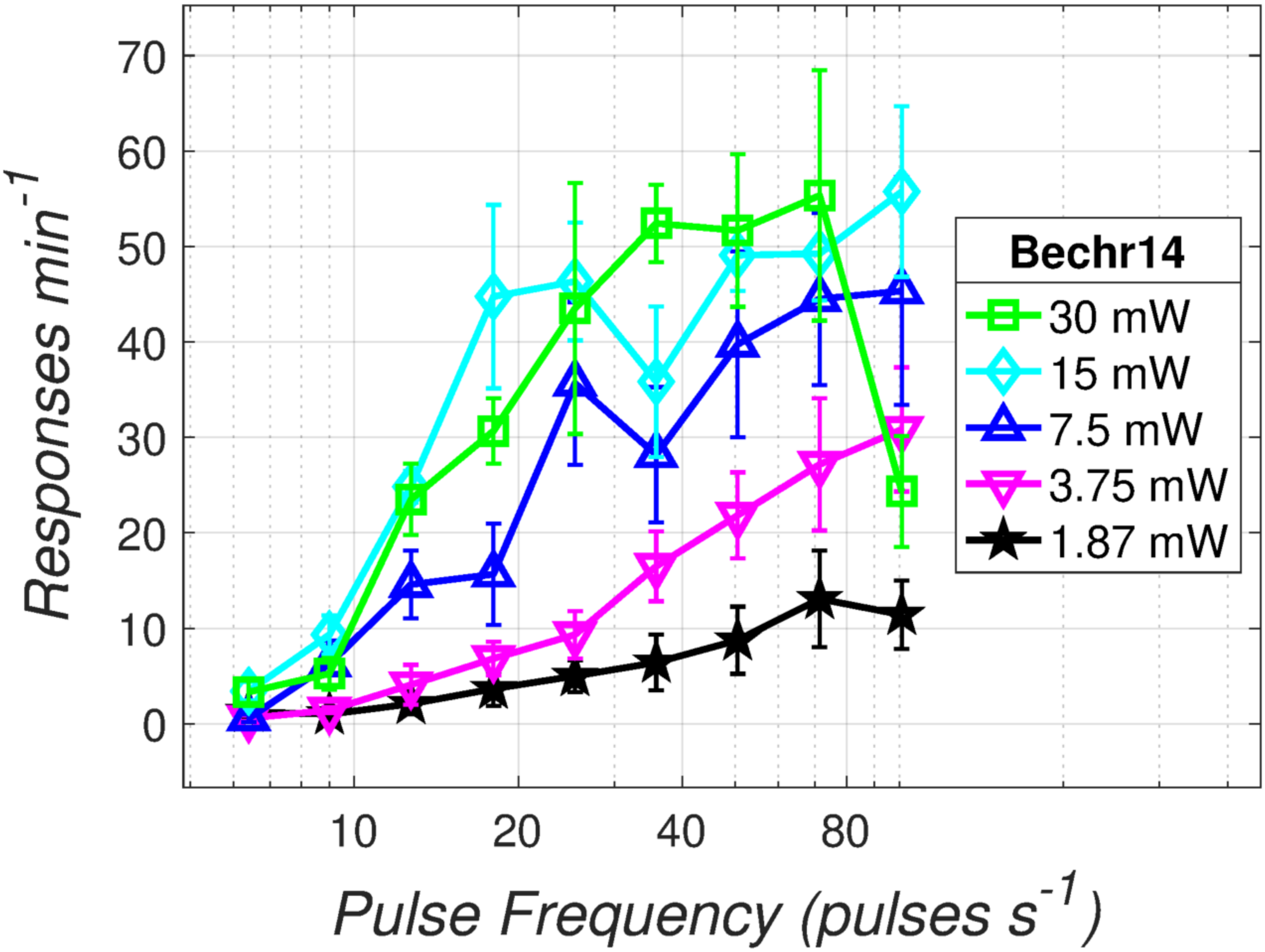
Responses min-1
70
60
50
40
30
20
10
0
10
20
40
80
Pulse Frequency (pulses s-1)
Bechr14
30 mW
15 mW
7.5 mW
3.75 mW
1.87 mW

Figure S1

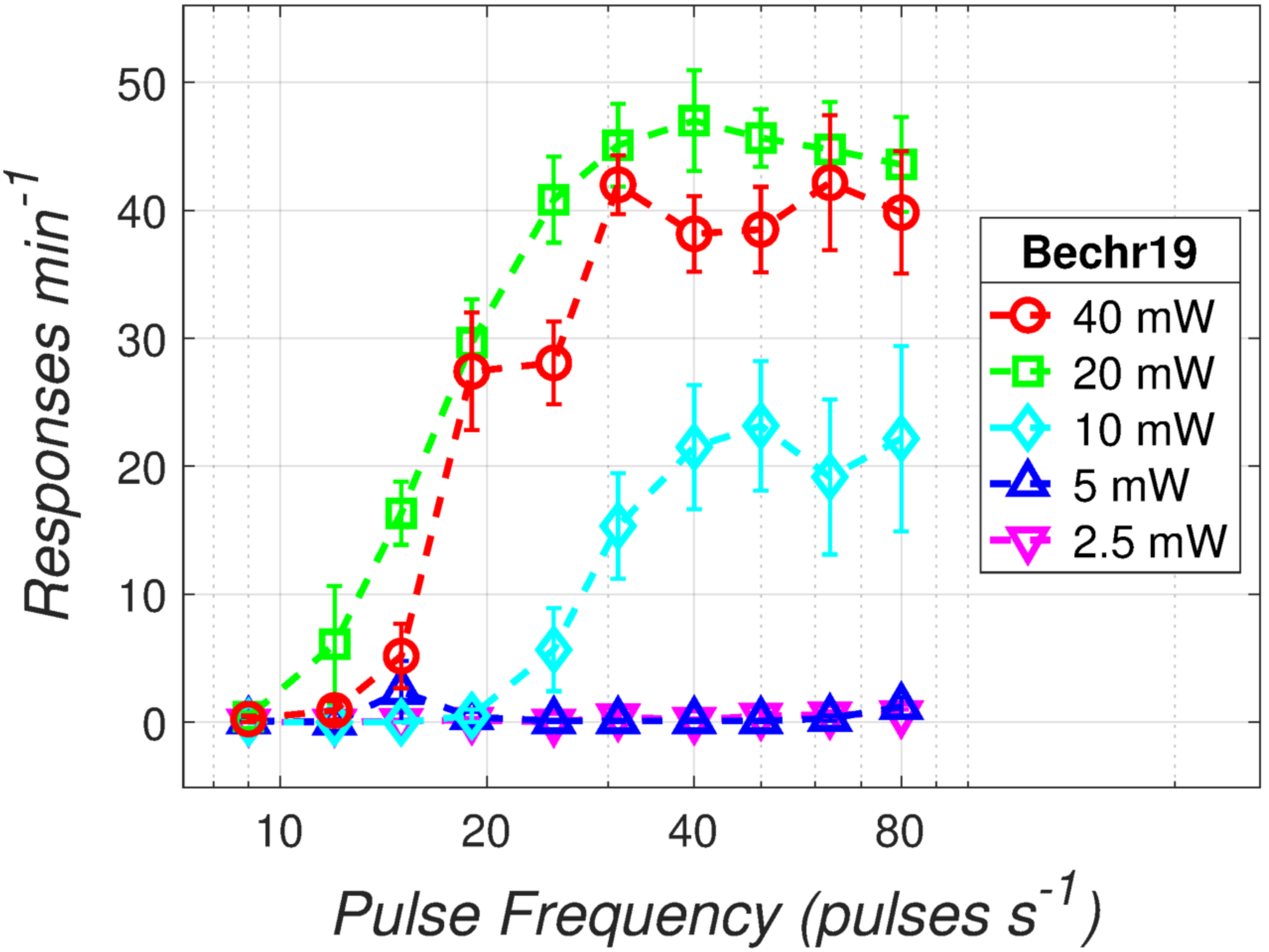
Responses min^{-1}
50
40
30
20
10
0
10
20
40
80
Pulse Frequency (pulses s^{-1})
Bechr19
40 mW
20 mW
10 mW
5 mW
2.5 mW

Figure S2

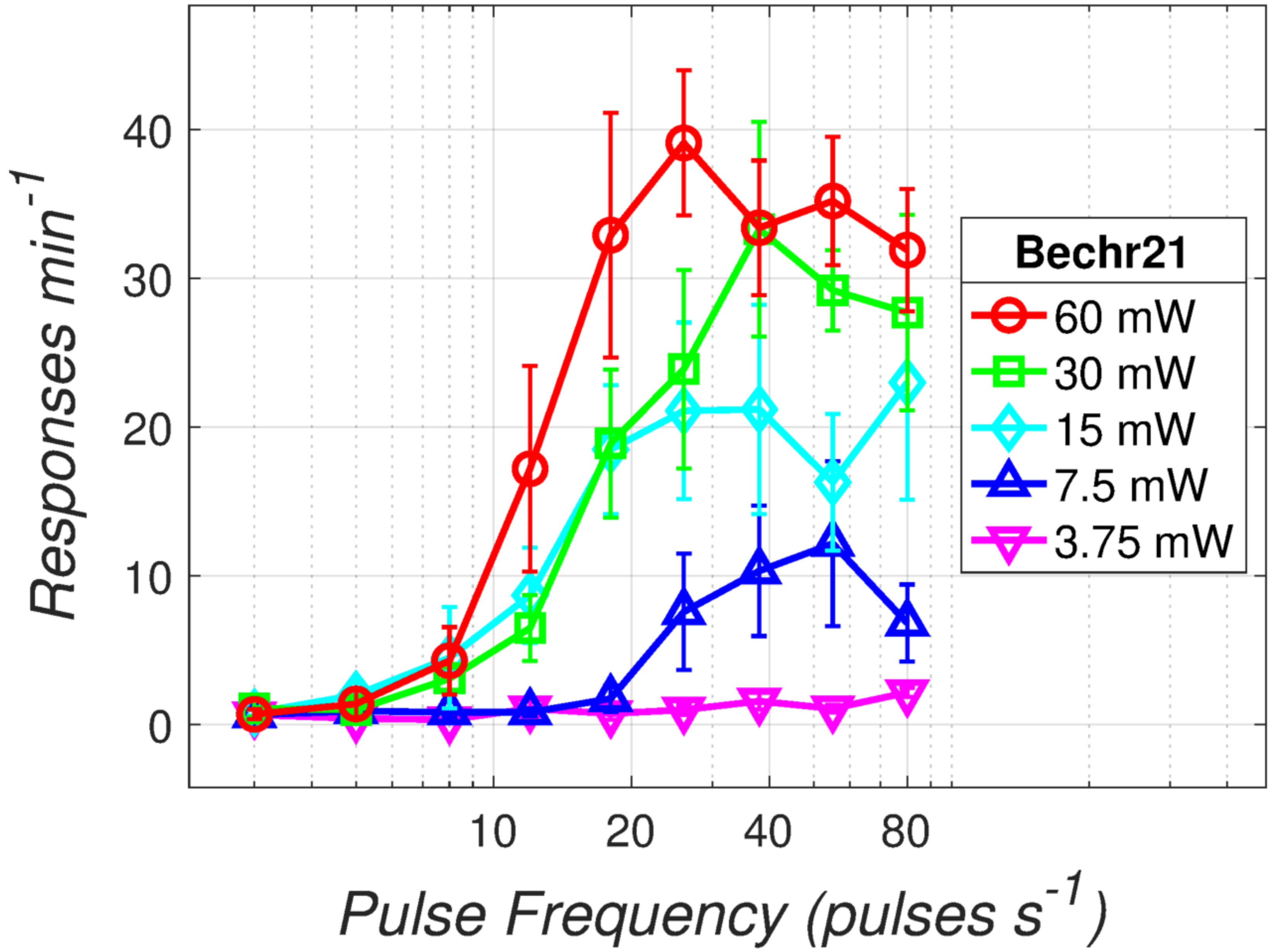
Responses min^-1
Pulse Frequency (pulses s^-1)
0
10
20
30
40
10
20
40
80
Bechr21
60 mW
30 mW
15 mW
7.5 mW
3.75 mW

Figure S3

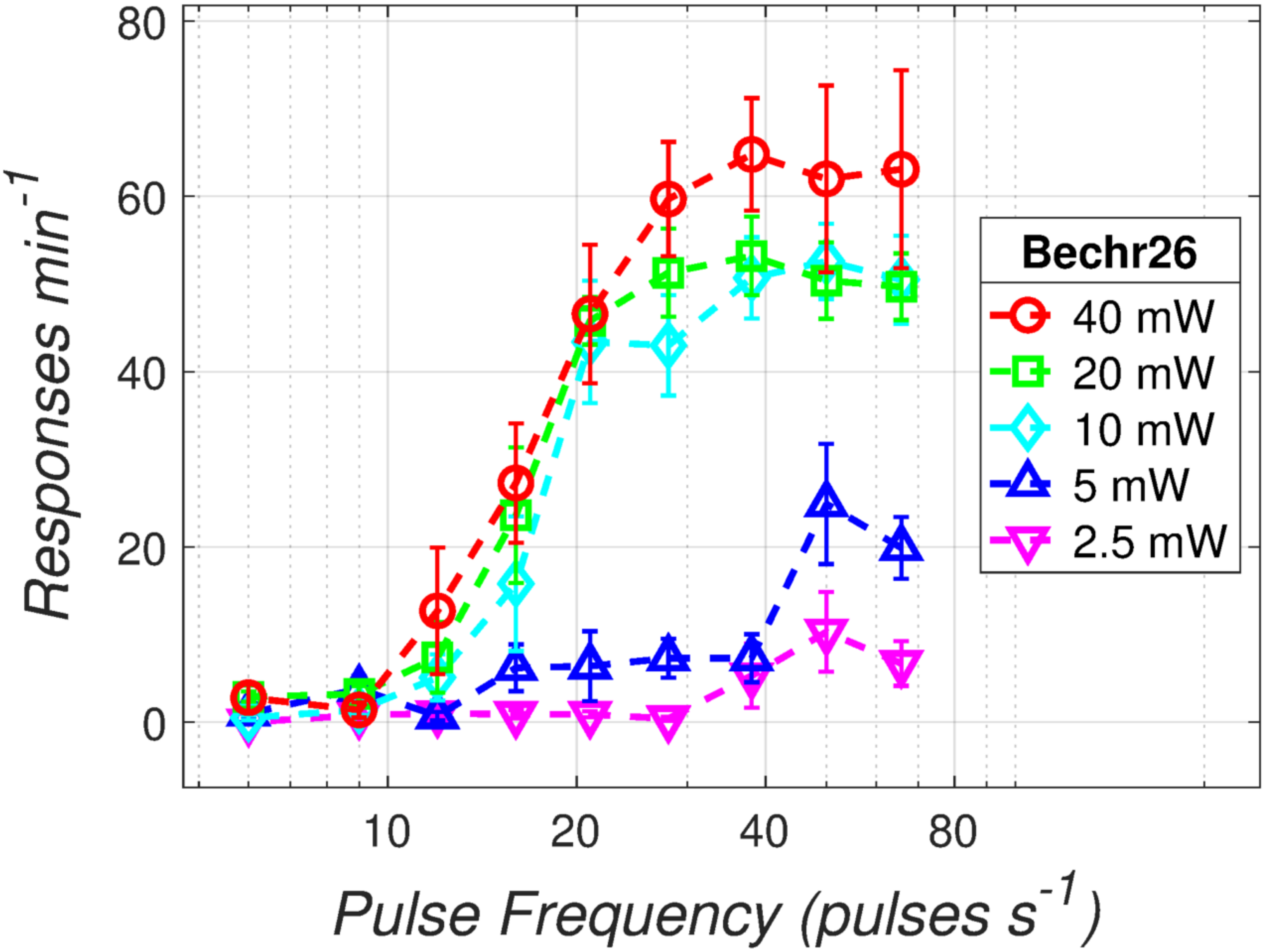

80
60
40
20
0
Responses min^{-1}
10
20
40
80
Pulse Frequency (pulses s^{-1})
Bechr26
40 mW
20 mW
10 mW
5 mW
2.5 mW

Figure S4

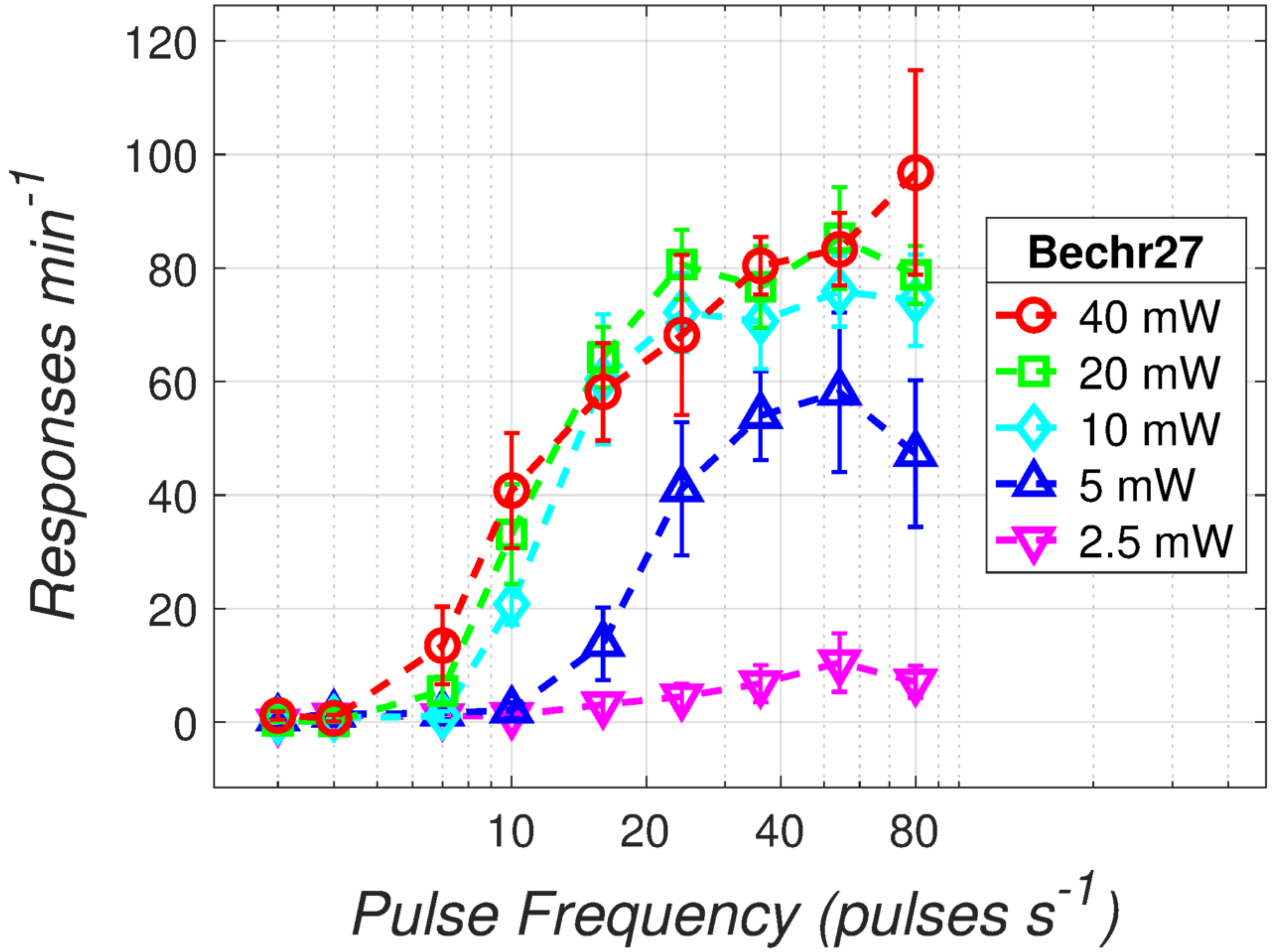
Bechr27
40 mW
20 mW
10 mW
5 mW
2.5 mW
Responses min-1
Pulse Frequency (pulses s-1)
0
20
40
60
80
100
120
10
20
40
80

Figure S5

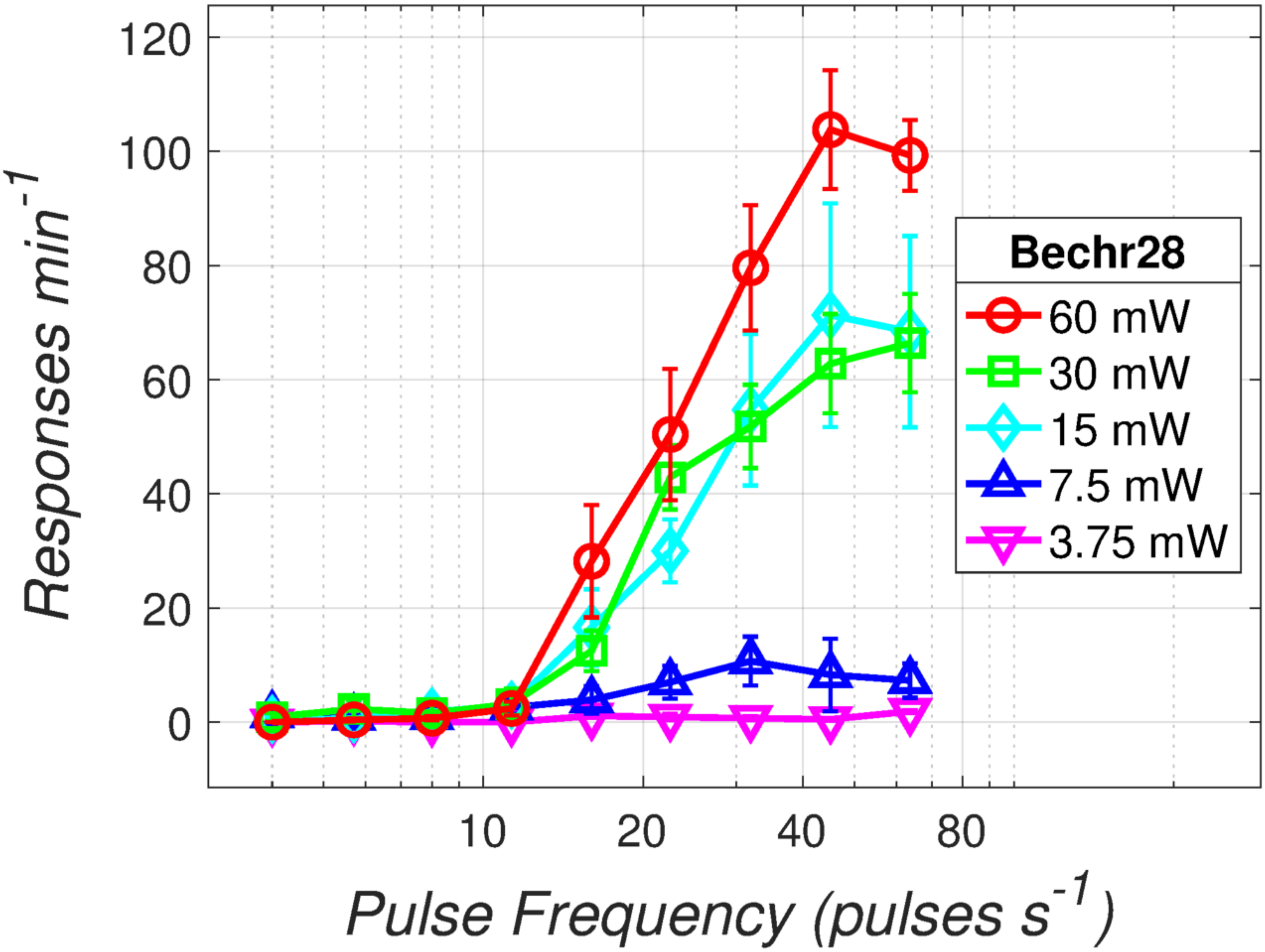

Responses min^{-1}
120
100
80
60
40
20
0
10
20
40
80
Pulse Frequency (pulses s^{-1})
Bechr28
60 mW
30 mW
15 mW
7.5 mW
3.75 mW

Figure S6

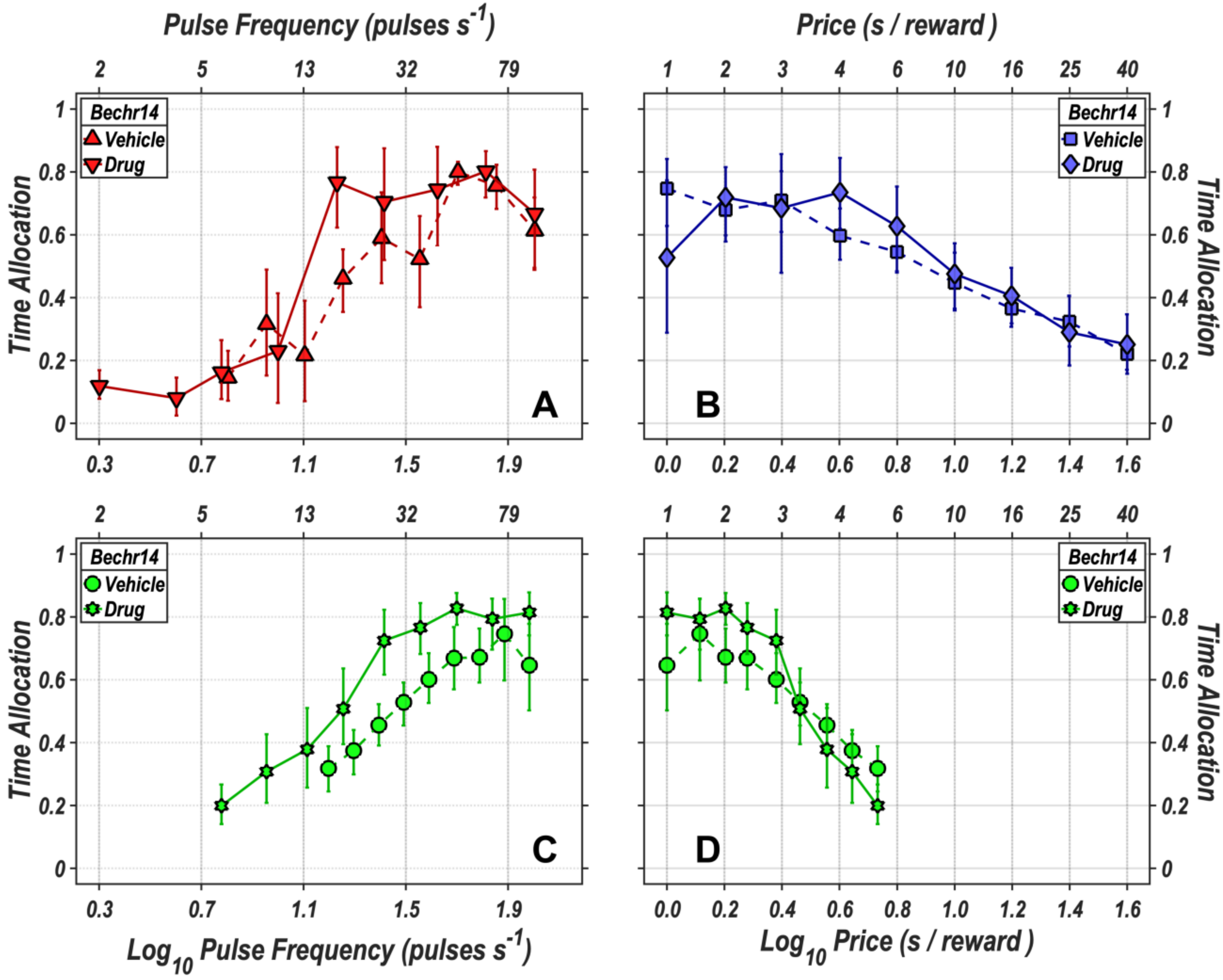

Pulse Frequency (pulses s-1)
Price (s / reward)
Bechr14
Vehicle
Drug
Time Allocation
A
B
C
D
Log10 Pulse Frequency (pulses s-1)
Log10 Price (s / reward)

Figure S7

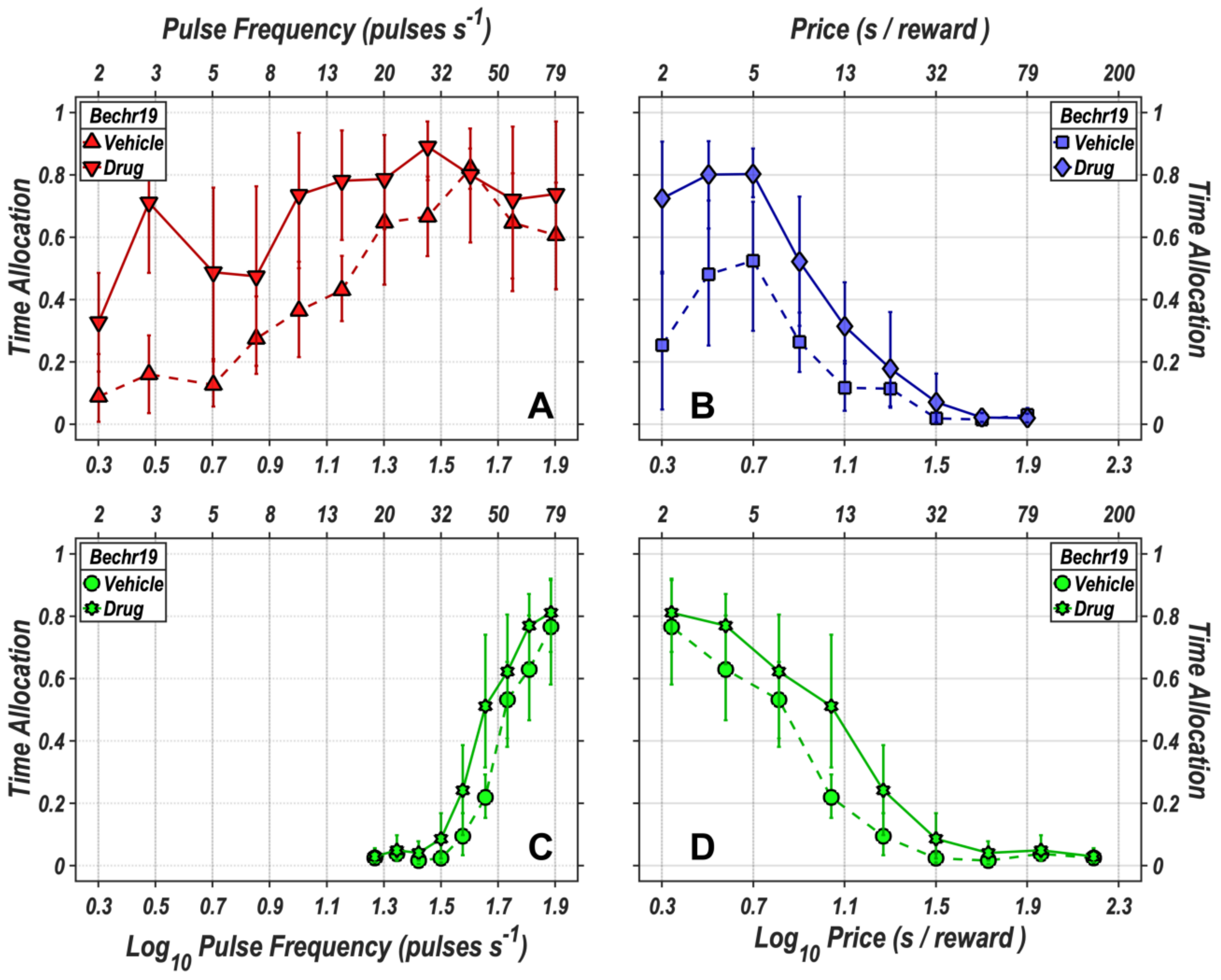

Pulse Frequency (pulses s-1)
Price (s / reward)
2 3 5 8 13 20 32 50 79
2 5 13 32 79 200
Bechr19
Vehicle
Drug
Time Allocation
1 0.8 0.6 0.4 0.2 0
0.3 0.5 0.7 0.9 1.1 1.3 1.5 1.7 1.9
0.3 0.7 1.1 1.5 1.9 2.3
A
B
C
D
Log10 Pulse Frequency (pulses s-1)
Log10 Price (s / reward)

Figure S8

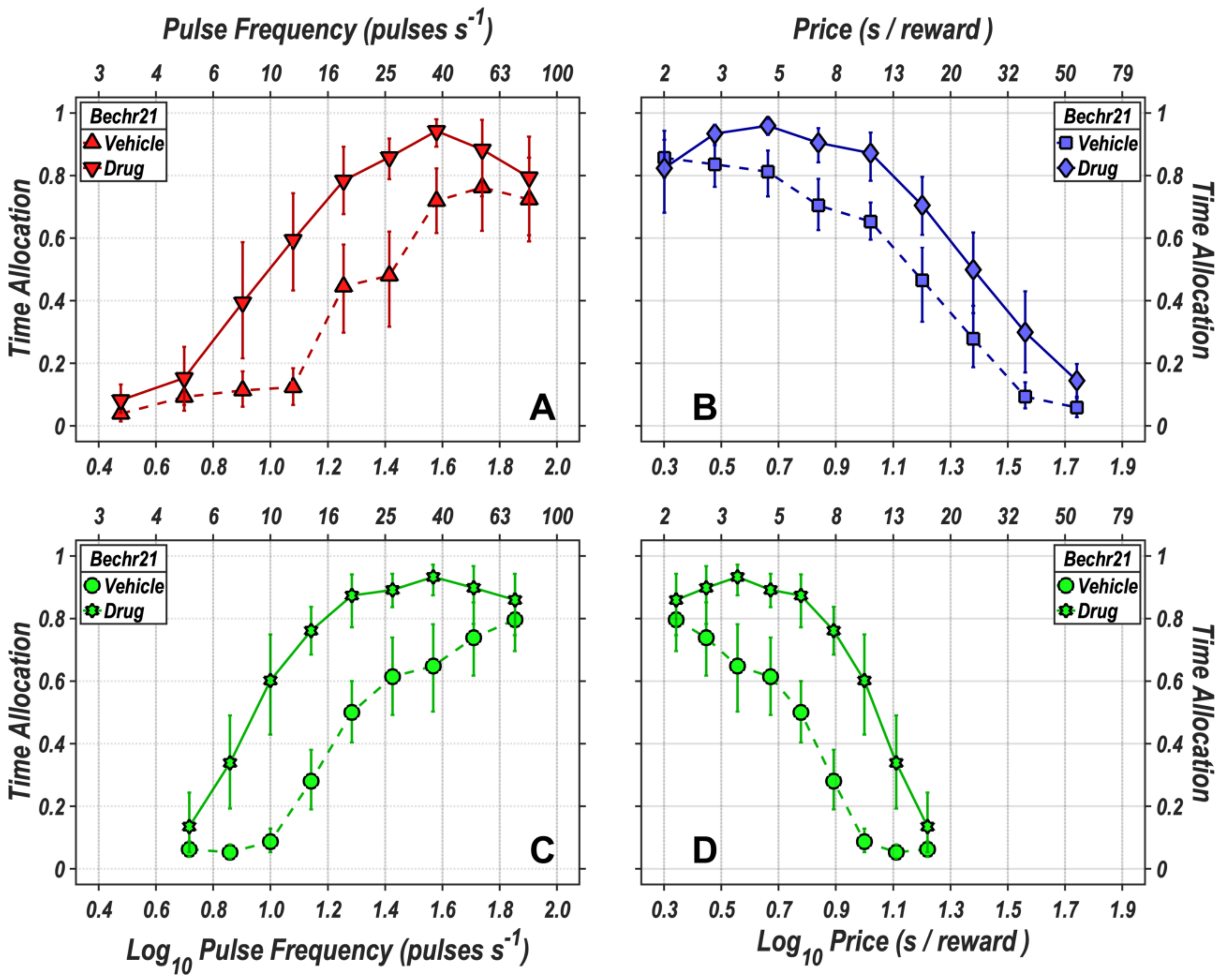
Pulse Frequency (pulses s-1)
Price (s / reward)
Bechr21
Vehicle
Drug
Time Allocation
A
B
C
D
Log10 Pulse Frequency (pulses s-1)
Log10 Price (s / reward)

Figure S9

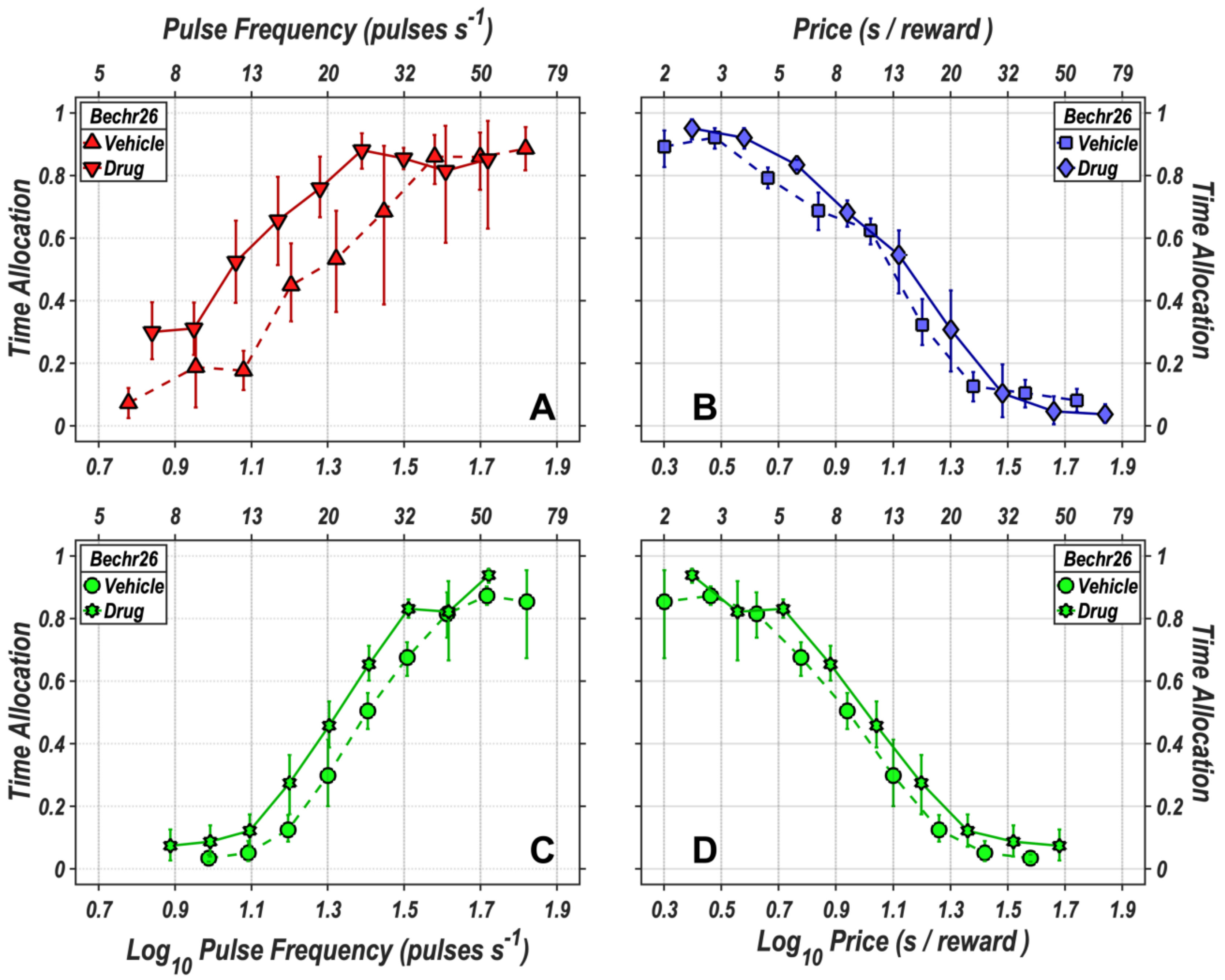

Pulse Frequency (pulses s-1)
Price (s / reward)
5
8
13
20
32
50
79
2
3
5
8
13
20
32
50
79
Bechr26
Vehicle
Drug
Time Allocation
A
B
C
D
0.7
0.9
1.1
1.3
1.5
1.7
1.9
0.3
0.5
0.7
0.9
1.1
1.3
1.5
1.7
1.9
0
0.2
0.4
0.6
0.8
1
Log10 Pulse Frequency (pulses s-1)
Log10 Price (s / reward)

Figure S10

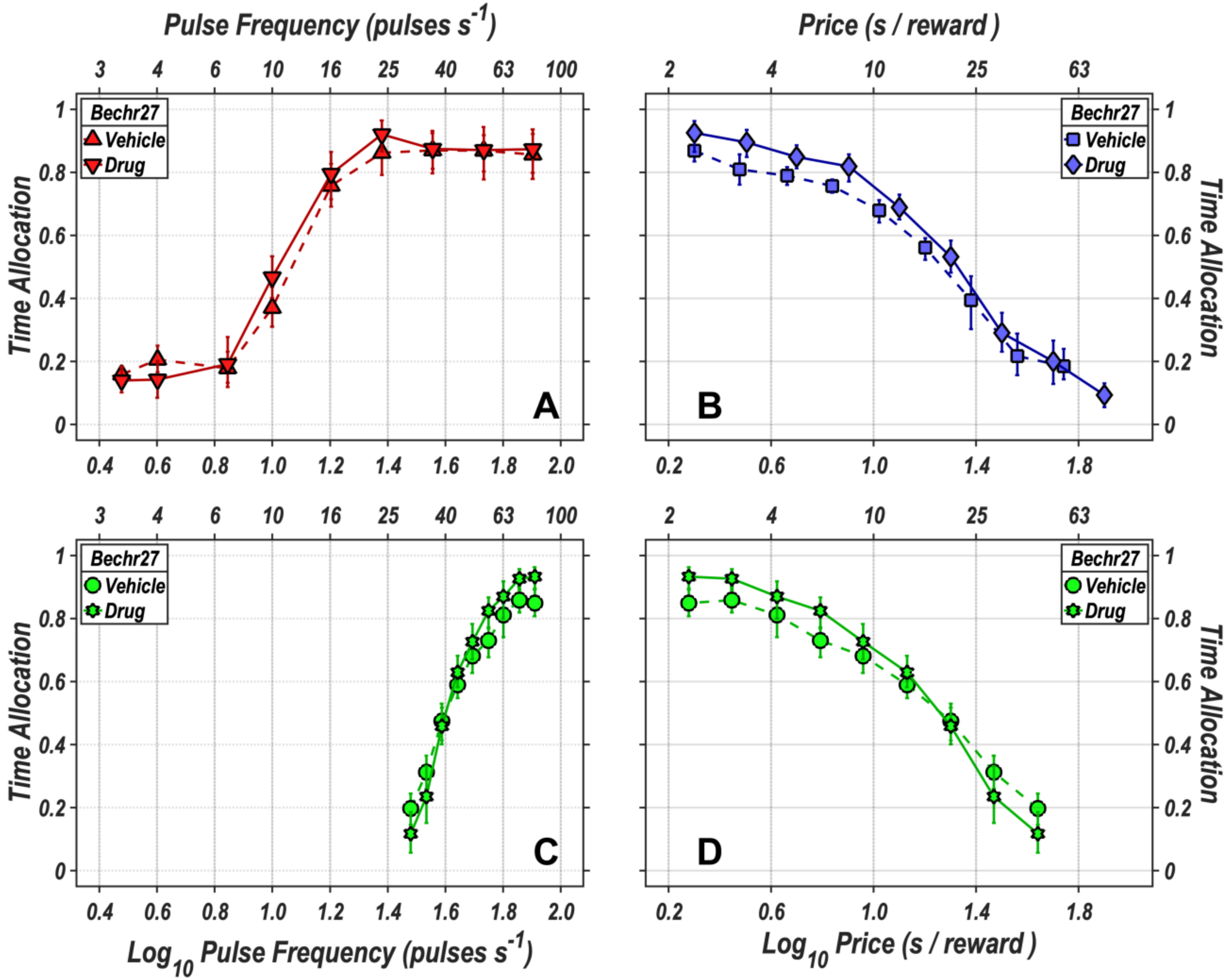
Pulse Frequency (pulses s-1)
Price (s / reward)
Bechr27
Vehicle
Drug
Time Allocation
A
B
C
D
Log10 Pulse Frequency (pulses s-1)
Log10 Price (s / reward)

Figure S11

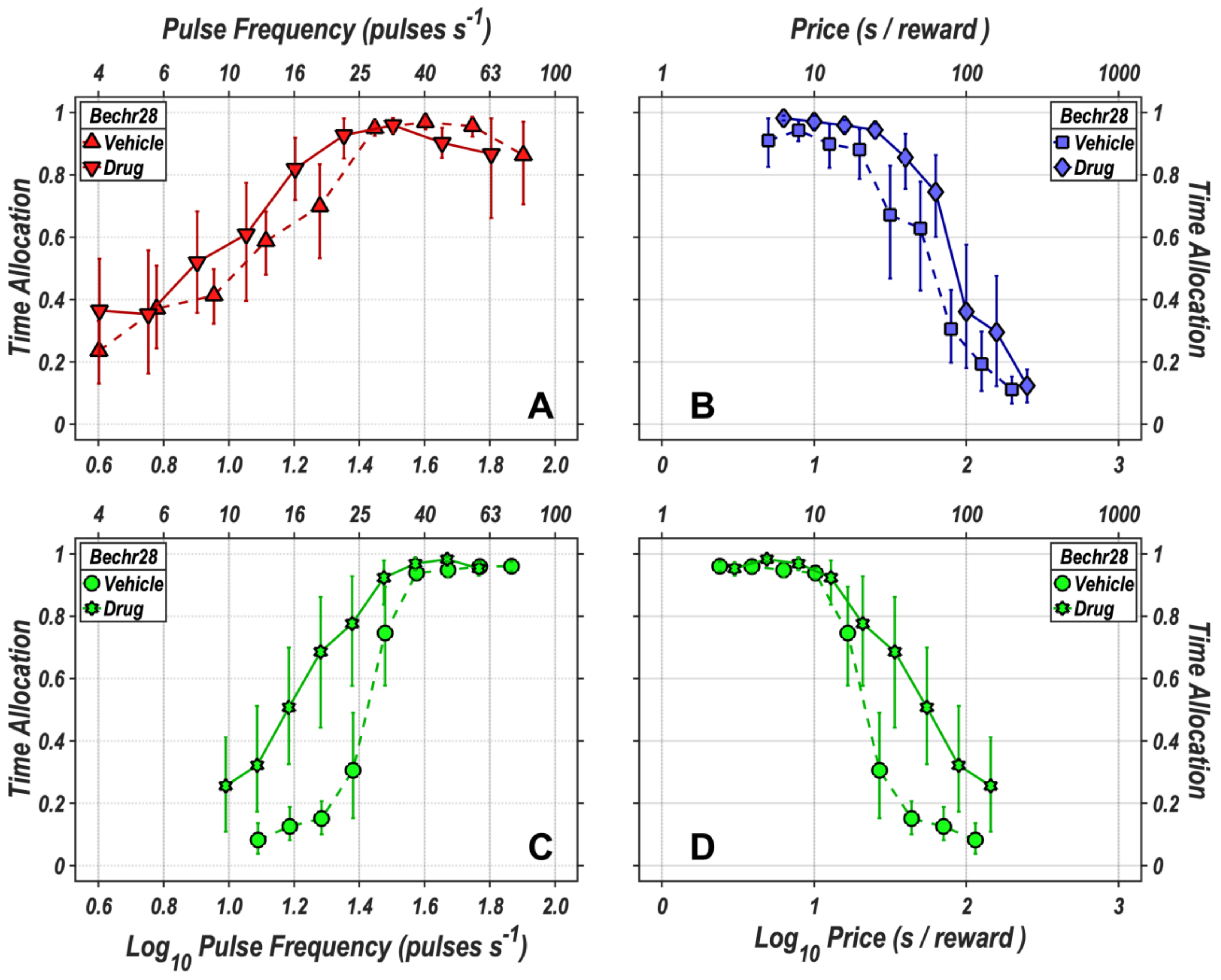
Pulse Frequency (pulses s-1)
4
6
10
16
25
40
63
100
Bechr28
Vehicle
Drug
Time Allocation
A
0.6
0.8
1.0
1.2
1.4
1.6
1.8
2.0
Price (s / reward)
1
10
100
1000
B
0
1
2
3
C
D
Log10 Pulse Frequency (pulses s-1)
Log10 Price (s / reward)

Figure S12

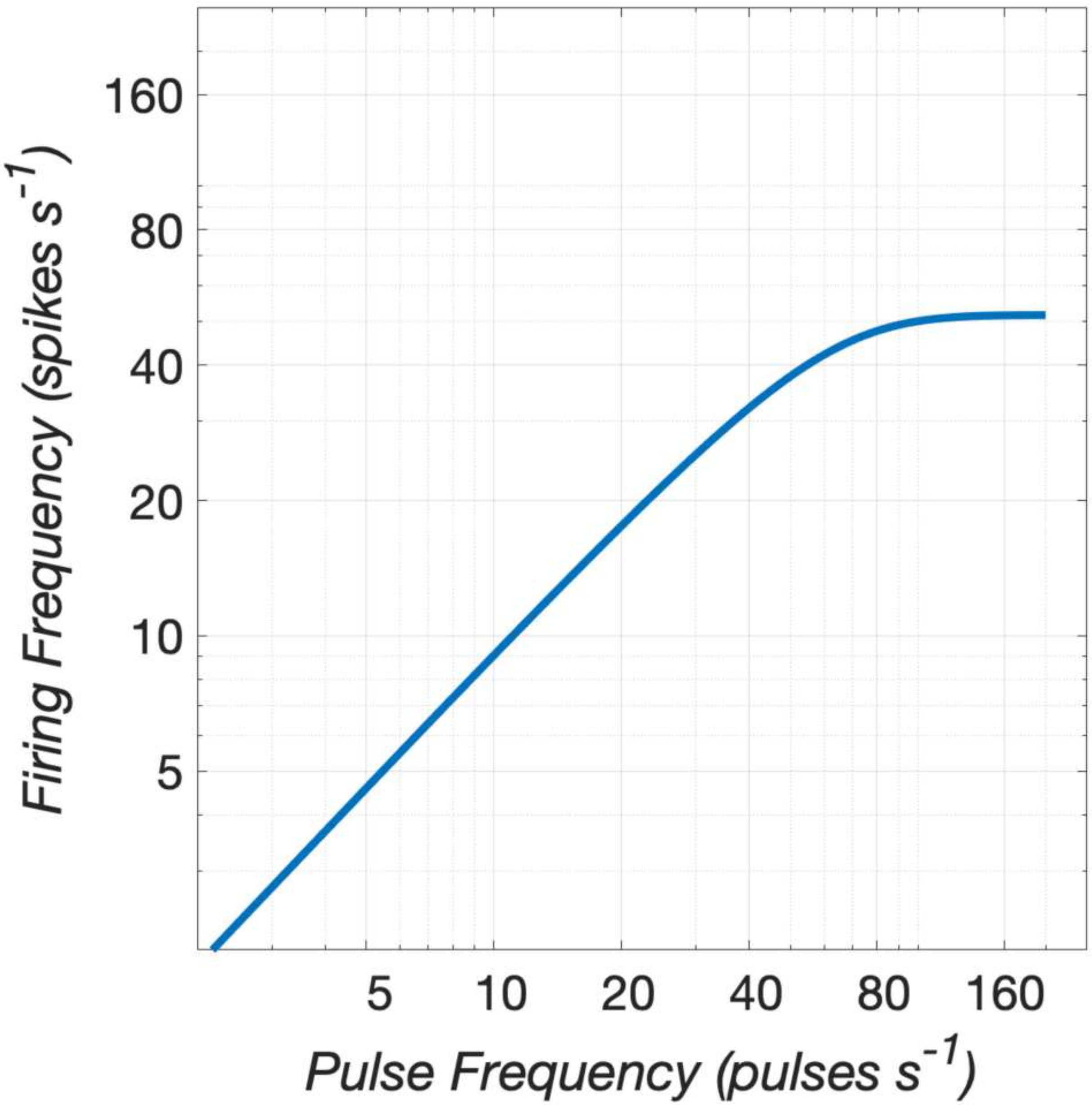
160
80
40
20
10
5
Firing Frequency (spikes s^{-1})
5
10
20
40
80
160
Pulse Frequency (pulses s^{-1})

Figure S13

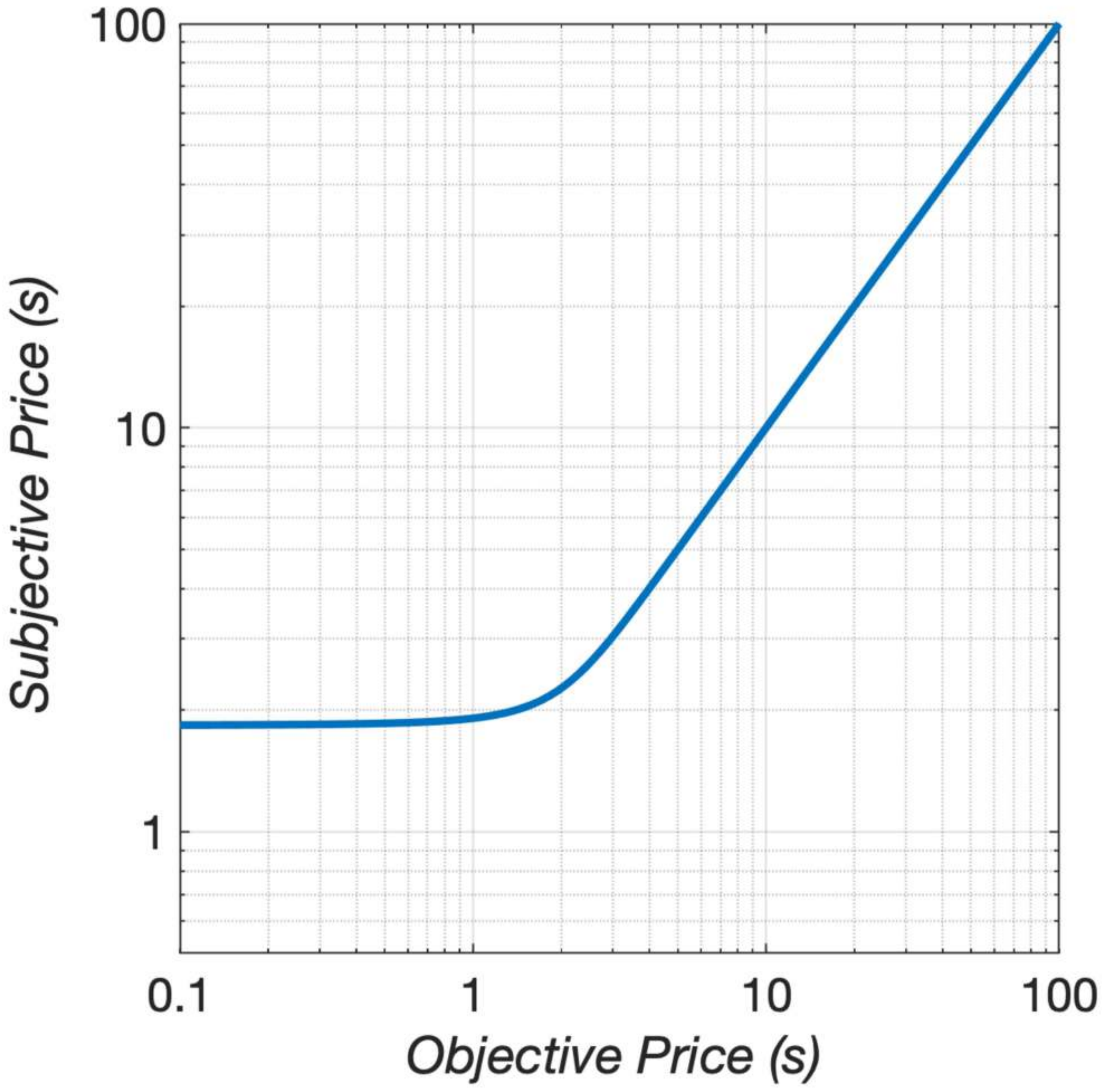

Figure S14

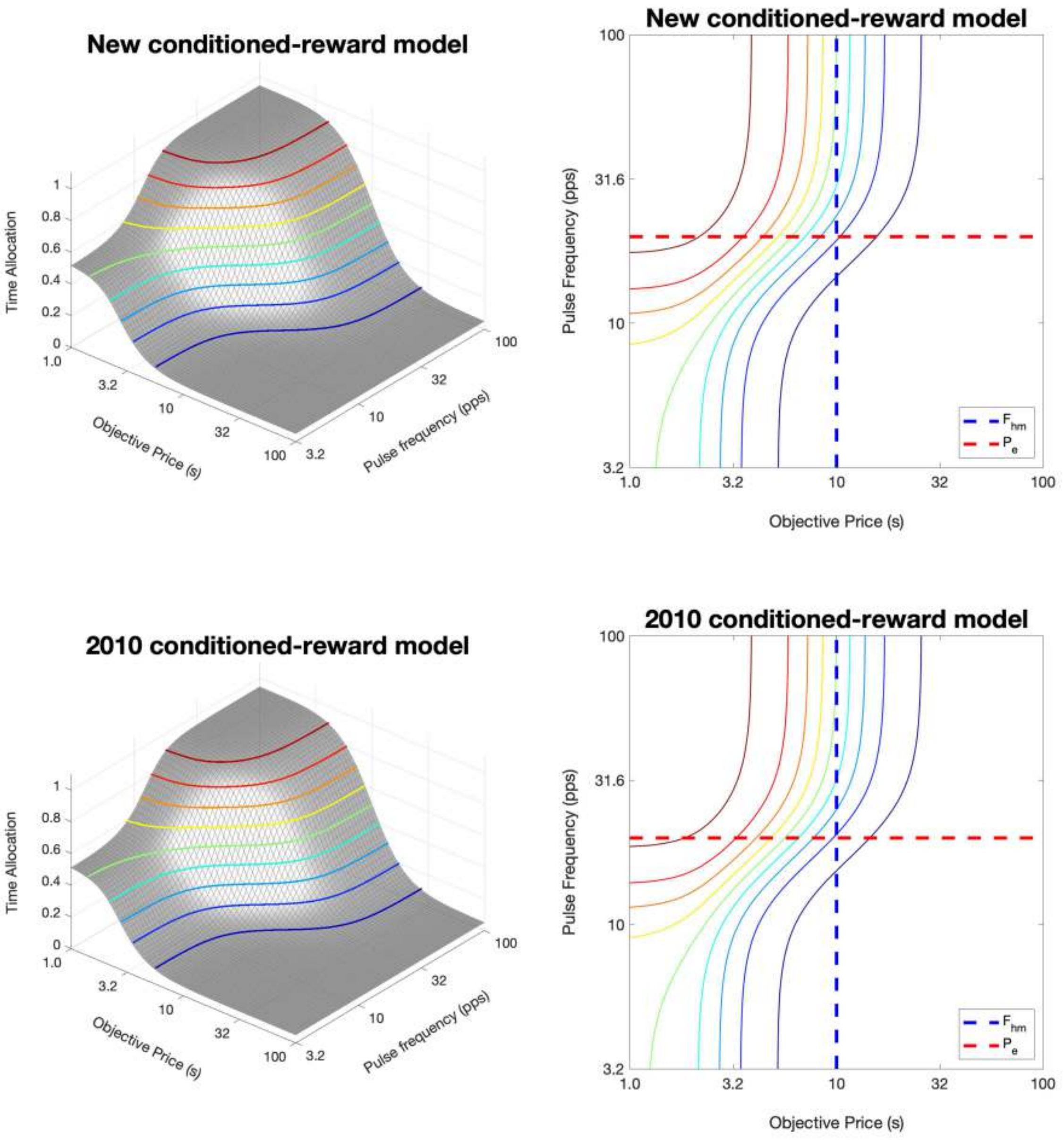
New conditioned-reward model
Time Allocation
0
0.2
0.4
0.6
0.8
1
1.0
3.2
10
32
100
Objective Price (s)
3.2
10
32
100
Pulse frequency (pps)
New conditioned-reward model
Pulse Frequency (pps)
3.2
10
31.6
100
1.0
3.2
10
32
100
Objective Price (s)
F_{hm}
P_e
2010 conditioned-reward model
Time Allocation
0
0.2
0.4
0.6
0.8
1
1.0
3.2
10
32
100
Objective Price (s)
3.2
10
32
100
Pulse frequency (pps)
2010 conditioned-reward model
Pulse Frequency (pps)
3.2
10
31.6
100
1.0
3.2
10
32
100
Objective Price (s)
F_{hm}
P_e

Figure S15

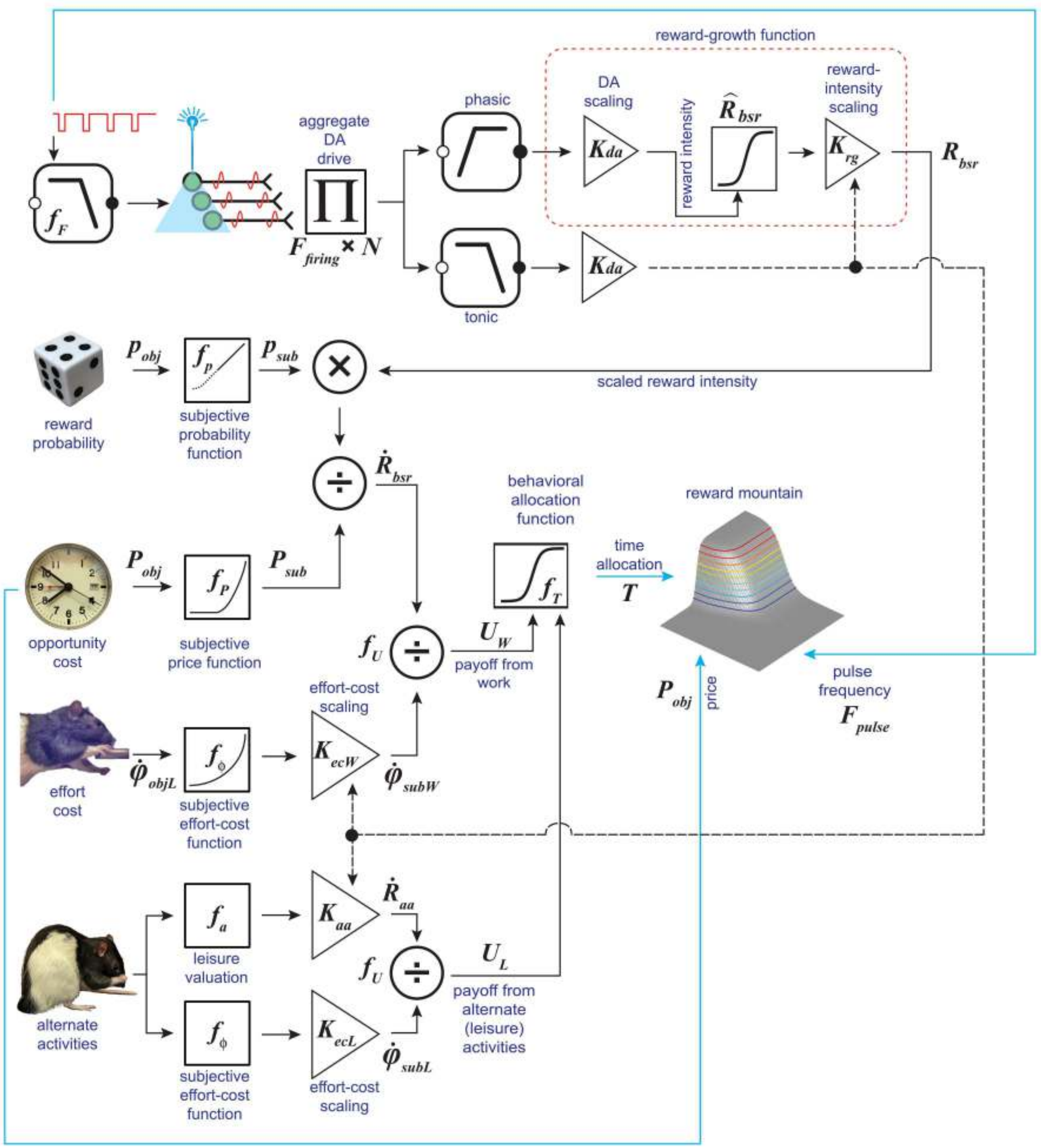

reward-growth function
aggregate DA drive
phasic
DA scaling
reward intensity
$\hat{R}_{bsr}$
reward-intensity scaling
K_{da}
K_{rg}
R_{bsr}
f_F
$F_{firing} \times N$
tonic
p_{obj}
f_p
p_{sub}
reward probability
subjective probability function
scaled reward intensity
$\dot{R}_{bsr}$
behavioral allocation function
reward mountain
P_{obj}
f_P
P_{sub}
time allocation
T
f_T
opportunity cost
subjective price function
f_U
U_W
payoff from work
effort-cost scaling
P_{obj}
price
pulse frequency
F_{pulse}
$\dot{\varphi}_{objL}$
f_ϕ
K_{ecW}
$\dot{\varphi}_{subW}$
effort cost
subjective effort-cost function
f_a
K_{aa}
$\dot{R}_{aa}$
U_L
leisure valuation
payoff from alternate (leisure) activities
alternate activities
K_{ecL}
$\dot{\varphi}_{subL}$
subjective effort-cost function
effort-cost scaling

Figure S16

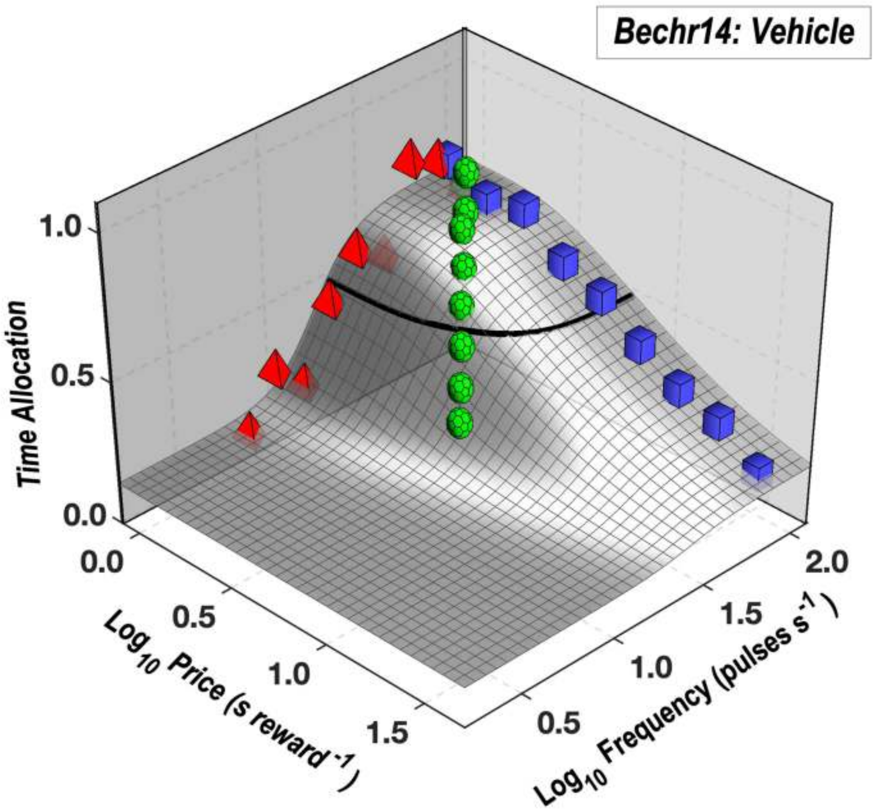

Bechr14: Vehicle
Time Allocation
1.0
0.5
0.0
0.0
0.5
1.0
1.5
Log10 Price (s reward-1)
0.5
1.0
1.5
2.0
Log10 Frequency (pulses s-1)

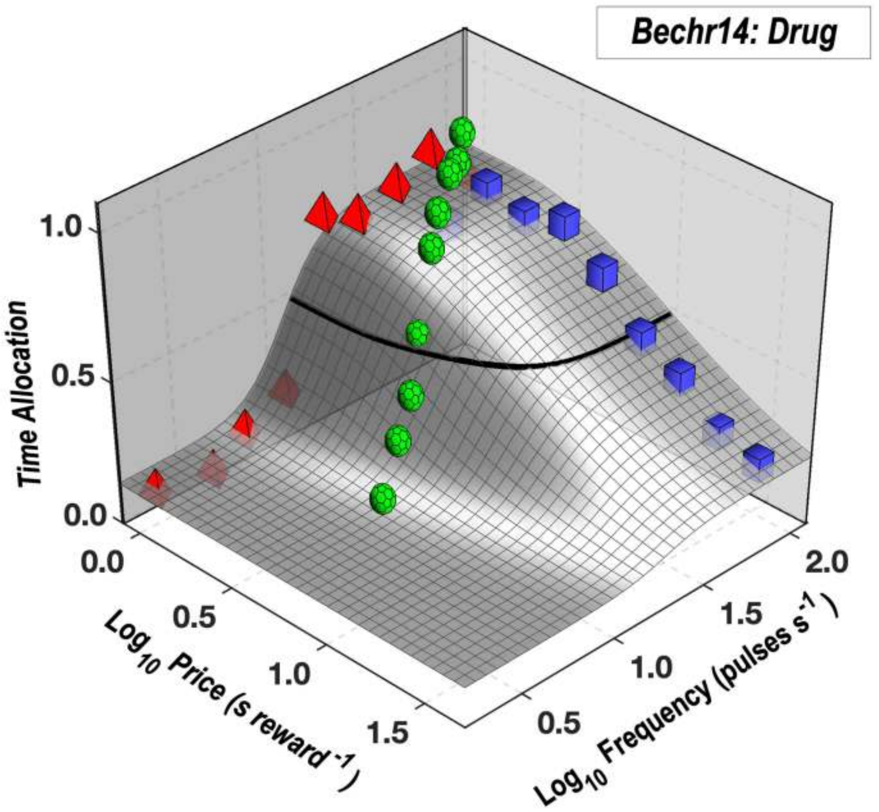

Bechr14: Drug
Time Allocation
1.0
0.5
0.0
0.0
0.5
1.0
1.5
Log10 Price (s reward-1)
0.5
1.0
1.5
2.0
Log10 Frequency (pulses s-1)

Figure S17

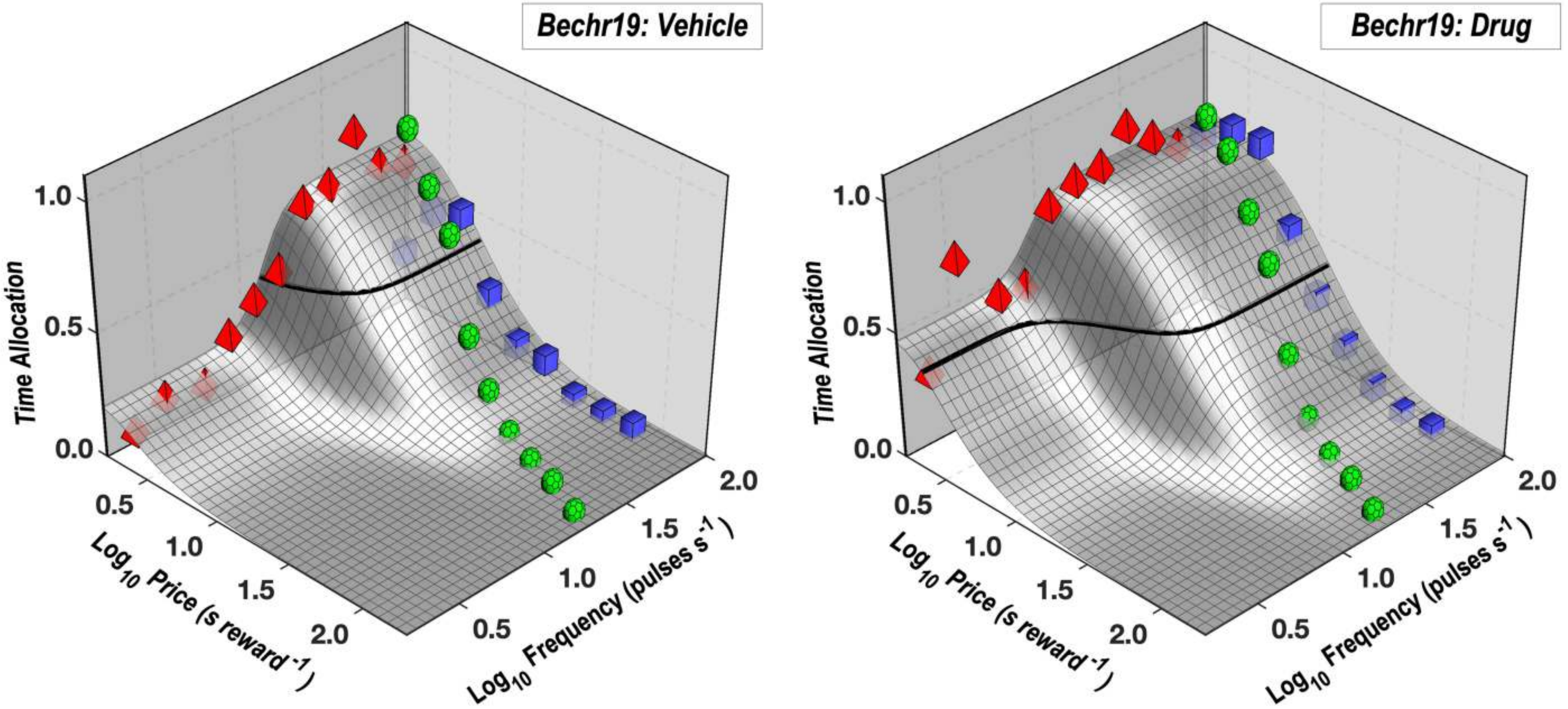
Bechr19: Vehicle
Time Allocation
1.0
0.5
0.0
0.5
1.0
1.5
2.0
Log10 Price (s reward-1)
0.5
1.0
1.5
2.0
Log10 Frequency (pulses s-1)
Bechr19: Drug
Time Allocation
1.0
0.5
0.0
0.5
1.0
1.5
2.0
Log10 Price (s reward-1)
0.5
1.0
1.5
2.0
Log10 Frequency (pulses s-1)

Figure S18

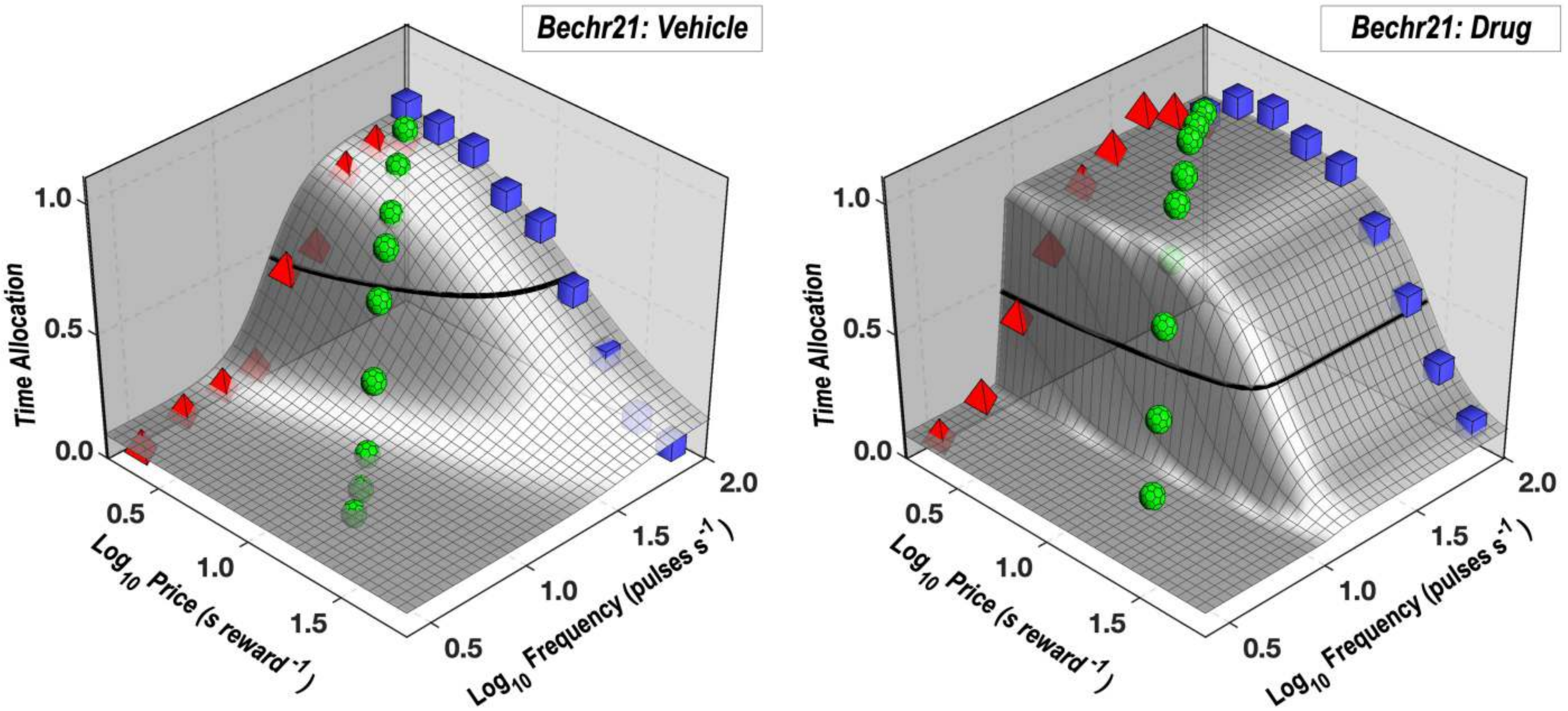

Bechr21: Vehicle
Bechr21: Drug
Time Allocation
1.0
0.5
0.0
0.5
1.0
1.5
2.0
Log10 Price (s reward -1)
Log10 Frequency (pulses s-1)

Figure S19

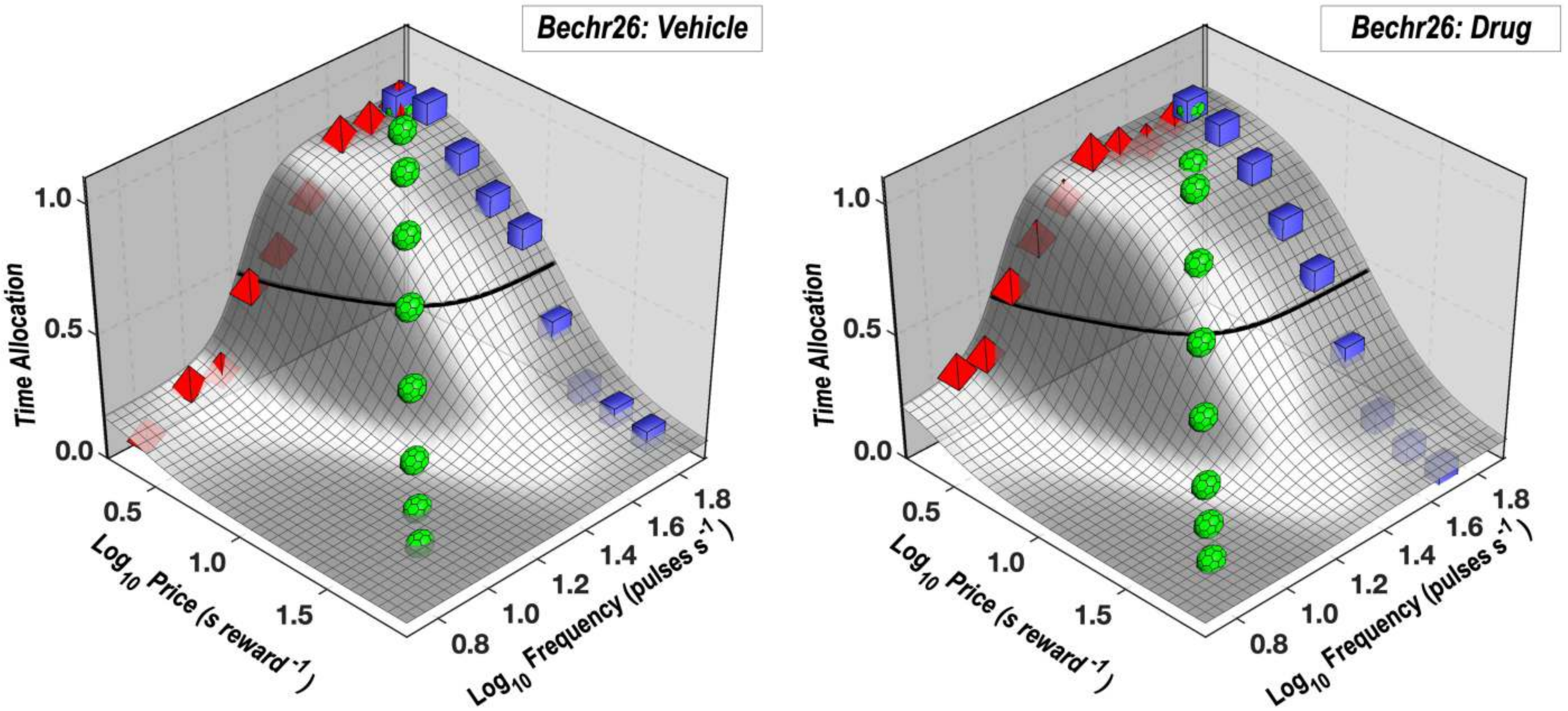
Bechr26: Vehicle
Time Allocation
1.0
0.5
0.0
0.5
1.0
1.5
Log10 Price (s reward -1)
0.8
1.0
1.2
1.4
1.6
1.8
Log10 Frequency (pulses s-1)
Bechr26: Drug
Time Allocation
1.0
0.5
0.0
0.5
1.0
1.5
Log10 Price (s reward -1)
0.8
1.0
1.2
1.4
1.6
1.8
Log10 Frequency (pulses s-1)

Figure S20

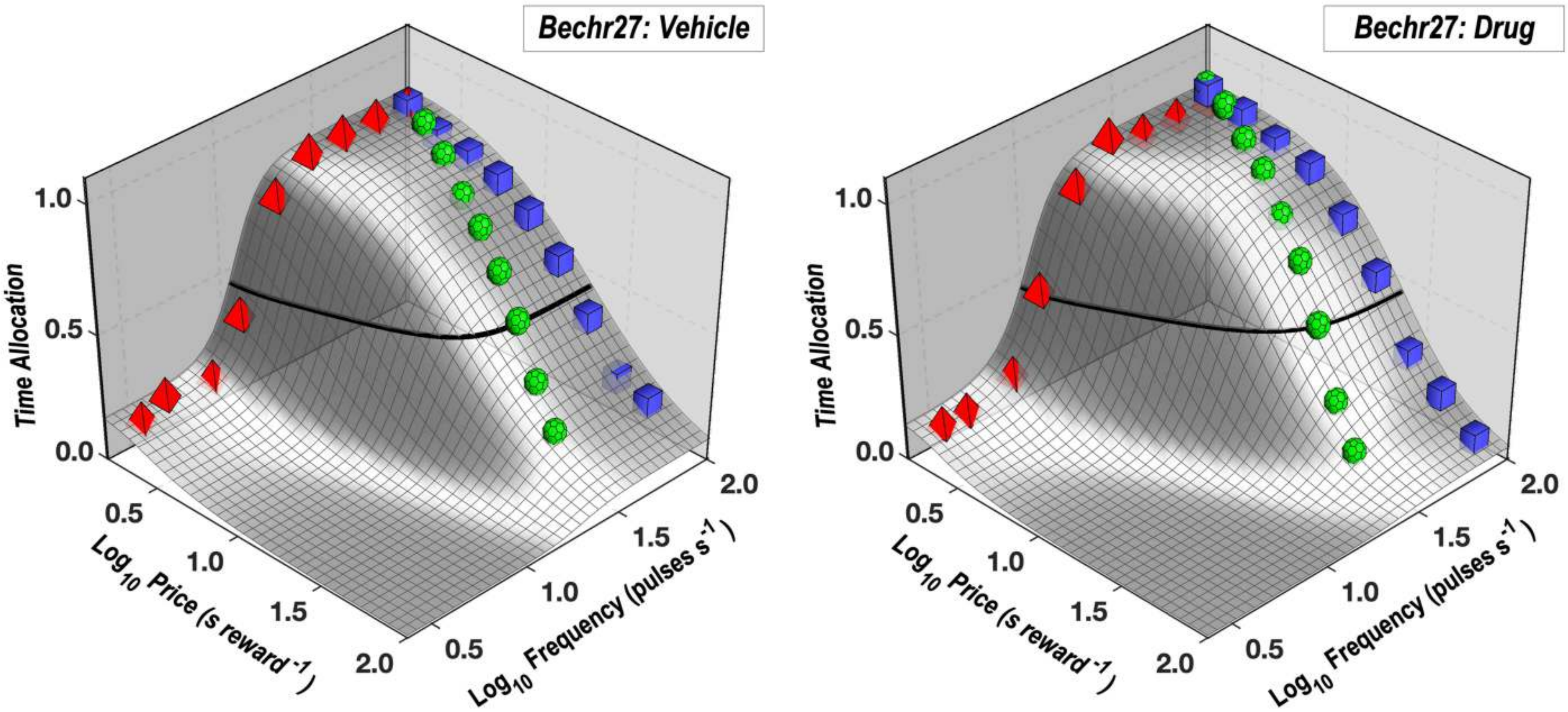

Bechr27: Vehicle
Bechr27: Drug
Time Allocation
0.0
0.5
1.0
Log10 Price (s reward -1)
0.5
1.0
1.5
2.0
Log10 Frequency (pulses s-1)
0.5
1.0
1.5
2.0

Figure S21

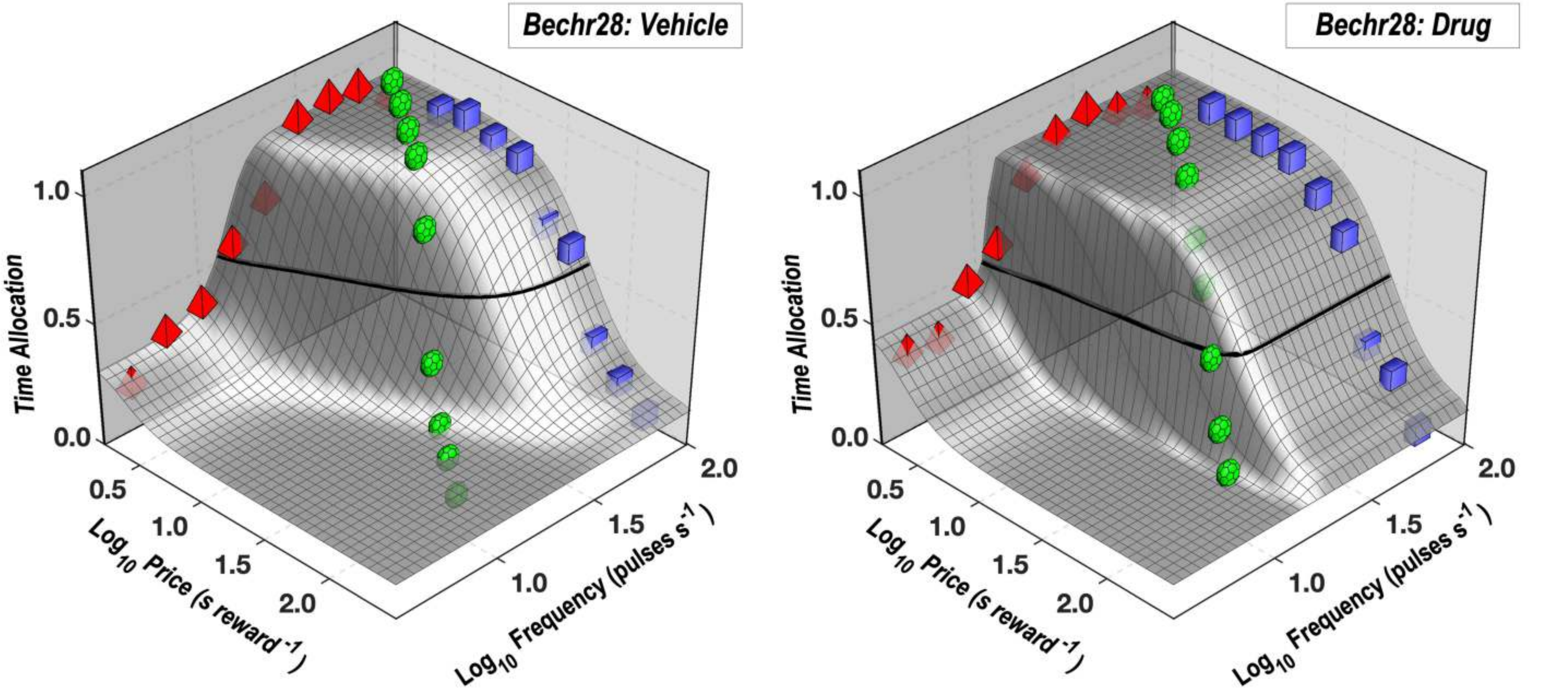

Bechr28: Vehicle
Bechr28: Drug
Time Allocation
1.0
0.5
0.0
0.5
1.0
1.5
2.0
Log10 Price (s reward -1)
Log10 Frequency (pulses s-1)

Figure S22

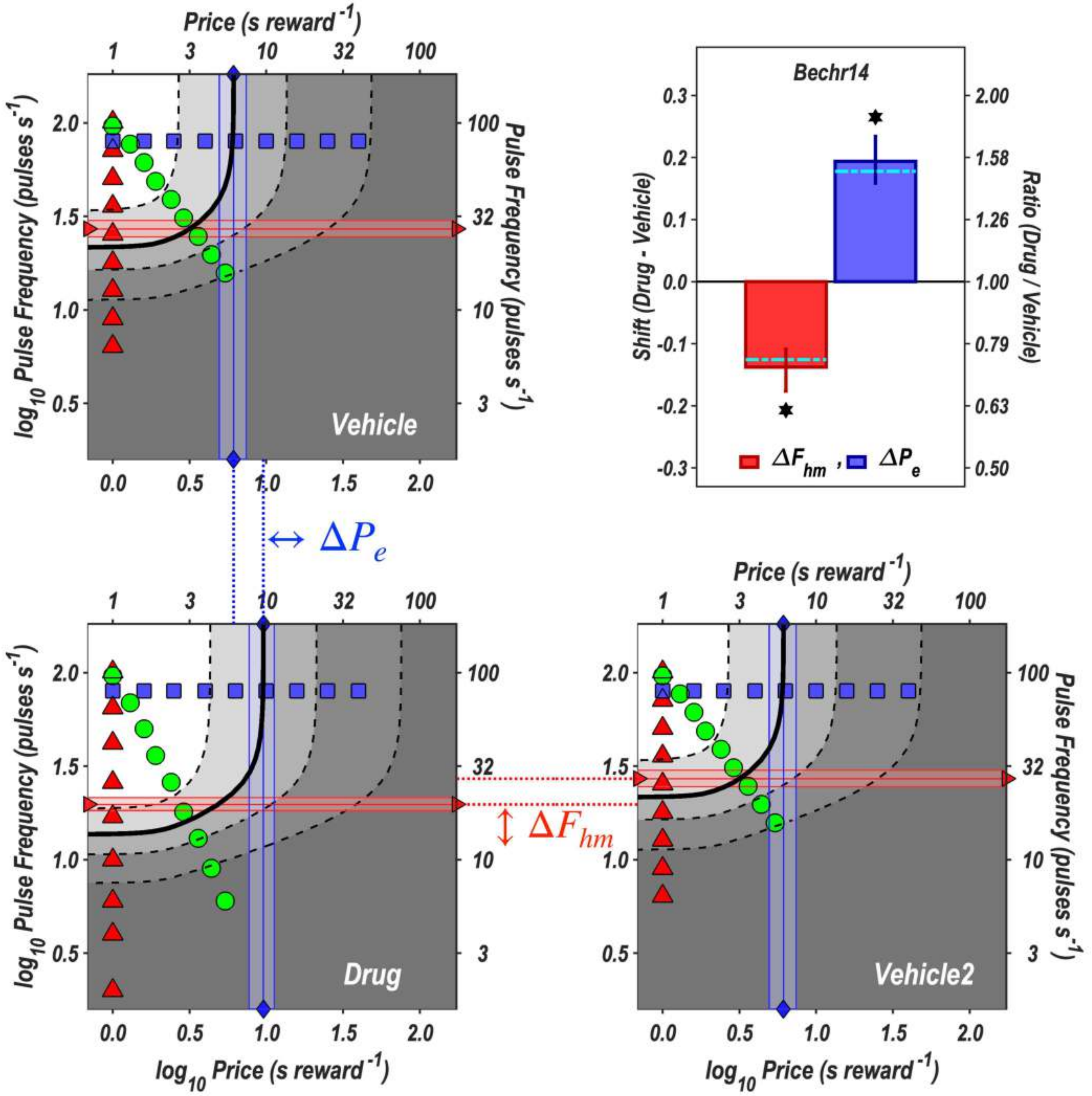

Price (s reward -1)
Vehicle
Drug
Vehicle2
Bechr14
Shift (Drug - Vehicle)
Ratio (Drug / Vehicle)
ΔF_hm , ΔP_e
log10 Pulse Frequency (pulses s-1)
Pulse Frequency (pulses s-1)
log10 Price (s reward -1)

Figure S23

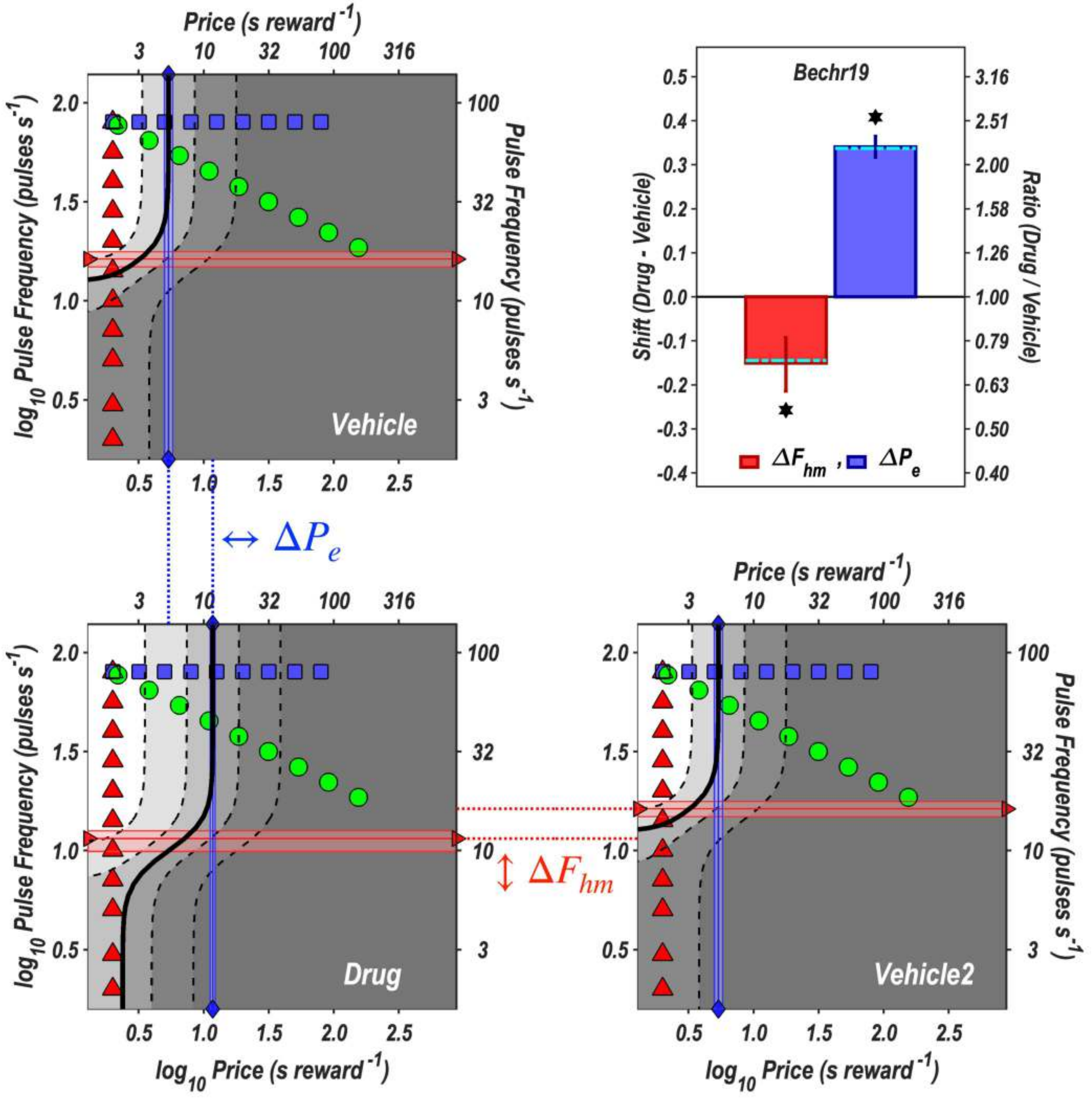

Price (s reward^{-1})
3
10
32
100
316
log$_{10}$ Pulse Frequency (pulses s^{-1})
Pulse Frequency (pulses s^{-1})
Vehicle
Drug
Vehicle2
log$_{10}$ Price (s reward^{-1})
$\leftrightarrow \Delta P_e$
$\updownarrow \Delta F_{hm}$
Bechr19
Shift (Drug - Vehicle)
Ratio (Drug / Vehicle)
ΔF_{hm} , ΔP_e

Figure S24

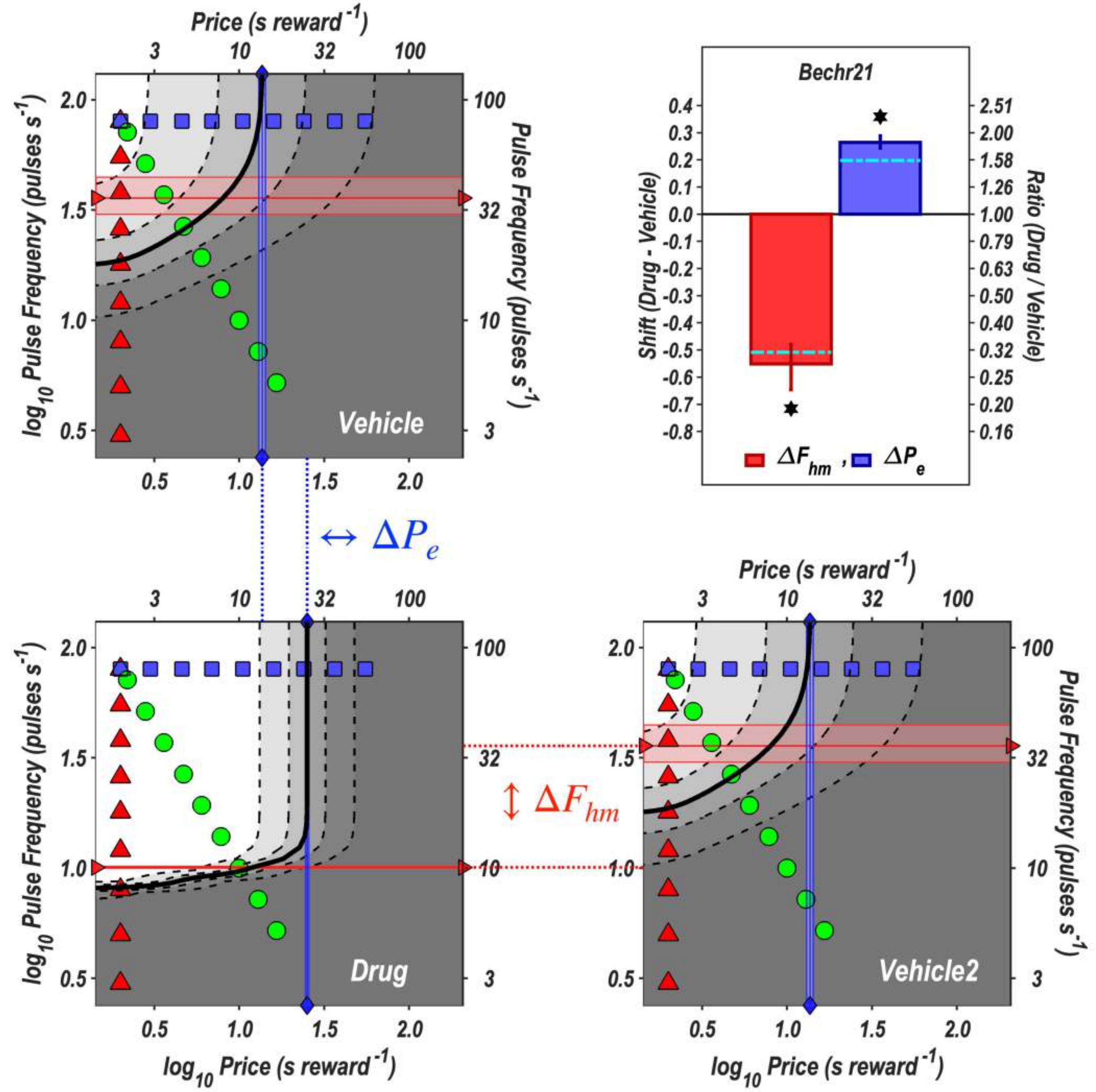
Price (s reward^{-1})
$\log_{10}$ Pulse Frequency (pulses s^{-1})
Pulse Frequency (pulses s^{-1})
Vehicle
Bechr21
Shift (Drug - Vehicle)
Ratio (Drug / Vehicle)
ΔF_{hm}, ΔP_e
$\leftrightarrow \Delta P_e$
$\updownarrow \Delta F_{hm}$
Drug
Vehicle2
$\log_{10}$ Price (s reward^{-1})

Figure S25

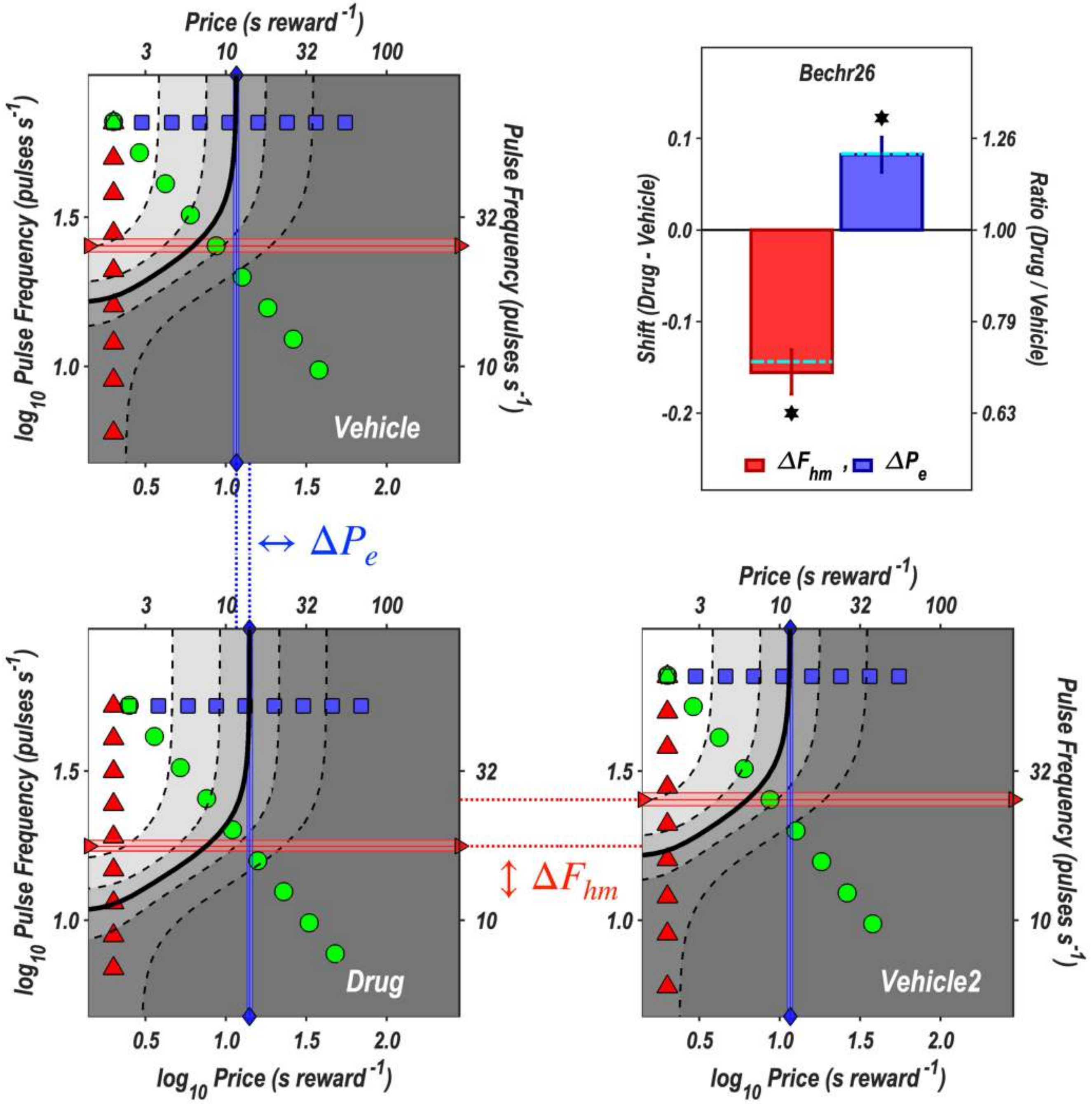
Price (s reward -1)
3
10
32
100
log10 Pulse Frequency (pulses s-1)
Pulse Frequency (pulses s-1)
1.5
1.0
32
10
Vehicle
0.5
1.0
1.5
2.0
Bechr26
Shift (Drug - Vehicle)
Ratio (Drug / Vehicle)
0.1
0.0
-0.1
-0.2
1.26
1.00
0.79
0.63
ΔFhm , ΔPe
↔ ΔPe
Drug
Vehicle2
↕ ΔFhm
log10 Price (s reward -1)

Figure S26

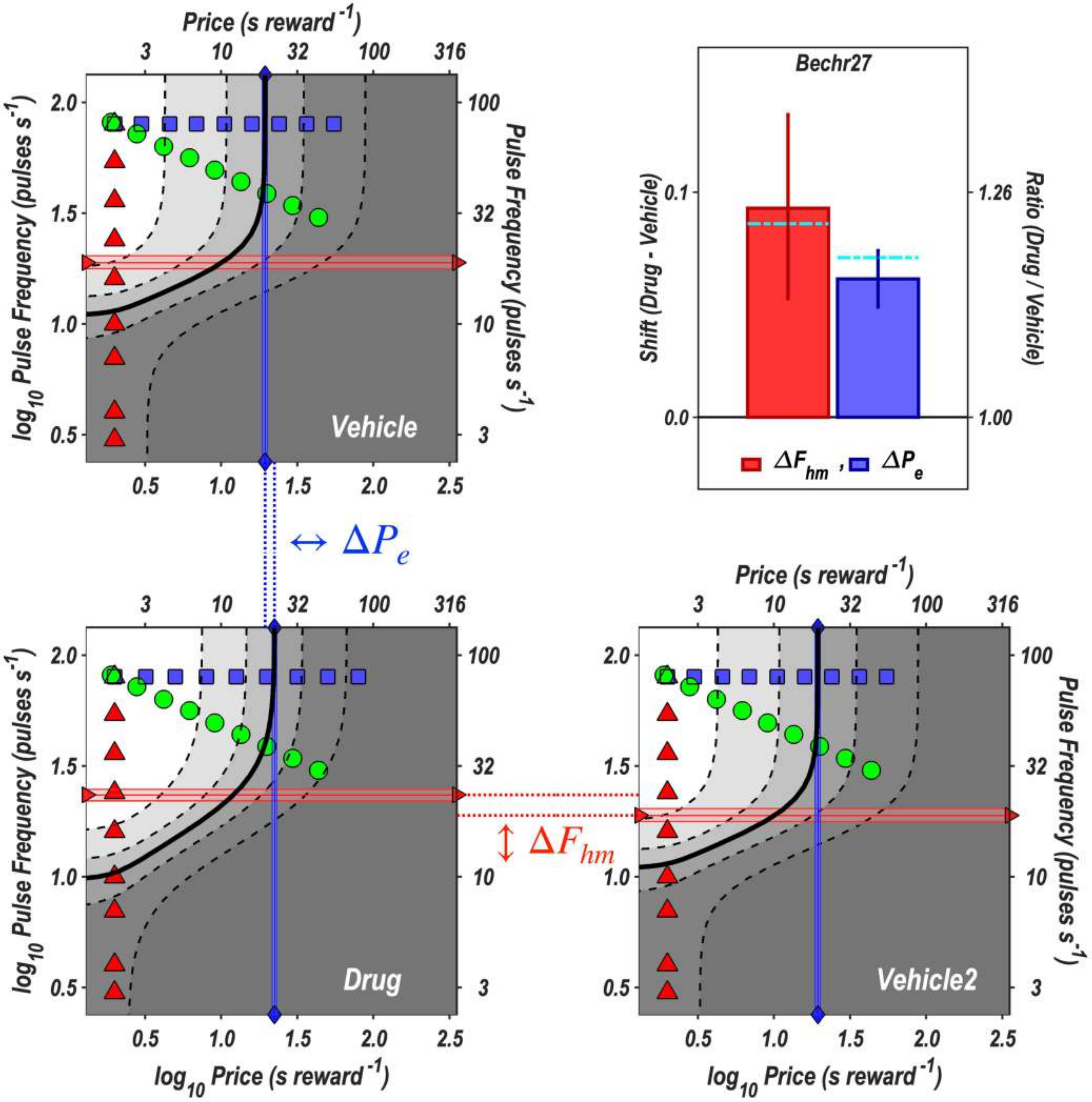

Price (s reward -1)
3
10
32
100
316
log10 Pulse Frequency (pulses s-1)
Pulse Frequency (pulses s-1)
Vehicle
Drug
Vehicle2
log10 Price (s reward -1)
↔ ΔPe
↕ ΔFhm
Bechr27
Shift (Drug - Vehicle)
Ratio (Drug / Vehicle)
0.1
0.0
1.26
1.00
ΔFhm , ΔPe

Figure S27

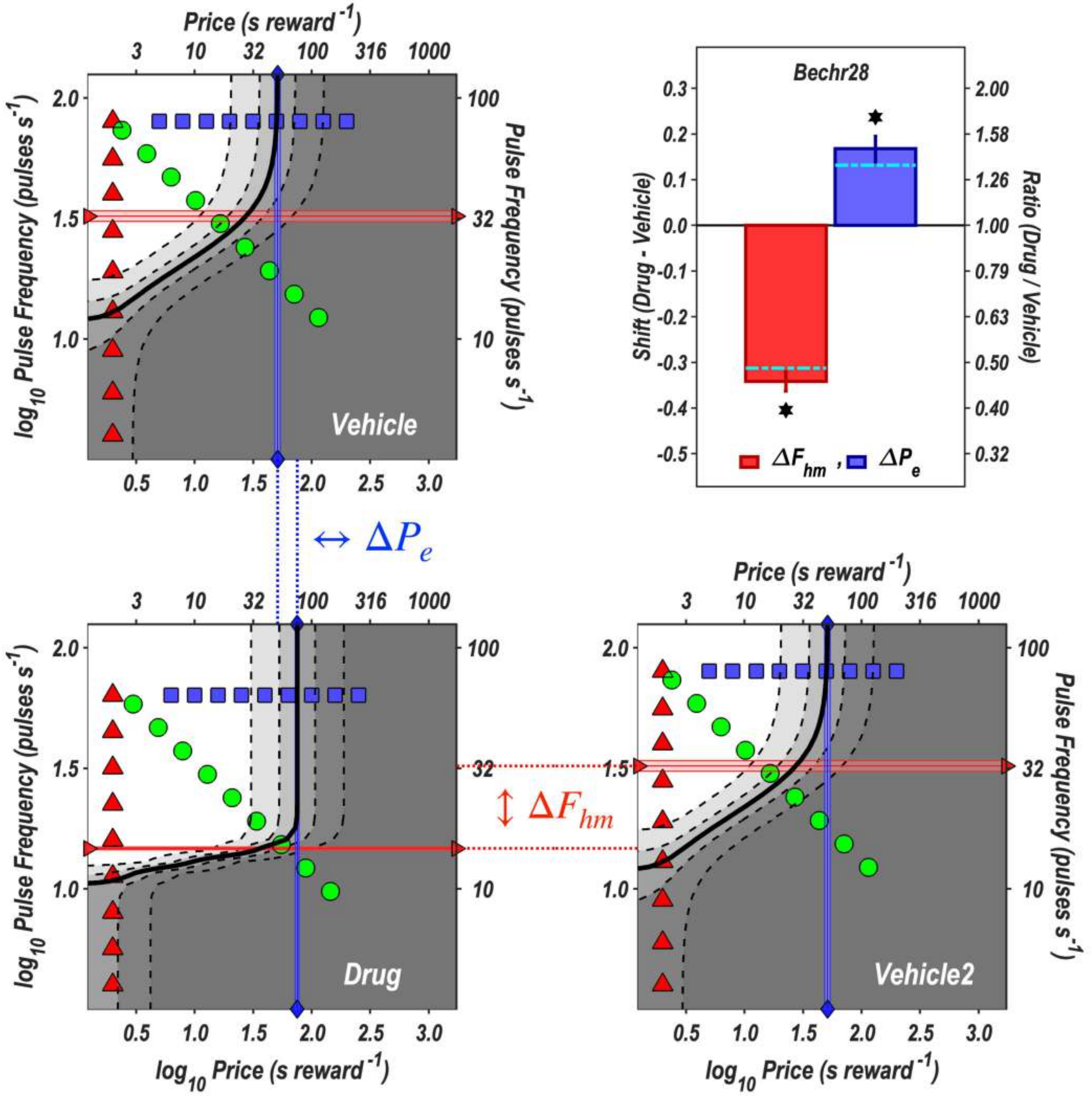

Price (s reward^{-1})
3 10 32 100 316 1000
2.0 1.5 1.0
100 32 10
log$_{10}$ Pulse Frequency (pulses s^{-1})
Pulse Frequency (pulses s^{-1})
Vehicle
0.5 1.0 1.5 2.0 2.5 3.0
Bechr28
0.3 0.2 0.1 0.0 -0.1 -0.2 -0.3 -0.4 -0.5
2.00 1.58 1.26 1.00 0.79 0.63 0.50 0.40 0.32
Shift (Drug - Vehicle)
Ratio (Drug / Vehicle)
ΔF_{hm}, ΔP_e
$\leftrightarrow \Delta P_e$
$\updownarrow \Delta F_{hm}$
Drug
Vehicle2
log$_{10}$ Price (s reward^{-1})

Figure S28

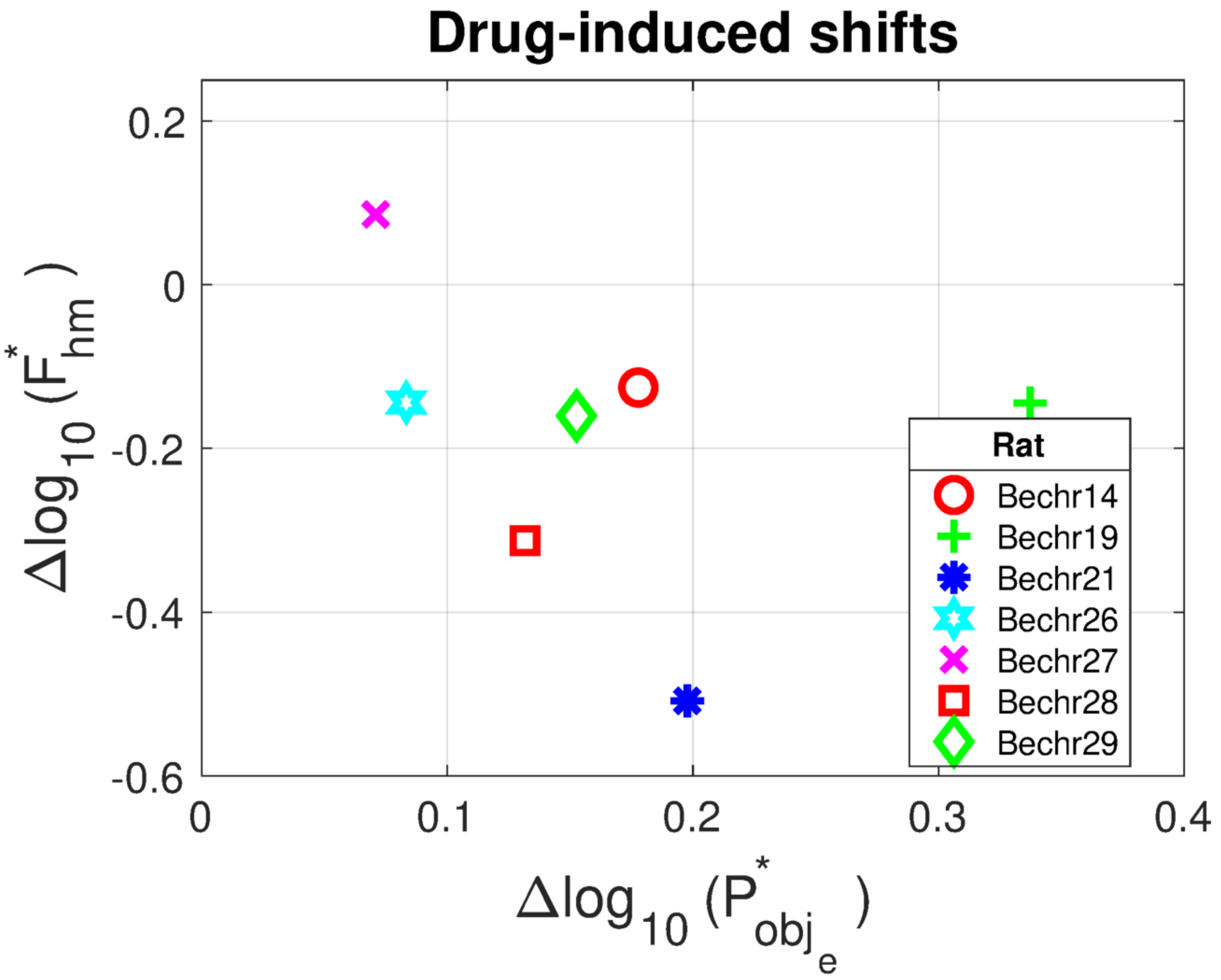

Drug-induced shifts
0.2
0
-0.2
-0.4
-0.6
0
0.1
0.2
0.3
0.4
$\Delta\log_{10}(F^*_{hm})$
$\Delta\log_{10}(P^*_{obj_e})$
Rat
Bechr14
Bechr19
Bechr21
Bechr26
Bechr27
Bechr28
Bechr29

Figure S29

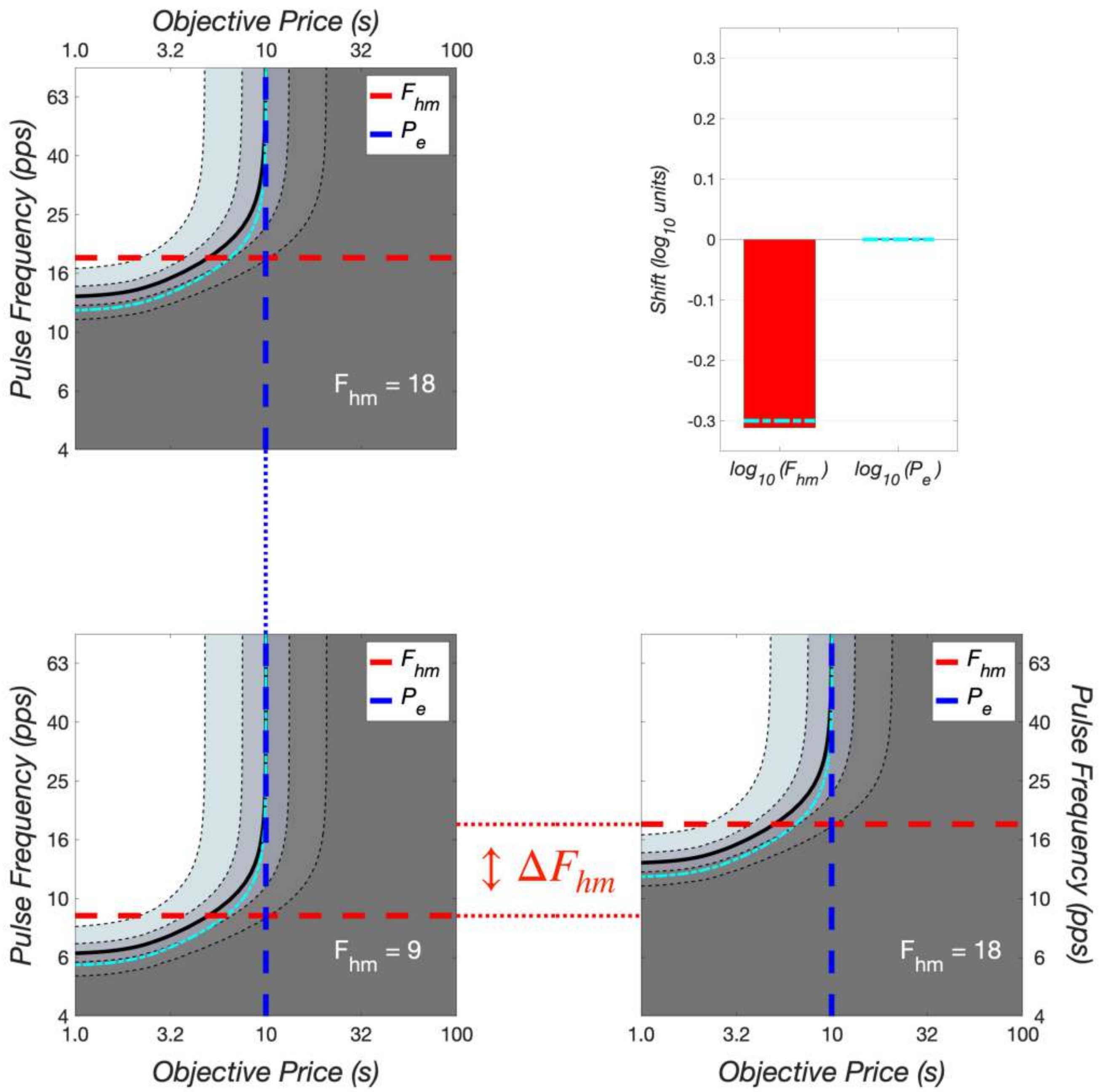
Objective Price (s)
Pulse Frequency (pps)
F_hm
P_e
F_hm = 18
Shift (log10 units)
log10(F_hm)
log10(P_e)
ΔF_hm
F_hm = 9
F_hm = 18

Figure S30

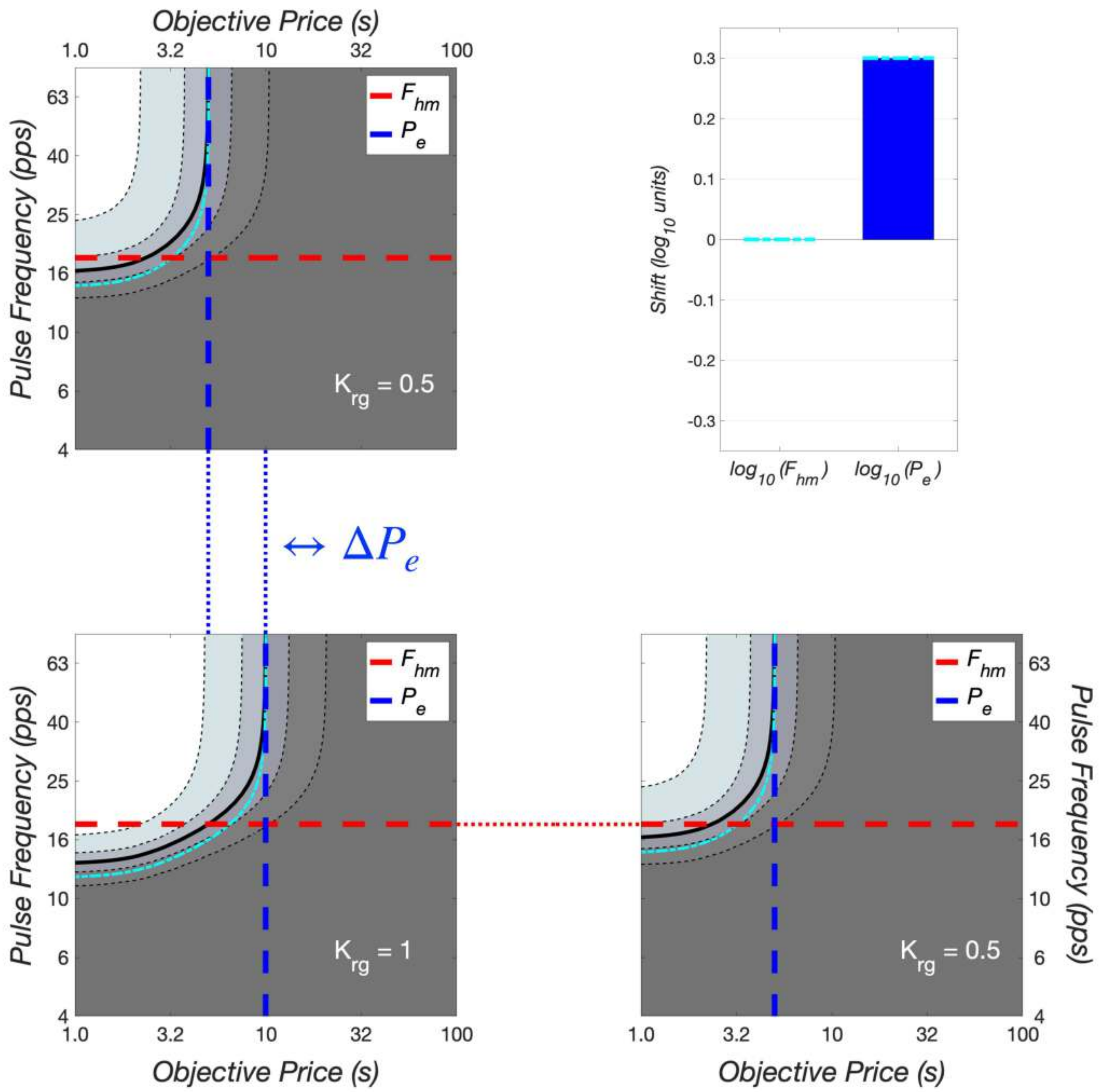
Objective Price (s)
Pulse Frequency (pps)
F_{hm}
P_e
K_{rg} = 0.5
K_{rg} = 1
$\leftrightarrow \Delta P_e$
Shift (log_{10} units)
$log_{10}(F_{hm})$
$log_{10}(P_e)$

Figure S31

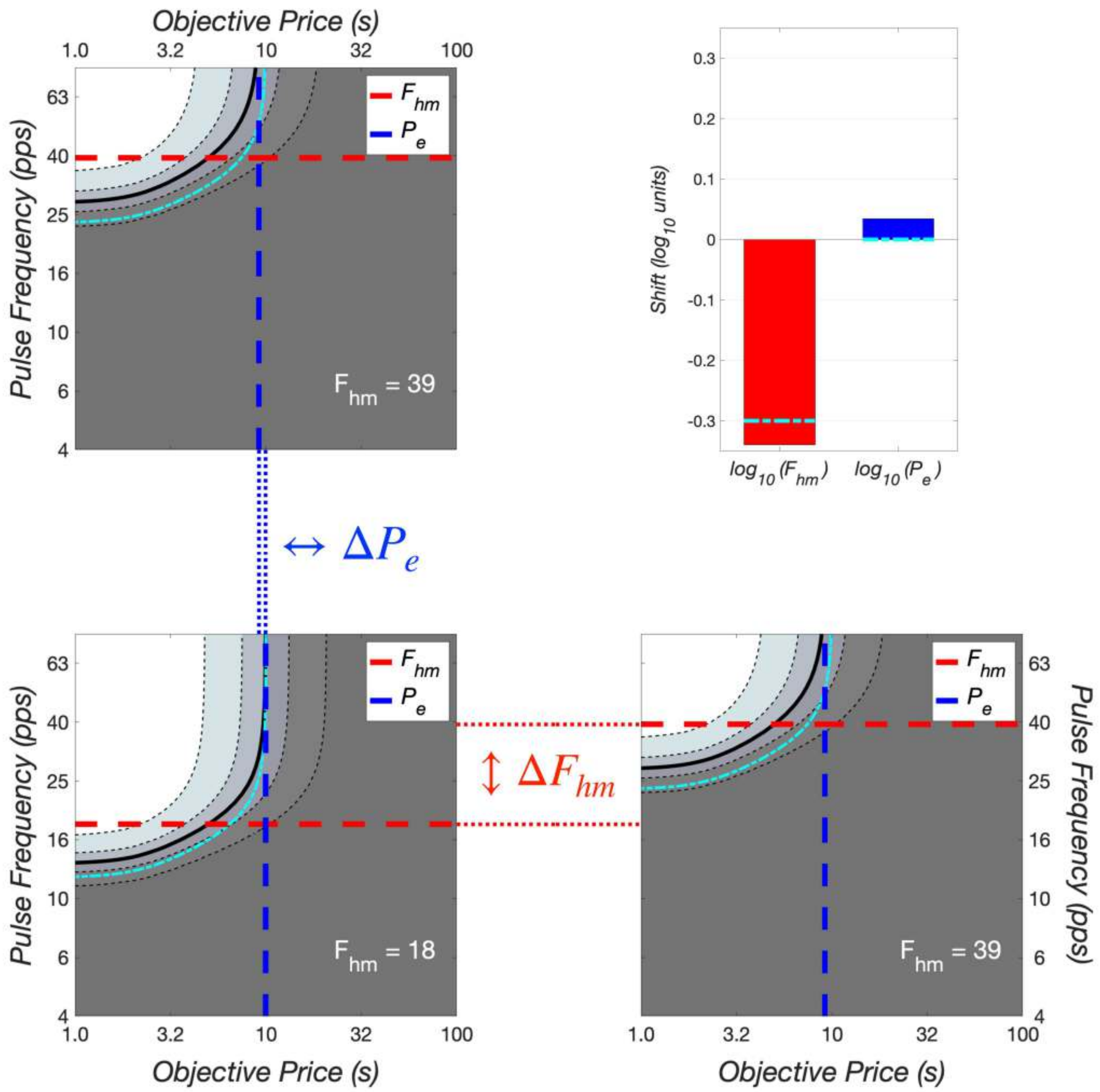

Objective Price (s)
1.0
3.2
10
32
100
Pulse Frequency (pps)
63
40
25
16
10
6
4
F_{hm}
P_e
F_{hm} = 39
Shift (log_{10} units)
0.3
0.2
0.1
0
-0.1
-0.2
-0.3
$log_{10}(F_{hm})$
$log_{10}(P_e)$
$\leftrightarrow \Delta P_e$
$\updownarrow \Delta F_{hm}$
F_{hm} = 18
F_{hm} = 39

Figure S32

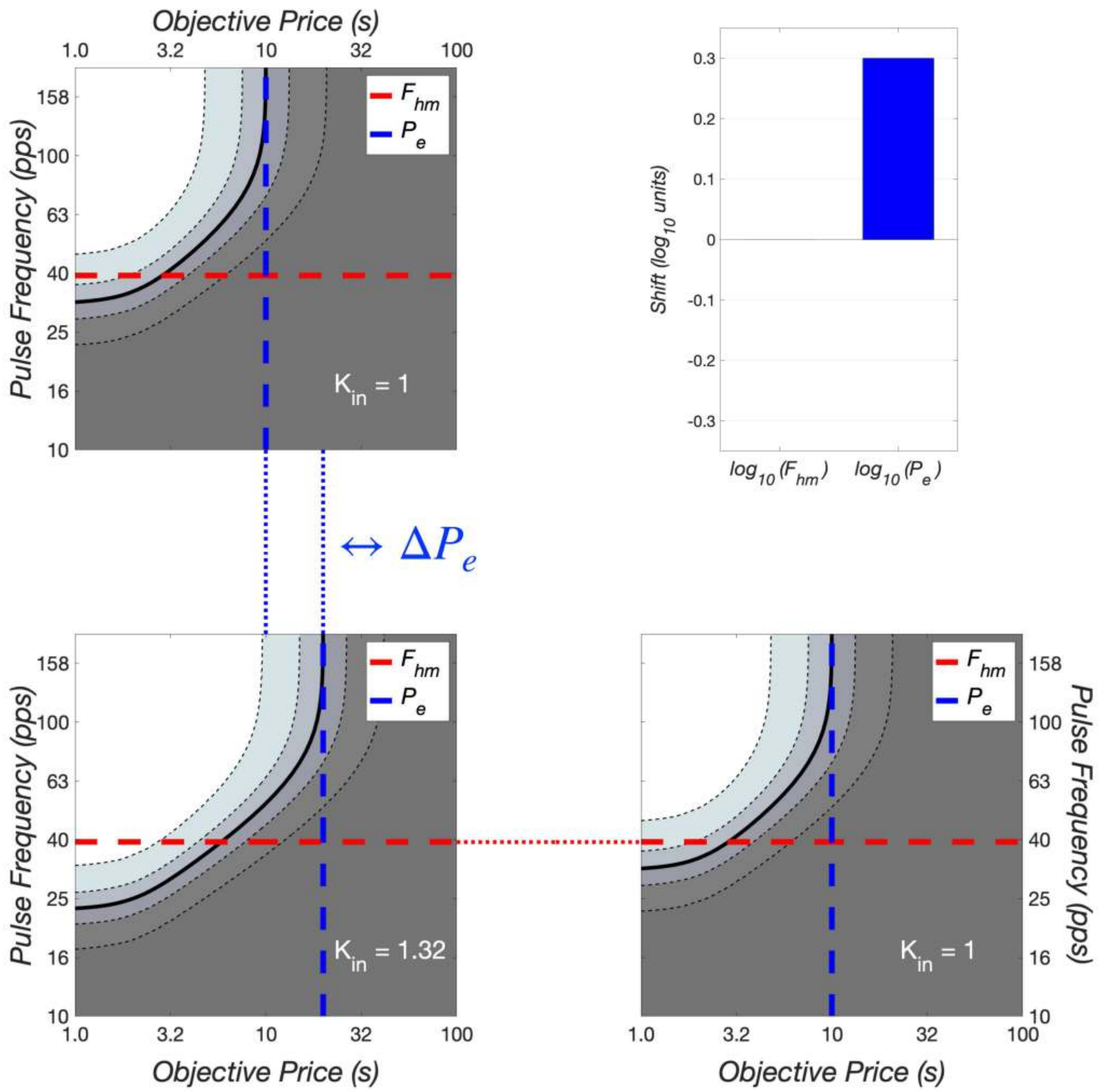
Objective Price (s)
1.0
3.2
10
32
100
Pulse Frequency (pps)
158
100
63
40
25
16
10
F_{hm}
P_e
K_{in} = 1
Shift (log_{10} units)
0.3
0.2
0.1
0
-0.1
-0.2
-0.3
$log_{10}(F_{hm})$
$log_{10}(P_e)$
$\leftrightarrow \Delta P_e$
K_{in} = 1.32
K_{in} = 1

Figure S33

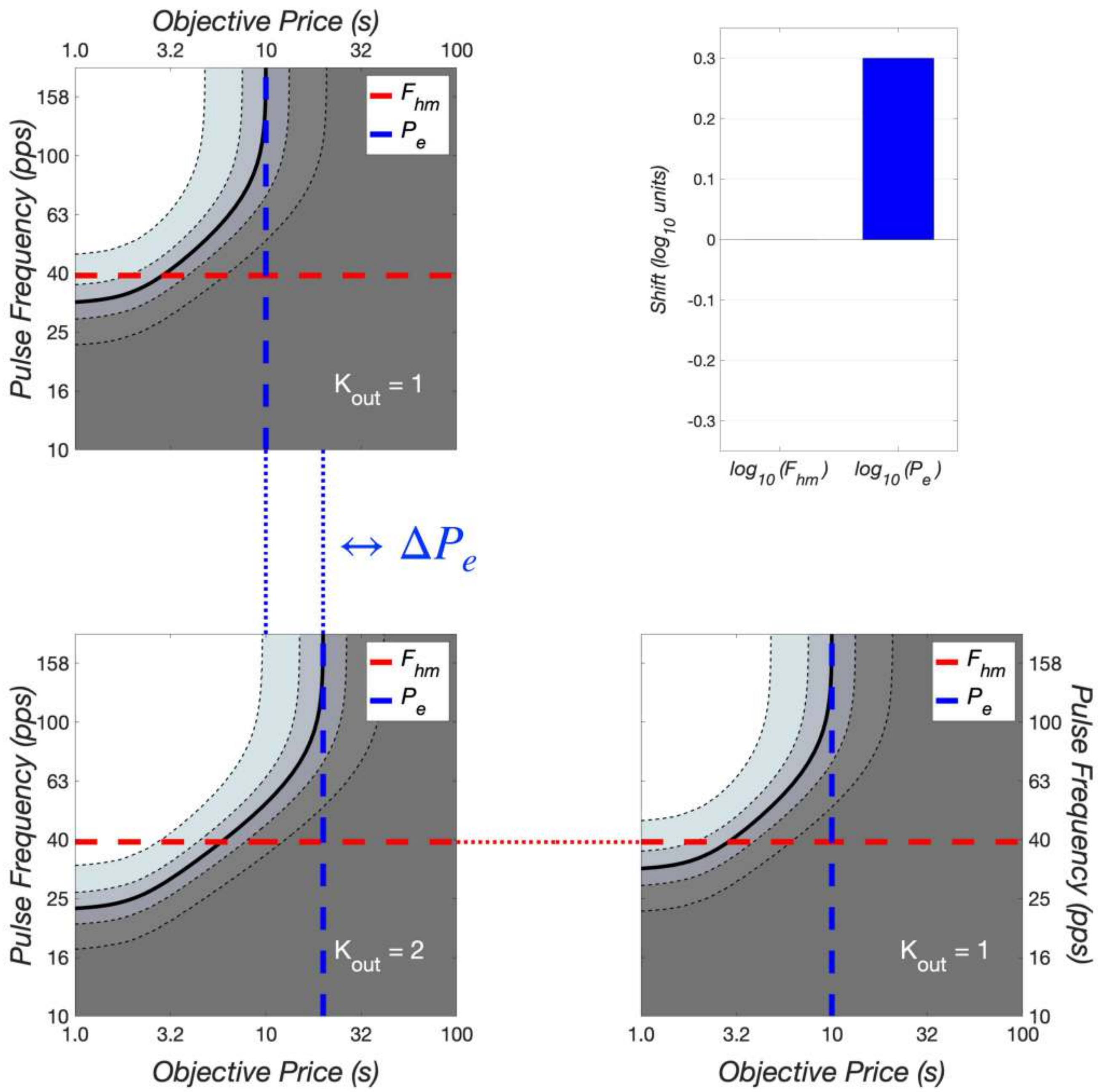
Objective Price (s)
1.0
3.2
10
32
100
Pulse Frequency (pps)
158
100
63
40
25
16
10
F_{hm}
P_e
K_{out} = 1
$\leftrightarrow \Delta P_e$
K_{out} = 2
K_{out} = 1
Shift (log_{10} units)
0.3
0.2
0.1
0
-0.1
-0.2
-0.3
$log_{10}(F_{hm})$
$log_{10}(P_e)$

Figure S34

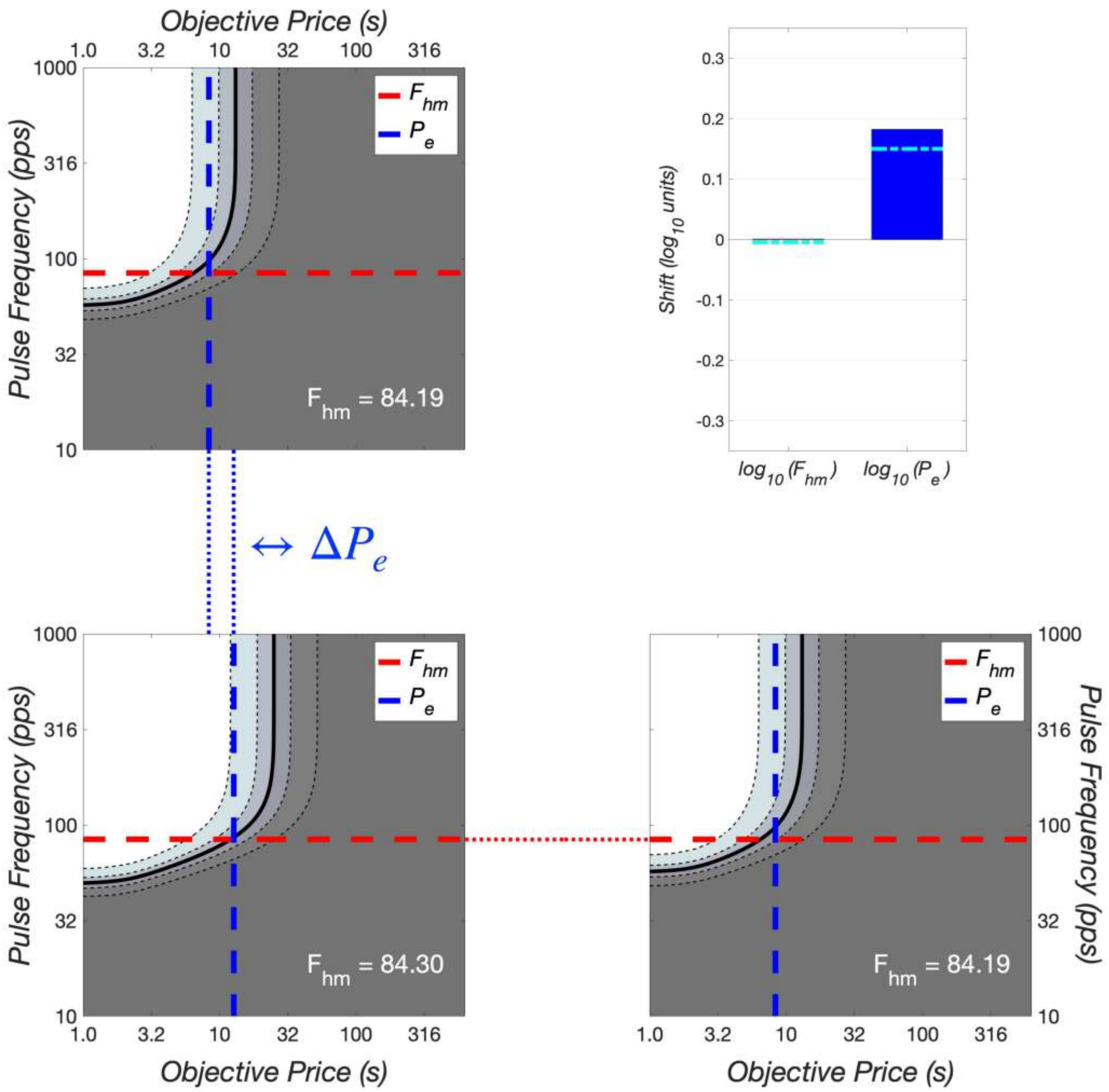

Objective Price (s)
1.0
3.2
10
32
100
316
1000
316
100
32
10
Pulse Frequency (pps)
F_{hm}
P_e
F_{hm} = 84.19
0.3
0.2
0.1
0
-0.1
-0.2
-0.3
Shift (log_{10} units)
$log_{10}(F_{hm})$
$log_{10}(P_e)$
$\leftrightarrow \Delta P_e$
F_{hm} = 84.30
F_{hm} = 84.19

Figure S35

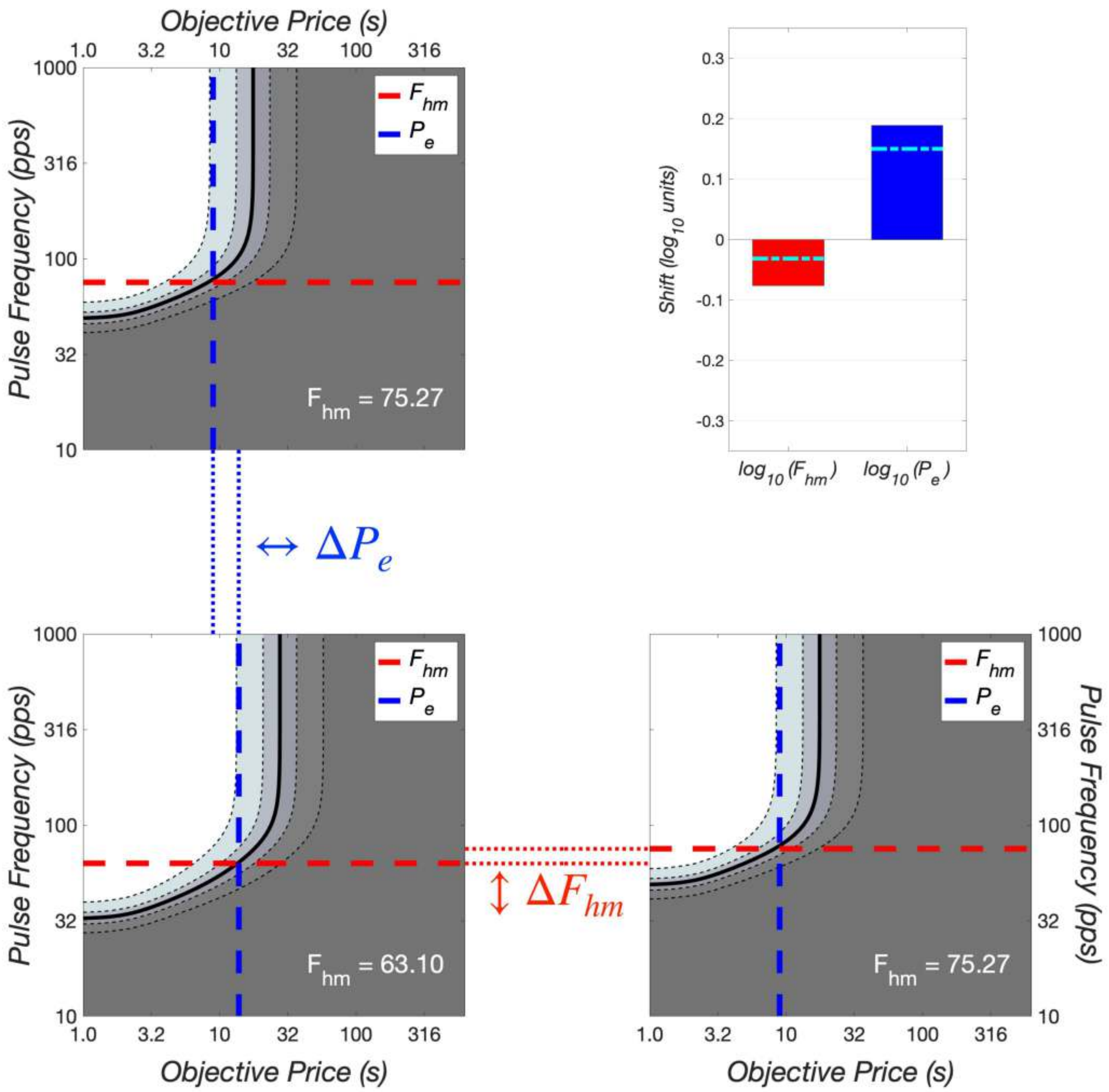
Objective Price (s)
1.0
3.2
10
32
100
316
1000
316
100
32
10
Pulse Frequency (pps)
F_{hm}
P_e
F_{hm} = 75.27
0.3
0.2
0.1
0
-0.1
-0.2
-0.3
Shift (log_{10} units)
$log_{10}(F_{hm})$
$log_{10}(P_e)$
$\leftrightarrow \Delta P_e$
F_{hm} = 63.10
$\updownarrow \Delta F_{hm}$
F_{hm} = 75.27

Figure S36

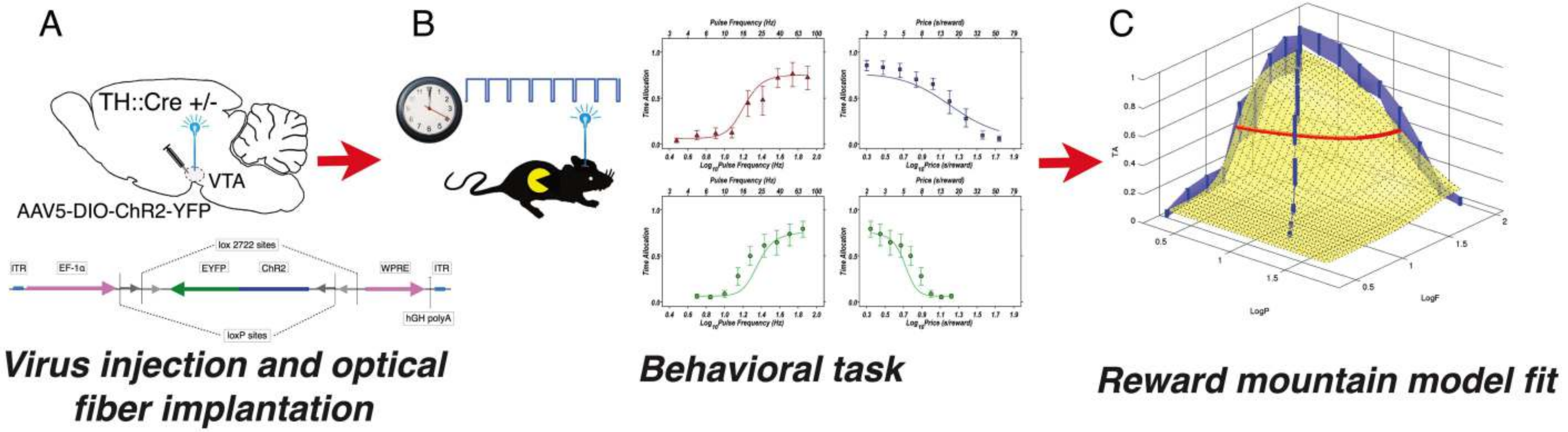
A
TH::Cre +/-
VTA
AAV5-DIO-ChR2-YFP
lox 2722 sites
ITR
EF-1α
EYFP
ChR2
WPRE
ITR
hGH polyA
loxP sites
Virus injection and optical fiber implantation
B
Pulse Frequency (Hz)
Price (s/reward)
Time Allocation
Behavioral task
C
TA
LogP
LogF
Reward mountain model fit

Figure S37

Instructions for the Matlab Live Script that accompanies the following paper:

Trujillo-Pisanty, I., Conover, K., Solis, P., Palacios, D., & Shizgal, P. Dopamine neurons do not constitute an obligatory stage in the final common path for the evaluation and pursuit of brain stimulation reward. PLOS ONE, 2020, in press.

Peter Shizgal
peter.shizgal@concordia.ca

The Matlab Live Script used in the simulations is available from the following GitHub repository:

https://github.com/ShizgalP/A-new-model-of-brain-reward-circuitry

This repository includes three files associated with the simulations:

- The Live Script: GBR_eICSS_oICSS_v10.mlx
- A non-executable HTML version of the Live Script that can be viewed in a browser.
- A .zip archive of the 17 graphics files that the Live Script will import: Imported_figures.zip

The setup of the Live Script is described in the section entitled "Preliminaries" and is implemented in lines 4-24.

The Live Script should run on any version of Matlab from R2018a onwards. The script hasn't been tested on earlier versions, but it may run on versions as far back as R2016a.

Execution of the entire script can take several minutes on a reasonably fast system. It may prove most practical to work through it in sections via the "Run Section" and "Run and Advance" buttons of the Live-Script editor.

Most variables are cleared at the end of each major section. The ones that are required throughout are all defined before line 316 ("Moving the mountain: validation studies"). Once the script has run to that point, the user can navigate directly to sections of interest via the "Go To" button, e.g.

- line 599: "The significance of orthogonal shifts"
- line 870: "At what stage of processing does perturbation of dopaminergic neurotransmission alter reward seeking in eICSS?"
- line 878: "Optical intracranial self-stimulation of midbrain dopamine neurons"
- line 1030: "Dopaminergic modulation of subjective effort cost"
- line 1122: "The series-circuit model of eICSS and oICSS"
- line 1406: "The convergence model"

MATLAB Live Script to supplement "The effect of dopamine transporter blockade on optical self-stimulation: behavioral and computational evidence for parallel processing in brain reward circuitry"

This document was not intended to stand alone; It is assumed that anyone working through this script has already read the manuscript, with particular attention to the derivation of the reward-mountain model in the supporting-information file.

Preamble

In the current experiment, rats worked for optical stimulation of midbrain dopamine neurons. Time allocated to reward seeking was measured as a function of the strength and cost of the stimulation. We call the resulting three-dimensional data structure the "reward mountain." Dopamine neurotransmission was perturbed by administration of a dopamine-transporter blocker, and the consequent displacement of the reward mountain was determined.

The reward-mountain model was developed to account for data from experiments on rats working for rewarding electrical brain stimulation (electrical intracranial self-stimulation: eICSS). This model incorporates information obtained over many decades of research on the neural circuitry that intervenes between the tip of the stimulating electrode and the behavioral effects of the stimulation. Although this work has been dogged by uncertainty about the identity of the directly stimulated neurons, painstaking psychophysical experiments have provided extensive information about the directly activated neurons responsible for the rewarding effect, the operating principles of the neural circuitry in which they are embedded, and the reward-seeking behavior generated by this circuitry. Of particular importance to the current study are experiments characterizing the spatiotemporal integration of the electrically evoked reward signals and the growth of the rewarding effect as a function of stimulation strength.

In contrast to the case of eICSS, the identity of the directly stimulated substrate responsible for optical intracranial self-stimulation (oICSS) of midbrain dopamine neurons is known - the stimulation specifically and directly activates the dopaminergic neurons. However, only rudimentary information is available to date about the growth or spatiotemporal integration of the optically induced reward signal. One purpose of this document is to explore what the current experiment reveals about spatiotemporal integration and reward growth in the neural circuitry underlying oICSS. Another purpose is to explore the implications of the current work for understanding how eICSS and oICSS are related at the level of neural circuitry. We show by means of simulation that the results of the current experiment pose grave difficulties for an intuitively appealing account we and others proposed previously: that eICSS arises from the indirect activation of the directly stimulated dopamine neurons that give rise to oICSS. These difficulties challenge widely held notions about the organization of brain circuitry underlying reward.

Preliminaries

Setting the ***graph2files*** variable to true will cause graphs to be written to external files. These files are stored in a subdirectory of the current folder called 'Figures.' Setting this variable to false will speed execution.

The ***show_graphics*** variable determines whether graphics are stored within this Live Script. This variable must be set to false to enable efficient editing of this Live Script. When this Live Script has been executed

with ***show_graphics*** set to true, subsequent editing and task switching will be slowed unacceptably. Set ***show_graphics*** to true only in preparation for saving updated html and/or pdf copies of this file. To prepare for editing after this script has been stored with ***show_graphics*** set to true, reset this variable to false, re-run the script, and save it.

Setting the ***tabs2files*** variable to true will store information about equations, figures, functions, and symbols in external Excel files. Setting this variable to false may speed execution slightly.

Setting the ***saveWS*** variable to true will cause the Matlab workspace to be stored in a .mat file in the folder from which this live script was run.

Be sure to set the default_dir to the directory you wish to use as the default on your system.

As its name implies, the '***Imported_Figures***' folder contains images that this Live Script will import from external files. Those images should be stored in a sub-folder of the folder from which this Live Script will be run. The name of that folder is the argument of the ***set_impfigdir*** command below.

Blocks of redundant code are included in various sections. Although this lengthens the Live Script, it reduces the memory load due to the accumulation of workspace variables. Given that most variables are cleared at the boundaries between major sections, the blocks of redundant code enable debugging by means of "Run Section," "Run and Advance," etc. In a future revision, efficiency may be increased by aggregating the variables assigned in these blocks in structures that are saved and re-loaded.

```
t_start = tic; % start timer for entire script
tic; % start timer for the current section
global graphs2files show_graphics
graphs2files = true; % save graphics to external files?
tabs2files = true; % save tables of equation #s, figure #s, function #s, and symbol #s?
saveWS = true; % save final workspace?
show_graphics = true; % display graphics in this Live Script?
version = 10; % used in the names of stored files

% define and set the default directory
default_dir = '~/Work/Research/papers/In_Progress/Opto_GBR2/Simulations';
if ~exist(default_dir, 'dir')
    disp(strcat({'Default directory '}, default_dir, {' does not exist.'}));
    return
end
cd(default_dir);

% Define the directory that will contain figures generated here & create it if necessar
global FigDir
FigDir = set_figdir('Figures', graphs2files); % Sub-directory of default directory
% Define the directory containing stored images to be imported.
ImpFigDir = set_impfigdir('Imported_figures'); % Sub-directory of default directory
% Define the directory that will house the tables & create if if necessary.
tabdir = set_tabdir('Tables', tabs2files); % Sub-directory of default directory
```

The following technical section implements preliminary steps required to set up the simulations.

In order to set up the simulations, some code must be executed to define basic functions and initialize variables.

This document was designed to run on any version of Matlab from R2018a onwards without requiring installation of external function files or scripts. All functions are either built-in (supplied by the Mathworks as part of the standard Matlab installation) or local (i.e., defined at the end of this Live-Script document). This should allow this script to execute on any standard installation of the supported Matlab versions.

The following code initializes the tables that store information about the formatted equations, figures and symbols used in this script.

```
%% Initialization
```

Initialize the tools that build the tables of equations, figures, functions, and symbols. Load the numbers and descriptions of the pre-defined functions (see below, "***Tools for this live script***" and "***Building blocks for the functions included in the simulations***."

```
global eqn_num eqn_tab fig_num fig_tab fun_num fun_tab sym_num sym_tab
init_all;
```

```
The equation table has been initialized.
The figure table has been initialized.
The function table has been initialized.
The symbol table has been initialized.
This is function #1: (init_all)
This is function #2: (add_eqn)
This is function #3: (add_fig)
This is function #4: (add_fun)
This is function #5: (add_sym)
This is function #6: (FilterFun)
This is function #7: (FilterFunBS)
This is function #8: (LogistNormFun)
This is function #9: (LogistNormBsFun)
This is function #10: (LogistNormBsLocFun)
This is function #11: (PsubFun)
This is function #12: (PsubBsFun)
This is function #13: (ScalarDivFun)
This is function #14: (ScalarDivBsFun)
This is function #15: (ScalarMultFun)
This is function #16: (ScalarMultBsFun)
This is function #17: (PsubEfun)
This is function #18: (PobjEfun)
```

Load the numbers and descriptions of the pre-defined functions (see below, "***Tools for this live script***" and "***Building blocks for the functions included in the simulations***."

Enter symbols defined in the introductory paragraphs above.

```
[sym_num, sym_tab] = add_sym(sym_num, sym_tab, "eICSS", "electrical intracranial self-s
[sym_num, sym_tab] = add_sym(sym_num, sym_tab, "oICSS", "optical intracranial self-stim
keepVars = who; % Store variables to be retained
toc
```

```
Elapsed time is 0.110595 seconds.
```

The mountain model

```
tic;
```

The reward-mountain model was developed to account for operant performance as a function of the cost and strength of an experimenter-controlled reward, within the context of eICSS studies. The task performed to obtain the rewarding stimulation entails putting time on a clock by holding down a lever; a reward is delivered when the cumulative time the lever has been depressed reaches an experimenter-defined criterion (the "***price***" of the reward). The measure of operant performance employed is time allocation: the proportion of trial time that the rat devotes to procurement of the rewarding stimulation ("work"). The model is derived in the main body of the accompanying manuscript and then adapted for application to oICSS. Papers cited here are listed in the reference section of the main body of the manuscript.

In this section, we define the functional building blocks that will be used to simulate the output of the reward-mountain model.

We distinguish between the "shell" and "core" of the model. The shell consists of the variables that are observed (time allocation), manipulated (pulse frequency, price), and controlled (stimulation parameters held constant, physical work required to hold down the lever, affordances of the test environment for leisure activites, such as grooming, resting, and exploring). The core consists of the functions that compute the intensity of the reward produced by the stimulation train and combine this value with the opportunity and effort costs entailed in its procurement, thus generating what we call "payoffs." A single function based on the generalized matching law translates the payoffs generated in the core into the time-allocation values that are manifest in the shell.

Please see the main body of the text for definitions, explanations, and details.

Shell→core functions

The frequency-following function

Solomon et al. (2015) studied frequency-following fidelity in rats working for rewarding electrical stimulation of the medial forebrain bundle (MFB). They showed that the following function provides a good description of the relationship between the induced frequency of firing in the directly stimulated neurons and the pulse frequency:

$$F_{firing} = f_F(F_{pulse}) = K_F \times F_{bend} \times \left(\ln\left[1 + e^{\frac{F_{ro}}{F_{bend}}}\right] - \ln\left[1 + e^{\frac{F_{ro}-F_{pulse}}{F_{bend}}}\right]\right)$$

where

$f_F =$ the frequency-following function

$F_{bend} =$ parameter determining the abruptness of the roll-off in the frequency response; units: *unitless*

$F_{firing} =$ induced firing rate in the first-stage neurons; units: $firings\ \ s^{-1}\ \ neuron^{-1}$

$F_{pulse} =$ the pulse frequency; units: $pulses\ \ s^{-1}$

$F_{ro} =$ the pulse frequency in the center of the roll-off region; units: $pulses\ \ s^{-1}$

$K_F =$ unit-translation constant; \units: $firings\ \ pulse^{-1}\ \ neuron^{-1}$

```
[eqn_num, eqn_tab] = ...
    add_eqn(eqn_num, eqn_tab, "FreqFolElec", "Frequency of firing as a function of elec
```

```
This is equation #1: (FreqFolElec)
```

```
[sym_num, sym_tab] = add_sym(sym_num, sym_tab, "fF", "frequency-following function");
[sym_num, sym_tab] = add_sym(sym_num, sym_tab, "Fpulse", "pulse frequency");
[sym_num, sym_tab] = add_sym(sym_num, sym_tab, "Fbend", "parameter determining sharpnes
```

```
[sym_num, sym_tab] = add_sym(sym_num, sym_tab, "Fro", "the pulse frequency in the cente
[sym_num, sym_tab] = add_sym(sym_num, sym_tab, "Ffiring", "induced firing rate of the c
[sym_num, sym_tab] = add_sym(sym_num, sym_tab, "Kf", "unit-translation constant for the
[fun_num, fun_tab] = add_fun(fun_num, fun_tab, "FilterFun", "F, Fbend, Fro",...
    "Frequency-following function");
```

```
Function FilterFun has already been entered.
```

The following graph relates the induced firing frequency to the electrical pulse frequency:

```
logFelecBend = 1.3222; % from Solomon et al., 2015
FelecBend = 10^logFelecBend;
logFelecRO = 2.5587; % from Solomon et al., 2015
FelecRO = 10^logFelecRO;
Felec = logspace(0,3,121);
Fmfb = FilterFun(Felec, FelecBend, FelecRO); % Compute firing rate using the frequency

FF_graph = plot_freqFoll(Felec,Fmfb,'mfb',2,1000,2,1000); % see "Functions that plot gr
if show_graphics
    FF_graph.Visible = 'on';
end
```

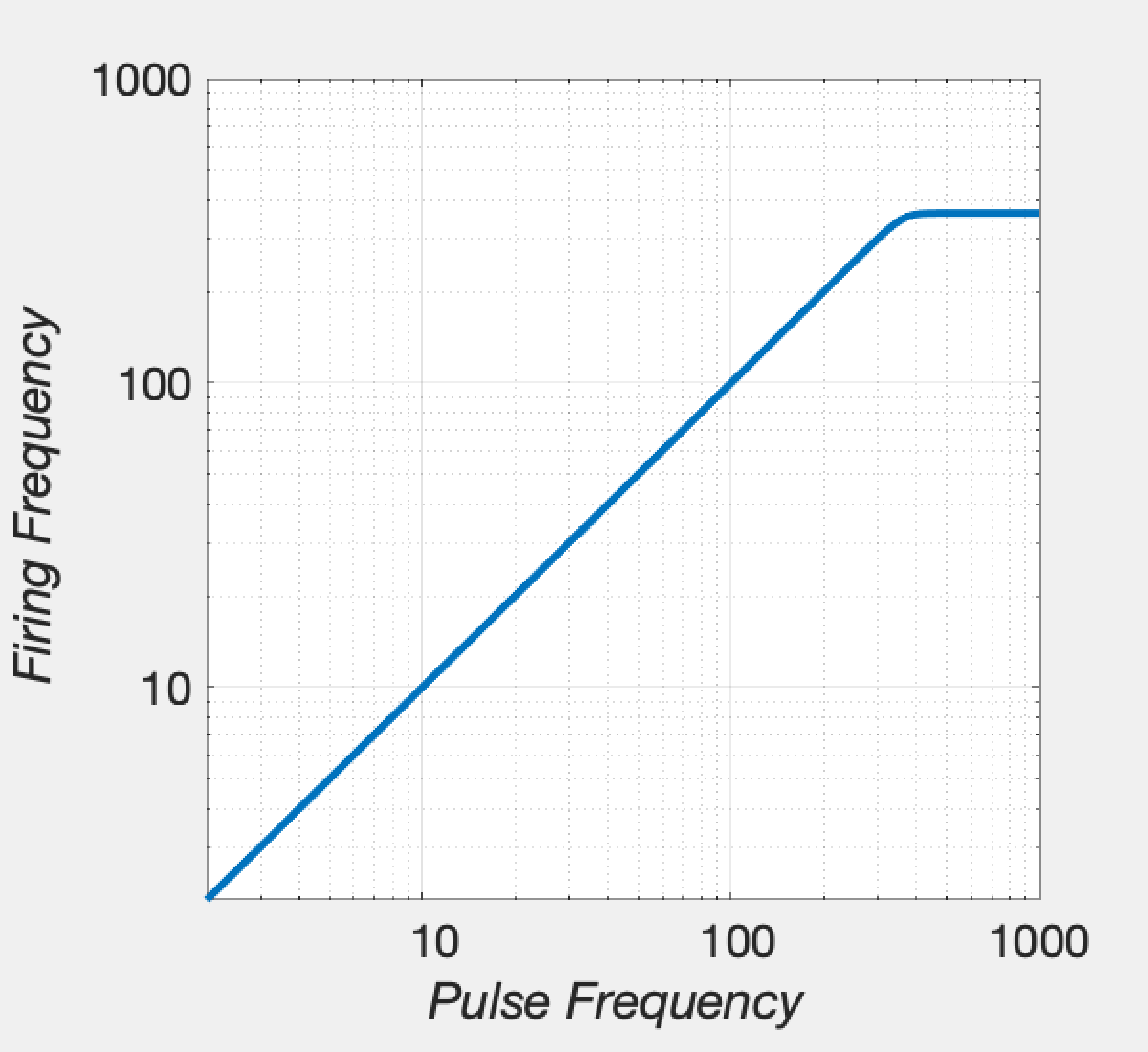

```
[fig_num, fig_tab] = add_fig(fig_num, fig_tab, "FreqFolMFB", ...
    "Induced firing frequency in the directly stimulated neurons as a function of pulse
```

```
This is figure #1: (FreqFolMFB)
```

This graph shows that the firing of the directly stimulated neurons subserving eICSS of the medial forebrain bundle can follow the pulse frequency faithfully up to very high values (>= 350 pulses per second); the response of the neurons flattens abruptly as pulse frequency is increased further. This high-fidelity frequency following is consistent with the view that the directly stimulated cells subserving the rewarding effect are non-dopaminergic neurons with myelinated axons (Shizgal, 1997; Bielajew & Shizgal, 1986; Gallistel, Yeomans & Shizgal, 1981; Bielajew & Shizgal, 1982; Shizgal et al., 1980).

In addition to finding firing frequencies corresponding to a given pulse frequency, we will also need to do the reverse: finding the pulse frequency required to produce a given firing frequency. Thus, we define a back-solution of the frequency-following function:

$$F_{pulse} = F_{ro} - F_{bend} \times \ln\left[\left(e^{\frac{F_{firing}}{F_{bend}}}\right) \times \left(e^{\frac{F_{ro}}{F_{bend}}} + 1\right) - 1\right]$$
$$\text{for } 0 \leq F_{firing} \leq F_{firing_{max}}$$

```
[eqn_num, eqn_tab] = add_eqn(eqn_num, eqn_tab, "fFbacksolved", "Back-solution of the fr
```

```
This is equation #2: (fFbacksolved)
```

The neural-recruitment function

The f_N function translates the pulse duration and current into the number of electrically excited neurons:

$$N = f_N(d, I) = \frac{K_{N_S}}{K_{I_S}} \times \frac{I}{\rho_I \times \left(1 + \frac{c}{d}\right)}$$

where

c = chronxie; pulse duration (units: ms) for which the threshold current is twice ρ_I

d = pulse duration (units: ms)

f_N = the neural (first-stage) recruitment function

I = current (units: μA)

K_{N_S} = neuron-distance constant in units of neurons mm^{-2}

K_{I_S} = current-distance constant in units of μA mm^{-2}

N = number of activated first-stage neurons; units: *neurons*

ρ_I = threshold current required to excite a first-stage neuron using a pulse of infinite duration; units: μA

```
[eqn_num, eqn_tab] = add_eqn(eqn_num, eqn_tab, "fN", "Neural-recruitment function");
```

```
This is equation #3: (fN)
```

```
[sym_num, sym_tab] = add_sym(sym_num, sym_tab, "c", "chronaxie of strength-duration fun
[sym_num, sym_tab] = add_sym(sym_num, sym_tab, "d", "pulse duration");
[sym_num, sym_tab] = add_sym(sym_num, sym_tab, "fN", "Neural-recruitment function");
[sym_num, sym_tab] = add_sym(sym_num, sym_tab, "I", "current");
```

```
[sym_num, sym_tab] = add_sym(sym_num, sym_tab, "Kns", "Neuron-recruitment function");
[sym_num, sym_tab] = add_sym(sym_num, sym_tab, "Kis", "Current-distance constant");
[sym_num, sym_tab] = add_sym(sym_num, sym_tab, "N", "Number of activated first-stage ne
[sym_num, sym_tab] = add_sym(sym_num, sym_tab, "rhoI", "rheobase: threshold current to
```

Although we define this function here, we do not implement it or use it in the simulations. Instead, an output value is chosen that, together with a value for the rheobase of the strength-duration function for trains, generates location-parameter values within the range observed in past studies. This function is included in order to summarize the relationships on which existing tests of the counter model have been based (e.g., Simmons & Gallistel, 1994).

The burst-duration function

For completeness, we include a function that maps the duration of the pulse train into the duration of the evoked increase in the firing of the first-stage neurons. In practice, we assume that the two are equal, as will be case when frequency-following fidelity is high.

$_{burst} = f_D(D_{train})$

here

$_{burst}$ = duration of the stimulation-induced increase in firing above baseline in the directly stimulated neurons subserving the reward effec

$_{train}$ = time interval between the leading edges of the first and last pulse in a stimulation train

= the duration-mapping function

```
[eqn_num, eqn_tab] = add_eqn(eqn_num, eqn_tab, "fD", "Train-burst equation");
```

```
This is equation #4: (fD)
```

```
[sym_num, sym_tab] = add_sym(sym_num, sym_tab, "Dburst", "Duration of stimulation-induc
[sym_num, sym_tab] = add_sym(sym_num, sym_tab, "Dtrain", "train duration");
[fun_num, fun_tab] = add_fun(fun_num, fun_tab, "fD", "Dtrain",...
    "Train-burst function");
```

```
This is function #19: (fD)
```

The subjective-probability function

This is another dummy function. As we review below, the subjective probability that a reward will be delivered upon satisfaction of the response requirement appears to equal the objective probability when the latter is 0.5 or higher. In the case of the present oICSS experiment, the objective probability of reward is always one.

n.b. A lower-case "p" is employed in the symbols.

$p_{sub} = f_p(p_{obj})$

where

p_{obj} = objective probability that reward will be delivered upon satisfaction of the response requirement

p_{sub} = subjective probability that reward will be delivered upon satisfaction of the response requirement

```
[eqn_num, eqn_tab] = add_eqn(eqn_num, eqn_tab, "fp", "Subjective-probability equation")
```

```
This is equation #5: (fp)
```

```
[sym_num, sym_tab] = add_sym(sym_num, sym_tab, "ProbObj", "Objective probability that r
```

```
[sym_num, sym_tab] = add_sym(sym_num, sym_tab, "ProbSub", "Subjective probability that
[fun_num, fun_tab] = add_fun(fun_num, fun_tab, "ProbSubFun", "ProbObj",...
    "Subjective-probability function");
```

```
This is function #20: (ProbSubFun)
```

The subjective-price (opportunity-cost) function

As described in Solomon et al., (2017), the subjective-price function is defined as

$$P_{sub} = f_P(P_{obj}) = P_{sub_{min}} + P_{sub_{bend}} \times \ln\left(1 + e^{\left[\frac{P_{obj} - P_{sub_{min}}}{P_{sub_{bend}}}\right]}\right)$$

where

$f_P =$ the subjective-price function

$P_{obj} =$ the "price" of a stimulation train: the cumulative time the lever must be depressed to trigger reward delivery; units:~s

$P_{sub} =$ the subjective price of a stimulation train; units:~s

$P_{sub_{min}} =$ the minimum subjective price; units:~s

$P_{sub_{bend}} =$ a constant that controls the abruptness of the transition from "blade" to ``handle;" unitless.

```
[eqn_num, eqn_tab] = ...
    add_eqn(eqn_num, eqn_tab, "SubjPriceFun", "Subjective-price function");
```

```
This is equation #6: (SubjPriceFun)
```

```
[sym_num, sym_tab] = add_sym(sym_num, sym_tab, "Pobj", "objective price (opportunity co
[sym_num, sym_tab] = add_sym(sym_num, sym_tab, "Psub", "subjective price");
[sym_num, sym_tab] = add_sym(sym_num, sym_tab, "PsubBend", "transition parameter of sub
[sym_num, sym_tab] = add_sym(sym_num, sym_tab, "PsubMin", "minimum subjective price");
```

and plotted below.

```
PsubBend = 0.5;
PsubMin = 1.82;
Pobj = logspace(-1,2,120);
Psub = PsubFun(Pobj, PsubBend, PsubMin);
SP_graph = plot_PsubFun(Pobj,Psub, graphs2files, FigDir);
if show_graphics
    SP_graph.Visible = 'on';
end
```

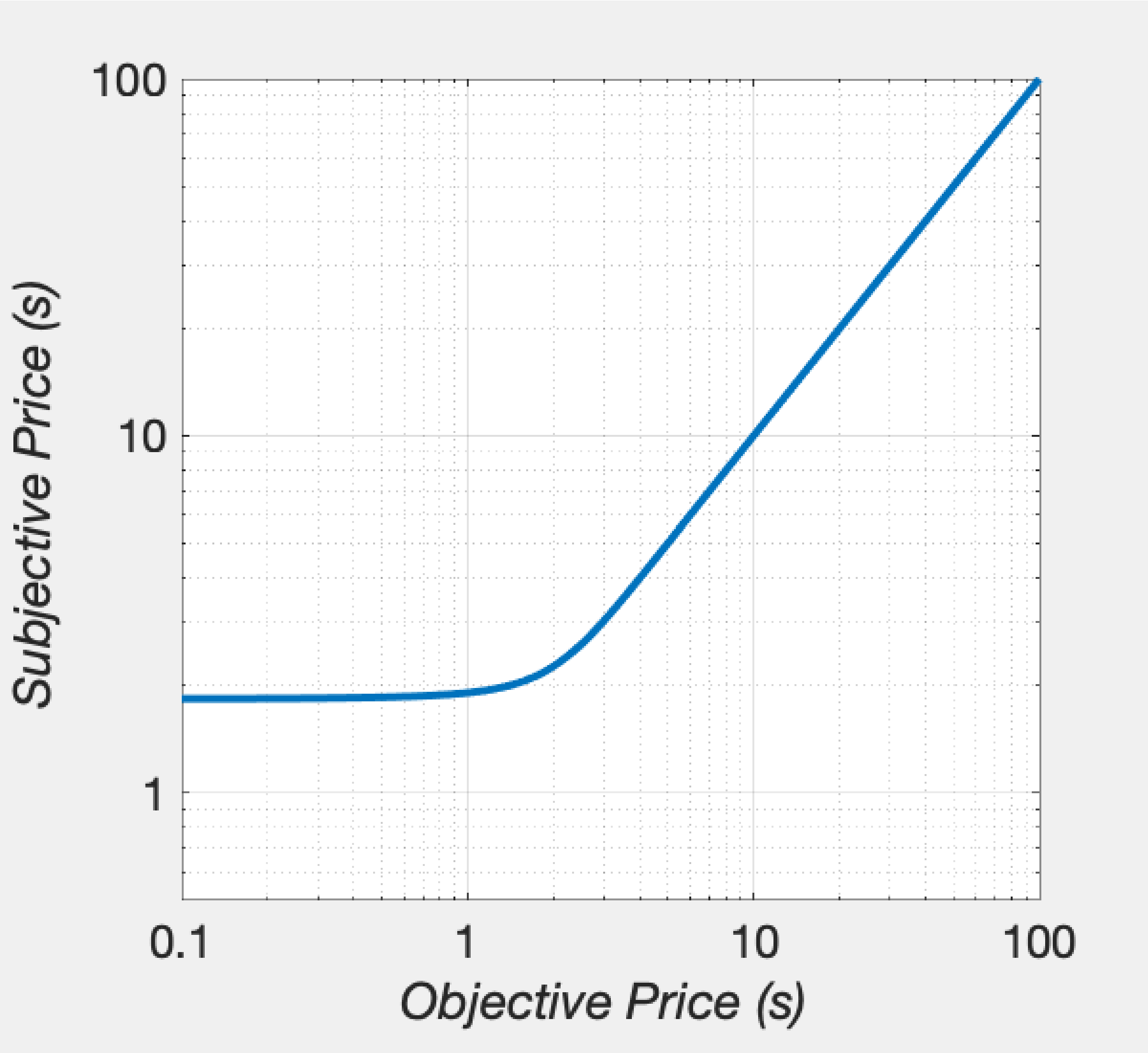


```
[fig_num, fig_tab] = add_fig(fig_num, fig_tab, "PsubFun", ...
    "The subjective-price function");
```

```
This is figure #2: (PsubFun)
```

In addition to finding subjective prices corresponding to objective ones, we will also need to do the reverse. Thus, we define a back-solution of the subjective-price function:

$$P_{obj} = P_{sub_{min}} + P_{sub_{bend}} \times \ln\left[-1 + e^{\left(\frac{P_{sub} - P_{sub_{min}}}{P_{sub_{bend}}}\right)}\right]$$

for $P_{sub} \geq P_{sub_0}$

where

$P_{sub_0} =$ subjective price corresponding to an objective price of zero

```
[eqn_num, eqn_tab] = add_eqn(eqn_num, eqn_tab, "fPbacksolved", "Back-solution of the su
```

```
This is equation #7: (fPbacksolved)
```

The subjective effort-cost function

The physical work entailed in holding down the lever is transformed into the subjective rate of exertion by the following function:

$$\dot{\phi}_{sub_W} = f_\phi\left(\dot{\phi}_{obj_W}, K_\phi\right)$$

f_ϕ = subjective-effort function (form unknown)

$\dot{\phi}_{obj_W}$ = rate of physical work required to hold down the lever; units: $J s^{-1}$

$\dot{\phi}_{sub_W}$ = subjective rate of exertion required to hold down the lever in units we call "*oomphs*" s^{-1}

K_ϕ = unit-conversion constant; units: *oomphs* J^{-1}

We do not attempt to model the form and parameters of this function. The dots over $\dot{\phi}_{obj_W}$ and $\dot{\phi}_{sub_W}$ signify that we define these quantities as rates.

```
[eqn_num, eqn_tab] = add_eqn(eqn_num, eqn_tab, "fphiW", "subjective-effort-cost equatio
```

```
This is equation #8: (fphiW)
```

```
[sym_num, sym_tab] = add_sym(sym_num, sym_tab, "fphi", "subjective effort-cost functior
[sym_num, sym_tab] = add_sym(sym_num, sym_tab, "dotPhiObj", "objective work rate requir
[sym_num, sym_tab] = add_sym(sym_num, sym_tab, "dotPhiSub", "subjective rate of exertio
[sym_num, sym_tab] = add_sym(sym_num, sym_tab, "Kphi", "unit-conversion scalar for the
[fun_num, fun_tab] = add_fun(fun_num, fun_tab, "fphi", "dotPhiObj, Kec",...
    "subjective-effort function");
```

```
This is function #21: (fphi)
```

The effect of the drug, if any, on the rate of subjective exertion is defined as:

$$\dot{\phi}_{sub_{W_{drug}}} = \dot{\phi}_{sub_{W_{vehicle}}} \times K_{ec_W}$$

where

K_{ec_W} = proportional drug-induced change in the subjective rate of exertion required to hold down the lever; unitless

```
[eqn_num, eqn_tab] = add_eqn(eqn_num, eqn_tab, "fphiWdrug", "drug-induced change in the
```

```
This is equation #9: (fphiWdrug)
```

```
[sym_num, sym_tab] = add_sym(sym_num, sym_tab, "KecW", "proportional drug-induced chang
```

In the vehicle condition, K_{ec_W} assumes an implicit value of one.

Activities such as grooming and exploring also entail performance of physical work. Thus, the subjective effort-cost function is also applied to these activities:

$$\dot{\phi}_{sub_L} = f_\phi\left(\dot{\phi}_{obj_L}, K_\phi\right)$$

where

$\dot{\phi}_{obj_L}$ = Average rate of physical work required to perform alternate (``Leisure") activities; units: $J s^{-1}$.

$\dot{\phi}_{sub_L}$ = average subjective rate of exertion entailed in performance of leisure activities in *oomphs* s^{-1}.

K_ϕ = unit-conversion constant; units: *oomphs* J^{-1}

```
[eqn_num, eqn_tab] = add_eqn(eqn_num, eqn_tab, "fphiL", "subjective-effort-cost equatio
```

```
This is equation #10: (fphiL)
```

```
[sym_num, sym_tab] = add_sym(sym_num, sym_tab, "dotPhiObjL", "average objective work ra
[sym_num, sym_tab] = add_sym(sym_num, sym_tab, "dotPhiSubL", "average subjective rate o
```

As in the case of the subjective rate of exertion entailed in work, we allow for drug-induced modulation of the subjective rate of exertion entailed in performance of leisure activities.

$$\dot{\phi}_{sub_{L_{drug}}} = \dot{\phi}_{sub_{L_{vehicle}}} \times K_{ec_L}$$

where

K_{ec_L} = proportional drug-induced change in the subjective rate of exertion required to hold down the lever; unitless

```
[eqn_num, eqn_tab] = add_eqn(eqn_num, eqn_tab, "fphiLdrug", "drug-induced change in the
```

```
This is equation #11: (fphiLdrug)
```

```
[sym_num, sym_tab] = add_sym(sym_num, sym_tab, "KecL", "proportional drug-induced chang
```

In the vehicle condition, K_{ec_L} assumes an implicit value of one.

Core functions

The reward-growth function for brain stimulation reward (BSR)

The reward-growth function translates the aggregate rate of stimulation-induced firing into the intensity of the rewarding effect. Gallistel's team used operant matching to describe this function (Gallistel & Leon, 1991, Leon & Gallistel, 1992, Mark & Gallistel, 1993; Simmons & Gallistel, 1994). Shizgal (2003) proposed the following form for this function:

$$= \hat{R}_{bsr} \times K_{rg} = \left(\frac{F_{firing}{}^{g}}{F_{firing}{}^{g} + F_{firing_{hm}}{}^{g}} \right) \times K_{rg}$$

th function

) = firing rate required to drive reward intensity to half its maximum value; units:~$firings\ \ neuron^{-1}\ \ s^{-1}$

uency required to drive reward intensity to half its maximum value; units:~$pulses\ \ s^{-1}$

t determines the steepness of reward-intensity growth as a function of pulse frequency

n scalar; units: *hedons*

ity produced by F_{firing}; units: *hedons*

ward intensity produced by a pulse frequency of F_{pulse}, which, in turn, produces a firing frequency of F_{firing} in each first-stage neuron; un

```
[eqn_num, eqn_tab] = add_eqn(eqn_num, eqn_tab, "fRbsr", "reward-growth equation for BSR
```

```
This is equation #12: (fRbsr)
```

```
[sym_num, sym_tab] = add_sym(sym_num, sym_tab, "fRbsr", "reward-growth function");
[sym_num, sym_tab] = add_sym(sym_num, sym_tab, "FfiringHM", "firing frequency that proc
[sym_num, sym_tab] = add_sym(sym_num, sym_tab, "FpulseHM", "pulse frequency that produc
[sym_num, sym_tab] = add_sym(sym_num, sym_tab, "g", "reward-growth exponent");
[sym_num, sym_tab] = add_sym(sym_num, sym_tab, "Krg", "output scalar of the reward-grow
[sym_num, sym_tab] = add_sym(sym_num, sym_tab, "Rbsr", "reward intensity produced by Ff
[sym_num, sym_tab] = add_sym(sym_num, sym_tab, "hatRbsr", "normalized reward intensity
[fun_num, fun_tab] = add_fun(fun_num, fun_tab, "fRbsr", "F, Fbend, Fhm, Fro, g, Krg",..
    "Logistic reward-growth function");
```

```
This is function #22: (fRbsr)
```

It follows from the above that

$$\hat{R}_{bsr} = \frac{F_{firing}{}^{g}}{F_{firing}{}^{g} + F_{firing_{hm}}{}^{g}}$$

```
[eqn_num, eqn_tab] = add_eqn(eqn_num, eqn_tab, "fRbsrNorm", "normalized reward-growth e
```

```
This is equation #13: (fRbsrNorm)
```

```
[fun_num, fun_tab] = add_fun(fun_num, fun_tab, "fRbsrNorm", "F, Fbend, Fhm, Fro, g",...
    "Normalized reward-growth function");
```

```
This is function #23: (fRbsrNorm)
```

To accommodate the predicted rescaling of the input to the reward-growth function for oICSS by dopamine-transporter blockade, a scalar, K_{da}, is added to the reward-growth equation in the drug condition of the experiment:

$$R_{bsr_{drug}} = \left[\frac{\left(f_{firing_{drug}} \times K_{da}\right)^g}{\left(F_{firing_{drug}} \times K_{da}\right)^g + \left(F_{firing_{hm_{vehicle}}}\right)^g} \right] \times K_{rg}$$
$$= \left[\frac{\left(f_{firing_{drug}}\right)^g}{\left(f_{firing_{drug}}\right)^g + \left(\frac{F_{firing_{hm_{vehicle}}}}{K_{da}}\right)^g} \right] \times K_{rg}$$

where

K_{da} = scalar representing the boost in dopamine release due to transporter blockade

```
[eqn_num, eqn_tab] = add_eqn(eqn_num, eqn_tab, "RGfunDrug", "reward-growth equation for
```

```
This is equation #14: (RGfunDrug)
```

```
[sym_num, sym_tab] = add_sym(sym_num, sym_tab, "Kda", "scalar representing the boost in
```

Thus,

$$F_{firing_{hm_{drug}}} = \frac{F_{firing_{hm_{vehicle}}}}{K_{da}}$$

```
[eqn_num, eqn_tab] = add_eqn(eqn_num, eqn_tab, "FhmDrug", "FhmDrug as a function of Fhm
```

```
This is equation #15: (FhmDrug)
```

The following graphs plot the reward-growth function for three values of each parameter:

```
logFelecBend = 1.3222;
FelecBend = 10^logFelecBend;
logFelecRO = 2.5587;
FelecRO = 10^logFelecRO;

numF = 121; % Number of pulse frequencies in Felec vector
Felec = logspace(0,3,numF);
FelecMat = repmat(logspace(0,3,numF),3,1); % add two rows in preparation for graphing

Fhm1 = 10^1.6; % The number of elements in the parameter vector must equal the # of row
Fhm2 = 10^1.8;
Fhm3 = 10^2.0;
FhmVec = [Fhm1; Fhm2; Fhm3];
FhmMat = repmat(FhmVec,1,numF); % Store the Fhm values in a matrix of the same size as

gElec1 = 5; % The number of elements in the parameter vector must equal the # of rows i
gElec2 = 10;
gElec3 = 20;
gElecVec = [gElec1; gElec2; gElec3];
gElecMat = repmat(gElecVec,1,numF); % Store the gElec values in a matrix of the same si

Krg1 = 10^-0.2; % The number of elements in the paramer vector must equal the # of rows
Krg2 = 10^0;
Krg3 = 10^0.2;
```

```
KrgVec = [Krg1; Krg2; Krg3];
KrgMat = repmat(KrgVec,1,numF); % Store the Krg values in a matrix of the same size as

RelecMat_Fhm = fRbsr(FelecMat, FelecBend, FhmMat, FelecRO, gElec1, Krg2);
RelecMat_gElect = fRbsr(FelecMat, FelecBend, Fhm2, FelecRO, gElecMat, Krg2);
RelecMat_Krg = fRbsr(FelecMat, FelecBend, Fhm2, FelecRO, gElec1, KrgMat);

TitleStrSemi = 'semi-log';
TitleStrLogLog = 'log-log';

pnam = "F_{hm}";
fnam = "Fhm";
% The data to be plotted must be in columns. Thus, FelecMat and RelecMat are transposed
RG_Fhm_semilog = plot_RG(FelecMat',RelecMat_Fhm',pnam,FhmVec,fnam,TitleStrSemi,'lin');
RG_Fhm_loglog = plot_RG(FelecMat',RelecMat_Fhm',pnam,FhmVec,fnam,TitleStrLogLog,'log');
RG_dual_Fhm = dual_subplot(RG_Fhm_semilog, RG_Fhm_loglog, 'RG_Fhm_semilog_loglog',...
    graphs2files,FigDir);
if show_graphics
    RG_dual_Fhm.Visible = 'on';
end
```

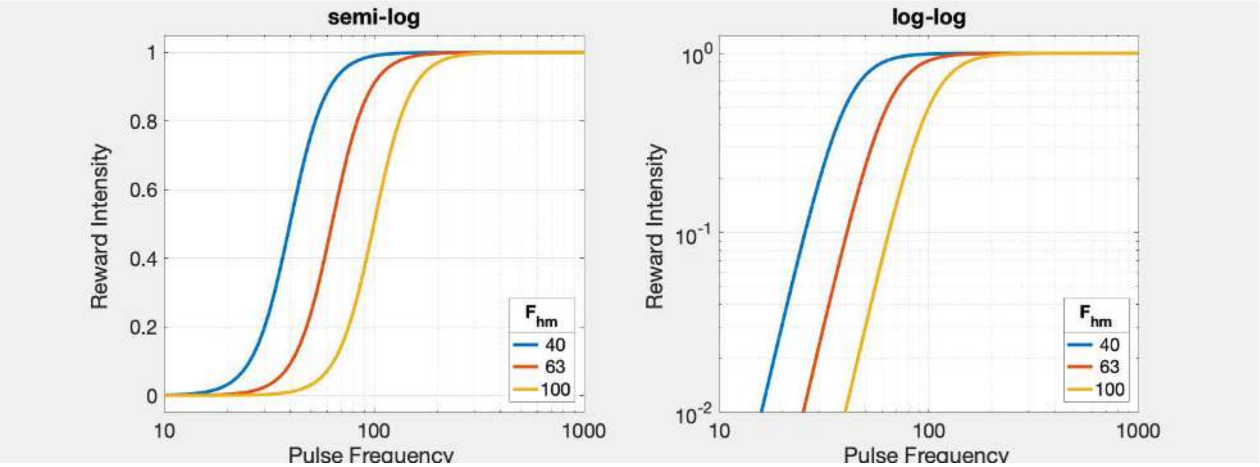


```
[fig_num, fig_tab] = add_fig(fig_num, fig_tab, "RGfunsElecFhm", ...
    "Growth of reward intensity at three values of the position parameter");
```

```
This is figure #3: (RGfunsElecFhm)
```

```
[fun_num, fun_tab] = add_fun(fun_num, fun_tab, "plot_RG", ...
    "Fmat,RMat,pnam,pVec,fnam,TitleStr,linlog, varargins",...
    "function to plot a single reward-growth function");
```

```
This is function #24: (plot_RG)
```

```
[fun_num, fun_tab] = add_fun(fun_num, fun_tab, "dual_subplot", ...
    "g1, g2, dual_sub_out, graphs2files, figdir",...
    "Function to plot two graphs side-by-side");
```

```
This is function #25: (dual_subplot)
```

The semi-log plot is on the left, and the log-log plot is on the right.

These graphs shows how the position parameter rescales the ***input*** required to drive reward intensity to a particular level, thus sliding the reward-growth functions laterally.

```
pnam = "g";
fnam = "g";
RG_gElec_semilog = plot_RG(FelecMat',RelecMat_gElect',pnam,gElecVec,fnam,TitleStrSemi,'
RG_gElec_loglog = plot_RG(FelecMat',RelecMat_gElect',pnam,gElecVec,fnam,TitleStrLogLog,
RG_dual_g = dual_subplot(RG_gElec_semilog, RG_gElec_loglog, 'RG_gElec_semilog_loglog',.
    graphs2files, FigDir);
if show_graphics
    RG_dual_g.Visible = 'on';
end
```

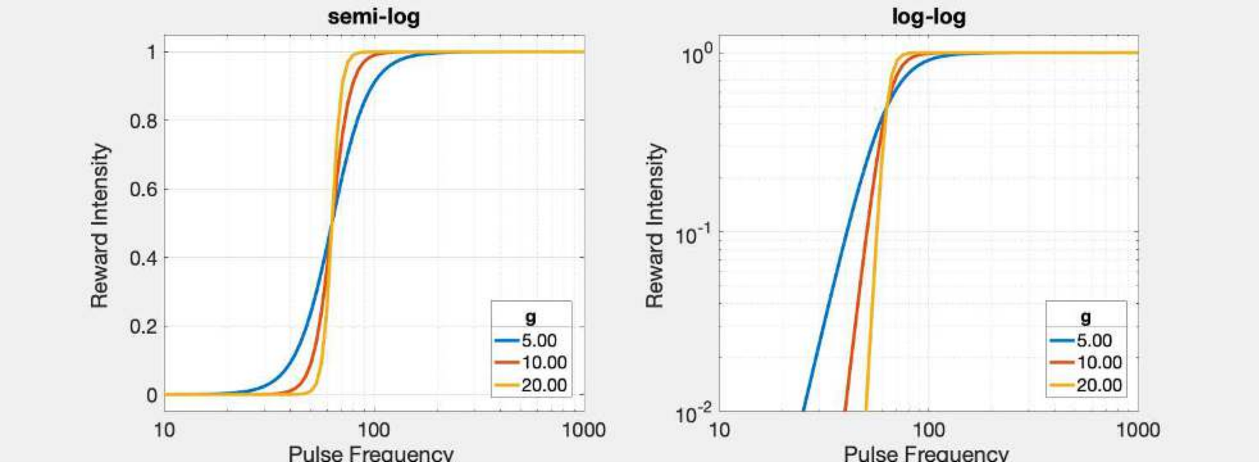


```
[fig_num, fig_tab] = add_fig(fig_num, fig_tab, "RGfunsElecG", ...
    "Growth of reward intensity at three values of the steepness parameter");
```

```
This is figure #4: (RGfunsElecG)
```

The semi-log plot is on the left, and the log-log plot is on the right.

These graphs shows how the steepness parameter rotates the reward-growth functions around their midpoint.

```
pnam = "K_{rg}";
fnam = "Krg";
RG_Krg_semilog = plot_RG(FelecMat',RelecMat_Krg',pnam,KrgVec,fnam,TitleStrSemi,'lin');
RG_Krg_loglog = plot_RG(FelecMat',RelecMat_Krg',pnam,KrgVec,fnam,TitleStrLogLog,'log');
RG_dual_Krg = dual_subplot(RG_Krg_semilog, RG_Krg_loglog, 'RG_Krg_semilog_loglog',...
    graphs2files, FigDir);
if show_graphics
    RG_dual_Krg.Visible = 'on';
end
```

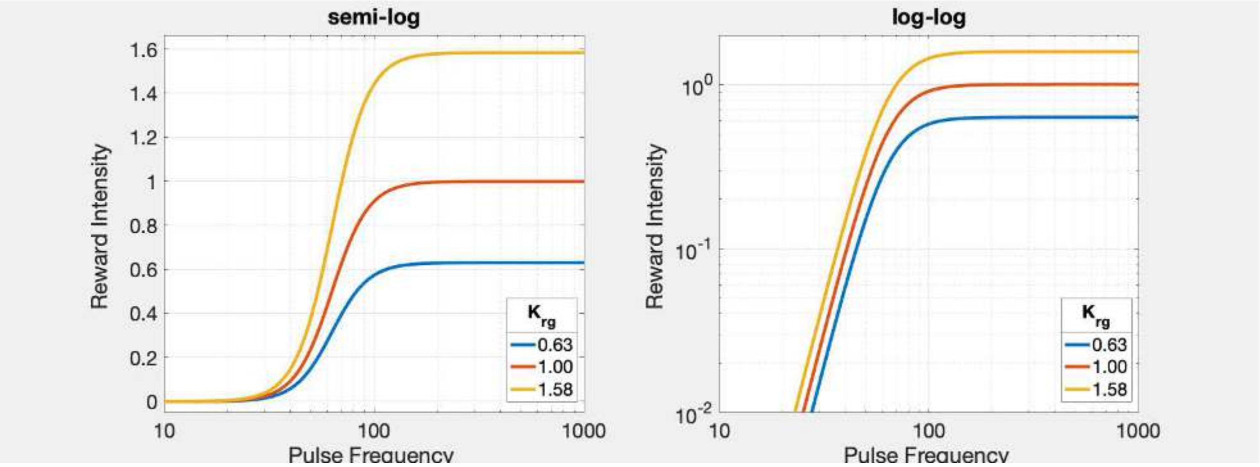


```
[fig_num, fig_tab] = add_fig(fig_num, fig_tab, "RGfunsElecKrg", ...
    "Growth of reward intensity at three values of the output-scaling parameter");
```

```
This is figure #5: (RGfunsElecKrg)
```

These graphs show how the parameter, (K_{rg}), rescales the ***output*** of the reward-growth function vertically, thus determining its asymptotic level.

The preceding graphs show how rescaling of the input to and output from the reward-growth function produce orthogonal changes. This is true as long as as the firing of the directly stimulated neurons keeps pace with the pulse frequency. However, once the value of the position parameter nears the maximum firing frequency of the directly stimulated neurons (e.g., when the current is very low), rightward shifts of the reward-growth function, such as those produced by further decreases in current, are accompanied by decreases in its upper asymptote. The neurons can no longer fire fast enough to drive reward intensity to the same maximum as was achieved when the value of the position parameter was lower (e.g., because the current was higher). This has implications for the interpretation of changes in the location parameters of the reward mountain, as discussed below.

```
Fhm4 = 10^2.0;
Fhm5 = 10^2.3;
Fhm6 = 10^2.6
```

```
Fhm6 = 398.1072
```

```
FhmVec = [Fhm4; Fhm5; Fhm6];
FhmMat = repmat(FhmVec,1,numF); % Store the Fhm values in a matrix of the same size as

RelecMat_FhmHi = fRbsr(FelecMat, FelecBend, FhmMat, FelecRO, gElec1, Krg2);
TitleStrSemi = 'HiF-semi-log';
TitleStrLogLog = 'HiF-log-log';
pnam = "F_{hm}";
fnam = "FhmHi";
% The data to be plotted must be in columns. Thus, FelecMat and RelecMat are transposed
RG_FhmHi_semilog = plot_RG(FelecMat',RelecMat_FhmHi',pnam,FhmVec,fnam,TitleStrSemi,'lin
RG_FhmHi_loglog = plot_RG(FelecMat',RelecMat_FhmHi',pnam,FhmVec,fnam,TitleStrLogLog,'lo
RG_dual_FhmHi = dual_subplot(RG_FhmHi_semilog, RG_FhmHi_loglog, 'RG_FhmHi_semilog_loglo
    graphs2files,FigDir);
```

```
if show_graphics
    RG_dual_FhmHi.Visible = 'on';
end
```

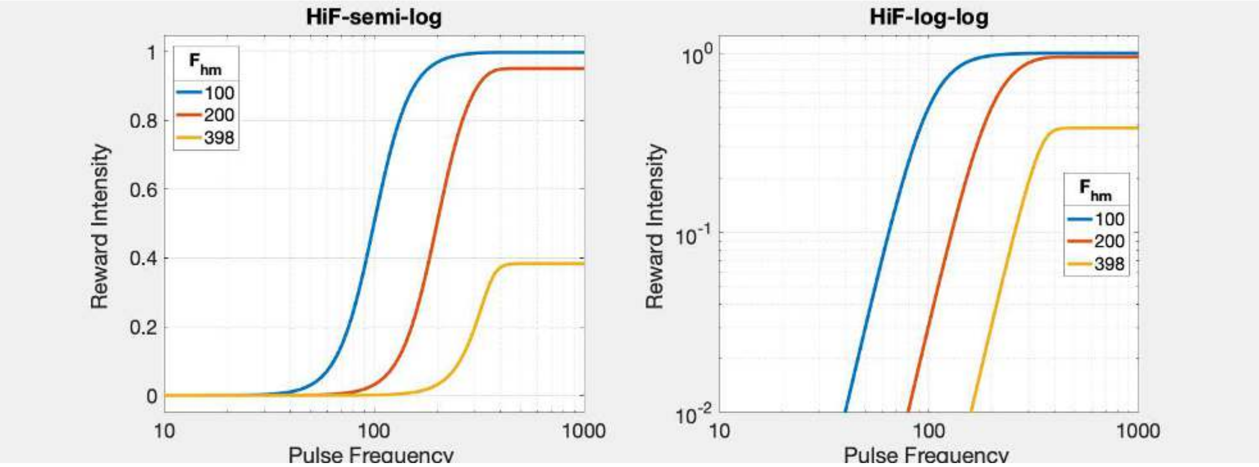


```
[fig_num, fig_tab] = add_fig(fig_num, fig_tab, "RGfunsElecFhmHi", ...
    "Growth of reward intensity at three values of the position parameter near FelecRO'
```

```
This is figure #6: (RGfunsElecFhmHi)
```

When the middle of the frequency roll-off zone ($F_{elect_{RO}}$) is positioned at 362 pulses s^{-1} (the median value reported by Solomon et al., 2015) and F_{hm} is 100 pulses s^{-1}, the normalized reward-growth function approaches an upper asymptote of one, as expected. However, doubling F_{hm} to ~200 pulses s^{-1} moderately decreases the upper asymptote. A further doubling to ~400 pulses s^{-1}pushes F_{hm} past $F_{elect_{RO}}$, markedly truncating the growth of reward intensity.

```
close all;
```

The location parameter of the reward-growth function for BSR

The location parameter of the reward-growth function is the firing rate that drives reward intensity to half its maximal value. The value of this parameter depends on the number of stimulated first-stage neurons, *N*, and the interval during which the stimulation train elevates their firing rate, D_{burst}.

A prior study of temporal integration in the neural circuitry responsible for eICSS of the MFB (Sonnenschein et al., 2003) implies the following form for the function that determines $F_{firing_{hm}}$:

$$[illegible]_{train}, N) = \frac{\rho_{\Pi} \times \left(1 + \frac{C}{D_{burst}}\right)}{N}$$

e: train duration at which $F_{firing_{hm}}$is twice the value of ρ_{Π}; units: s

e rate of firing required to produce a reward of half-maximal intensity when the train duration is infinite; uni

```
[eqn_num, eqn_tab] = ...
    add_eqn(eqn_num, eqn_tab, "fHfiring", "strengh-duration function for trains express
```

```
This is equation #16: (fHfiring)
```

```
[fun_num, fun_tab] = add_fun(fun_num, fun_tab, "fHfiring", "C, D, N, RhoPi, varargin",.
    "strengh-duration function for trains expressed as a firing frequency");
```

```
This is function #26: (fHfiring)
```

```
[eqn_num, eqn_tab] = ...
    add_eqn(eqn_num, eqn_tab, "fH", "strengh-duration function for trains expressed as
```

```
This is equation #17: (fH)
```

```
[fun_num, fun_tab] = add_fun(fun_num, fun_tab, "fH", "C, D, RhoPi",...
    "strengh-duration function for trains expressed as an aggreagate firing frequency")
```

```
This is function #27: (fH)
```

```
[sym_num, sym_tab] = add_sym(sym_num, sym_tab, "Cmfb", "chronaxie of the strength-durat
[sym_num, sym_tab] = add_sym(sym_num, sym_tab, "Dtrain", "duration of an electrical pul
```

```
Symbol Dtrain has already been entered.
```

```
[sym_num, sym_tab] = add_sym(sym_num, sym_tab, "RhoPi", "rheobase of the strength-durat

[fun_num, fun_tab] = add_fun(fun_num, fun_tab, "fH", "C, D, rhoPi",...
    "Strength-duration function for trains; generates aggregate firing rate required to
```

```
Function fH has already been entered.
```

We assume that $D_{train} = D_{burst}$.

The counter model is implicit in the equation for $F_{firing_{hm}}$. According to this model, the aggregate rate of firing in the directly stimulated neurons determines the intensity of the rewarding effect.

The payoff from work

In keeping with the generalized matching law (Killeen, 1972), the benefit from work and its costs are combined in scalar fashion to yield a net payoff. We can format the expression for the payoff as a ratio of two rates (see: Gallistel & Gibbon, 2000):

$$U_W = f_U\left(\frac{R_{bsr}}{P_{sub}},\ p_{sub},\ \dot{\phi}_{sub_W}\right) = \frac{\frac{R_{bsr}}{P_{sub}} \times p_{sub}}{\dot{\phi}_{sub_W}}$$

where

$f_U =$ utility function

$U_W =$ payoff from a train of rewarding stimulation; units: $hedons\ \ oomph^{-1}$

```
[eqn_num, eqn_tab] = add_eqn(eqn_num, eqn_tab, "fUW", "payoff from work");
```

```
This is equation #18: (fUW)
```

```
[sym_num, sym_tab] = add_sym(sym_num, sym_tab, "fU", "utility function");
[sym_num, sym_tab] = add_sym(sym_num, sym_tab, "UW", "payoff from work");
```

or as a benefit/cost ratio:

$$U_W = f_U\left(\frac{R_{bsr}}{P_{sub}},\ p_{sub},\ \dot{\phi}_{sub_W}\right) = \frac{R_{bsr} \times p_{sub}}{P_{sub} \times \dot{\phi}_{sub_W}}$$

The reward-growth function for leisure activities

We define a reward rate for the leisure activities that compete with pursuit of BSR as follows:

$$\dot{R}_{aa} = f_R(\_\_) = \hat{\dot{R}}_{aa} \times K_{aa}$$

where

K_{aa} = alternate-activity scalar; units:~$hedons\ \ oomphs^{-1}$

$\dot{R}_{aa}$ = average rate of reward from alternate (leisure) activities that compete with pursuit of BSR; units:~$hedons\ \ oomph^{-1}$

$\hat{\dot{R}}_{aa}$ = normalized rate of reward from alternate (leisure) activities; unitless. $0 \leq \hat{\dot{R}}_{aa} \leq 1$

$(\_\_)$ = the unknown variables that give rise to $\dot{R}_{aa}$

```
[eqn_num, eqn_tab] = add_eqn(eqn_num, eqn_tab, "fRaa", "Leisure reward rate");
```

```
This is equation #19: (fRaa)
```

```
[sym_num, sym_tab] = add_sym(sym_num, sym_tab, "Kaa", "subjective reward-rate scalar");
[sym_num, sym_tab] = add_sym(sym_num, sym_tab, "DotRaa", "Average subjective reward rat
[sym_num, sym_tab] = add_sym(sym_num, sym_tab, "DotHatRaa", "normalized average subject
[fun_num, fun_tab] = add_fun(fun_num, fun_tab, "fRaa", "Raa,Kaa",...
    "reward-growth function for leisure activities");
```

```
This is function #28: (fRaa)
```

The payoff from leisure activities

$$U_L = f_U\left(\dot{R}_{aa},\ \dot{\phi}_{sub_L}\right) = \frac{\hat{\dot{R}}_{aa} \times K_{aa}}{\dot{\phi}_{sub_L}}$$

```
[eqn_num, eqn_tab] = add_eqn(eqn_num, eqn_tab, "fUL", "payoff from leisure");
```

```
This is equation #20: (fUL)
```

```
[sym_num, sym_tab] = add_sym(sym_num, sym_tab, "UL", "payoff from leisure activities");
```

The core → shell function

The behavioral-allocation function

The payoffs from pursuit of BSR and engagement in leisure activities are used by the sole core→shell function to compute the allocation of time to pursuit of BSR.

$$\text{time allocation} = \frac{(\text{payoff from BSR})^a}{(\text{payoff from BSR})^a + (\text{payoff from alternate activities})^a}$$

where

a = payoff-sensitivity exponent

```
[sym_num, sym_tab] = add_sym(sym_num, sym_tab, "a", "payoff-sensitivity exponent");
```

The exponent (a) determines how abruptly time allocation changes as a function of changes in payoff.

Restating this equation symbolically, we obtain

$$T = T_{min} + \left[(T_{max} - T_{min}) \times \frac{(U_W)^a}{(U_W)^a + (U_L)^a} \right]$$

where

T = time allocation

T_{max} = maximum time allocation

T_{min} = minimum time allocation

```
[eqn_num, eqn_tab] = ...
    add_eqn(eqn_num, eqn_tab, "TU", "Time allocation defined in terms of payoffs");
```

```
This is equation #21: (TU)
```

```
[sym_num, sym_tab] = add_sym(sym_num, sym_tab, "T", "time allocation");
[sym_num, sym_tab] = add_sym(sym_num, sym_tab, "Tmax", "maximum time allocation");
[sym_num, sym_tab] = add_sym(sym_num, sym_tab, "Tmin", "minimum time allocation");
```

We also define a normalized measure of time allocation

$$\hat{T} = \frac{(U_W)^a}{(U_W)^a + (U_L)^a} = \frac{T - T_{min}}{T_{max} - T_{min}}$$

where $0 \leq \hat{T} \leq 1$

```
[eqn_num, eqn_tab] = ...
    add_eqn(eqn_num, eqn_tab, "Tnorm", "Normalized time allocation");
```

```
This is equation #22: (Tnorm)
```

as well as a value of time allocation, T_{mid}, at which the payoffs from work and leisure are equal, and thus, time allocation falls halfway between its minimal and maximal values:

$$T_{mid} = T_{min} + \left(\frac{T_{max} - T_{min}}{2} \right)$$

```
[eqn_num, eqn_tab] = ...
    add_eqn(eqn_num, eqn_tab, "Tmid", "Mid-range time allocation");
```

```
This is equation #23: (Tmid)
```

When the reward intensity produced by the stimulation approaches its upper asymptote, the price at which $T = T_{mid}$ is:

$$P_{sub_e}(P_{obj_e}) = \frac{K_{rg}}{K_{aa} \times K_{ec_W}} \times \frac{p_{Sub} \times \hat{R}_{bsr_{max}}}{\hat{\phi}_{sub_W} \times U_L}$$

where

P_{obj_e} = objective price at which $T = T_{mid}$ when $\hat{R}_{bsr} = \hat{R}_{bsr_{max}}$

P_{sub_e} = subective price at which $T = T_{mid}$ when $\hat{R}_{bsr} = \hat{R}_{bsr_{max}}$

```
[eqn_num, eqn_tab] = ...
    add_eqn(eqn_num, eqn_tab, "Psub_e", "Definition of Psub_e");
```

```
This is equation #24: (Psub_e)
```

```
[fun_num, fun_tab] = add_fun(fun_num, fun_tab, "PsubEfun", ...
    "dotPhiObj, Kaa, Kec, Krg, Raa, pObj, RnormMax, varargin",...
    "function to compute PsubE");
```

```
Function PsubEfun has already been entered.
```

To obtain an expression for P_{obj_e}, we back-solve the subjective-price equation, as shown above in

```
disp(string({strcat({'Equation '},num2str(eqn_tab.Number(eqn_tab.Name=='fPbacksolved'))
```

```
Equation 7
```

```
[fun_num, fun_tab] = add_fun(fun_num, fun_tab, "PobjEfun", ...
    "dotPhiObj, Kaa, Kec, Krg, ObjAA, pObj, PsubBend, PsubMin, RnormMax, varargin",...
    "Back-solution of the subjective-price function to return PobjE from PsubE");
```

```
Function PobjEfun has already been entered.
```

We can now define the equation for normalized time allocation as:

$$\hat{T} = \frac{(\hat{R}_{bsr})^a}{(\hat{R}_{bsr})^a + \left[\hat{R}_{bsr_{max}} \times \left(\frac{P_{sub}}{P_{sub_e}}\right)\right]^a}$$

```
[eqn_num, eqn_tab] = ...
    add_eqn(eqn_num, eqn_tab, "TnormExpand", "Normalized time allocation expanded");
```

```
This is equation #25: (TnormExpand)
```

and the full equation for time allocation as:

$$T = T_{min} + (T_{max} - T_{min}) \times \frac{\left(\frac{(f_{firing})(F_{pulse})^g}{F_{firing}(F_{pulse})^g + F_{firing}(F_{pulse_{hm}})^g}\right)^a}{\left(\frac{F_{firing}(F_{pulse})^g}{F_{firing}(F_{pulse})^g + F_{firing}(F_{pulse_{hm}})^g}\right)^a + \left(\widehat{R}_{bsr_{max}} \times \left[\frac{P_{sub}(P_{obj})}{P_{sub}(P_{obj_e})}\right]\right)^a}$$

```
[eqn_num, eqn_tab] = ...
    add_eqn(eqn_num, eqn_tab, "Tfull", "Full 6-parameter time-allocation equation");
```

```
This is equation #26: (Tfull)
```

```
[fun_num, fun_tab] = add_fun(fun_num, fun_tab, "TAfun", "a, F, Fbend, FpulseHM, Fro, g,
    "6-parameter TA function");
```

```
This is function #29: (TAfun)
```

The reward mountain

```
disp(string({strcat({'Equation '},num2str(eqn_tab.Number(eqn_tab.Name=='Tfull')))}));
```

```
Equation 26
```

defines the surface of the reward-mountain model in a three-dimensional space defined by the two independent variables, the pulse frequency (F_{pulse}) and the price (P_{obj}), and a single dependent variable, time allocation (T). Thus, this equation bridges the core of the model by linking three shell variables, two inputs and one output; the bridge is constructed from the shell → core, core, and core → shell functions. The slope of the mountain surface depends on the payoff-sensitivity parameter (a) and the steepness parameter of the reward-growth equation, (g). The location of the mountain in the plane defined by the independent variables is determined by the two location parameters: whereas P_{obj_e} positions the mountain along the price axis, $F_{pulse_{hm}}$ positions the mountain along the pulse-frequency axis. Finally, the altitude of the "valley floor" and "summit" are set by T_{min} and T_{max}, respectively.

To simulate the mountain model, we must obtain values for the position parameters. $F_{pulse_{hm}}$. We proceed as follows:

```
C = 0.473; % median from Sonnenschein et al., 2003
Dtrain = 0.5; % train duration in eICSS reward-mountain studies
logFelecBend = 1.3222; % from Solomon et al., 2015
FelecBend = 10^logFelecBend;
logFelecRO = 2.5587; % from Solomon et al., 2015
FelecRO = 10^logFelecRO;
FPmax =1000; % for determining RbsrNormMax. This value is sufficiently above FelecRO to
N = 100; % arbitrary
RhoPi = 5000; % arbitrary
% RhoPi/N = 50, which is roughly consistent with Sonnenchein et al., 2003

FmfbHM = FpulseHMfun(C, Dtrain, FelecBend, FPmax, FelecRO, N, RhoPi)
```

```
FmfbHM = 97.3001
```

```
[fun_num, fun_tab] = add_fun(fun_num, fun_tab, "FpulseHMfun", ...
    "C, D, Fbend, FPmax, Fro, N, RhoPi, varargin",...
    "Function to compute the pulse frequency that produces half-maximal reward intensit
```

```
This is function #30: (FpulseHMfun)
```

The parameter that determines the location of the mountain along the price axis is P_{obj_e}.

```
dotPhiObj = 1;
Kaa = 1;
Kec = 1;
Krg = 1;
dotRaa = 0.1;
pObj = 1;
PsubBend = 0.5;
PsubMin = 1.82;
```

Before computing P_{obj_e}, we must determine the maximum value of the normalized reward intensity ($\hat{R}_{bsr_{max}}$).

If the value of the position parameter of the reward-growth function is too high given the frequency roll-off parameters, $R_{norm_{Max}}$ will be less than one.

```
[sym_num, sym_tab] = add_sym(sym_num, sym_tab, "RnormMax", "maximum normalized reward i
g = 5;
%RnormMax = RnormMaxFun(Fmax, FelecBend, FmfbHM, FelecRO, g)
RnormMax = fRbsrNorm(FPmax, FelecBend, FmfbHM, FelecRO, g)
```

```
RnormMax = 0.9986
```

```
PobjE = PobjEfun(dotPhiObj, Kaa, Kec, Krg, dotRaa, pObj, PsubBend, PsubMin, RnormMax)
```

```
PobjE = 9.9860
```

We will now assign a value to the price-sensitivity parameter, *a*, and will simulate the mountain surface. (See ***Functions composing the reward-mountain model*** below.)

```
a = 3;
Felec = logspace(0,3,121)'; % column variable
Pobj = logspace(0,3,121); % row variable
Tmfb = TAfun(a, Felec, FelecBend, FmfbHM, FelecRO, g, Pobj, PobjE, PsubBend, PsubMin, F
% n.b., numel(Felec) = numel(Pobj). Felec has been transposed. Thus, Tmfb is a square m
MTN = plot_MTN(Felec, Pobj, Tmfb, 'off', 'MTN', 'reward mountain', ...
    graphs2files, FigDir);
if show_graphics
    MTN.Visible = 'on';
end
```

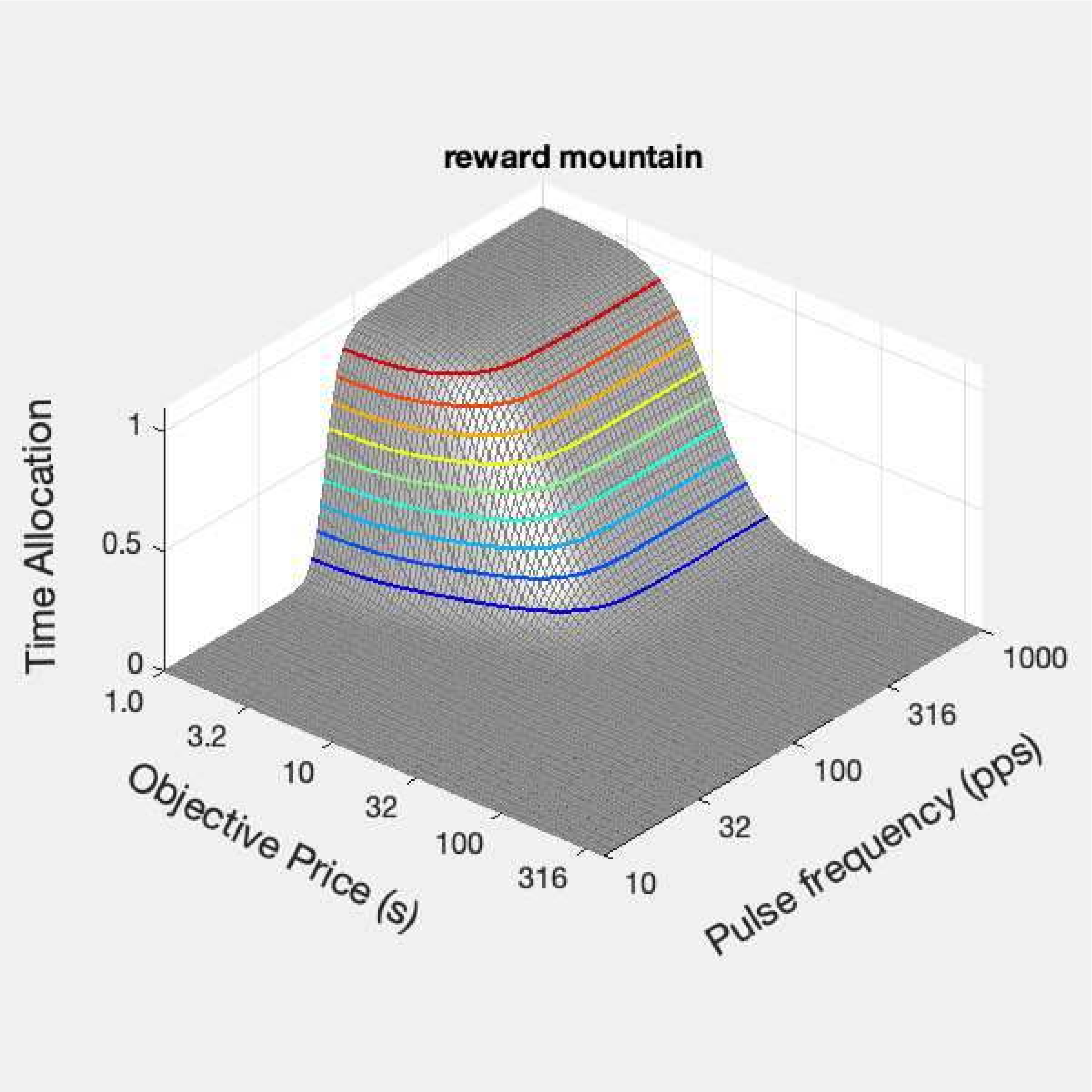


```
[fig_num, fig_tab] = add_fig(fig_num, fig_tab, "Mountain", ...
    "3D plot of the reward mountain");
```

```
This is figure #7: (Mountain)
```

```
[fun_num, fun_tab] = add_fun(fun_num, fun_tab, "plot_MTN", ...
    "F, Pobj, T, Visible, mtn_root, title_str, graphs2files, figdir, varargin",...
    "Function to plot a single reward mountain");
```

```
This is function #31: (plot_MTN)
```

```
clearvars('-except',keepVars{:})
keepVars = who; % Restore cell array containing names of variables to be retained
toc
```

```
Elapsed time is 25.821254 seconds.
```

Moving the mountain: validation studies

```
tic;
close all;
```

We have carried out a series of experiments that test the mountain model. Given that there are two independent position parameters $\{P_{sub\_e}, F_{elec_{HM}}\}$, it should be possible to move the mountain independently along either of the axes representing the objective price and the pulse frequency. This prediction has been tested in three ways.

Effect of varying the stimulation current

Changing the stimulation current alters the number of reward-related axons recruited. According to

```
disp(string({strcat({'Equation '},num2str(eqn_tab.Number(eqn_tab.Name=='FmfbHM')))}));
```

```
Equation
```

```
disp(string({strcat({'Equation '},num2str(eqn_tab.Number(eqn_tab.Name=='FelecHM')))}));
```

```
Equation
```

this will change the value of the parameter that positions the mountain along the pulse-frequency axis $(F_{pulse_{hm}})$, but according to

```
disp(string({strcat({'Equation '},num2str(eqn_tab.Number(eqn_tab.Name=='Psub_e')))}));
```

```
Equation 24
```

and

```
disp(string({strcat({'Equation '},num2str(eqn_tab.Number(eqn_tab.Name=='fPbacksolved'))
```

```
Equation 7
```

changing the current will have no effect on the value of the parameter (P_{obj_e}) that positions the mountain along the price axis. We will now simulate the effect of changing the current by solving the time-allocation equation for two different values of *N*.

```
C = 0.473; % median from Sonnenschein et al., 2003
D = 0.5; % typical train duration
logFelecBend = 1.3222; % from Solomon et al., 2015
FelecBend = 10^logFelecBend;
logFelecRO = 2.5587; % from Solomon et al., 2015
FelecRO = 10^logFelecRO;
FPmax =1000; % for determining RbsrNormMax. This value is sufficiently above FelecRO to
N = [79,158]; % Number of neurons recruited by low & hi currents (2-element vector)
RhoPi = 5000; % arbitrary

FmfbHM = FpulseHMfun(C, D, FelecBend, FPmax, FelecRO, N, RhoPi) % FmfbHM is a 2-element
```

```
FmfbHM = 1×2
  123.1648   61.5823
```

```
dotPhiObj = 1;
Kaa = 1;
Kec = 1;
Krg = 1;
dotRaa = 0.1;
pObj = 1;
PsubBend = 0.5;
PsubMin = 1.82;

FPmax = 1000; % This value is sufficiently above FelecRO to maximize the firing rate
g = 5;
RnormMax = fRbsrNorm(FPmax, FelecBend, FmfbHM, FelecRO, g) % RnormMax is a 2-element ve
```

```
RnormMax = 1×2
    0.9955    0.9999
```

```
PobjE = PobjEfun(dotPhiObj, Kaa, Kec, Krg, dotRaa, pObj, PsubBend, PsubMin, RnormMax)
```

```
PobjE = 1×2
    9.9546    9.9986
```

```
% PobjE is a 2-element vector

a = 3;
Felec = logspace(0,3,121)'; % column variable
Pobj = logspace(0,3,121); % row variable
Tmfb1 = TAfun(a, Felec, FelecBend, FmfbHM(1), FelecRO, g, Pobj, PobjE(1), ...
    PsubBend, PsubMin, RnormMax(1));
Tmfb2 = TAfun(a, Felec, FelecBend, FmfbHM(2), FelecRO, g, Pobj, PobjE(2), ...
    PsubBend, PsubMin, RnormMax(2));
% n.b., numel(Felec) = numel(Pobj). Felec has been transposed. Thus, Tmfb is a square m
MTNloI = plot_MTN(Felec, Pobj, Tmfb1, 'off', 'MTNloI', 'low current', ...
    graphs2files, FigDir);
MTNhiI = plot_MTN(Felec, Pobj, Tmfb2, 'off', 'MTNhiI', 'high current', ...
    graphs2files, FigDir);
dual_I_plot = dual_subplot(MTNloI, MTNhiI, 'MTNloI_hiI',...
    graphs2files,FigDir);
if show_graphics
    dual_I_plot.Visible = 'on';
end
```

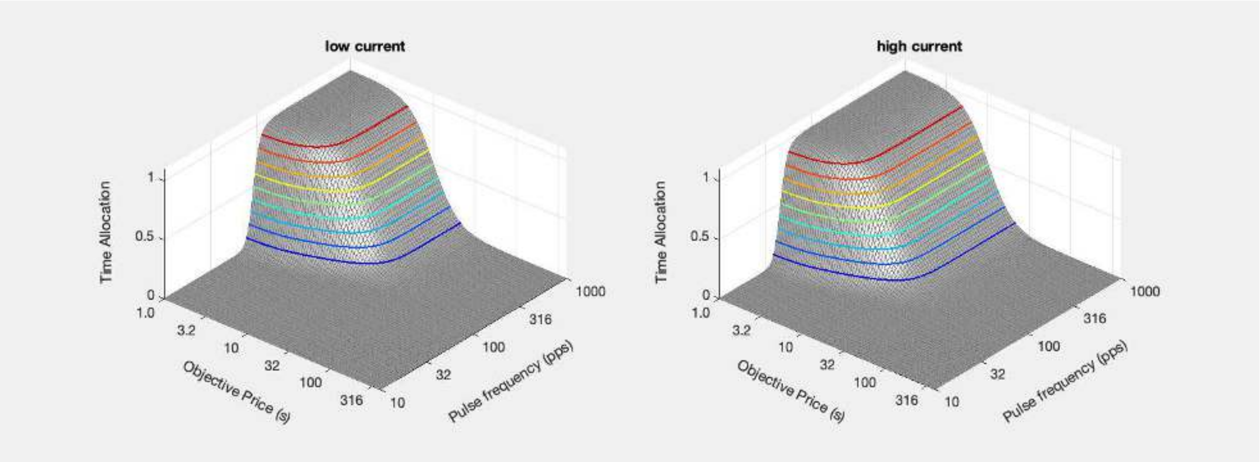


```
[fig_num, fig_tab] = add_fig(fig_num, fig_tab, "LoHiI_mtns", ...
    "Effect of changing the current on the reward mountain");
```

```
This is figure #8: (LoHiI_mtns)
```

(See ***Functions that plot graphs and set attributes*** below.)

Although the change in the position of the mountain following and increase in the number of reward-related neurons recruited can be discerned readily in the 3D plots, the effect is best depicted by means of contour graphs and a bar graph showing the changes in the position paramter. The contour graphs capture in two dimensions all of the spatial information in the 3D plots.

```
ContLoI = plot_contour(Felec, Pobj, Tmfb1, PobjE(1), FmfbHM(1), 'off', 'ContLoI', 'low
    strcat({'N = '}, num2str(N(1))), graphs2files, FigDir);
ContHiI = plot_contour(Felec, Pobj, Tmfb2, PobjE(2), FmfbHM(2), 'off', 'ContHiI', 'high
    strcat({'N = '}, num2str(N(2))), graphs2files, FigDir);
bg_LoHiI = plot_bg(FmfbHM(1), FmfbHM(2), PobjE(1), PobjE(2), 'off', 'bg_LoHiI',...
    graphs2files, FigDir);
bg_root ='bg_LoHiI';
quad_I_plot = quad_subplot(ContLoI, ContHiI, bg_LoHiI, 'quad_LoHiI', bg_root, ...
    graphs2files, FigDir);
if show_graphics
    quad_I_plot.Visible = 'on';
end
```

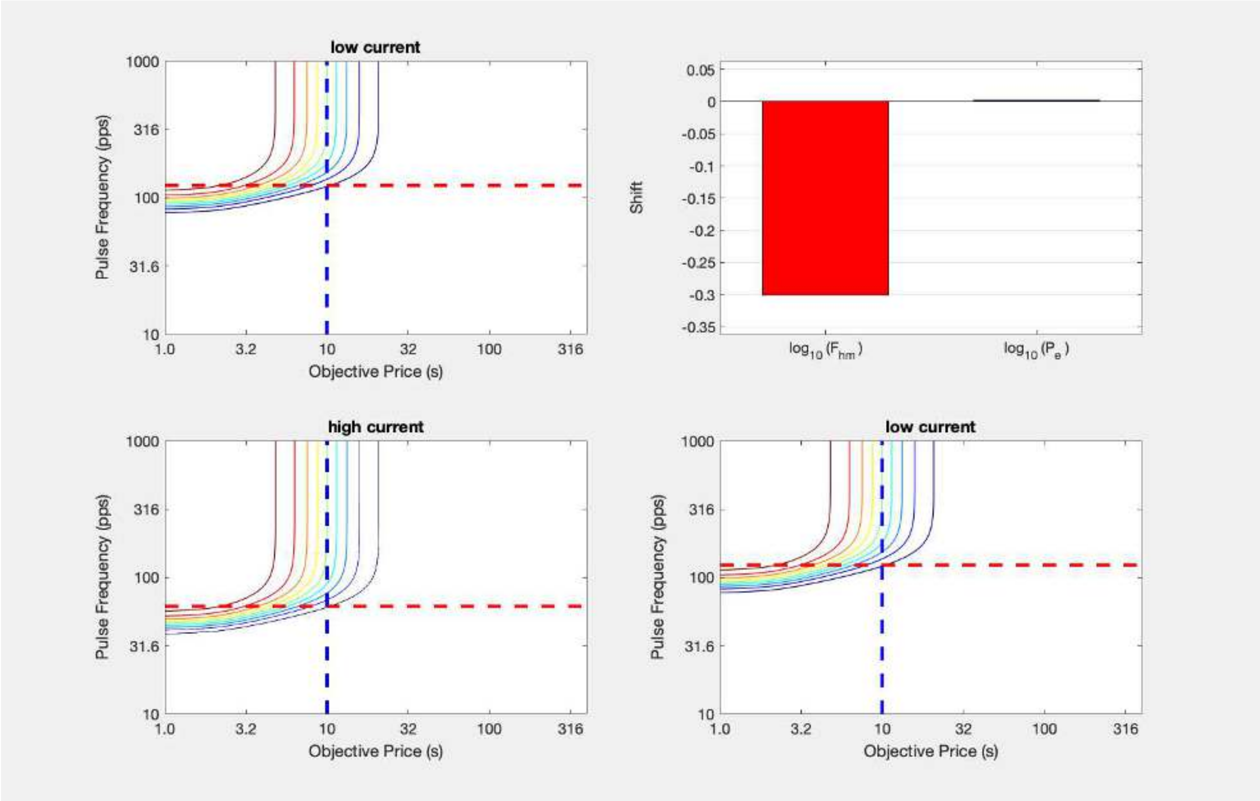


```
[fig_num, fig_tab] = add_fig(fig_num, fig_tab, "quad_LoHiI", ...
    "Effect of changing the number of recruited neurons on the position of the reward m
```

This is figure #9: (quad_LoHiI)

```
[fun_num, fun_tab] = add_fun(fun_num, fun_tab, "plot_contour", ...
    "F, Pobj, T, Pobj_e, Fhm, Visible, mtn_root, title_str, annot_str, graphs2files, fi
    "Function to plot the contour graph of a single mountain");
```

This is function #32: (plot_contour)

```
[fun_num, fun_tab] = add_fun(fun_num, fun_tab, "quad_subplot", ...
    "cont1, cont2, bg, quad_sub_out, bg_root, graphs2files, figdir",...
    "Function to plot four graphs in a 2 x 2 mosaic");
```

This is function #33: (quad_subplot)

The contour graph for the low-current condition is shown twice, once in the upper-left panel and once in the lower-right panel. By comparing the horizontal position of the mountain in the left column, the reader can quickly discern whether there has been a shift along the price axis, and by comparing the vertical position of the mountains in the bottom row, the reader can quickly discern whether there has been a shift along the pulse-frequency axis. As required by the mountain model, the latter shift is observed when the number of neurons recruited is increased due to boost in the stimulation current. The bar graph in the upper right provides a summary of the simulated shifts. The tiny rightward shift along the price axis is due to the fact that $R_{norm_{Max}}$ is a little closer to one at the higher current than at the lower current (due to the fact that F_{hm} is lower).

Arvanitogiannis and Shizgal (2008) tested the effect of varying the stimulation current on the position of the reward mountain. In all four rats, the mountain shifted along the pulse-frequency axis as predicted. In two of

these rats, there was no corresponding shift along the price axis, but rightward shifts were seen in the remaining two subjects. This deviation from the predictions was attributed to the heterogeneity of the stimulated neurons. Such a deviation would be expected if the stimulation activated two subpopulations of reward-related neurons that project to separate integrators with converging outputs (Arvanitogiannis, Waraczynski & Shizgal, 1996; Arvanitogiannis & Shizgal, 2008). The convergence model described below provides an example of such an arrangement.

```
clearvars('-except',keepVars{:})
keepVars = who; % Restore cell array containing names of variables to be retained
toc
```

```
Elapsed time is 10.356255 seconds.
```

Effect of varying the train duration

```
tic;
close all;
```

In

```
disp(string({strcat({'Figure '},num2str(fig_tab.Number(fig_tab.Name=='quad_LoHiI')))}))
```

```
Figure 9
```

the mountain shifts along the pulse-frequency axis because changing the current alters the denominator of the ratio on the right-hand side of

```
disp(string({strcat({'Equation '},num2str(eqn_tab.Number(eqn_tab.Name=='fH')))}));
```

```
Equation 17
```

Changing the train duration should produce the same qualitative effect as changing the current, but by altering the numerator of the ratio on the right-hand side of

```
disp(string({strcat({'Equation '},num2str(eqn_tab.Number(eqn_tab.Name=='fH')))}));
```

```
Equation 17
```

instead. By increasing the duration of the train, there is more time for temporal summation and thus, the pulse frequency required to produce a reward of half-maximal intensity decreases.

```
C = 0.473; % median from Sonnenschein et al., 2003
D = 0.5; % typical train duration
logFelecBend = 1.3222; % from Solomon et al., 2015
FelecBend = 10^logFelecBend;
logFelecRO = 2.5587; % from Solomon et al., 2015
FelecRO = 10^logFelecRO;
FPmax =1000; % for determining RbsrNormMax. This value is sufficiently above FelecRO to
N = 126;
D = [0.25,1.00]; % Short & long train durations (2-element vector)
RhoPi = 5000; % arbitrary
% RhoPi/N = 50, which is roughly consistent with Sonnenchein et al., 2003
```

```
FmfbHM = FpulseHMfun(C, D, FelecBend, FPmax, FelecRO, N, RhoPi) % FmfbHM is a 2-element
```

```
FmfbHM = 1×2
  114.7621   58.4524
```

```
dotPhiObj = 1;
Kaa = 1;
Kec = 1;
Krg = 1;
dotRaa = 0.1;
pObj = 1;
PsubBend = 0.5;
PsubMin = 1.82;

g = 5;
RnormMax = fRbsrNorm(FPmax, FelecBend, FmfbHM, FelecRO, g) % RnormMax is a 2-element ve
```

```
RnormMax = 1×2
    0.9968    0.9999
```

```
PobjE = PobjEfun(dotPhiObj, Kaa, Kec, Krg, dotRaa, pObj, PsubBend, PsubMin, RnormMax)
```

```
PobjE = 1×2
    9.9681    9.9989
```

```
% PobjE is a 2-element vector

a = 3;
Felec = logspace(0,3,121)'; % column variable
Pobj = logspace(0,3,121); % row variable
Tmfb1 = TAfun(a, Felec, FelecBend, FmfbHM(1), FelecRO, g, Pobj, ...
    PobjE(1), PsubBend, PsubMin, RnormMax(1));
Tmfb2 = TAfun(a, Felec, FelecBend, FmfbHM(2), FelecRO, g, Pobj, ...
    PobjE(2), PsubBend, PsubMin, RnormMax(2));

MTNloD = plot_MTN(Felec, Pobj, Tmfb1, 'off', 'MTNloD', 'short-duration train', ...
    graphs2files, FigDir);
MTNhiD = plot_MTN(Felec, Pobj, Tmfb2, 'off', 'MTNhiD', 'long-duation train', ...
    graphs2files, FigDir);
dual_D_plot = dual_subplot(MTNloD, MTNhiD, 'MTNLoHiD',...
    graphs2files,FigDir);
if show_graphics
    dual_D_plot.Visible = 'on';
end
```

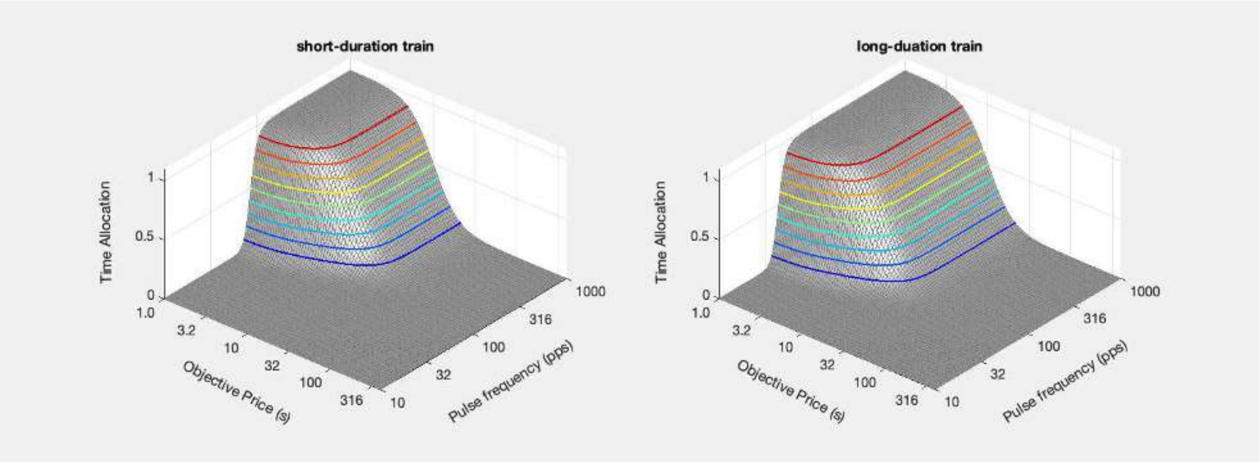


```
[fig_num, fig_tab] = add_fig(fig_num, fig_tab, "LoHiD_mtns", ...
    "Effect of changing the train duration on the reward mountain");
```

```
This is figure #10: (LoHiD_mtns)
```

```
ContLoD = plot_contour(Felec, Pobj, Tmfb1, PobjE(1), FmfbHM(1), 'off', 'ContLoD', 'shor
    strcat({'D = '}, num2str(D(1))), graphs2files, FigDir);
ContHiD = plot_contour(Felec, Pobj, Tmfb2, PobjE(2), FmfbHM(2), 'off', 'ContHiD', 'long
    strcat({'D = '}, num2str(D(2))), graphs2files, FigDir);
bg_LoHiD = plot_bg(FmfbHM(1), FmfbHM(2), PobjE(1), PobjE(2), 'off', 'bg_LoHiD',...
    graphs2files, FigDir);
bg_root ='bg_LoHiD';
quad_D_plot = quad_subplot(ContLoD, ContHiD, bg_LoHiD, 'quad_LoHiD', bg_root, ...
    graphs2files, FigDir);
if show_graphics
    quad_D_plot.Visible = 'on';
end
```

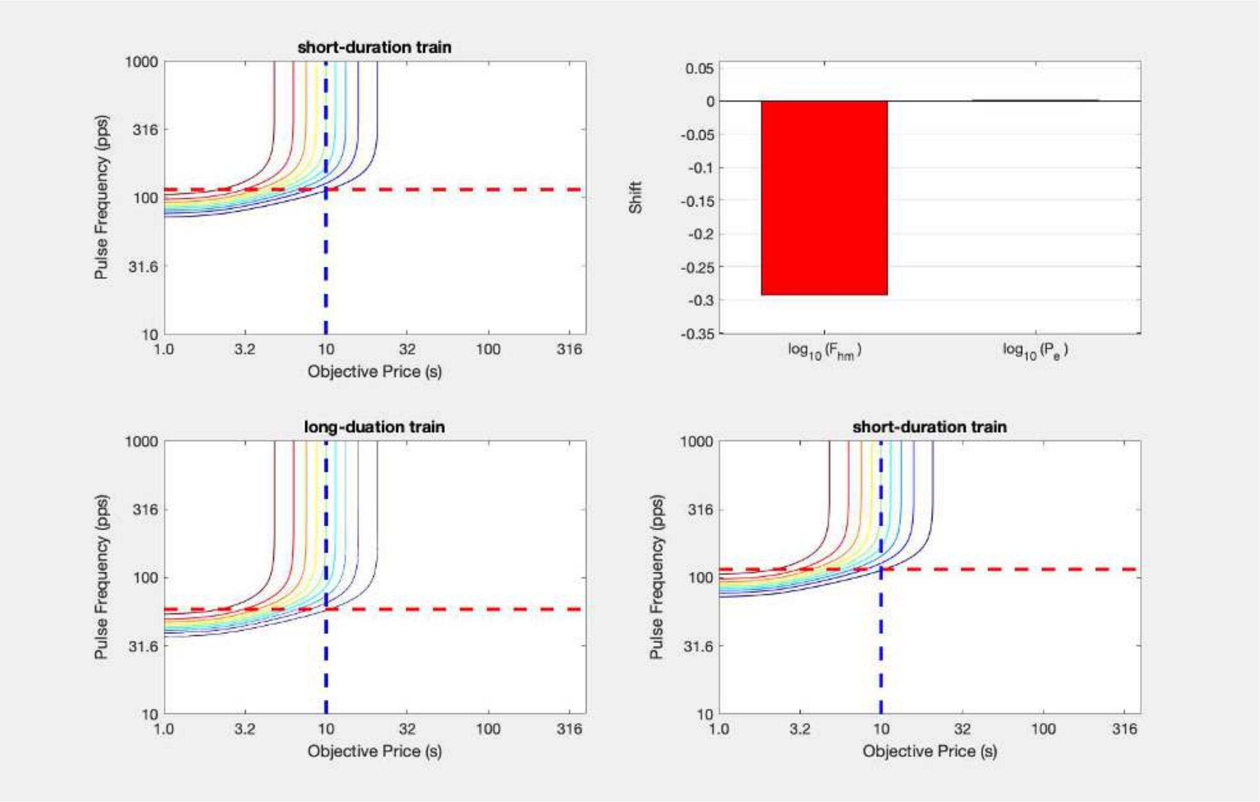


```
[fig_num, fig_tab] = add_fig(fig_num, fig_tab, "LoHiD_quad", ...
    "Effect of changing the train duration on the position of the reward mountain");
```

```
This is figure #11: (LoHiD_quad)
```

```
clearvars('-except',keepVars{:})
keepVars = who; % Restore cell array containing names of variables to be retained
toc
```

```
Elapsed time is 13.785165 seconds.
```

```
tic;
close all;
```

Arvanitogiannis and Shizgal (2008) assessed the effect of increasing the train duration from 0.25 to 1.00 s in four rats. The data in all four cases correspond to the prediction: there were statistically reliable shifts of the reward mountain along the pulse-frequency axis but not along the price axis. The effect of increasing the train duration was tested in an additional six rats by Breton et al. (2014). In all six cases, the mountain shifted as predicted along the pulse-frequency axis, and in three of these cases, no reliable shifts along the price axis were observed. In the remaining three cases, increasing the train duration did produce rightward shifts along the price axis. These were again hypothesized to due to the recruitment of reward-related neurons projecting to separate integrators. The convergence model describe below offers a new interpretations of the rightward shifts.

```
if show_graphics
    show_imported_graphic('TD_quad_Y14.png', 25, ImpFigDir);
```

```
end
```

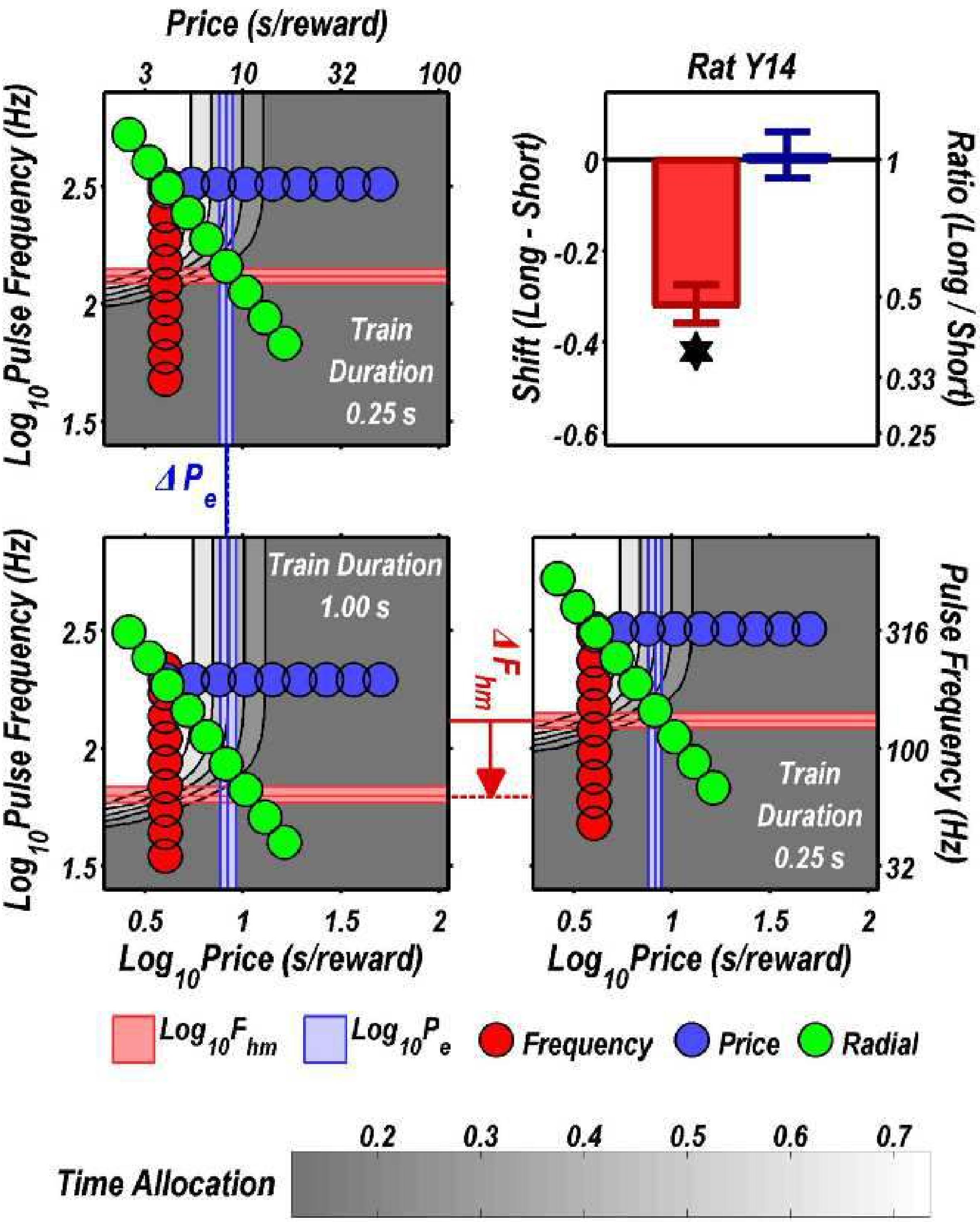


```
[fig_num, fig_tab] = add_fig(fig_num, fig_tab, "LoHiD_quad_Y14", ...
    "Rat Y14: Effect of changing the train duration on the position of the reward mount
```

```
This is figure #12: (LoHiD_quad_Y14)
```

A figure from Breton et al. (2014) showing single-subject data is reproducted above. Note the strong similarity between the simulated results in

```
disp(string({strcat({'Figure '},num2str(fig_tab.Number(fig_tab.Name=='LoHiD_quad')))}))
```

```
Figure 11
```

and the empirical results in

```
disp(string({strcat({'Figure '},num2str(fig_tab.Number(fig_tab.Name=='LoHiD_quad_Y14'))
```

```
Figure 12
```

```
toc
```

```
Elapsed time is 1.508525 seconds.
```

Effect of varying reward probability

```
tic;
close all;
```

Whereas changing the current or train duration is predicted to shift the reward mountain along the pulse-frequency axis but not along the price axis, changing the probability of delivering a reward upon satisfaction of the response requirement is predicted to produce an orthogonal shift: the mounain should move along the price axis but not along the pulse-frequency axis. This can be seen readily by inspection of

```
disp(string({strcat({'Equation '},num2str(eqn_tab.Number(eqn_tab.Name=='Pobj_e')))}));
```

```
Equation
```

,

```
disp(string({strcat({'Equation '},num2str(eqn_tab.Number(eqn_tab.Name=='StrDurFunTr')))
```

```
Equation
```

and

```
disp(string({strcat({'Equation '},num2str(eqn_tab.Number(eqn_tab.Name=='FmfbHM')))}));
```

```
Equation
```

. Intuitively, changing the reward probability should have no effect on the pulse-frequency required to produce a reward of half-maximal intensity, but is should rescale the payoff produced by this reward. Following the change in reward probability, the reward produced by a given pulse frequency is as intense as it was previously, but the ability of this pulse train to compete with alternate sources of reward will depend on the likelihood that the rat gets paid for the work it performs to obtain the electrical stimulation.

We will simulate the effect of changing the reward probability, first from 1.0 to 0.75 and then from 1.0 to 0.5:

```
C = 0.473; % median from Sonnenschein et al., 2003
D = 0.5; % typical train duration
```

```
logFelecBend = 1.3222; % from Solomon et al., 2015
FelecBend = 10^logFelecBend;
logFelecRO = 2.5587; % from Solomon et al., 2015
FelecRO = 10^logFelecRO;
FPmax =1000; % for determining RbsrNormMax. This value is sufficiently above FelecRO to
N = 126;
D = 0.5; % train duration
RhoPi = 5000; % arbitrary
% RhoPi/N is roughly equal to 50, which is roughly consistent with Sonnenchein et al.,

FmfbHM = FpulseHMfun(C, D, FelecBend, FPmax, FelecRO, N, RhoPi)
```

```
FmfbHM = 77.2222
```

```
% n.b., FmfbHM1 = FmfbHM2

dotPhiObj = 1;
Kaa = 1;
Kec = 1;
Krg = 1;
dotRaa = 0.1;
pObj = [1, 0.75];
PsubBend = 0.5;
PsubMin = 1.82;

g = 5;
RnormMax = fRbsrNorm(FPmax, FelecBend, FmfbHM, FelecRO, g)
```

```
RnormMax = 0.9996
```

```
PobjE = PobjEfun(dotPhiObj, Kaa, Kec, Krg, dotRaa, pObj, PsubBend, PsubMin, RnormMax)
```

```
PobjE = 1×2
    9.9956    7.4967
```

```
a = 3;
Felec = logspace(0,3,121)'; % column variable
Pobj = logspace(0,3,121); % row variable
Tmfb1 = TAfun(a, Felec, FelecBend, FmfbHM, FelecRO, g, Pobj, ...
    PobjE(1), PsubBend, PsubMin, RnormMax);
Tmfb2 = TAfun(a, Felec, FelecBend, FmfbHM, FelecRO, g, Pobj, ...
    PobjE(2), PsubBend, PsubMin, RnormMax);

MTNp1 = plot_MTN(Felec, Pobj, Tmfb1, 'off', 'MTNp1', 'p = 1.0', ...
    graphs2files, FigDir);
MTNp0p75 = plot_MTN(Felec, Pobj, Tmfb2, 'off', 'MTNp0p75', 'p = 0.75', ...
    graphs2files, FigDir);
dual_p0p75_plot = dual_subplot(MTNp1, MTNp0p75, 'MTNp1vsp0p75',...
    graphs2files,FigDir);
if show_graphics
    dual_p0p75_plot.Visible = 'on';
end
```

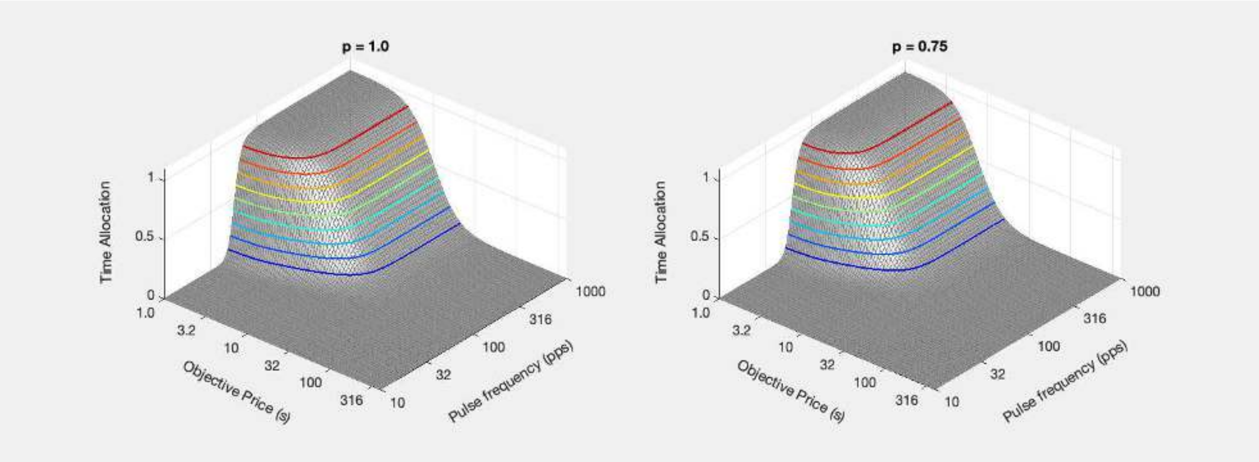


```
[fig_num, fig_tab] = add_fig(fig_num, fig_tab, "p1vsp0p75_mtns", ...
    "Effect of changing the reward probability on the reward mountain");
```

```
This is figure #13: (p1vsp0p75_mtns)
```

```
Contp1 = plot_contour(Felec, Pobj, Tmfb1, PobjE(1), FmfbHM, 'off', 'Contp1', 'p = 1.0',
    strcat({'p = '}, num2str(pObj(1))), graphs2files, FigDir);
Contp0p75 = plot_contour(Felec, Pobj, Tmfb2, PobjE(2), FmfbHM, 'off', 'Contp0p75', 'p =
    strcat({'p = '}, num2str(pObj(2))), graphs2files, FigDir);
bg_p1vsp0p75 = plot_bg(FmfbHM, FmfbHM, PobjE(1), PobjE(2), 'off', 'p1vs0p75_bg',...
    graphs2files, FigDir, -0.375, 0.075);
bg_root ='bg_p1vsp0p75';
quad_p0p75_plot = quad_subplot(Contp1, Contp0p75, bg_p1vsp0p75, 'quad_p1vsp0p75', bg_ro
    graphs2files, FigDir);
if show_graphics
    quad_p0p75_plot.Visible = 'on';
end
```

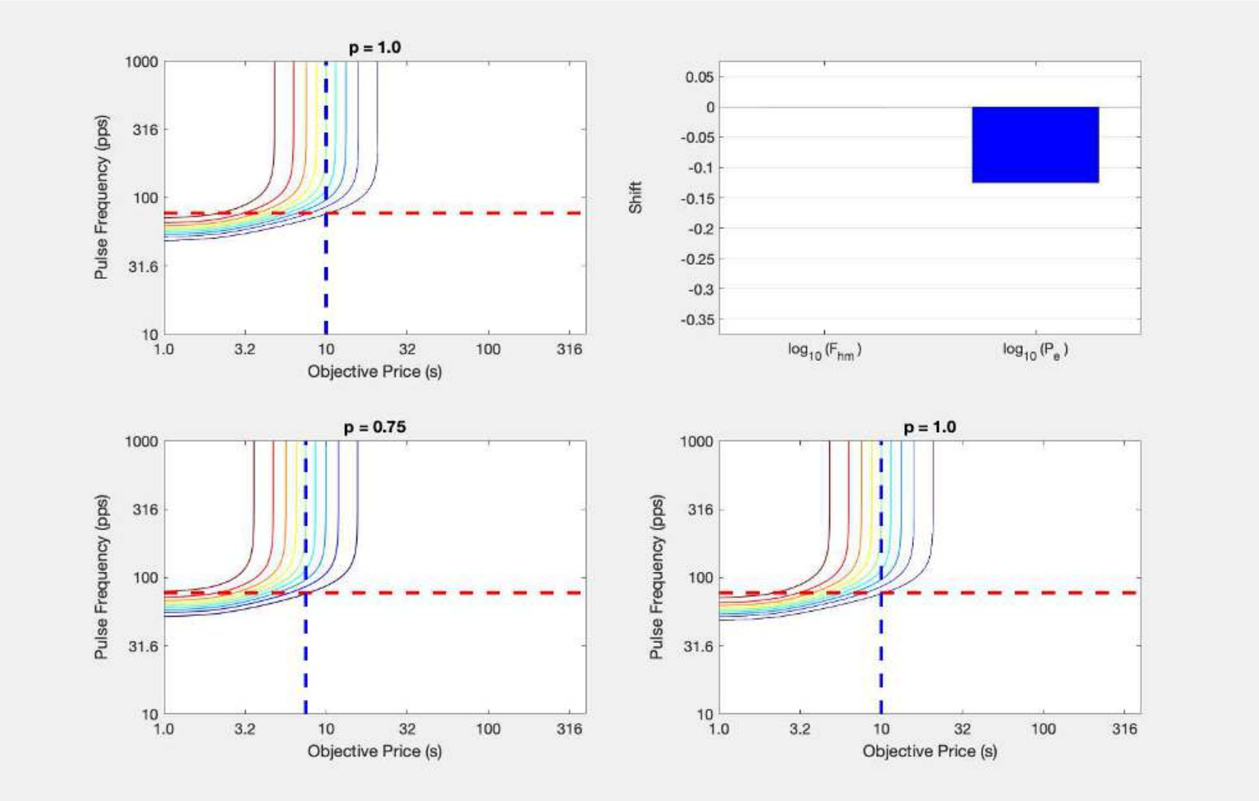


```
[fig_num, fig_tab] = add_fig(fig_num, fig_tab, "quad_p1vsp0p75", ...
    "Effect of changing the reward probability on the reward mountain");
```

This is figure #14: (quad_p1vsp0p75)

```
clear -regexp ^bg ^Cont ^dual ^MTN ^quad; % Clear only restricted set of variables here
toc
```

Elapsed time is 9.850066 seconds.

```
tic;
close all;
```

```
pObj = [1, 0.5];

PobjE = PobjEfun(dotPhiObj, Kaa, Kec, Krg, dotRaa, pObj, PsubBend, PsubMin, RnormMax)
```

```
PobjE = 1×2
    9.9956    4.9969
```

```
a = 3;
Felec = logspace(0,3,121)'; % column variable
Pobj = logspace(0,3,121); % row variable
Tmfb1 = TAfun(a, Felec, FelecBend, FmfbHM, FelecRO, g, Pobj, ...
    PobjE(1), PsubBend, PsubMin, RnormMax);
Tmfb2 = TAfun(a, Felec, FelecBend, FmfbHM, FelecRO, g, Pobj, ...
    PobjE(2), PsubBend, PsubMin, RnormMax);
```

```
MTNp1 = plot_MTN(Felec, Pobj, Tmfb1, 'off', 'MTNp1', 'p = 1.0', ...
    graphs2files, FigDir);
MTNp0p5 = plot_MTN(Felec, Pobj, Tmfb2, 'off', 'MTNp0p5', 'p = 0.5', ...
    graphs2files, FigDir);
dual_p0p5_plot = dual_subplot(MTNp1, MTNp0p5, 'MTNp1vsp0p5',...
    graphs2files,FigDir);
if show_graphics
    dual_p0p5_plot.Visible = 'on';
end
```

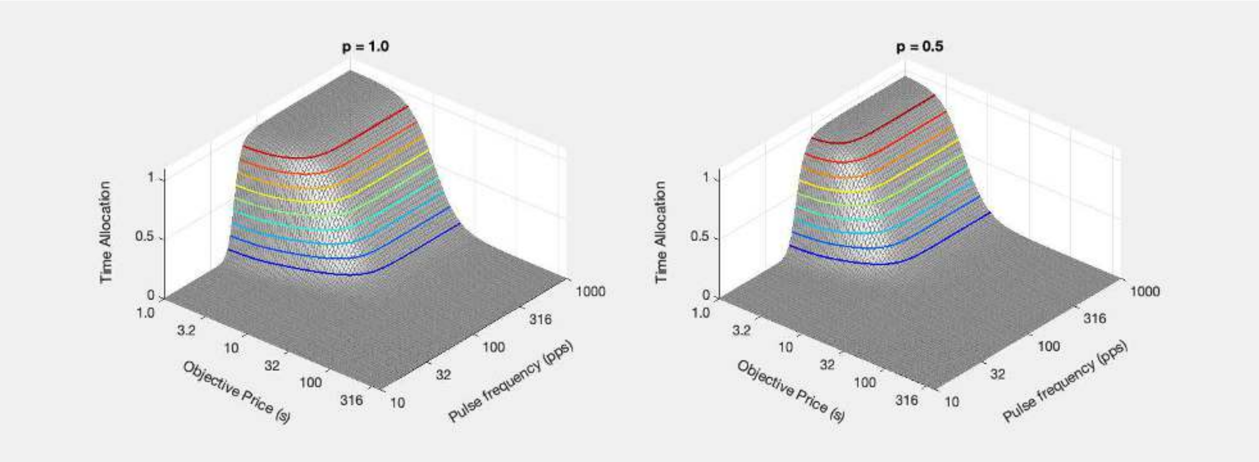


```
[fig_num, fig_tab] = add_fig(fig_num, fig_tab, "p1vsp0p5_mtns", ...
    "Effect of changing the reward probability on the reward mountain");
```

```
This is figure #15: (p1vsp0p5_mtns)
```

```
Contp1 = plot_contour(Felec, Pobj, Tmfb1, PobjE(1), FmfbHM, 'off', 'Contp1', 'p = 1.0',
    strcat({'p = '}, num2str(pObj(1))), graphs2files, FigDir);
Contp0p5 = plot_contour(Felec, Pobj, Tmfb2, PobjE(2), FmfbHM, 'off', 'Contp0p5', 'p = (
    strcat({'p = '}, num2str(pObj(2))), graphs2files, FigDir);
bg_p1vsp0p5 = plot_bg(FmfbHM, FmfbHM, PobjE(1), PobjE(2), 'off', 'p1vsp0p5_bg',...
    graphs2files, FigDir, -0.375, 0.075);
[fig_num, fig_tab] = add_fig(fig_num, fig_tab, "p1vsp0p5_quad", ...
    "Effect of changing the reward probability on the reward mountain");
```

```
This is figure #16: (p1vsp0p5_quad)
```

```
bg_root ='bg_p1vsp0p5';
quad_p0p5_plot = quad_subplot(Contp1, Contp0p5, bg_p1vsp0p5, 'quad_p1vsp0p5', bg_root,
    graphs2files, FigDir);
if show_graphics
    quad_p0p5_plot.Visible = 'on';
end
```

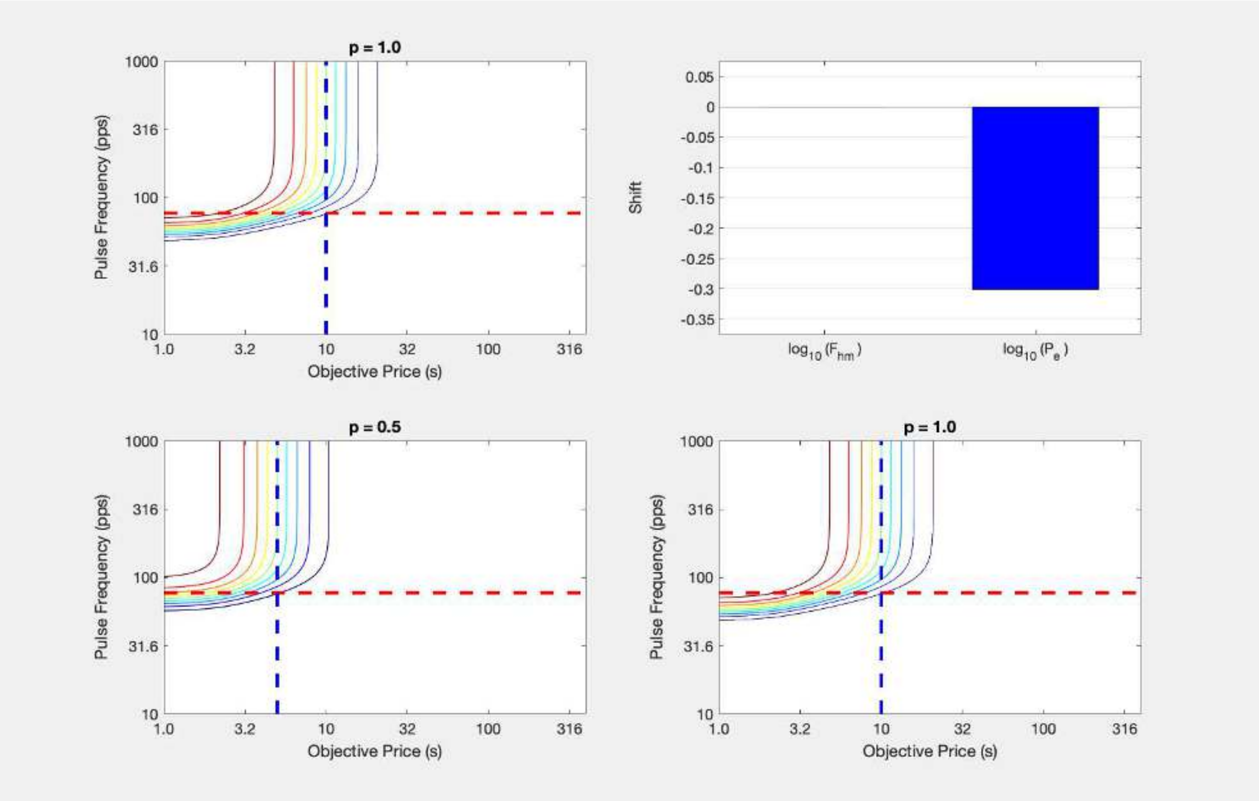


```
[fig_num, fig_tab] = add_fig(fig_num, fig_tab, "quad_p1vsp0p5", ...
    "Effect of changing the reward probability on the reward mountain");
```

```
This is figure #17: (quad_p1vsp0p5)
```

```
clearvars('-except',keepVars{:})
keepVars = who; % Restore cell array containing names of variables to be retained
toc
```

```
Elapsed time is 8.880286 seconds.
```

```
tic;
close all;
```

Breton, Conover and Shizgal (2014) tested the effect of decreasing the reward probability from 1.00 to 0.75. The reward mountains obtained from all 10 rats shifted along the price axis as predicted. There was no statistically reliable shift along the pulse-frequency axis in the position of the mountains obtained from six of these rats. In the remaining four cases, the shifts along the pulse-frequency axis were small in comparison to the shifts along the price axis and were inconsistent in direction. Decreasing the probability of reward further to 0.5, increased the size of the shifts along the price axis in all seven rats tested. In five cases, there were no shifts observed along the pulse-frequency axis, and in the two cases in which such shifts were detected, they were inconsistent in direction. Data from one subject showing the effect of reducing the reward probability to 0.5 are shown below

```
if show_graphics
    show_imported_graphic('prob_shifts_PD8.png',25,ImpFigDir);
```

```
end
```

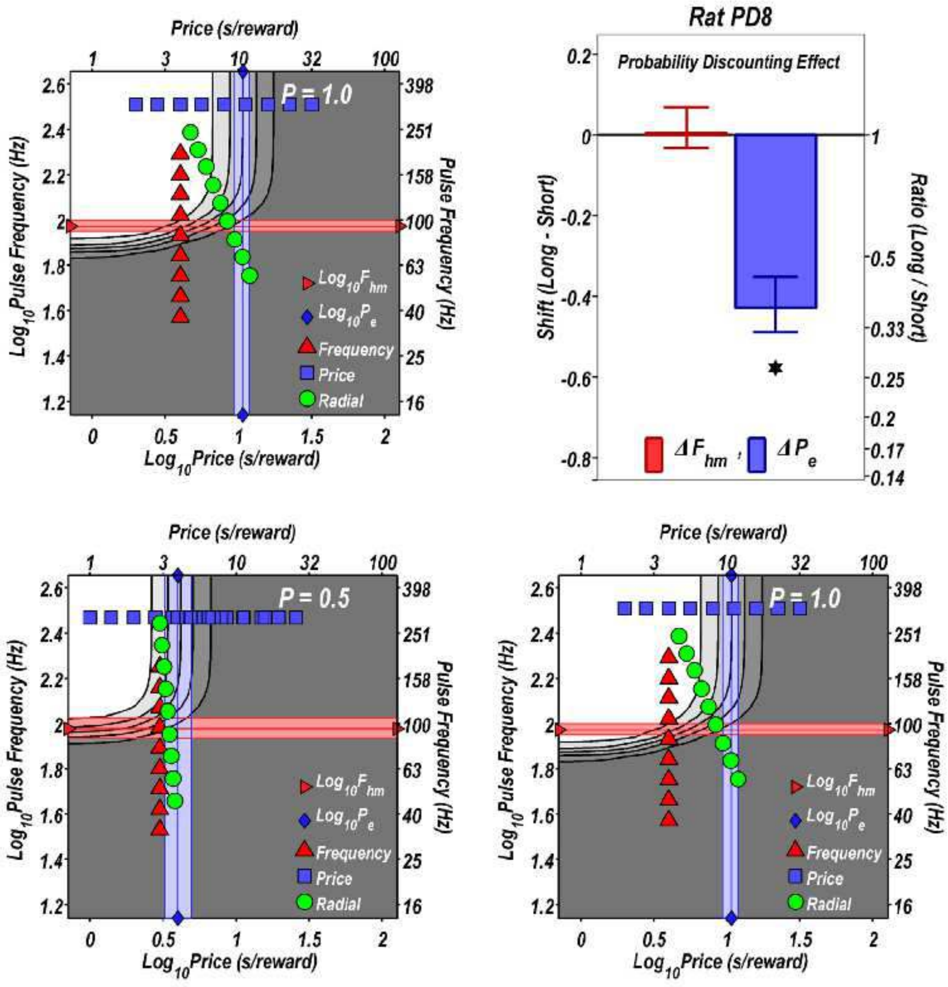


```
[fig_num, fig_tab] = add_fig(fig_num, fig_tab, "p1vsp0p5_quad_PD8", ...
    "Rat PD8: Effect of changing the reward probability on the reward mountain");
```

```
This is figure #18: (p1vsp0p5_quad_PD8)
```

followed by a summary of the entire dataset.

```
if show_graphics
    show_imported_graphic('prob_disc_all.png',30,ImpFigDir);
end
```

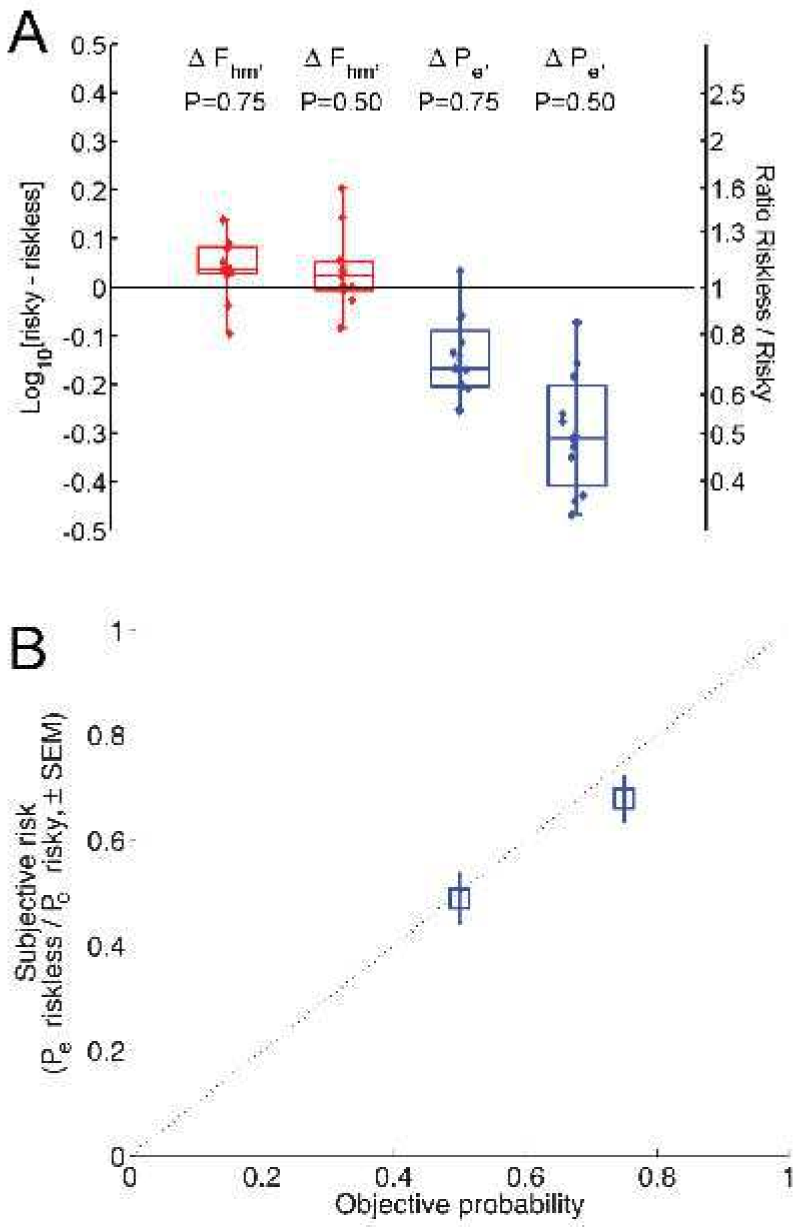


```
[fig_num, fig_tab] = add_fig(fig_num, fig_tab, "ProbDiscSummary", ...
    "Summary of the effects of changing the reward probability on the reward mountain")
```

```
This is figure #19: (ProbDiscSummary)
```

The upper panel of the summary shows that changing reward probability moves the mountain almost exclusively along the price axis and does so in a graded manner; the larger the change in reward probability, the larger the shift along the price axis. On the basis of

```
disp(string({strcat({'Equation '},num2str(eqn_tab.Number(eqn_tab.Name=='PayoffElec')))}
```

```
Equation
```

we can estimate the subjective reward probability from the observed shift along the price axis. The lower panel of the summary shows that the subjective reward probabilities estimated in this manner are all but indistinguishable from the objective reward probabilities.

```
toc
```

```
Elapsed time is 1.608664 seconds.
```

The significance of orthogonal shifts

```
tic;
close all;
```

The validation experiments just described show conclusively that the reward mountain can be displaced along either the pulse-frequency or price axes, as predicted by the underlying model. In most cases, these displacements are orthogonal: the mountain shifts either along one axis or the other.

These observations have important implications for the form of the reward-growth function at the heart of the mountain model. In order for shifts along more than one axis to be possible, the form of the reward-growth function must distinguish changes that rescale its input and output. The logistic form implied by the matching data from the Gallistel lab instantiates such a distinction. Changes in the value of the position parameter, $[F_{pulse_{hm}}(d_{Elec}, D_{elec}, I_{elec})]$, rescale the input, thereby shifting the reward-growth function along the pulse-frequency axis, whereas changes in the value of the maximum attainable reward, (K_{rg}), rescale the output, thereby shifting the maximum value of the reward-growth function vertically. In the figures below, we change the value of the input-rescaling position parameter $[F_{pulse_{hm}}(d_{Elec}, D_{elec}, I_{elec})]$ by varying the current (thereby changing *N*, the number of reward-generating neurons recruited), and we rescale the output of the reward-growth function by changing the value of the maximum attainable reward, (K_{rg}).

```
logFelecBend = 1.3222; % from Solomon et al., 2015
FelecBend = 10^logFelecBend;
logFelecRO = 2.5587; % from Solomon et al., 2015
FelecRO = 10^logFelecRO;

numF = 121; % Number of pulse frequencies in Felec vector
Felec = logspace(0,3,numF);
FelecMat = repmat(logspace(0,3,numF),3,1); % add two rows in preparation for graphing

C = median([0.453,0.503, 0.268, 0.224, 0.493, 0.64]); % from Sonnenschein et al., 2003
D = 0.5;
RhoPi = (5000 / fHfiring(C, D, 100, 5000)) * 100; % To position the logFhm values near

gElec = 5;

NnLG1 = 10^1.8; % The number of elements in the parameter vector must equal the # of ro
NnLG2 = 10^2;
NnLG3 = 10^2.2;
NnLGvec = [NnLG1;NnLG2;NnLG3];
NnLGmat = repmat(NnLGvec,1,numF); % Store the N values in a matrix of the same size as

KrgLG1 = 10^-0.2; % The number of elements in the parameter vector must equal the # of
KrgLG2 = 10^0;
KrgLG3 = 10^0.2;
KrgLGvec = [KrgLG1;KrgLG2;KrgLG3];
```

```
KrgLGmat = repmat(KrgLGvec,1,numF); % Store the Krg values in a matrix of the same size

FhmLG1 = fHfiring(C, D, NnLG1, RhoPi); % The number of elements in the parameter vector
FhmLG2 = fHfiring(C, D, NnLG2, RhoPi);
FhmLG3 = fHfiring(C, D, NnLG3, RhoPi);
FhmLGvec = [FhmLG1;FhmLG2;FhmLG3];
FhmLGmat = repmat(FhmLGvec,1,numF); % Store the Fhm values in a matrix of the same size

RelecLGmat_Nn = fRbsrFull(C,D,FelecMat,FelecBend,FelecRO,gElec,KrgLG2,NnLGmat,RhoPi);
RelecLGmat_Krg = fRbsrFull(C,D,FelecMat,FelecBend,FelecRO,gElec,KrgLGmat,NnLG2,RhoPi);
[fun_num, fun_tab] = add_fun(fun_num, fun_tab, "fRbsrFull", ...
    "C, D, F, Fbend, Fro, g, Krg, N, RhoPi, varargin",...
    "Full reward-growth function for BSR");
```

This is function #34: (fRbsrFull)

```
TitleStrSemiLog = 'logistic growth';
TitleStrLogLog = 'logistic growth';

pnam = "F_{hm}";
fnam = "Fhm";
% The data to be plotted must be in columns. Thus, FelecMat and RelecMat are transposed
RG_NnLG_semilog = plot_RG(FelecMat',RelecLGmat_Nn',pnam,FhmLGvec,fnam,TitleStrSemiLog,'
RG_NnLG_loglog = plot_RG(FelecMat',RelecLGmat_Nn',pnam,FhmLGvec,fnam,TitleStrSemiLog,'l

pnam = "K_{rg}";
fnam = "Krg";
RG_KrgLG_semilog = plot_RG(FelecMat',RelecLGmat_Krg',pnam,KrgLGvec,fnam,TitleStrSemiLog
RG_KrgLG_loglog = plot_RG(FelecMat',RelecLGmat_Krg',pnam,KrgLGvec,fnam,TitleStrSemiLog,

dual_subplot(RG_NnLG_loglog, RG_KrgLG_loglog, 'RG_NnLG_KrgLG_loglog',...
    graphs2files,FigDir);
if show_graphics
    RG_NnLG_KrgLG_loglog.Visible = 'on';
end
[fig_num, fig_tab] = add_fig(fig_num, fig_tab, "LogisticRGLL", ...
    "When reward intensity grows as a logistic function of pulse frequency, shifts are
```

This is figure #20: (LogisticRGLL)

```
RG_NnLG_KrgLG_semilog = dual_subplot(RG_NnLG_semilog, RG_KrgLG_semilog, 'RG_NnLG_KrgLG_
    graphs2files,FigDir);
if show_graphics
    RG_NnLG_KrgLG_semilog.Visible = 'on';
end
```

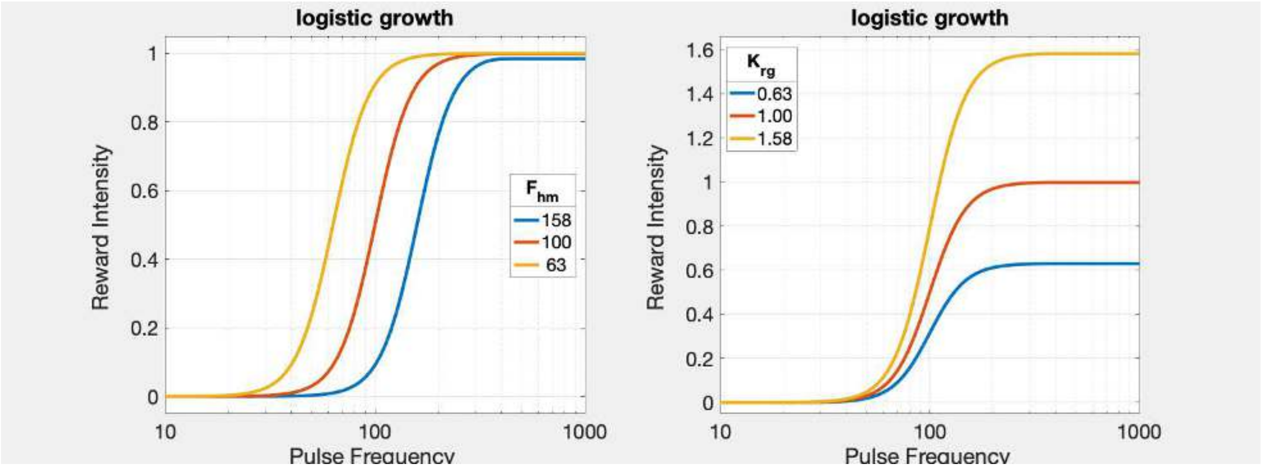


```
[fig_num, fig_tab] = add_fig(fig_num, fig_tab, "LogisticRGSL", ...
    "When reward intensity grows as a logistic function of pulse frequency, shifts are
```

```
This is figure #21: (LogisticRGSL)
```

The horizontal shifts of the reward-growth function are translated into shifts of the reward mountain along the pulse-frequency axis, whereas vertical shifts of the reward-growth function are translated in shifts of the reward mountain along the price axis. Above, we have illustrated such orthogonal shifts by simulating the effects of changing the current or train duration, as shown in

```
disp(string({strcat({'Figure '},num2str(fig_tab.Number(fig_tab.Name=='LoHiI_quad'))))}))
```

```
Figure
```

```
disp(string({strcat({'Figure '},num2str(fig_tab.Number(fig_tab.Name=='LoHiD_quad'))))}))
```

```
Figure 11
```

```
disp(string({strcat({'Figure '},num2str(fig_tab.Number(fig_tab.Name=='LoHiD_quad_Y14'))
```

```
Figure 12
```

and the effects of changing the reward probability, as shown in

```
disp(string({strcat({'Figure '},num2str(fig_tab.Number(fig_tab.Name=='p1vsp0p75_quad'))
```

```
Figure
```

```
disp(string({strcat({'Figure '},num2str(fig_tab.Number(fig_tab.Name=='p1vsp0p5_quad')))
```

```
Figure 16
```

```
disp(string({strcat({'Figure '},num2str(fig_tab.Number(fig_tab.Name=='p1vsp0p5_quad_PD8
```

```
Figure 18
```

What would happen if frequency-following fidelity remained the same, but the input-scaling and output-scaling parameters of the reward-growth function were no longer independent? We can simulate such a case by replacing the logistic reward-growth function with a power function:

$$\frac{R}{K_{out}} = \left(\frac{F_{firing}}{K_{in}}\right)^{g}$$

where

K_{in} = the input-scaling parameter

K_{out} = the output-scaling parameter

```
[eqn_num, eqn_tab] = ...
    add_eqn(eqn_num, eqn_tab, "RGpg", "Power growth of reward intensity");
```

```
This is equation #27: (RGpg)
```

```
[fun_num, fun_tab] = add_fun(fun_num, fun_tab, "fRpg", "F, Fbend, Fro, g, Kin, Krg",...
    "Function to compute power growth of reward");
```

```
This is function #35: (fRpg)
```

This power-growth function can be rewritten as

$$\frac{R}{K_{out} \times K_{in}^{-g}} = F_{firing}^{g}$$

```
[eqn_num, eqn_tab] = ...
    add_eqn(eqn_num, eqn_tab, "RGpgReformat", "Power growth of reward intensity");
```

```
This is equation #28: (RGpgReformat)
```

In contrast to the case of logistic growth, the two scaling constants act jointly, in an inseparable manner, and exclusively on the output of the function. Changing either scaling constant shifts the power-growth function along the y axis but does not change its position along the x axis. As in the case of the logistic reward-growth function, *g* determines the steepness of reward growth (the slope on double-logarithmic coordinates). Although K_{out} appears to scale the output, whereas K_{in} appears to scale the induced firing frequency,

```
disp(string({strcat({'Equation '},num2str(eqn_tab.Number(eqn_tab.Name=='RGpgReformat'))
```

```
Equation 28
```

shows that the two constants function as one. Thus, reward intensity will grow as a power function of the electrical pulse frequency until the induced firing frequency in the stimulation reward-related neurons asymptotes. Changing the current displaces the ***logistic*** reward-growth function laterally, a shift that is orthogonal to the one produced by changing the scale parameter, K_{rg}. In contrast, changing the current displaces the ***power*** reward-growth function vertically, the same direction as the shift produced by changing the scale parameter. This is shown graphically below.

In the following simulations, changing the value of *N* serves as a proxy for changes in current. The K_{in} parameter is inversely proportional to the number of stimulated neurons (*N*). In the figure legends, the value of the K_{in} parameter is normalized to the K_{in} value corresponding to the middle value of *N*.

```
logFelecBend = 1.3222;
FelecBend = 10^logFelecBend;
logFelecRO = 2.5587;
```

```
FelecRO = 10^logFelecRO;
FPmax = 1000;

numF = 121; % Number of pulse frequencies in Felec vector
Felec = logspace(0,3,numF);
FelecMat = repmat(logspace(0,3,numF),3,1); % add two rows in preparation for graphing

gElec = 2.5;

NnPG1 = 10^(2-(0.3/gElec)); % The number of elements in the parameter vector must equal
NnPG2 = 10^2;
NnPG3 = 10^(2+(0.3/gElec));
% NnPGvec = [NnPG1;NnPG2;NnPG3];
% NnPGmat = repmat(NnPGvec,1,numF); % Store the N values in a matrix of the same size a

FFaggNorm = FilterFun(FPmax,FelecBend, FelecRO) * NnPG2; % Normalization factor
% When Kout = 1 and NnPG = NnPG2, FFaggNorm ensures that R = 1 when FF = FFmax
KinPGnorm = FFaggNorm/NnPG2;
KinPG1 = FFaggNorm/NnPG1;
KinPG2 = FFaggNorm/NnPG2;
KinPG3 = FFaggNorm/NnPG3;
KinPGvec = [KinPG1;KinPG2;KinPG3];
KinPGmat = repmat(KinPGvec,1,numF); % Store the Kin values in a matrix of the same size

% The number of elements in the parameter vector must equal the # of rows in FelecMat
KoutPG1 = 10^-0.3;
KoutPG2 = 10^0;
KoutPG3 = 10^0.3; % RpgMax = 10^0.15 = 1.4125
KoutPGvec = [KoutPG1;KoutPG2;KoutPG3];
KoutPGmat = repmat(KoutPGvec,1,numF); % Store the Kout values in a matrix of the same s


RelecPGmat_Kin = fRpg(FelecMat,FelecBend,FelecRO,gElec,KinPGmat,KoutPG2);
RelecPGmat_Kout = fRpg(FelecMat,FelecBend,FelecRO,gElec,KinPG2,KoutPGmat);

TitleStrSemiLog = 'power growth';
TitleStrLogLog = 'power growth';

pnam = "K_{in} / K_{in_{norm}}";
fnam = "Kin";
KinLgndVec = KinPGvec ./ KinPGnorm;

RG_KinPG_semilog = plot_RG(FelecMat',RelecPGmat_Kin',pnam,KinLgndVec,fnam,TitleStrSemiL
RG_KinPG_loglog = plot_RG(FelecMat',RelecPGmat_Kin',pnam,KinLgndVec,fnam,TitleStrLogLog
pnam = "K_{out}";
fnam = "Kout";
RG_KoutPG_semilog = plot_RG(FelecMat',RelecPGmat_Kout',pnam,KoutPGvec,fnam,TitleStrSemi
RG_KoutPG_loglog = plot_RG(FelecMat',RelecPGmat_Kout',pnam,KoutPGvec,fnam,TitleStrLogLo

RG_KinPG_KoutPG_semilog = dual_subplot(RG_KinPG_semilog, RG_KoutPG_semilog, 'RG_KinPG_K
    graphs2files,FigDir);
if show_graphics
    RG_KinPG_KoutPG_semilog.Visible = 'on';
end
```

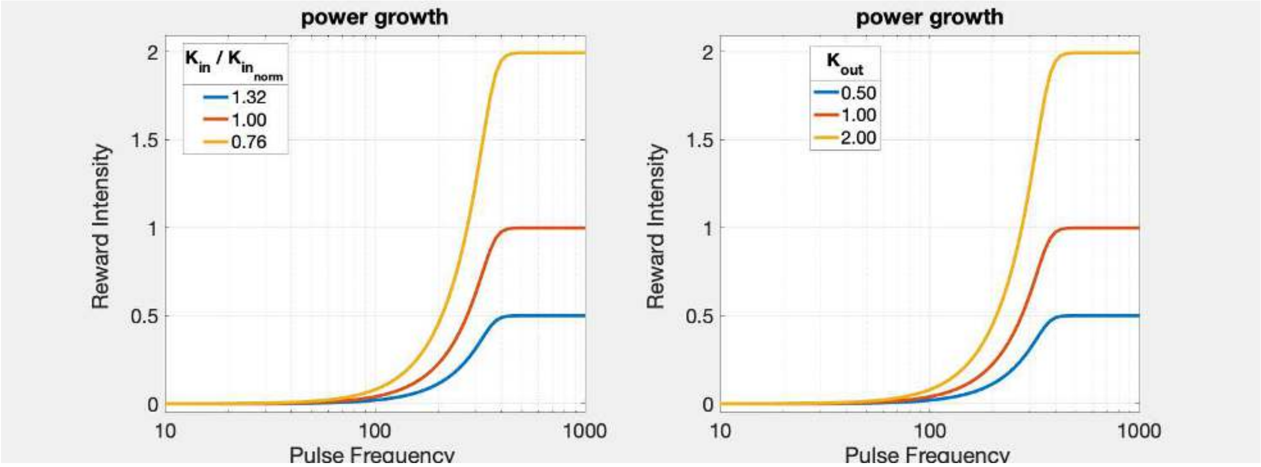


```
[fig_num, fig_tab] = add_fig(fig_num, fig_tab, "PowerRGSL", ...
    "When reward intensity grows as a power function, all shifts are vertical");
```

```
This is figure #22: (PowerRGSL)
```

```
dual_subplot(RG_KinPG_loglog, RG_KoutPG_loglog, 'RG_KinPG_KoutPG_loglog',...
    graphs2files,FigDir);
if show_graphics
    RG_KinPG_KoutPG_loglog.Visible = 'on';
end
%shg
[fig_num, fig_tab] = add_fig(fig_num, fig_tab, "PowerRGLL", ...
    "When reward intensity grows as a power function, all shifts are vertical");
```

```
This is figure #23: (PowerRGLL)
```

```
disp(string({strcat({'Equation '},num2str(eqn_tab.Number(eqn_tab.Name=='RGpg')))}));
```

```
Equation 27
```

Unlike the case with logistic growth of reward intensity, the variable that scales the input to the power reward-growth function (K_{in}) and the variable that scales its output (K_{out}) are no longer independent and cannot move the reward-growth curve in orthogonal directions. As a result, the reward mountain can shift only along a single axis when the underlying reward-growth function lacks independent input- and output-scaling parameters. This is demonstated in the following section.

```
clearvars('-except',keepVars{:})
keepVars = who; % Restore cell array containing names of variables to be retained
toc
```

```
Elapsed time is 15.340981 seconds.
```

```
tic;
close all;
```

The reward mountain can shift only along the price axis when the underlying reward-growth function lacks indepedent input- and output-scaling parameters

```
dotPhiObj = 1;
Kaa = 1;
Kec = 1;
dotRaa = 0.1;
pObj = 1;

a = 3;
Felec = logspace(0,3,121)'; % column variable
Pobj = logspace(0,3,121); % row variable

logFelecBend = 1.3222;
FelecBend = 10^logFelecBend;
logFelecRO = 2.5587;
FelecRO = 10^logFelecRO;

gElec = 2.5;
N = 100;
PsubBend = 0.5;
PsubMin = 1.82;

FPmax = 1000;
FFmax = FilterFun(FPmax,FelecBend, FelecRO);

Kin = [FFmax, FFmax / 2^(1/gElec)];
Kout = [1,2];

RmaxBase = fRpg(1000,FelecBend,FelecRO,gElec,Kin(1),Kout(1));
RmaxKin = fRpg(1000,FelecBend,FelecRO,gElec,Kin,Kout(1));
RmaxKout = fRpg(1000,FelecBend,FelecRO,gElec,Kin(1),Kout);

PsubEpgBase = PsubEpgFun(dotPhiObj, Kaa, Kec, Kout, dotRaa, pObj, RmaxBase);
PsubEpgKin = PsubEpgFun(dotPhiObj, Kaa, Kec, Kout, dotRaa, pObj, RmaxKin);
PsubEpgKout = PsubEpgFun(dotPhiObj, Kaa, Kec, Kout, dotRaa, pObj, RmaxKout);
[fun_num, fun_tab] = add_fun(fun_num, fun_tab, "PsubEpgFun", ...
    "dotPhiObj, Kaa, Kec, Krg, ObjAA, pObj, Rmax",...
    "Function to compute PsubE for power reward growth");
```

This is function #36: (PsubEpgFun)

```
TpgBase = TApgFun(a, Felec, FelecBend, FelecRO, gElec, Kin(1), Kout(1), Pobj, PsubBend,
TpgKin2 = TApgFun(a, Felec, FelecBend, FelecRO, gElec, Kin(2), Kout(1), Pobj, PsubBend,
TpgKout2 = TApgFun(a, Felec, FelecBend, FelecRO, gElec, Kin(1), Kout(2), Pobj, PsubBenc
[fun_num, fun_tab] = add_fun(fun_num, fun_tab, "TApgFun", ...
    "a, Felec, FelecBend, FelecRO, gElec, Kin, Kout, N, Pobj, PsubBend, PsubEpgBase, Ps
    "Time allocation in response to power growth of reward intensity");
```

This is function #37: (TApgFun)

```
xmin = 0;
xmax = 2.3;
ymin = 1.5;
ymax = 3;
```

```
MTNpgKin1 = plot_MTN(Felec, Pobj, TpgBase, 'off', 'MTNpgKin1', 'Kin = Kin1', ...
    graphs2files, FigDir, xmin, xmax, ymin, ymax);
MTNpgKin2 = plot_MTN(Felec, Pobj, TpgKin2, 'off', 'MTNpgKin2', strcat('Kin1','\div','2.
    graphs2files, FigDir, xmin, xmax, ymin, ymax);
dual_pgKin_plot = dual_subplot(MTNpgKin1, MTNpgKin2, 'MTNpgKin1Kin2',...
    graphs2files,FigDir);
if show_graphics
    dual_pgKin_plot.Visible = 'on';
end
[fig_num, fig_tab] = add_fig(fig_num, fig_tab, "PG_K1_1vsK1_2_mtns", ...
    "Effect of changing the power-growth input-scaling parameter");
```

```
This is figure #24: (PG_K1_1vsK1_2_mtns)
```

```
MTNpgKout1 = plot_MTN(Felec, Pobj, TpgBase, 'off', 'MTNpgKout1', 'Kout = Kout1', ...
    graphs2files, FigDir, xmin, xmax, ymin, ymax);
MTNpgKout2 = plot_MTN(Felec, Pobj, TpgKout2, 'off', 'MTNpgKout2', 'Kout = 2*Kout1', ...
    graphs2files, FigDir, xmin, xmax, ymin, ymax);
```

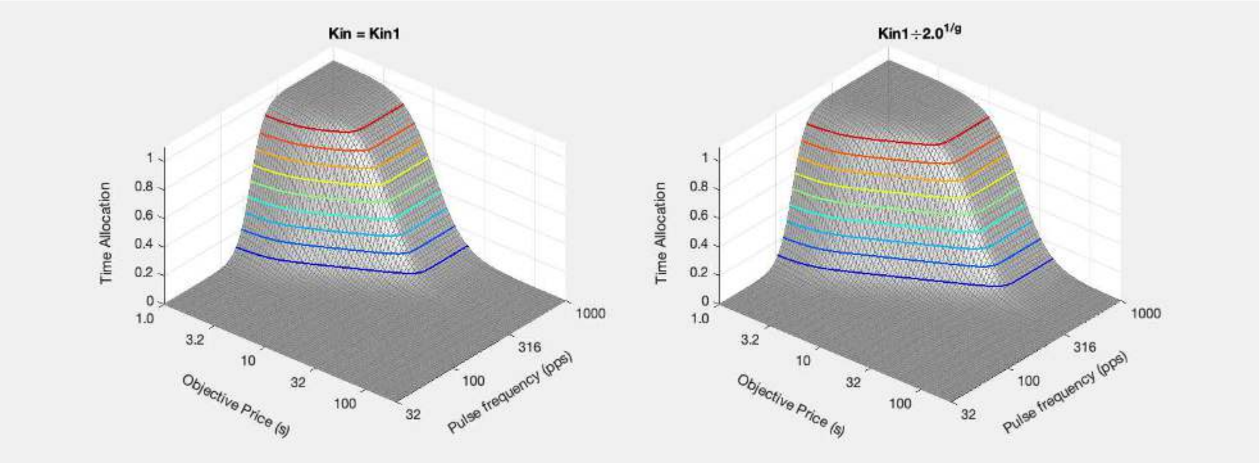


```
dual_pgKout_plot = dual_subplot(MTNpgKout1, MTNpgKout2, 'MTNpgKout1Kout2',...
    graphs2files,FigDir);
if show_graphics
    dual_pgKout_plot.Visible = 'on';
end
```

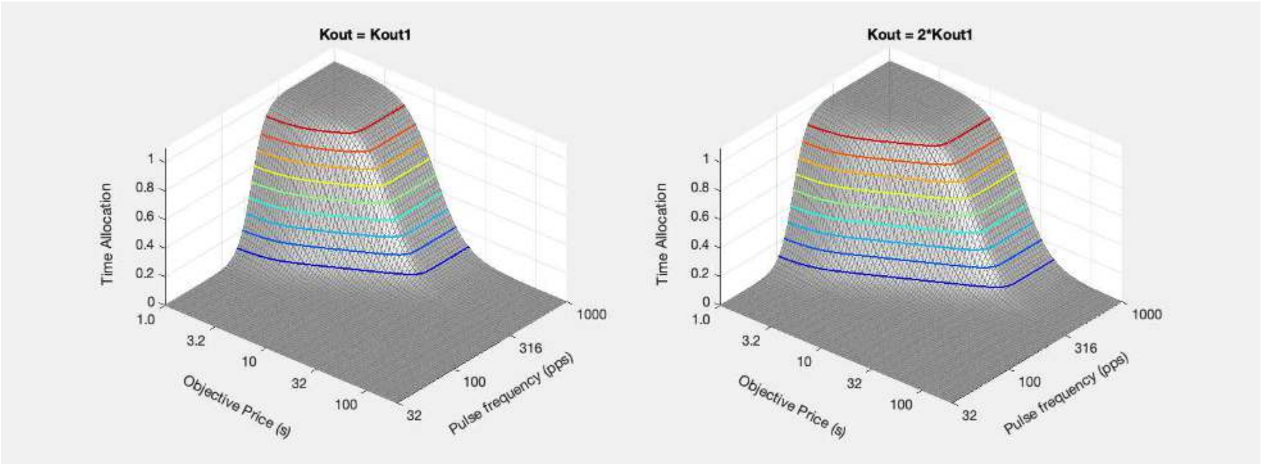


```
[fig_num, fig_tab] = add_fig(fig_num, fig_tab, "PG_Kout1vsKout2_mtns", ...
    "Effect of changing the power-growth output-scaling parameter");
```

This is figure #25: (PG_Kout1vsKout2_mtns)

```
FhmPGKin = find_FhmPG(FPmax, FelecBend, FelecRO, gElec, Kin, Kout(1), RmaxKin);
FhmPGKout = find_FhmPG(FPmax, FelecBend, FelecRO, gElec, Kin(1), Kout, RmaxKout);
[fun_num, fun_tab] = add_fun(fun_num, fun_tab, "find_FhmPG", ...
    "FPmax, Fbend, Fro, g, Kin, Kout, RmaxPG",...
    "Function to compute the Fhm value for power reward growth");
```

This is function #38: (find_FhmPG)

```
PobjEpgBase = PsubBsFun(PsubEpgBase, PsubBend, PsubMin);
PobjEpgKin = PsubBsFun(PsubEpgKin, PsubBend, PsubMin);
PobjEpgKout = PsubBsFun(PsubEpgKout, PsubBend, PsubMin);

ContKin1 = plot_contour(Felec, Pobj, TpgBase, PobjEpgKin(1), FhmPGKin(1), 'off', 'Cont
    strcat({'Kin = '}, num2str(round(Kin(1),2))), graphs2files, FigDir,...
    xmin, xmax, ymin, ymax);
ContKin2 = plot_contour(Felec, Pobj, TpgKin2, PobjEpgKin(2), FhmPGKin(2), 'off', 'Cont
    strcat({'Kin = '}, num2str(round(Kin(2),2))), graphs2files, FigDir,...
    xmin, xmax, ymin, ymax);
bg_Kin1vskKin2 = plot_bg(FhmPGKin(1), FhmPGKin(2), PobjEpgKin(1), PobjEpgKin(2), 'off',
    graphs2files, FigDir);
bg_root = 'bg_Kin1vsKin2';
quad_Kin1vsKin2 = quad_subplot(ContKin1, ContKin2, bg_Kin1vskKin2, 'quad_Kin1vsKin2', b
    graphs2files, FigDir);
if show_graphics
    quad_Kin1vsKin2.Visible = 'on';
end
```

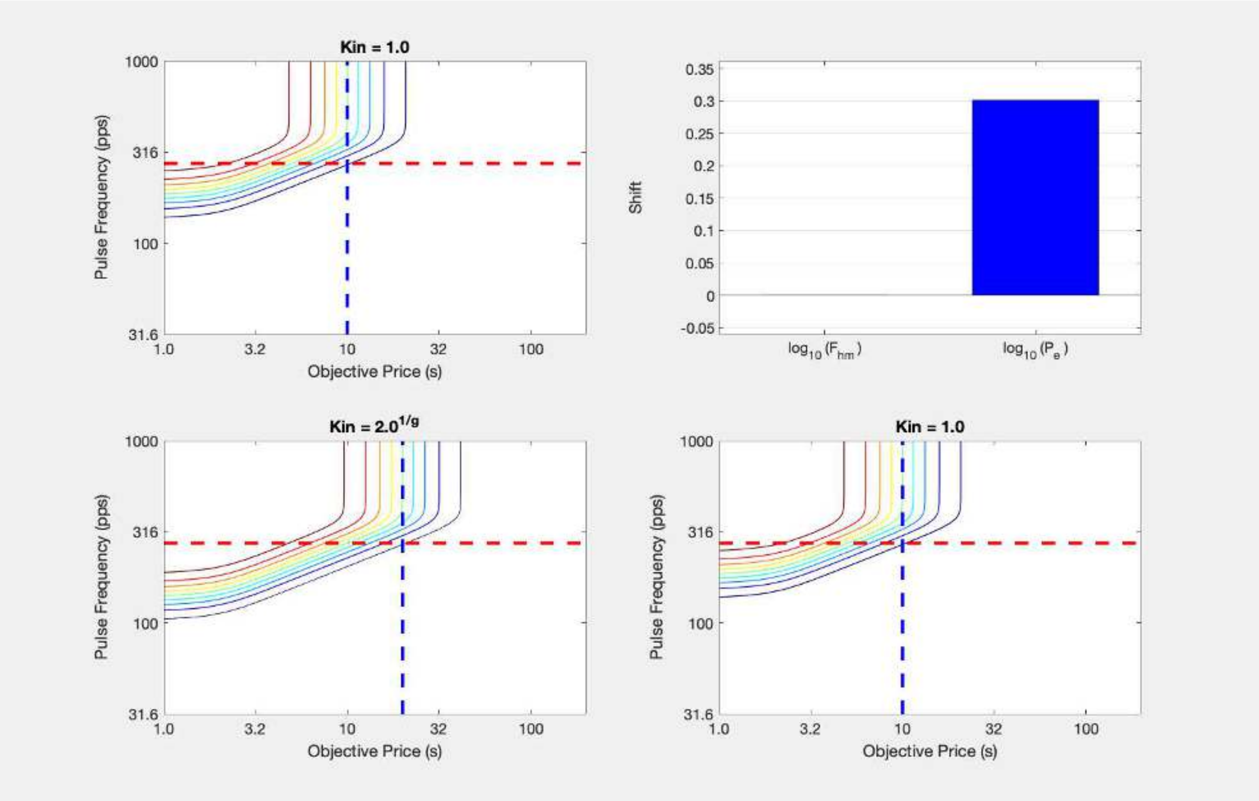


```
[fig_num, fig_tab] = add_fig(fig_num, fig_tab, "quad_Kin1vsKin2", "Effect of increasing
```

This is figure #26: (quad_Kin1vsKin2)

```
ContKout1 = plot_contour(Felec, Pobj, TpgBase, PobjEpgKout(1), FhmPGKout(1), 'off', 'Co
    strcat({'Kout = '}, {'Kout1'}), graphs2files, FigDir,...
    xmin, xmax, ymin, ymax);
ContKout2 = plot_contour(Felec, Pobj, TpgKout2, PobjEpgKout(2), FhmPGKout(2), 'off', 'C
    strcat({'Kout = '}, {'2 * Kout1'}), graphs2files, FigDir,...
    xmin, xmax, ymin, ymax);
bg_Kout1vskKout2 = plot_bg(FhmPGKout(1), FhmPGKout(2), PobjEpgKout(1), PobjEpgKout(2),
    graphs2files, FigDir);
bg_root = 'bg_Kout1vsKout2';
quad_Kout1vsKout2 = quad_subplot(ContKout1, ContKout2, bg_Kout1vskKout2, 'quad_Kout1vsK
    graphs2files, FigDir);
if show_graphics
    quad_Kout1vsKout2.Visible = 'on';
end
```

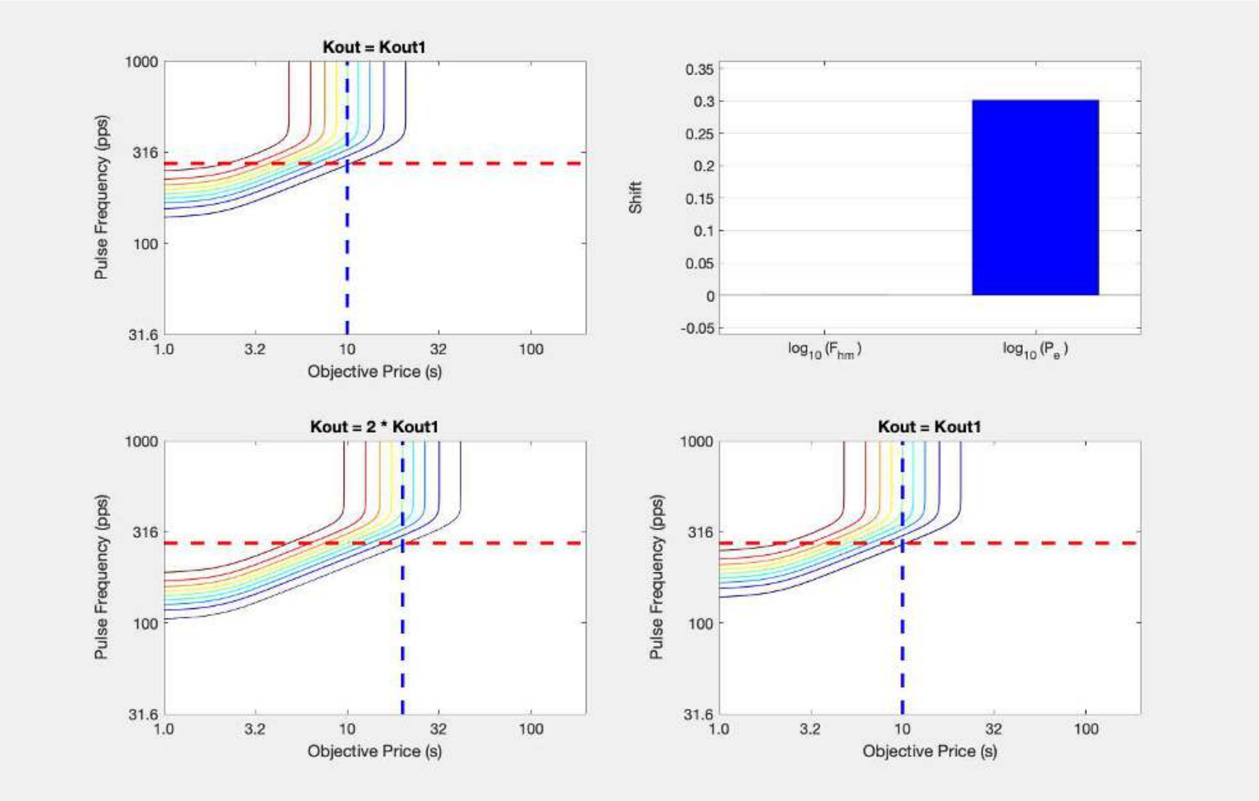


```
[fig_num, fig_tab] = add_fig(fig_num, fig_tab, "quad_Kout1vsKout2", "Effect of increasi
```

```
This is figure #27: (quad_Kout1vsKout2)
```

The graphs shown above demonstrate that changes to the input- and output-scaling parameters of the power-growth function produce identical shifts in the position of the reward mountain. Changing the value of either parameter shifts the mountain rightward along the price axis, in sharp contrast to the orthogonal shifts observed when reward intensity grows as a logistic function of pulse frequency.

In the case of eICSS, we know from the experiments of Gallistel's group that reward intensity grows in a manner similar to a logistic function of pulse frequency. Unlike the case of power growth, logistic growth is characterized by independent parameters that scale the input and output. This characteristic is what causes the effect of varying reward probability to shift the reward mountain in a direction orthogonal to the effects of varying current or train duration. It follows that when such orthogonal shifts are observed, this requires that the underlying reward-growth function incorporates independent parameters that scale the input (the position parameter) and the output (the parameter representing the maximum reward intensity). Thus even in the absence of data from matching experiments or other methods for measuring the growth of reward intensity, we can make inferences about the reward-growth function by observing the way in which various manipulations shift the reward mountain. This will be important for the interpretation of the oICSS data.

```
clearvars('-except',keepVars{:})
keepVars = who; % Restore cell array containing names of variables to be retained
toc
```

```
Elapsed time is 18.280454 seconds.
```

At what stage of processing does perturbation of dopaminergic neurotransmission alter reward seeking in eICSS?

```
tic;
close all;
```

We have described changes in the position parameter $(F_{pulse_{hm}})$ as alterations in the ***sensitivity*** of the reward-growth function. One might best label $F_{pulse_{hm}}$ as the "inverse-sensitivity" parameter because the lower its value, the weaker the input required to drive reward intensity to a given level. In contrast, we have described changes in the output-scaling parameter (K_{rg}) as alterations in the ***gain*** of the reward-growth function. Just as turning the volume knob on an audio amplifier changes the loudness produced by weak and strong inputs by the same percentage, changing K_{rg} alters the reward intensity produced by both low and high pulse frequencies (within the frequency-following range) by the same percentage. Changes in sensitivity shift the reward mountain along the pulse-frequency axis, whereas changes in gain shift the mountain along the price axis.

Operant peformance in the eICSS paradigm is altered by perturbation of dopaminergic neurotransmission. Boosting dopaminergic signalling typically enhances performance, whereas attenuating dopaminergic signalling typically attenuates performance. These changes have long been attributed to the modulation of reward sensitivity by dopaminergic agents (See: Hernandez et al., 2010). If so, drugs that alter dopaminergic neurotransmission should shift the reward mountain along the pulse-frequency axis and not along the price axis. We have shown that this is not so. In rats working for electrical stimulation of the medial forebrain bundle, we demonstrated that enhancement of dopaminergic signalling by the highly specific reuptake blocker, GBR-12909, shifted the reward mountain rightward along the price axis in 8/10 rats without producing any statistically reliable shifts along the pulse-frequency axis (Hernandez et al., 2012). The D2/D4/5HT7 receptor blocker, pimozide, shifted the reward mountain leftward along the price axis in 5/6 rats without producing any statistically reliable shifts along the pulse-frequency axis (Trujillo-Pisanty, Conover & Shizgal, 2014). To account for these complementary effects, the changes in dopaminergic neurotransmission had to have altered one or more of the variables that determine the position of the reward mountain along the price axis, such as the output-scaling parameter of the reward-growth function (K_{rg}), the subjective effort cost ($\dot{\phi}_{sub_W}$), or the payoff from alternate activities (U_L).

```
if show_graphics
    show_imported_graphic('GBR_pimozide_summary.png', 100, ImpFigDir);
end
```

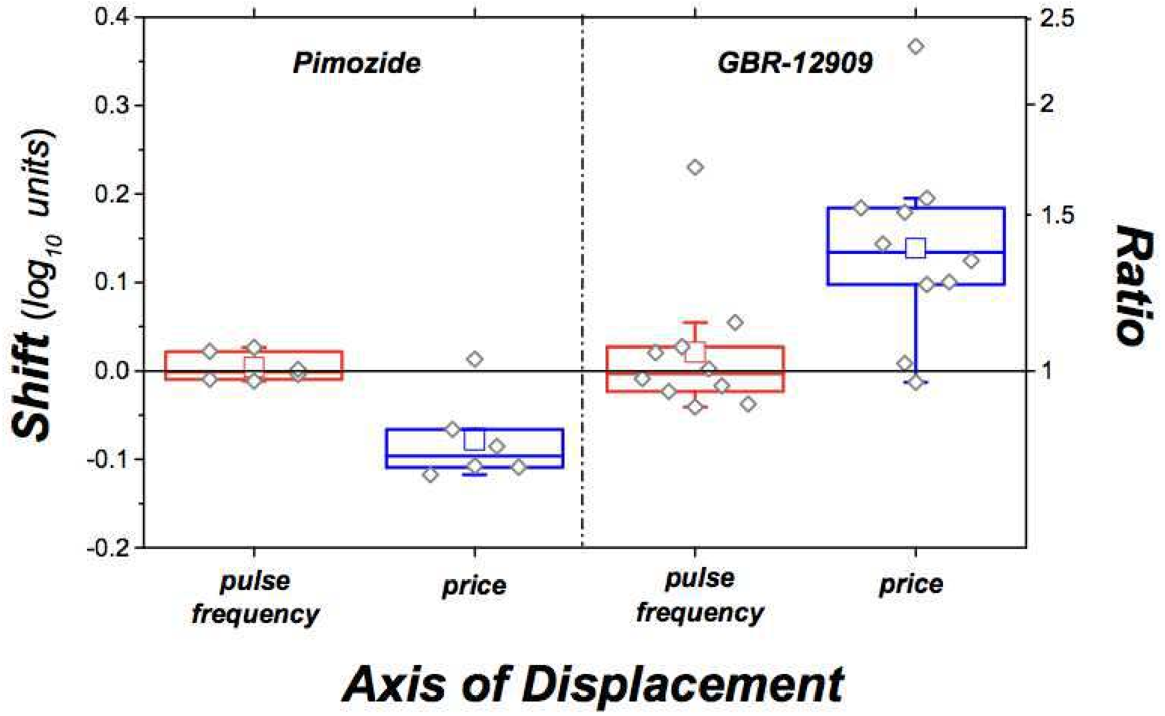


```
[fig_num, fig_tab] = add_fig(fig_num, fig_tab, "PimGBRshifts", ...
    "Changes in dopaminergic neurotransmission shift the mountain along the price axis'
```

```
This is figure #28: (PimGBRshifts)
```

An intuitively appealing interpretation of these data is based on the notion that the neurons subserving eICSS of the medial forebrain bundle project to midbrain dopamine neurons and that it is the transynaptic activation of the dopamine neurons that gives rise to the reward effect of the medial forebrain bundle stimulation (the "series" model of brain-reward circuitry). In the following section, we apply the reward mountain model to the oICSS data from the present paper. We show that the series model ***cannot*** account for the data from both the eICSS and oICSS studies. After demonstrating this, we discuss "convergence" models that show promise for an intergrated account of both eICSS and oICSS, and we propose experiments that put such accounts to empirical test.

```
toc
```

```
Elapsed time is 0.748551 seconds.
```

Optical intracranial self-stimulation of midbrain dopamine neurons

```
tic;
close all;
```

In the experiments reviewed above, rats worked to trigger trains of electrical current pulses applied to the medial forebrain bundle. In contrast, in the experiment we now discuss, rats worked to trigger trains of optical pulses delivered through an optical fiber positioned over the ventral tegmental area of the midbrain. As a result of viral transcription and Cre-Lox recombination, dopamine neurons with somata in, or adjacent to, the

VTA expressed channelrhodopsin-2 (ChR2) and thus could be excited by light delivered at an appropriate wavelength (473 nm). As others have reported previously (Witten et al., 2012), rats expressing ChR2 in midbrain dopamine neurons work vigorously to obtain such optical stimulation.

We show that TH-Cre rats expressing ChR2 in midbrain dopamine neurons learn to perform the cumulative hold-down task to receive optical stimulation of the ventral tegmental area (VTA), where the somata of dopamine neurons projecting to forebrain targets reside. As in the case of eICSS, time allocation varies smoothly as a function of the strength and price of the optical stimulation. The surface defined by the mountain model fits the data well.

```
if show_graphics
    show_imported_graphic('Bechr29_veh_mtn.png', 30, ImpFigDir);
end
```

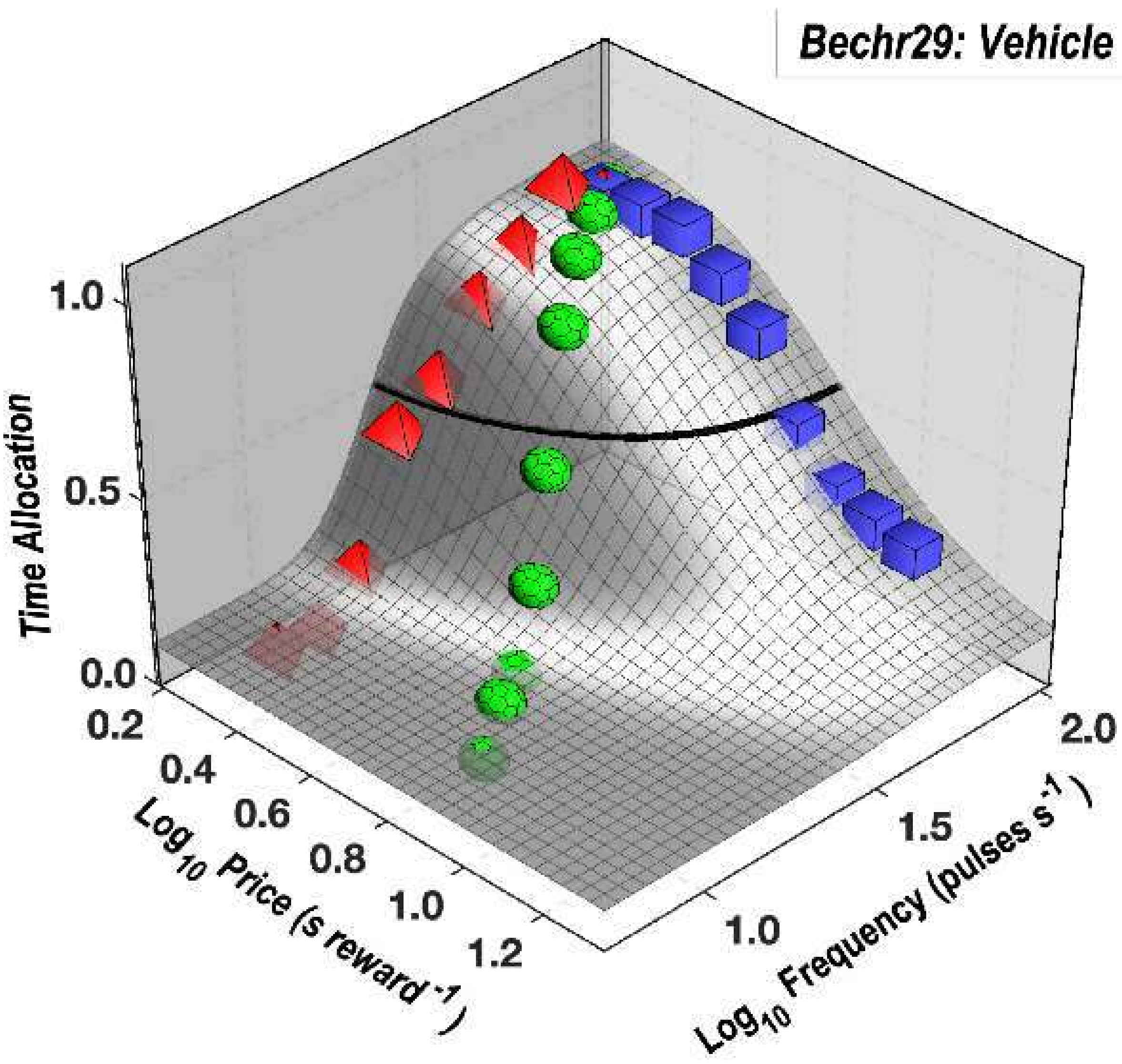


```
[fig_num, fig_tab] = add_fig(fig_num, fig_tab, "BeChR29vehMtn", ...
    "The mountain model fitted to vehicle-condition data from rat BeChR29");
```

```
This is figure #29: (BeChR29vehMtn)
```

The effect of the dopamine-transporter blocker, GBR-12909.

To boost dopaminergic neurotransmission, we administered 20 mg/kg of the dopamine-transporter blocker, GBR-12909. Under the influence of the drug, the mountain fitted to the data from rat BeChR29 shifted leftwards along the pulse-frequency axis and rightwards along the price axis.

```
if show_graphics
    show_imported_graphic('Bechr29_dual_mountains.png', 30, ImpFigDir);
end
```

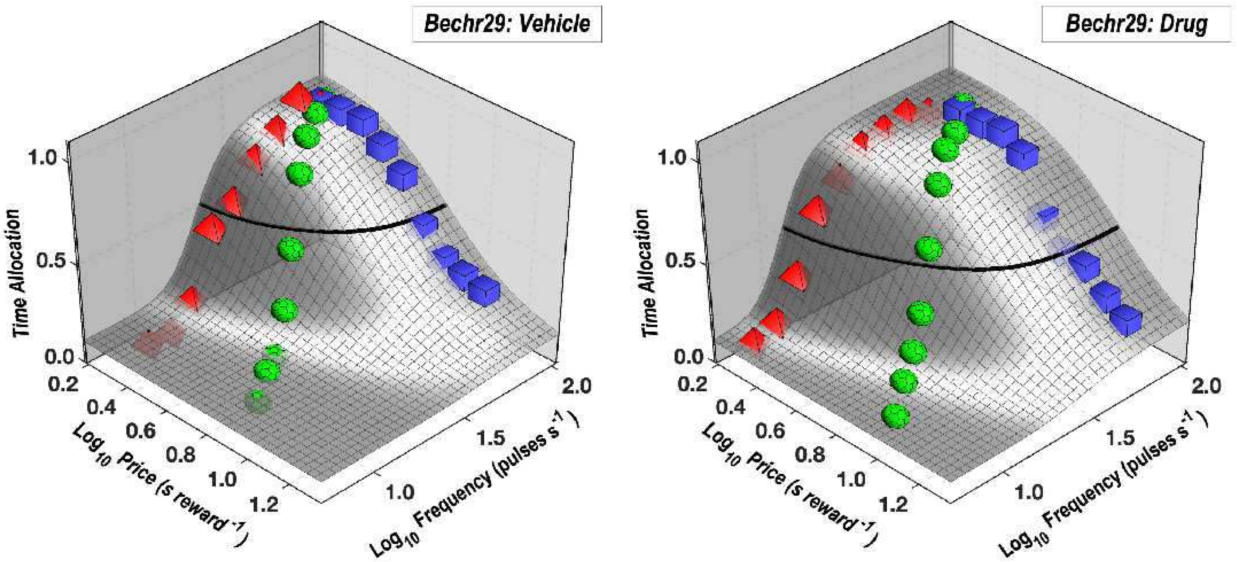


```
[fig_num, fig_tab] = add_fig(fig_num, fig_tab, "BeChR29vehdrgMtn", ...
    "Effect of GBR-12909 on the reward-mountain data obtained from rat BeChR29");
```

```
This is figure #30: (BeChR29vehdrgMtn)
```

These shifts can be most clearly discerned in the contour-plot display and bargraph summary:

```
if show_graphics
    show_imported_graphic('BeChR29_quad_GBR.png', 25, ImpFigDir);
end
```

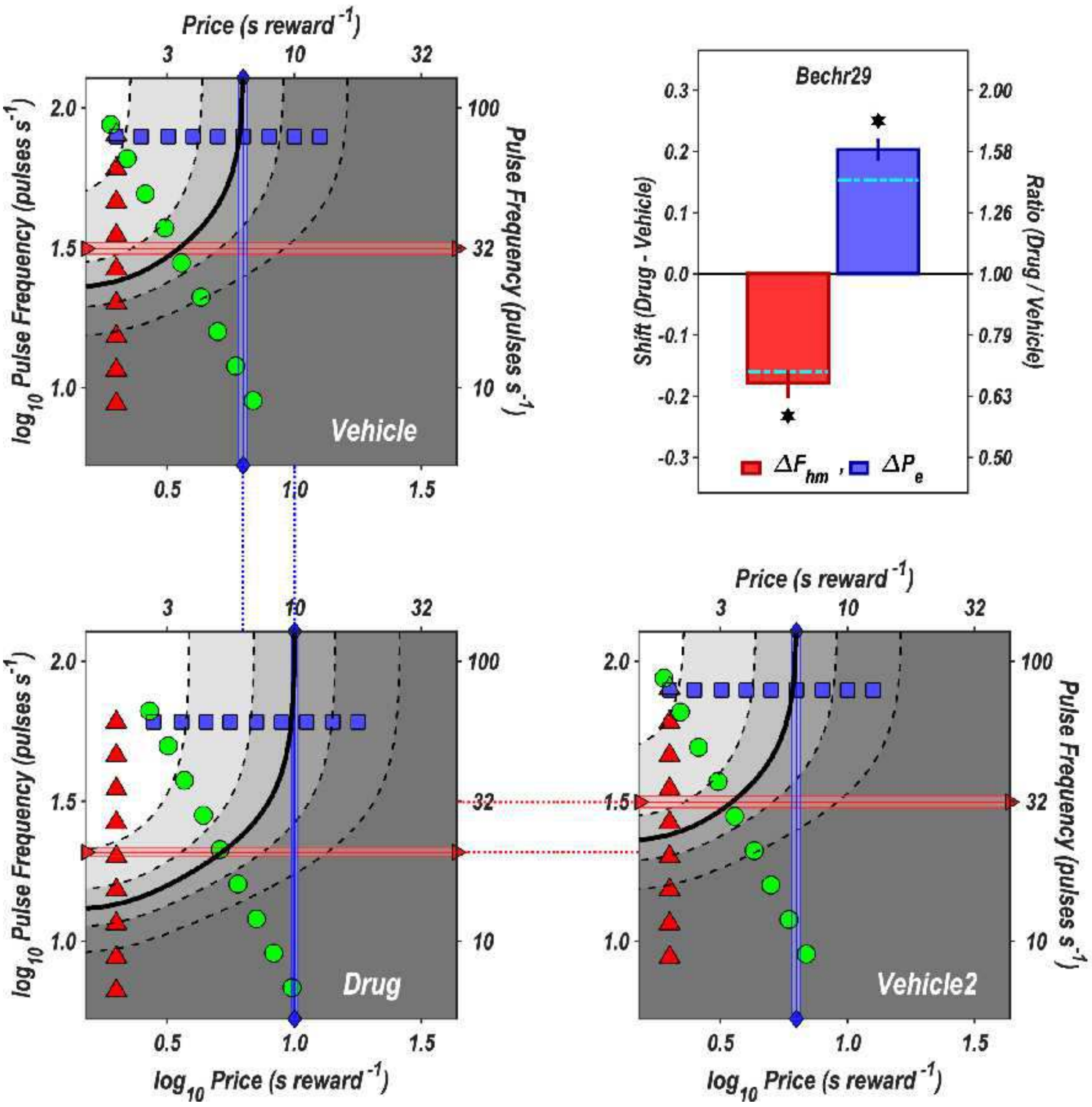


```
[fig_num, fig_tab] = add_fig(fig_num, fig_tab, "BeChR29quad", ...
    "Effect of GBR-12909 on the reward-mountain data obtained from rat BeChR29");
```

```
This is figure #31: (BeChR29quad)
```

This pattern of shifts is seen in the results from 6 of the 7 rats:

```
if show_graphics
    show_imported_graphic('optoGBR_shift_summary.png', 100, ImpFigDir);
end
```

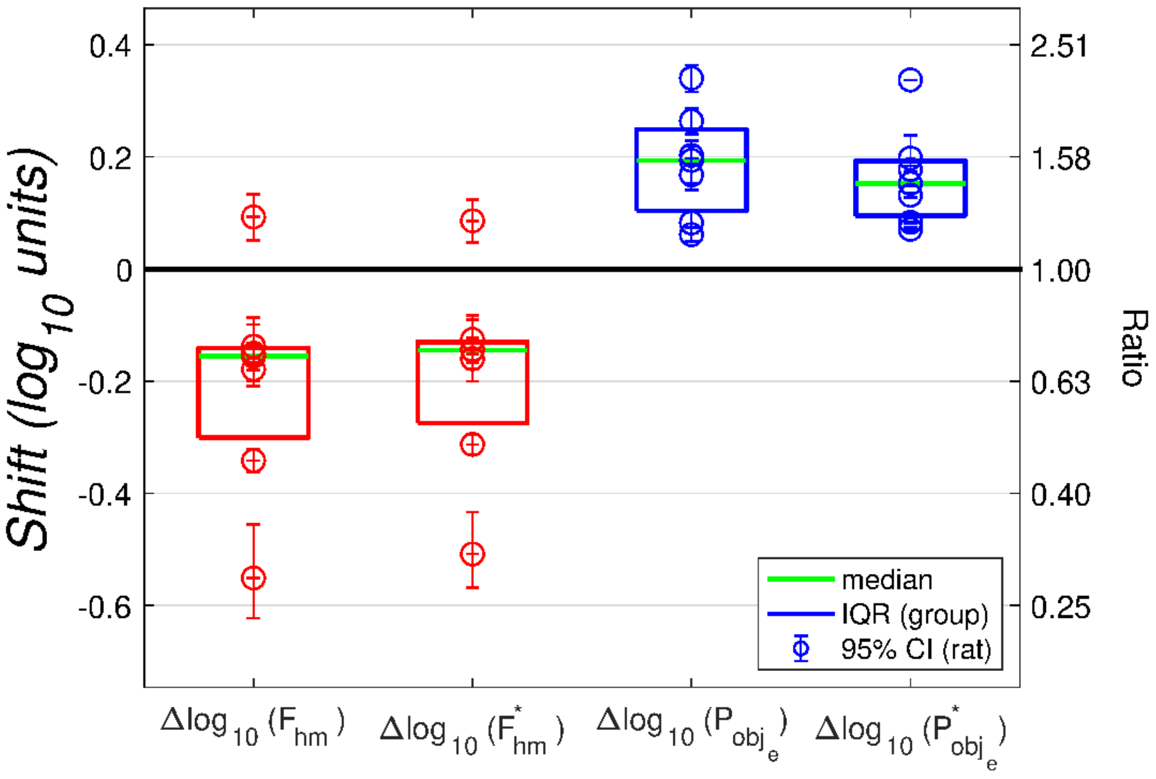


```
[fig_num, fig_tab] = add_fig(fig_num, fig_tab, "GBRshiftSummary", ...
    "Effect of GBR-12909 on the reward-mountain data obtained from all 7 rats");
```

```
This is figure #32: (GBRshiftSummary)
```

The error bars surrounding the data point for each rat represent the bootstrapped 95% confidence interval (CI) for the parameter in question, whereas the boxes denoted by the heavy solid lines represent the inter-quartile range (IQR) of the median parameter estimate for the group of seven rats. The $\Delta \log_{10}\left(F^*_{hm}\right)$ and $\Delta \log_{10}\left(P^*_{obj_e}\right)$ values have been corrected for differential frequency-following fidelity in the vehicle and drug conditions. (See below and main body of the manuscript.)

Modeling the results

We now undertake modeling to determine how the optical pulse train can be translated into the observed behavioral responses in a manner consistent with the observed effect of dopamine-transporter blockade on the reward mountain.

The first step is to relate the induced frequency of firing in the dopamine neurons to the optical pulse frequency. For this purpose, we adopt a filter function of the form defined by

```
disp(string({strcat({'Function '},num2str(fun_tab.Number(fun_tab.Name=='FilterFun')))}))
```

```
Function 6
```

, which provides an accurate description of how medial forebrain bundle fibers subserving electrical intracranial self-stimulation respond to the electrical pulse frequency. It is not known how well the functional form describes frequency-following in optically activated, ChR2-expressing, midbrain dopamine neurons. As explained in the main body of the manuscript, parameter values have been chosen on the basis of published electrophysiological, voltammetric, and behavioral data.

The maximum firing frequency in the dopamine neurons is far lower than in the directly stimulated neurons responsible for the rewarding effect of electrical medial forebrain bundle stimulation (Solomon et al., 2015). To reflect this in the equation for the frequency response, we used updated names and values for the parameters:

$$F_{da_{Rate}} = F_{da_{Bend}} \times \left(\ln \left[1 + e^{\frac{F_{da_{RO}}}{F_{da_{Bend}}}} \right] - \ln \left[1 + e^{\frac{F_{da_{RO}} - F_{opt}}{F_{da_{Bend}}}} \right] \right)$$

where

$F_{da_{Bend}}$ = parameter determining the abruptness of the roll-off in the frequency response of the dopamine neurons

F_{opt} = the optical pulse frequency

$F_{da_{Rate}}$ = optically induced firing rate of the dopamine neurons

$F_{da_{RO}}$ = the pulse frequency in the center of the roll-off region

```
[eqn_num, eqn_tab] = ...
    add_eqn(eqn_num, eqn_tab, "FreqFolDA", "Frequency of firing as a function of pulse
```

```
This is equation #29: (FreqFolDA)
```

```
[sym_num, sym_tab] = add_sym(sym_num, sym_tab, "FdaBend", "parameter determining sharpn
[sym_num, sym_tab] = add_sym(sym_num, sym_tab, "Fopt", "optical pulse frequency");
[sym_num, sym_tab] = add_sym(sym_num, sym_tab, "FdaRate", "optically induced firing rat
[sym_num, sym_tab] = add_sym(sym_num, sym_tab, "FdaRO", "the pulse frequency in the cen
```

In later stages of the modeling, we use the inverse function, FilterFunBS, to return an optical pulse frequency given the dopamine firing frequency it produces. (see ***Functional building blocks for the simulations)***.

The following graph show the function employed to model frequency-following fidelity in the ChR2-expressing dopamine neurons in response to the optical pulse frequency:

```
FdaBend = 20;
FdaRO = 50;
logFopt = 0:0.025:2.4;
Fopt = 10.^logFopt;
FRda = FilterFun(Fopt, FdaBend, FdaRO); % Compute firing rate using the frequency roll-

FF_graphDA = plot_freqFoll(Fopt,FRda,'DA',1,250,1,100);
if show_graphics
    FF_graphDA.Visible = 'on';
end
```

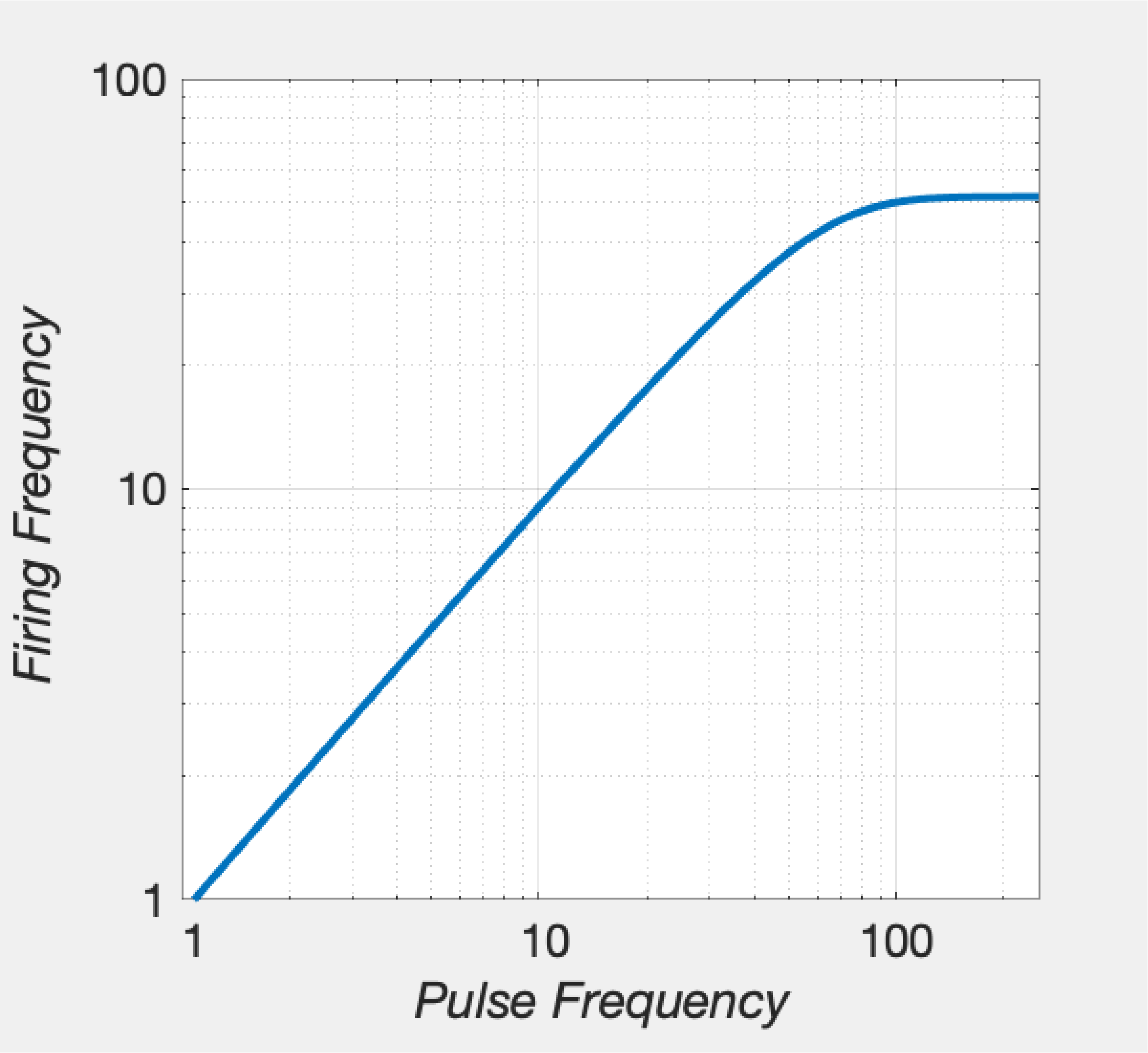


```
[fig_num, fig_tab] = add_fig(fig_num, fig_tab, "FreqFolDA", ...
    "Induced firing frequency in dopamine neurons as a function of pulse frequency");
```

```
This is figure #33: (FreqFolDA)
```

The spike counter

Performance for optical stimulation of midbrain dopamine neurons depends both on the induced firing frequency and the number of dopamine neurons excited by the optical input (Ilango et al., 2014). The simplest assumption consistent with this finding is that the behavioral effects depend on the aggregate rate of induced firing, as is the case of the behavioral effects produced by rewarding electrical stimulation of the medial forebrain bundle. The aggregate rate of firing induced by the optical stimulation in the population of dopamine neurons is the product of the number of optically activated neurons and the induced firing frequency:

$$F_{DA_{agg}} = N_{da} \times F_{DA_{rate}}$$

where

$F_{DA_{agg}}$ = aggregate firing rate of the optically excited dopamine neurons

$F_{DA_{rate}}$ = average firing rate of indindividual dopamine neurons

N_{da} = number of optically excited dopamine neurons

```
[eqn_num, eqn_tab] = ...
    add_eqn(eqn_num, eqn_tab, "AggDAfr", "Aggregate firing rate in the dopamine neurons
```

```
This is equation #30: (AggDAfr)
```

```
[sym_num, sym_tab] = add_sym(sym_num, sym_tab, "FdaAgg", "aggregate firing rate of the
[sym_num, sym_tab] = add_sym(sym_num, sym_tab, "Nda", "number of optically excited dopa

clear a Fopt FRda logFopt;
clear -regexp ^Fda ^FF_graph;
toc
```

```
Elapsed time is 3.287460 seconds.
```

The effect on the reward mountain produced by modulation of dopaminergic neurotransmission

```
tic;
close all;
```

The influence of the induced firing of the dopamine neurons can be modulated by drugs, such as transporter blockers, that alter synaptic transmission. To capture such effects, we add a scalar at the output of the dopamine spike count:

$$D_{drive} = F_{DA_{rate}} \times N_{DA} \times K_{DA}$$

where

D_{drive} = scaled post-synaptic influence of the induced dopamine firing

$F_{DA_{rate}}$ = average firing rate of indindividual dopamine neurons

K_{DA} = constant that scales the postsynaptic impact of the dopamine firing

N_{DA} = number of optically excited dopamine neurons

```
[eqn_num, eqn_tab] = ...
    add_eqn(eqn_num, eqn_tab, "DAdrive", "scaled post-synaptic influence of the induced
```

```
This is equation #31: (DAdrive)
```

```
[sym_num, sym_tab] = add_sym(sym_num, sym_tab, "Kda", "constant that scales the postsyn
```

```
Symbol Kda has already been entered.
```

In the following schema, the aggregate rate of firing is represented by the Π symbol and the dopamine-drive scalar by a triangle:

```
if show_graphics
    show_imported_graphic('counter_opto.png',60,ImpFigDir);
end
```

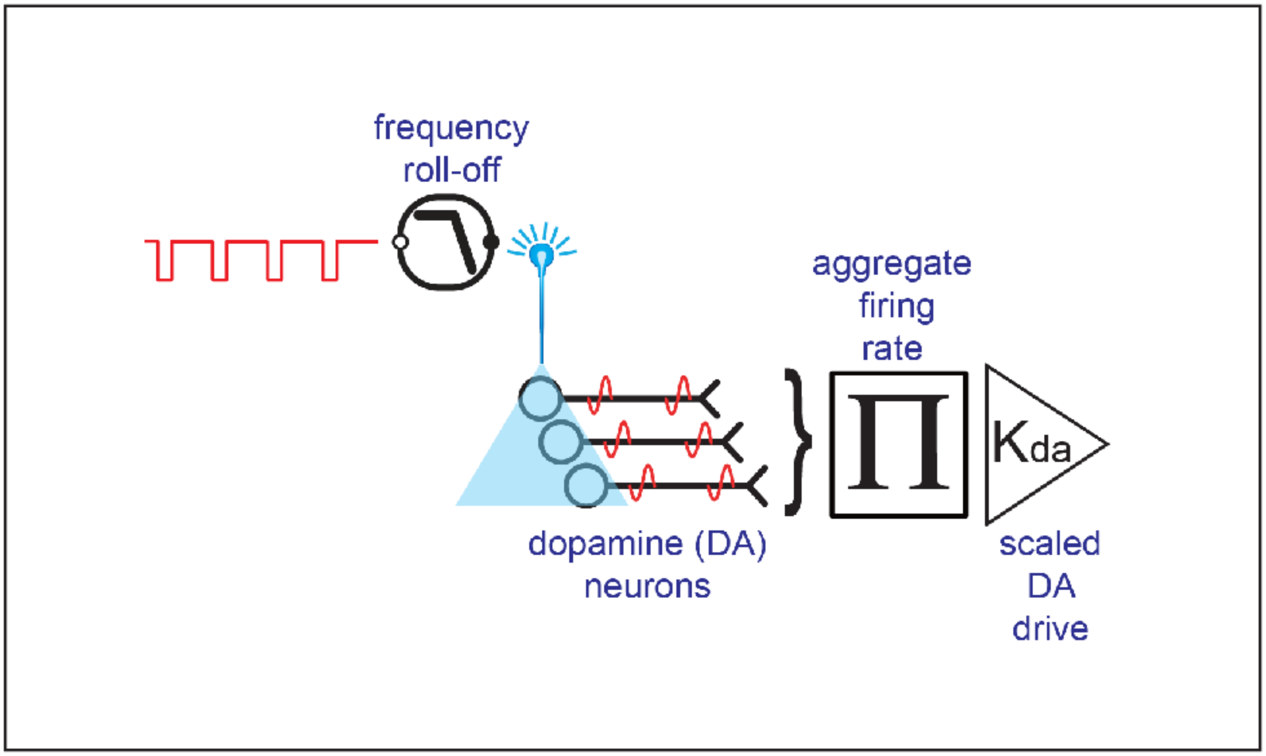


```
[fig_num, fig_tab] = add_fig(fig_num, fig_tab, "DAdrive", ...
    "Influence of optically stimulated dopamine release on downstream targets");
```

```
This is figure #34: (DAdrive)
```

We refer to the scaled output of the activated population of midbrain dopamine neurons as "dopamine drive."

The reward-growth function for oICSS

In six of the seven rats, dopamine-transporter blockade shifted the mountain leftward along the pulse-frequency axis. As we demonstrated above, such shifts require that the function that translates dopamine drive into reward intensity (i.e., the reward-growth function) must have a position parameter that is independent of the parameter that sets the maximum reward intensity attainable. The logistic function described by Gallistel's group in the case of eICSS has this property, and we have therefore added such a function at the output of the counter.

```
if show_graphics
    show_imported_graphic('DA_logisitc_simplified.png',50,ImpFigDir);
end
```

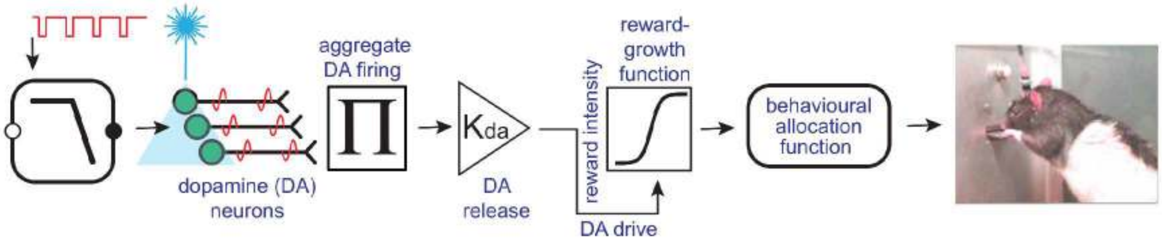


```
[fig_num, fig_tab] = add_fig(fig_num, fig_tab, "oICSS_simplified", ...
    "simplied oICSS model with logistic reward growth");
```

```
This is figure #35: (oICSS_simplified)
```

In the case of eICSS, the form of the two-dimensional reward-growth function employed in the simulations is based on the empirical measurements obtained by Gallistel's group. No such measurements have yet been obtained for oICSS. Future experiments will be required to determine whether the logistic form or another form with independent input- and output-scaling parameters provides the best description of reward growth in the case of oICSS.

In the case of eICSS, the reward-growth function has been generalized to three dimensions {reward intensity, pulse frequency, train duration} on the basis of additional empirical data (Sonnenschein, Conover, & Shizgal, 2003). Both the pulse frequency and train duration have been manipulated in oICSS experiments (Ilango et al., 2014), but not in a way that makes it possible to describe the joint dependence of reward intensity on these two variables. Future experiments will be required to determine this. For present purposes, we will again adopt the function derived from eICSS data for use in simulating oICSS performance.

We define the position parameter of the reward-growth function for oICSS of midbrain dopamine neurons by modifying

```
disp(string({strcat({'Equation '},num2str(eqn_tab.Number(eqn_tab.Name=='fH')))}));
```

```
Equation 17
```

as follows:

$$F_{da_{Agg_{HM}}}(D_{opt}) = \frac{\rho_{da_{Agg}}}{K_{da}} \times \left(1 + \frac{C_{da}}{D_{opt}}\right)$$

where

C_{da} = the train duration at which $F_{da_{Agg_{HM}}}$ is twice the value of $\rho_{da_{Agg}}$

D_{opt} = the train duration

K_{da} = scalar representing the effectiveness of dopaminergic neurotransmission

$\rho_{da_{Agg}}$ = the asymptotic value of $F_{da_{Agg_{HM}}}$ i.e., the value as $D_{opt} \rightarrow \infty$

```
[eqn_num, eqn_tab] = ...
    add_eqn(eqn_num, eqn_tab, "FHda", "strength-duration function for optical trains de
```

```
This is equation #32: (FHda)
```

```
[sym_num, sym_tab] = add_sym(sym_num, sym_tab, "Cda", "chronaxie of the strength-durati
[sym_num, sym_tab] = add_sym(sym_num, sym_tab, "Dopt", "duration of an optical pulse tr
[sym_num, sym_tab] = add_sym(sym_num, sym_tab, "RhoDA", "rheobase of the strength-durat
```

The function `FpulseHMfun`

```
disp(string({strcat({'Function '},num2str(fun_tab.Number(fun_tab.Name=='FpulseHMfun')))
```

```
Function 30
```

`(See` ***Functions composing the reward-mountain model*** `below), accepts an argument list of variable length. If` K_{da} is appended to the end of the argument list, then it will enter into the calculation of $F_{da_{Agg_{HM}}}$ as specified in the above equation. If K_{da} is omitted, it assumes an implicit value of one.

The following code produces estimates of $F_{da_{HM}}$ for two values of K_{da}. The lower value (1) represents the effectiveness of dopaminergic neurotransmission in the vehicle condition, whereas the higher value (2) represents the effectiveness of dopaminergic neurotransmission under the influence of dopamine-transporter blockade.

```
C = 0.473; % median from Sonnenschein et al., 2003
D = 1.0; % train duration for oICSS study
FdaBend = 20;
FdaRO = 50;
Kda = [1,10^.2];
NnDA = 100;
RhoPiDA = 1572; % See the calculation in the Series-circuit section below for D = 1 s
FPmax = 1000;
FdaHMkDA = FpulseHMfun(C, D, FdaBend, FPmax, FdaRO, NnDA, RhoPiDA, Kda)
```

```
FdaHMkDA = 1×2
   27.1046   16.4598
```

```
%FdaHMkDA is a two-element vector
```

Note that although Kda(1) is half the value of Kda(2), FdaHMkDA(2) is less than half the value of FdaHMkDA(1). The reason for this is that the frequency-response function for the dopamine neurons has already started to roll off by FdaHMkDA(1) (some dopamine neurons fail to fire once per pulse at this pulse frequency). This will be reflected in a greater discrepancy between RnormMax(1) and one than between RnormMax(2) and one.

To compute time allocation, we also require the value of the parameter that positions the mountain along the price axis, P_{obj_e}. In the initial simulation, we assume that the value of this parameter is unaffected by the blockade of the dopamine transporter.

```
dotPhiObj = 1;
```

```
Kaa = 1;
Kec = 1;
Krg = 1;
dotRaa = 0.1;
pObj = 1;
PsubBend = 0.5;
PsubMin = 1.82;

FPmax = 1000; % This value is sufficiently above FdaRO to maximize the firing rate
g = 5;
RnormMax = fRbsrNorm(FPmax, FdaBend, FdaHMkDA, FdaRO, g)
```

```
RnormMax = 1×2
    0.9615    0.9967
```

```
% RnormMax is a two-element vector

PobjE = PobjEfun(dotPhiObj, Kaa, Kec, Krg, dotRaa, pObj, PsubBend, PsubMin, RnormMax)
```

```
PobjE = 1×2
    9.6147    9.9670
```

```
% PobjE is a two-element vector
```

We can now estimate time allocation and simulate the reward mountains for the vehicle and drug conditions using the full logistic reward-growth model shown in the figure below:

```
if show_graphics
    show_imported_graphic('oICSS_logistic+3Pe_noMasks_v4.png',25,ImpFigDir);
end
```

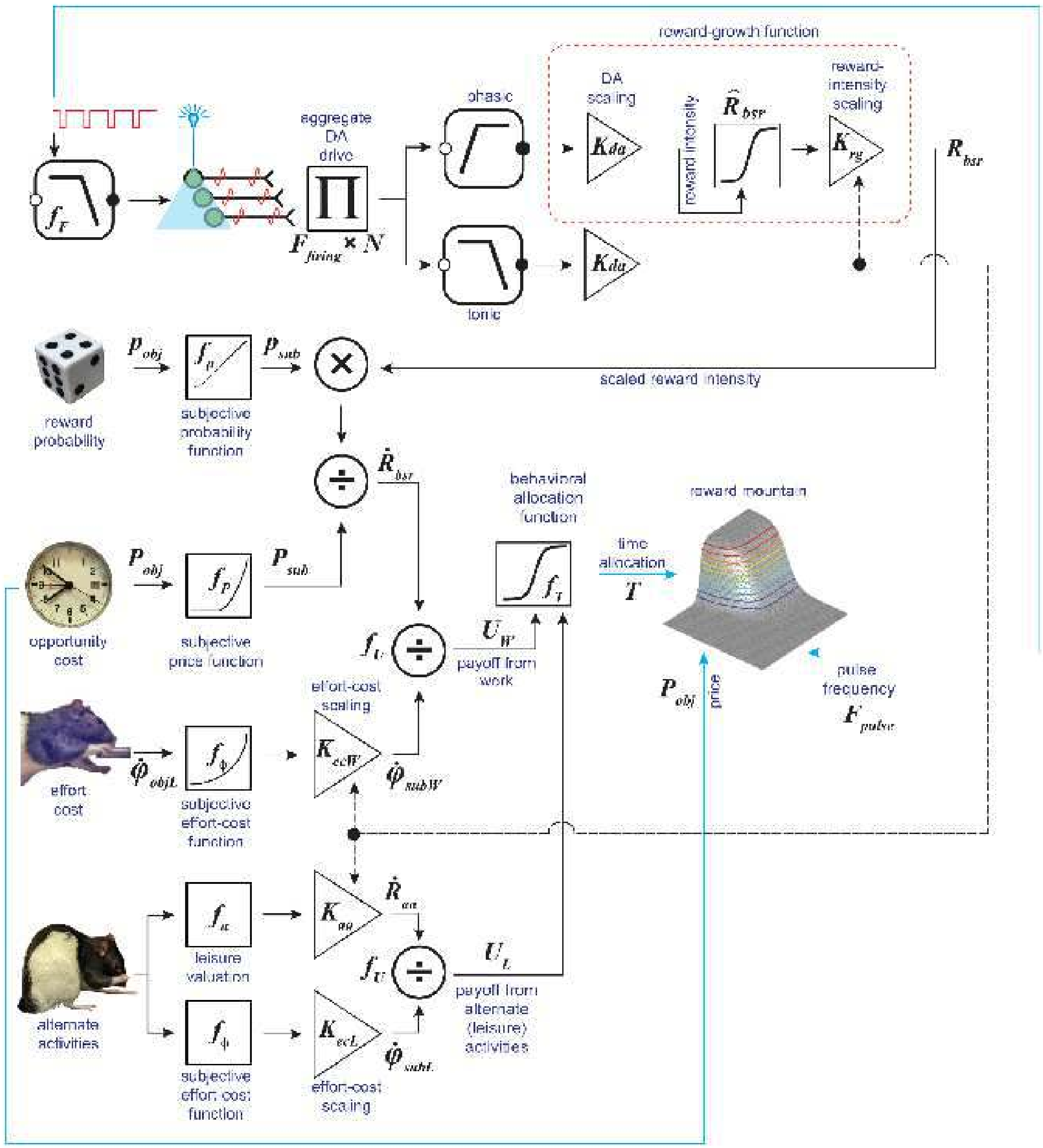


```
[fig_num, fig_tab] = add_fig(fig_num, fig_tab, "oICSS_logistic", ...
    "oICSS model with logistic reward growth");
```

This is figure #36: (oICSS_logistic)

We will now simulate the output of this model, disregarding, for the time being, any drug-induced changes in dopamine tone .

```
a = 3;
Fopt = logspace(0,3,121)'; % column variable
Pobj = logspace(0,3,121); % row variable
Tda1 = TAfun(a, Fopt, FdaBend, FdaHMkDA(1), FdaRO, g, Pobj, PobjE(1), ...
    PsubBend, PsubMin, RnormMax(1));
```

```
Tda2 = TAfun(a, Fopt, FdaBend, FdaHMkDA(2), FdaRO, g, Pobj, PobjE(2), ...
    PsubBend, PsubMin, RnormMax(2));
```

Before plotting the bar graphs, we need to correct the estimates of the location parameters for the differential frequency-following fidelity in the drug and vehicle conditions. The mountain is shifted downwards along the pulse-frequency axis in the drug condition and thus, frequency-following fidelity is better than in the vehicle condition.

As we explain in the main body of the manuscript,

$$F^*_{pulse_{hm}} = F_{firing_{hm}}$$

and

$$P^*_{sub_e} = \frac{P_{sub_e}}{\widehat{R}_{bsr_{max}}}$$

```
FdaHMkDAstar = FilterFun(FdaHMkDA,FdaBend,FdaRO);

PsubE = PsubFun(PobjE,PsubBend,PsubMin);
PsubEstar = PsubE ./ RnormMax;
PobjEstar = PsubBsFun(PsubEstar,PsubBend,PsubMin);

title_str1 = strcat({'Kda = '}, sprintf('%2.2f', Kda(1)));
xmin = 0;
xmax = 2.3;
ymin = 0.3;
ymax = 2.3;
MTNkDA1 = plot_MTN(Fopt, Pobj, Tda1, 'off', 'MTNkDA1', title_str1, ...
    graphs2files, FigDir, xmin, xmax, ymin, ymax);
title_str2 = strcat({'Kda = '}, sprintf('%2.2f', Kda(2)));
MTNkDA2 = plot_MTN(Fopt, Pobj, Tda2, 'off', 'MTNkDA2', title_str2, ...
    graphs2files, FigDir, xmin, xmax, ymin, ymax);
MTNkDA1vskDA2 = dual_subplot(MTNkDA1, MTNkDA2, 'MTNkDA1vskDA2',...
    graphs2files,FigDir);
if show_graphics
    MTNkDA1vskDA2.Visible = 'on';
end
```

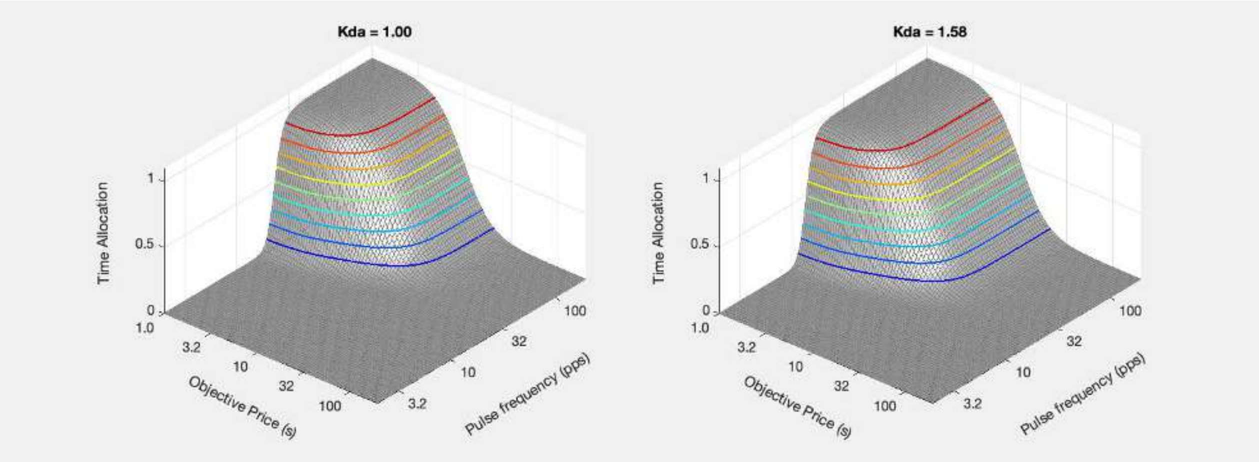


```
[fig_num, fig_tab] = add_fig(fig_num, fig_tab, "kDA1vskDA2_mtns", ...
    "Effect of dopamine-transporter blockade on the reward mountain");
```

```
This is figure #37: (kDA1vskDA2_mtns)
```

```
ContkDA1 = plot_contour(Fopt, Pobj, Tda1, PobjE(1), FdaHMkDA(1), 'off', 'ContkDA1', tit
    strcat({'Kda = '}, num2str(Kda(1))), graphs2files, FigDir,...
    xmin, xmax, ymin, ymax);
ContkDA2 = plot_contour(Fopt, Pobj, Tda2, PobjE(2), FdaHMkDA(2), 'off', 'ContkDA2', tit
    strcat({'Kda = '}, num2str(Kda(2))), graphs2files, FigDir,...
    xmin, xmax, ymin, ymax);
bg_kDA1vskDA2 = plot_bgStar(FdaHMkDA(1), FdaHMkDA(2), FdaHMkDAstar(1), FdaHMkDAstar(2),
    PobjE(1), PobjE(2), PobjEstar(1), PobjEstar(2),...
    'off', 'kDA1vskDA2_bg', graphs2files, FigDir);
bg_root = 'bg_kDA1vskDA2';
quad_kDA1vskDA2 = quad_subplot(ContkDA1, ContkDA2, bg_kDA1vskDA2, 'quad_kDA1vskDA2', bg
    graphs2files, FigDir);
if show_graphics
    quad_kDA1vskDA2.Visible = 'on';
end
```

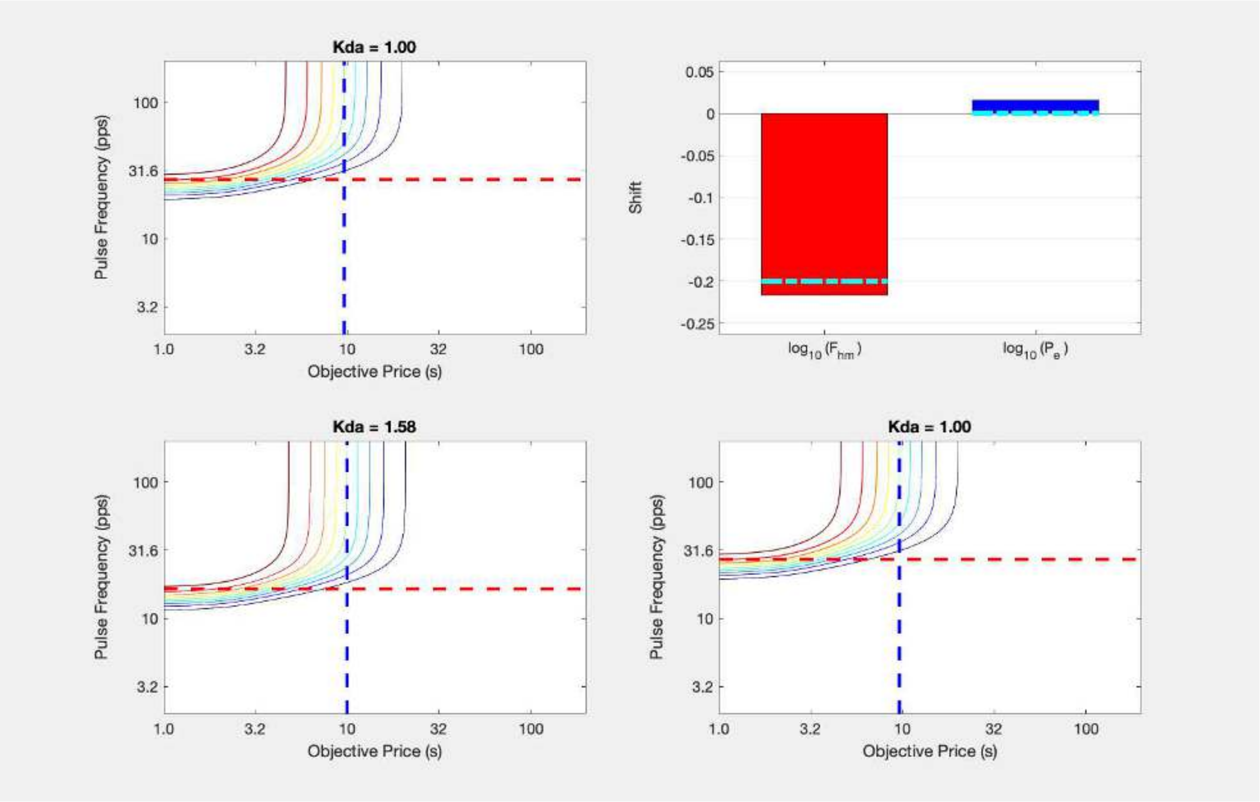


```
[fig_num, fig_tab] = add_fig(fig_num, fig_tab, "kDA1vskDA2_quad", ...
    "Effect of dopamine-transporter blockade on the reward mountain");
```

```
This is figure #38: (kDA1vskDA2_quad)
```

In the above simulation, the drug-induced boost in the effectiveness of dopaminergic neurotransmission has rescaled the input to the reward-growth function. As a result, the mountain shifts leftwards along the pulse-frequency axis. This captures one of the observed effects of GBR-12909.

The small shift along the price axis is due to the fact that $R_{norm_{Max}}$ is closer to one in the drug condition than in the vehicle condition. This is due to the fact that F_{hm} is lower in the drug condition and thus further from the pulse frequency at which frequency following begins to roll off. The corrected value of $\log_{10}(F_{hm})$ is designated by the dot-dash cyan line on the red bar.

The difference in frequency-following fidelity in the drug and vehicle conditions also produces a small shift in $\log_{10}(P_e)$. This shift is removed by the correction (dot-dash cyan line on the blue bar).

The empricially observed rightward shifts along the pulse axis survive the correction for differential frequency-following fidelity. To capture these, a scalar increase must be produced at or beyond the output of the reward-growth function. In the example shown below, we do this by having the drug-induced increase in dopamine tone reduce the subjective effort cost, as proposed by Salomone and colleagues.

```
clearvars('-except',keepVars{:})
keepVars = who; % Restore cell array containing names of variables to be retained
toc
```

```
Elapsed time is 10.708417 seconds.
```

Dopaminergic modulation of subjective effort cost

```
tic;
close all;
```

In

```
disp(string({strcat({'Figure '},num2str(fig_tab.Number(fig_tab.Name=='oICSS_logistic'))
```

```
Figure 36
```

, blockade of the dopamine transporter changes both phasic and tonic dopamine signaling. Given that the lack of correlation between the magnitudes of the resulting shifts along the pulse-frequency and price axes, separate scalars are included for phasic and tonic signaling. The schema indicates three different ways in which increases in tonic signaling could shift the mountain along the price axis, as designated by the black dashed line. The one simulated here is a reduction in the rate of subjective exertion entailed in holding down the lever.

```
% Set Fhm to the values obtained from ratBechr29 in the vehicle and drug conditions
Kda = [1,1.4458]; % Kda value for Bechr29
FdaHMkDA = [31.34679397, 20.77560124]; % values for Bechr29
FdaHMkDAstar = [26.29253041, 18.18388501]; % values for Bechr29

PsubBend = 0.5;
PsubMin = 1.8197;

FdaBend = 20;
FdaRO = 50;
FPmax = 200; % This is the value that was employed in the correction of the location pa
g = 3.160774009; % value for Bechr29; value common for Veh and Drg conditions
RnormMax = [0.893580092, 0.964209143]; % means of resampled values for Bechr29

% PobjE = PobjEfun(dotPhiObj, Kaa, Kec, Krg, dotRaa, pObj, PsubBend, PsubMin, RnormMax,
PobjE = [6.295449067, 10.04096421]; % values for Bechr29
PobjEstar = [7.375024269, 10.47998071]; % values for Bechr29

% We can't simply perform the corrections of the location parameters on the means.
% The original corrections were performed on the 250 resampled log values.
% The statistics are based on these vectors.
% The log of the mean is not equal to the mean of the logs.
% Similarly, we cannot simply use FilterFun to correct the mean Fhm values and use thes
% logFfiringHM (i.e., logFhmStar) and logPobjEstar were estimated from each of the 250
% logFpulseHM and logPobjE. We encounter the same issue.
% The values used above are based on the 250 resampled log values, which were loaded di
% saved workspace that was used in the fits.
```

```
a = 3;
Fopt = logspace(0,3,121)'; % column variable
Pobj = logspace(0,3,121); % row variable
```

```
Tda1 = TAfun(a, Fopt, FdaBend, FdaHMkDA(1), FdaRO, g, Pobj, PobjE(1), ...
    PsubBend, PsubMin, RnormMax(1));
Tda2 = TAfun(a, Fopt, FdaBend, FdaHMkDA(2), FdaRO, g, Pobj, PobjE(2), ...
    PsubBend, PsubMin, RnormMax(2));

xmin = 0;
xmax = 2.3;
ymin = 0.3;
ymax = 2.3;
title_str1 = strcat({'Kda = '}, sprintf('%2.2f', Kda(1)));
MTNkDAeffMod1 = plot_MTN(Fopt, Pobj, Tda1, 'off', 'MTNoICSSkDAeffMod1', title_str1, ...
    graphs2files, FigDir, xmin, xmax, ymin, ymax);
title_str2 = strcat({'Kda = '}, sprintf('%2.2f', Kda(2)));
MTNkDAeffMod2 = plot_MTN(Fopt, Pobj, Tda2, 'off', 'MTNoICSSkDAeffMod2', title_str2, ...
    graphs2files, FigDir, xmin, xmax, ymin, ymax);
MTNoICSSkDA1vskDA2 = dual_subplot(MTNkDAeffMod1, MTNkDAeffMod2, 'MTNoICSSkDA1vskDA2',...
    graphs2files,FigDir);
if show_graphics
    MTNoICSSkDA1vskDA2.Visible = 'on';
end
```

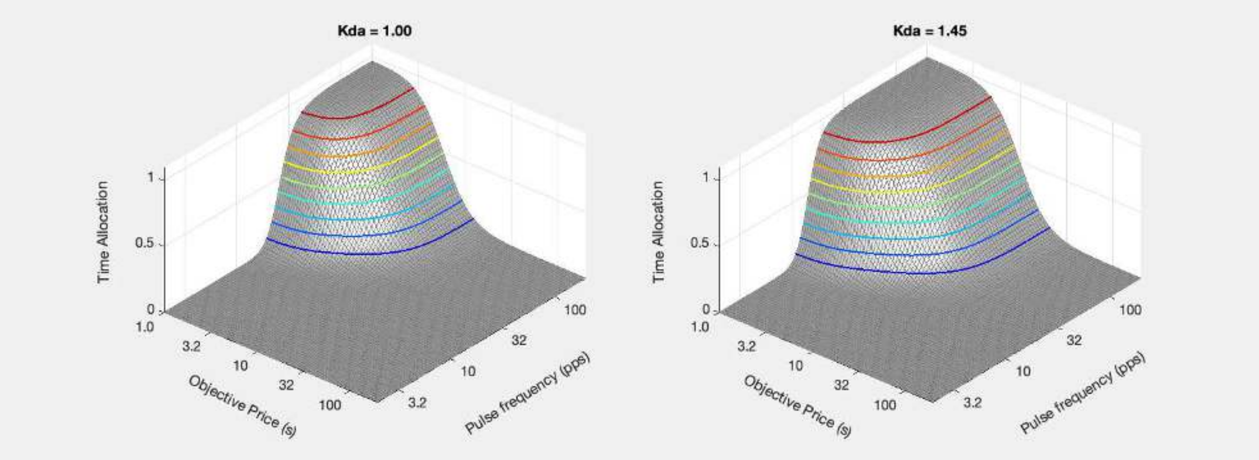


```
[fig_num, fig_tab] = add_fig(fig_num, fig_tab, "kDA1vskDAeffMod2_mtns", ...
    "Effect of dopamine-transporter blockade on the reward mountain");
```

This is figure #39: (kDA1vskDAeffMod2_mtns)

```
ContkDAeffMod1 = plot_contour(Fopt, Pobj, Tda1, PobjE(1), FdaHMkDA(1), 'off', 'ContkDAe
    strcat({'Kda = '}, num2str(Kda(1))), graphs2files, FigDir,...
    xmin, xmax, ymin, ymax);
ContkDAeffMod2 = plot_contour(Fopt, Pobj, Tda2, PobjE(2), FdaHMkDA(2), 'off', 'ContkDAe
    strcat({'Kda = '}, num2str(Kda(2))), graphs2files, FigDir,...
    xmin, xmax, ymin, ymax);
ymin = -0.3581; % from the bargraph for Bechr29
ymax = 0.3652; % from the bargraph for Bechr29
bg_kDA1vskDA2effMod = plot_bgStar(FdaHMkDA(1), FdaHMkDA(2), FdaHMkDAstar(1), FdaHMkDAst
    PobjE(1), PobjE(2), PobjEstar(1), PobjEstar(2),...
    'off', 'kDA1vskDA2_bg', graphs2files, FigDir,...
        ymin, ymax);
bg_root = 'bg_kDA1vskDAeffMod2';
quad_kDA1vskDA2effMod = quad_subplot(ContkDAeffMod1, ContkDAeffMod2, bg_kDA1vskDA2effMo
```

```
    'quad_kDA1vskDAeffMod2', bg_root, graphs2files, FigDir);
if show_graphics
    quad_kDA1vskDA2effMod.Visible = 'on';
end
```

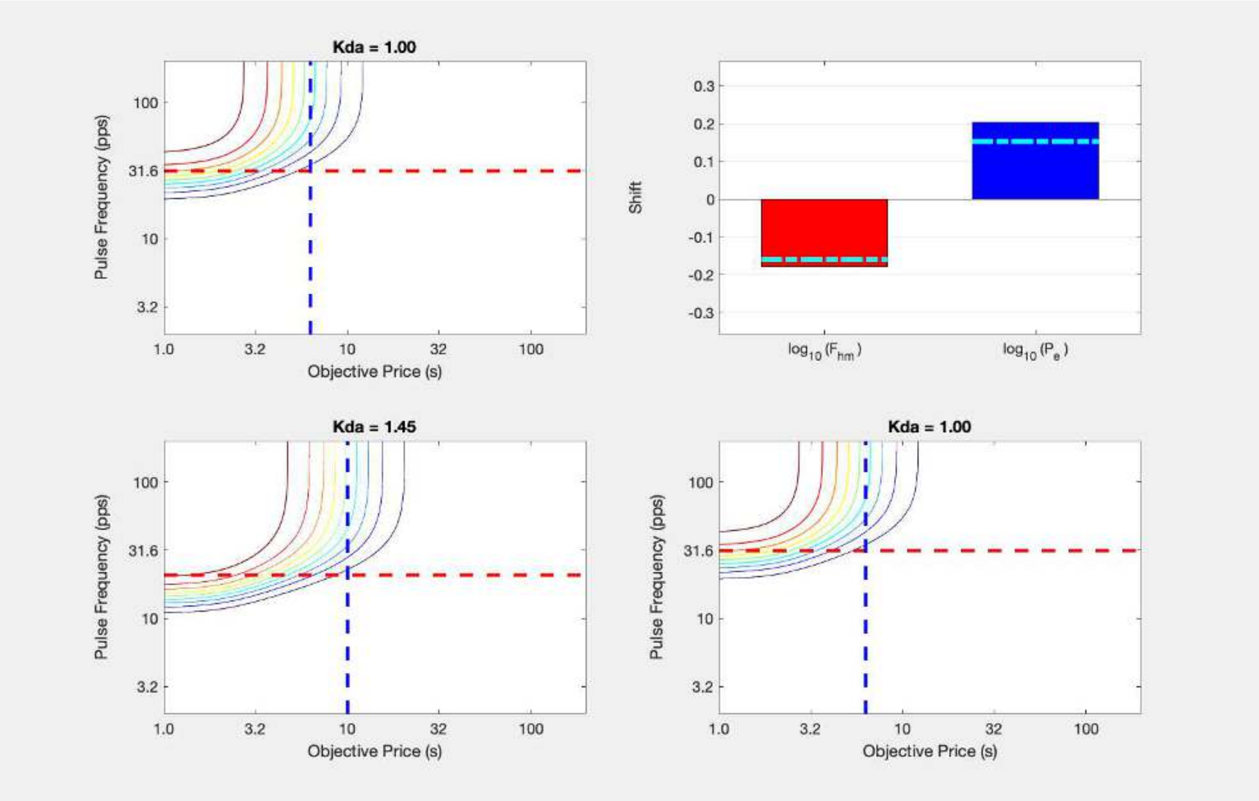


```
[fig_num, fig_tab] = add_fig(fig_num, fig_tab, "kDA1vskDA2effMod_quad", ...
    "Effect of dopamine-transporter blockade on the reward mountain");
```

```
This is figure #40: (kDA1vskDA2effMod_quad)
```

The simulated results are now in qualitative accord with the empirical findings: the mountain shifts leftwards along the pulse-frequency axis and rightwards along the price axis in response to blockade of the dopamine transporter.

Here are the shifts observed in the mountains obtained from rat BeChR29 and the shifts simulated above using the K_{da} and $P^*_{obj_e}$ values derived from that rat's data:

```
BeChR29_bg = make_fig_from_png(fullfile(ImpFigDir,'BeChR29_bg.png'), 30);
BeChR29bgVSsimBG = dual_subplot(BeChR29_bg, bg_kDA1vskDA2effMod, 'BeChR29bgVSsimBG',...
    graphs2files,FigDir);
% rescale the right panel to 60% width, 80% height & center in right panel
BeChR29bgVSsimBGrs = adjust_right_panel(BeChR29bgVSsimBG, 0.6, 0.825);
if show_graphics
    BeChR29bgVSsimBGrs.Visible = 'on';
end
```

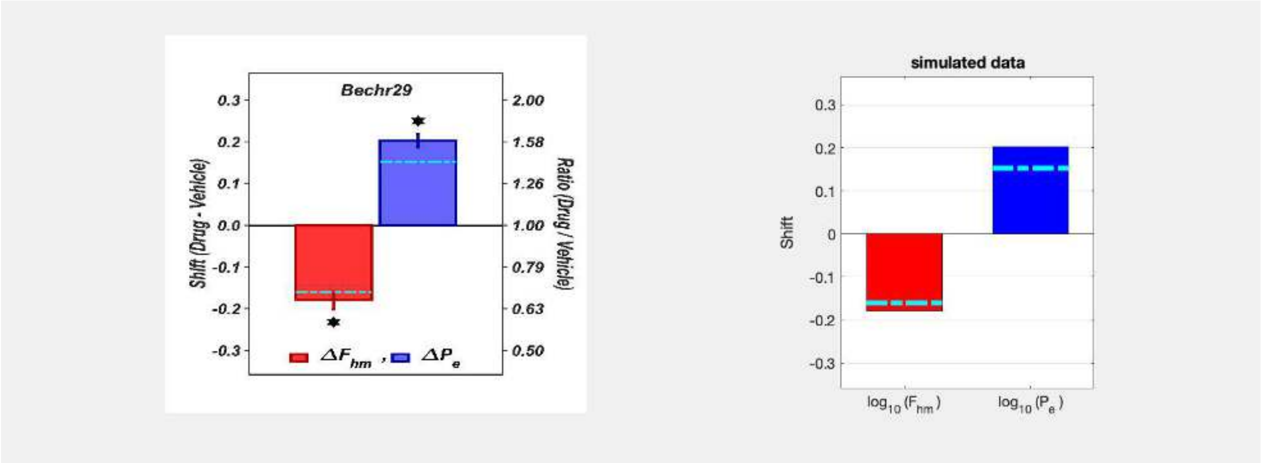


```
[fig_num, fig_tab] = add_fig(fig_num, fig_tab, "BeChR29bgVSsimBGrs", ...
    "Simulated and observed effects of dopamine-transporter blockade on the reward mour
```

```
This is figure #41: (BeChR29bgVSsimBGrs)
```

The correspondence is forced by inputting values of $F^*_{pulse_{hm}}$, $P^*_{obj_e}$, and g obtained from the fit of the reward-mountain model to the empirical data. All that this comparison does is to verify the functions for computing time allocation and graphing the results.

Full simulation would yield the same results if we adjusted $\dot{\phi}_{obj}, K_{aa}, K_{ec}, K_{rg}$, and $\dot{R}_{aa}$ so as to generate the observed values.

```
clearvars('-except',keepVars{:})
keepVars = who; % Restore cell array containing names of variables to be retained
toc
```

```
Elapsed time is 11.140447 seconds.
```

The series-circuit model of eICSS and oICSS

```
tic;
close all;
```

In the previous section, we demonstrate how the effects of dopamine-transporter blockade on oICSS can be explained within the context of the mountain model by a combination of changes in phasic and tonic dopamine signaling. In this section, we pose an additional challenge: can the explanation proposed for the oICSS data also account for the known effects of dopamine-transporter blockade on eICSS? Below, we show that the proposed explanation fails to meet this challenge, we discuss the implications for the "series-circuit" model of eICSS, and we develop a new model that can account for the effects of dopamine-transporter blockade on both oICSS and eICSS.

The series-circuit model treats oICSS and eICSS as behavioral manifestations of the effects produced by injecting signals at two different neural stages of the same pathway. On this view, eICSS arises from electrically induced activation of highly excitable, non-dopaminergic neurons that project directly or indirectly to midbrain dopamine neurons. In other words, the directly activated neurons that give rise to eICSS are in series with the

dopamine neurons that render the electrical stimulation rewarding. The midbrain dopamine neurons are excited trans-synaptically in the case of eICSS and directly in the case of oICSS. The consequences of their activation are the same: the subject seeks to re-initiate the stimulation and will pay large effort and opportunity costs when the strength of the stimulation suffices to produce a large increment in the aggregate firing rate of the dopamine neurons.

Here, we show only the portion of the model that generates the reward-intensity signal.

```
if show_graphics
    show_imported_graphic('series-circuit_DA_double_logisitc_RG_v2.png',15,ImpFigDir);
end
```

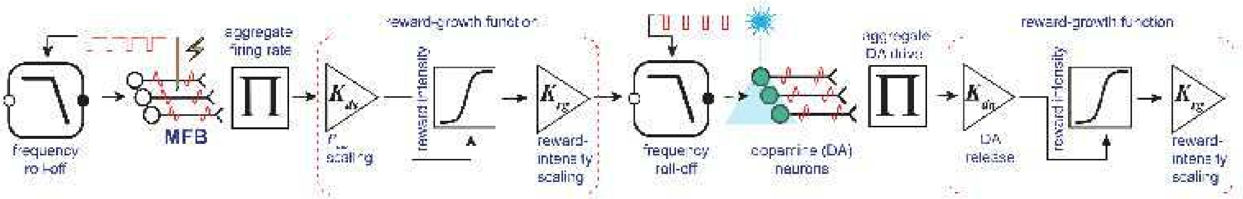


```
[fig_num, fig_tab] = add_fig(fig_num, fig_tab, "SeriesCircuit_eICSS_oICSS", ...
    "Series-circuit model of eICSS and oICSS");
```

```
This is figure #42: (SeriesCircuit_eICSS_oICSS)
```

As the figure above shows, two different reward-growth functions are required, one upstream of the dopamine neurons and another downstream. The upstream reward-growth function is necessary in order to account for the data summarized in

```
disp(string({strcat({'Figure '},num2str(fig_tab.Number(fig_tab.Name=='PimGBRshifts')))}
```

```
Figure 28
```

That figure shows that modulation of dopaminergic neurotransmission alters eICSS performance by shifting the reward mountain along the price axis and not along the pulse-frequency axis. This implies that the drug-induced change in dopamine signaling acts at or beyond the output of the reward-growth function for eICSS. For this to occur in the series-circuit model, the reward-growth function for eICSS must lie upstream (to the left) of the dopamine neurons.

A second reward-growth function must lie downstream (to the right) of the dopamine neurons. This is required because GBR-12909 shifted the reward mountain for oICSS along the pulse-frequency axis, as shown in

```
disp(string({strcat({'Figures '},num2str(fig_tab.Number(fig_tab.Name=='BeChR29vehdrgMtr
```

```
Figures 30
```

and

```
disp(string({strcat({''},num2str(fig_tab.Number(fig_tab.Name=='GBRshiftSummary')))}));
```

```
32
```

We explain above (***The significance of orthogonal shifts***) that the mountain will move along the pulse-frequency axis only if the input to the reward-growth function has been rescaled. Such rescaling occurs when

the magnitude of dopamine transients is boosted by GBR-12909 and the reward-growth function for oICSS is positioned downstream of the dopamine neurons.

<u>Series-circuit model: changes in the reward mountain for eICSS in response to dopamine-transporter blockade</u>

To generate a reward mountain for eICSS from the series-circuit model, we proceed in several stages. As was done above (**The reward-growth function for oICSS**), the optical pulse frequency required to produce a half-maximal reward intensity is calculated directly from

```
disp(string({strcat({'Equation '},num2str(eqn_tab.Number(eqn_tab.Name=='fH')))}));
```

```
Equation 17
```

We next need to compute the electrical pulse frequency (applied to a medial forebrain bundle electrode) that produces excitation in the dopamine neurons equivalent to that produced by a given train of optical pulses delivered to the midbrain dopamine neurons. According the series-circuit model, all inputs that produce the same peak output from the dopamine neurons will produce the same rewarding effect. This will be true regardless of whether the dopamine neurons are excited directly by optical activation or indirectly by trans-synaptic input from medial forebrain bundle neurons activated by electrical stimulation. According to the model, an observer placed downstream from the dopamine neurons and supplied only with information about the aggregate peak output of these neurons cannot know whether optical or electrical stimulation was responsible for a given phasic increase in dopamine release. It is the peak magnitude of this phasic increase in aggregate firing that determines the intensity of the rewarding effect.

(See "**The spike counter"** above.)

To obtain the electrical pulse frequency required to produce a reward of half-maximal intensity, we need to back-solve the equations describing the stages of the model that intervene between the electrode and the dopamine neurons. The back-solutions return the electrical pulse frequency that delivers an input to dopamine neurons equivalent to the optical pulse frequency that produces a half-maximal reward intensity.

The reward-growth functions (S-shaped curves in rectangular boxes) in the flow diagrams are normalized: Their output varies from zero to one and is then scaled by the variable in the triangle to their right. In the case of the upstream reward-growth function in

```
disp(string({strcat({'Figure '},num2str(fig_tab.Number(fig_tab.Name=='SeriesCircuit_eIC
```

```
Figure 42
```

(the reward-growth function to the left of the dopamine neurons), that scaling variable is $K_{rg_{eq}}$. The value of this variable determines the maximum input that the electrode can deliver to the dopamine neurons, scaled in terms of the equivalent optical pulse frequency. In the initial simulation below, $K_{rg_{eq}}$ is set to 63 pulses per second, and given the parameters of the frequency-following function of the dopamine neurons $\{F_{da_{RO}}, F_{da_{Bend}}\}$, this will drive the dopamine neurons to a near-maximal level (~43 spikes s^{-1}). We demonstrate below that the qualitative effect of dopamine transporter blockade is little affected by the value of $K_{rg_{eq}}$.

The function that computes the required electrical pulse frequency in the series-circuit model, *FscHMbs*, comprises three back-solutions. (See ***Functions composing the reward-mountain model*** below.) First, we invert the scaling of the output of the upstream reward-growth function: we divide the optical pulse frequency required to produce a reward of half-maximal intensity by $K_{rg_{eq}}$. This gives us the output of the upstream normalized reward-growth function (a value between zero and one), the fraction of $K_{rg_{eq}}$ required to produce a half-maximal reward intensity at the output of the dopamine neurons (the FdaHMkDA value). We then backsolve the upstream reward-growth function (by means of the `LogistNormBsFun` function) to obtain the average firing rate of the medial forebrain bundle neurons required to produce the reward intensity in question, given the position parameter of the logistic reward-growth function and the value of its exponent. The position parameter of the logistic is calculated using

```
disp(string({strcat({'Equation '},num2str(eqn_tab.Number(eqn_tab.Name=='fH')))}));
```

```
Equation 17
```

(the *FFmfbHM* function). Finally, we use the *FilterFunBS* function to obtain the electrical pulse frequency that produces this average firing rate.

The input to *FscHMbs* is the optical pulse frequency required to produce a reward of half-maximal intensity ($F_{da_{hm_{Kda}}}$) by direct activation of the dopamine neurons, and the output is the equivalent electrical pulse frequency ($F_{sc_{HM}}$).

```
[fun_num, fun_tab] = add_fun(fun_num, fun_tab, "FscHMbs", ...
    "FhmDA, FmfbBend, FhmMFB, FmfbRO, gMFB, KrgEq",...
    "Back solution of the full reward-growth function for the series-circuit (sc) model
```

```
This is function #39: (FscHMbs)
```

```
[sym_num, sym_tab] = add_sym(sym_num, sym_tab, "FscHM", "position parameter of RG funct
```

We now compute the value of the *FscHMbs* function for the vehicle and drug conditions.

```
%% Calculate FscHM for the vehicle and drug conditions
% Calculate the position parameter of the upstream reward-growth function
C = 0.473; % median from Sonnenschein et al., 2003
D = 0.5; % typical train duration
FdaBend = 20;
logFmfbBend = 1.3222; % from Solomon et al., 2015
FmfbBend = 10^logFmfbBend
```

```
FmfbBend = 20.9991
```

```
FdaRO = 50;
gMFB = 1.58; % reduce the value due to the embedding within the DA RG function
logFmfbRO = 2.5587; % from Solomon et al., 2015
FmfbRO = 10^logFmfbRO
```

```
FmfbRO = 361.9929
```

```
NnMFB = 126;
```

```
RhoPiMFB = 5000; % RhoPi/N is within ~20% of 50, which is roughly consistent with Sonne

FPmax = 1000;
FmfbHM = FpulseHMfun(C, D, FmfbBend, FPmax, FmfbRO, NnMFB, RhoPiMFB)
```

```
FmfbHM = 77.2222
```

In the vehicle condition of the current experiment, the median $F_{pulse_{hm}}$ value for oICSS was 27.1. The train duration was 1 s, whereas it was 0.5 s in the corresponding eICSS study. The form and parameters of the temporal-integration function for oICSS of midbrain dopamine neurons are unknown. Faut de mieux, we will use the functional form and parameters obtained from eICSS to compute the Fhm value for a 0.5 s train that corresponds to the value obtained in the current oICSS study for 1 s trains.

In the series-circuit model, the output of the MFB neurons must pass through the midbrain dopamine neurons. If so, the temporal-integration characteristics obtained in eICSS experiments reflect those of both the directly stimulated and dopamine stages of the circuit. According to this model, integration in the dopamine stage cannot be faster than estimated in the eICSS study.

It follows from

```
disp(string({strcat({'Equation '},num2str(eqn_tab.Number(eqn_tab.Name=='fH')))}));
```

```
Equation 17
```

that the Fhm value for a 0.5 s train that corresponds to an Fhm value for a 1 s train is given by

$$F_{firing_{hm_{D_2}}} = F_{firing_{hm_{D_1}}} \times \frac{1 + \frac{C}{D_2}}{1 + \frac{C}{D_1}}$$

```
[eqn_num, eqn_tab] = add_eqn(eqn_num, eqn_tab, ...
    "FfiringD1D2", "calculates equivalently effective pulse frequencies at two differen
```

```
This is equation #33: (FfiringD1D2)
```

Given the frequency-following function, the median $F_{pulse_{hm}}$ value for the vehicle condition (27.1) corresponds to a firing frequency of 23.1.

```
FpulseHMmedVeh = 27.094;
FfiringHMmedVeh = FilterFun(FpulseHMmedVeh, FdaBend, FdaRO)
```

```
FfiringHMmedVeh = 23.1475
```

Given these values

```
C = 0.473;
D1 = 1;
D2 = 0.5;
FfiringHmD1 = FfiringHMmedVeh
```

```
FfiringHmD1 = 23.1475
```

```
FfiringHmD2 = FfiringHmD1 .* (1+(C./D2)) ./ (1+(C./D1))
```

```
FfiringHmD2 = 30.5805
```

```
FPmax = 1000;
FpulseHmD2 = FilterFunBS(FdaBend, FdaRO, FfiringHmD2,FPmax)
```

```
FpulseHmD2 = 37.6179
```

The MFB will have to deliver excitation equivalent to $F_{pulse_{hmD_2}}$ to deliver a half-maximal reward intensity from the dopamine neurons at a train duration of 0.5 s. We will determine the values of $K_{rg_{eq}}$ and $\rho_{\Pi_{da}}$ so as to obtain the above $F_{firing_{hmD_2}}$ value in the vehicle condition.

We first set the number of activated dopamine neurons arbitrarily to 100.

```
NnDA = 100;
```

Then, we use

```
disp(string({strcat({'Equation '},num2str(eqn_tab.Number(eqn_tab.Name=='FfiringD1D2')))
```

```
Equation 33
```

to find the required value of $\rho_{\Pi_{da}}$, keeping in mind that $\rho_{\Pi_{da}}$ is the aggregate firing rate required to produce a half-maximal reward intensity with a train of infinite duration. Setting D1 to infinity makes the denominator of

```
disp(string({strcat({'Equation '},num2str(eqn_tab.Number(eqn_tab.Name=='FfiringD1D2')))
```

```
Equation 33
```

equal to one, so the equation is simplified to:

```

RhoPiDA = NnDA * (FfiringHmD2/(1+(C/D2)))
```

```
RhoPiDA = 1.5715e+03
```

We are now positioned to compute the MFB pulse frequency that will drive the dopamine neurons to produce a reward of half-maximal intensity.

```
Kda = [1,10^0.15]; % Effect of dopamine-transporter blockade on the frequency of firin
% to produce a reward of half-maximal intensity. Median Kda in current oICSS study: 10
FPmax = 1000; % This pulse frequency well above FdaRO and can thus serve to estimate th
FdaHMkDA = FpulseHMfun(C, D2, FdaBend, FPmax, FdaRO, NnDA, RhoPiDA, Kda) % result is a
```

```
FdaHMkDA = 1×2
   37.6179   25.1418
```

Now, we work backwards through the MFB input to find the electrical pulse frequency that will drive the dopamine neurons to produce a reward of half-maximal intensity.

```
KrgEq = 63;
```

```
FFmax = FilterFun(FPmax, FmfbBend, FmfbRO);
if FFmax > LogistNormBsFun(gMFB,FmfbHM,max(FdaHMkDA)/KrgEq)
    for j = 1:length(FdaHMkDA)
        FscHM(j) = FscHMbs(FdaHMkDA(j), FmfbBend, FmfbHM, FmfbRO, FPmax, gMFB, KrgEq) %
    end
else
    display("FscHM could not be calculated because KrgEq is too low.");
    display("Set KrgEq such that FFmax > FilterFun(FPmax, Fbend, Fro).");
    return
end
```

```
FscHM = 99.0574
FscHM = 1×2
   99.0574   59.5983
```

```
% Calculate PobjE for the vehicle and drug conditions
dotPhiObj = 1;
Kaa = 1;
Kec = 1;
KeffMod = Kda; % We tie the subjective effort cost to the effect of the drug
Kda
```

```
Kda = 1×2
    1.0000    1.4125
```

```
% Median shift in Pe in the current oICSS study: 10^0.1525
Krg = 1;
dotRaa = 0.1;
pObj = 1;
PsubBend = 0.5;
PsubMin = 1.82;

Felec = logspace(0,3,121)'; % column variable
```

To solve the time-allocation equation for the series-circuit model, we must first compute the drive on the dopamine neurons that is produced by each value of the electrical pulse frequency (F_{elec}). We express this drive in terms of the optical pulse frequency that produces equivalent firing in the dopamine neurons, $F_{opt_{equiv}}$.

The *FoptEquivFun* function first translates the electrical pulse frequency into the induced rate of firing in the medial forebrain bundle neurons, then translates this firing rate into a normalized reward intensity, and then converts the normalized reward intensity into the equivalent optical pulse frequency. Once this has been done, time allocation can be computed using the same equation and parameter values that were used to generate the reward mountain for optical stimulation. The *FoptEquivFun* function is the inverse of the *FscHMbs* function. (See ***Functions composing the reward-mountain model*** below.)

```
[fun_num, fun_tab] = add_fun(fun_num, fun_tab, "FoptEquivFun", ...
    "Felec, FmfbBend, FmfbHM, FmfbRO, gMFB, KrgE",...
    "Function to compute the optical pulse frequency that produces the same DA output a
```

```
This is function #40: (FoptEquivFun)
```

```
[sym_num, sym_tab] = add_sym(sym_num, sym_tab, "FoptEquiv", "optical pulse frequency th

FoptEquiv = FoptEquivFun(Felec, FmfbBend, FmfbHM, FmfbRO, gMFB, KrgEq);
```

```
FPmaxEq = max(FoptEquiv) % This is the highest equivalent optical pulse frequency that
```

```
FPmaxEq = 57.9538
```

```
gDA = 5; % Use a value consistent with the oICSS study; median in vehicle condition was
RnormMax = fRbsrNorm(FPmaxEq, FdaBend, FdaHMkDA, FdaRO, gDA)
```

```
RnormMax = 1×2
    0.6146    0.9228
```

```
% RnormMax is a 2-element vector
PobjE = PobjEfun(dotPhiObj, Kaa, Kec, Krg, dotRaa, pObj, PsubBend, PsubMin, RnormMax, K
```

```
PobjE = 1×2
    6.1461   13.0354
```

```
% PobjE is a two-element vector

Pobj = logspace(0,3,121); % row variable

% Compute time allocation for the vehicle and drug conditions
a = 2; % median in vehicle condition was 1.84
Tsc1 = TAfun(a, FoptEquiv, FdaBend, FdaHMkDA(1), FdaRO, gDA, Pobj, ...
    PobjE(1), PsubBend, PsubMin, RnormMax(1));
Tsc2 = TAfun(a, FoptEquiv, FdaBend, FdaHMkDA(2), FdaRO, gDA, Pobj, ...
    PobjE(2), PsubBend, PsubMin, RnormMax(2));

title_str1 = strcat({'Kda = '}, sprintf('%2.2f', Kda(1)));
MTNkDA1sc = plot_MTN(Felec, Pobj, Tsc1, 'off', 'MTNkDA1sc', title_str1, ...
    graphs2files, FigDir);
title_str2 = strcat({'Kda = '}, sprintf('%2.2f', Kda(2)));
MTNkDA2sc = plot_MTN(Felec, Pobj, Tsc2, 'off', 'MTNkDA2sc', title_str2, ...
    graphs2files, FigDir);
MTNkDA1vskDA2sc = dual_subplot(MTNkDA1sc, MTNkDA2sc, 'MTNkDA1vskDA2sc',...
    graphs2files,FigDir);
if show_graphics
    MTNkDA1vskDA2sc.Visible = 'on';
end
```

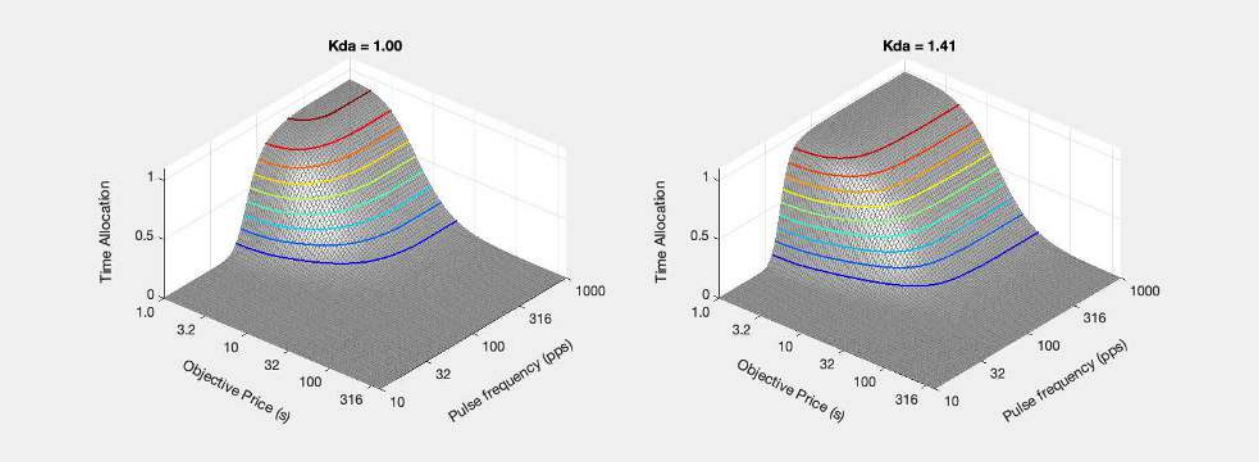


```
[fig_num, fig_tab] = add_fig(fig_num, fig_tab, "kDA1vskDA2_sc_mtns", ...
```

```
    "Effect of dopamine-transporter blockade on the reward mountain");
```

This is figure #43: (kDA1vskDA2_sc_mtns)

```
FscHMstar = FilterFun(FdaHMkDA,FdaBend,FdaRO);
% Computed on the basis of frequency following in the dopamine neurons

PsubE = PsubFun(PobjE,PsubBend,PsubMin);
PsubEstar = PsubE ./ RnormMax;
PobjEstar = PsubBsFun(PsubEstar,PsubBend,PsubMin);
```

```
ContkDA1sc = plot_contour(Felec, Pobj, Tsc1, PobjE(1), FscHM(1), 'off', 'ContkDA1sc', t
    strcat({'Kda = '}, num2str(Kda(1))), graphs2files, FigDir);
ContkDA2sc = plot_contour(Felec, Pobj, Tsc2, PobjE(2), FscHM(2), 'off', 'ContkDA2sc', t
    strcat({'Kda = '}, num2str(Kda(2))), graphs2files, FigDir);
bg_kDA1vskDA2sc = plot_bgStar(FscHM(1), FscHM(2), FscHMstar(1), FscHMstar(2),...
    PobjE(1), PobjE(2), PobjEstar(1), PobjEstar(2),...
    'off', 'kDA1vskDA2sc_bg', graphs2files, FigDir);
bg_root = 'bg_kDA1vskDA2sc';
quad_kDA1vskDA2sc = quad_subplot(ContkDA1sc, ContkDA2sc, bg_kDA1vskDA2sc, 'quad_kDA1vsk
    graphs2files, FigDir);
if show_graphics
    quad_kDA1vskDA2sc.Visible = 'on';
end
```

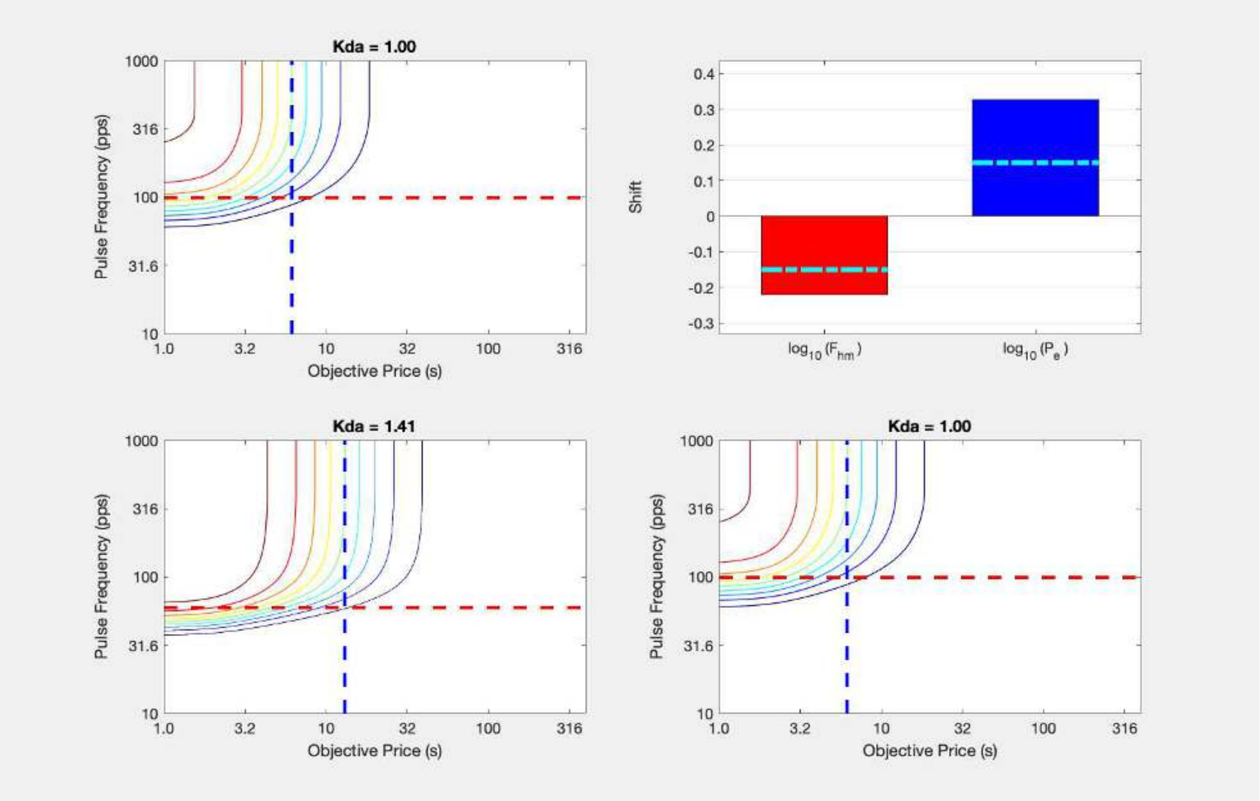


```
[fig_num, fig_tab] = add_fig(fig_num, fig_tab, "kDA1vskDA2_sc_quad", ...
    "Effect of dopamine-transporter blockade on the reward mountain");
```

```
This is figure #44: (kDA1vskDA2_sc_quad)
```

```
toc
```

```
Elapsed time is 9.650534 seconds.
```

```
tic
clear -regexp ^Cont ^dual ^MTN ^quad;
```

The following two figures compare the shifts in the position of the reward mountain predicted by the series-circuit model and observed empirically when eICSS is challenged with GBR-12909, a dopamine-transporter blocker:

```
GBR_eICSS_summary = make_fig_from_png(fullfile(ImpFigDir,'GBR_eICSS_summary.png'), 100)
GBReICSSVSsimBG = dual_subplot(GBR_eICSS_summary, bg_kDA1vskDA2sc, 'GBReICSSVSsimBG',..
    graphs2files,FigDir);
% rescale the right panel to 60% width, 80% height & center in right panel
GBReICSSVSsimBGrs = adjust_right_panel(GBReICSSVSsimBG, 0.75, 1);
if show_graphics
    GBReICSSVSsimBGrs.Visible = 'on';
end
```

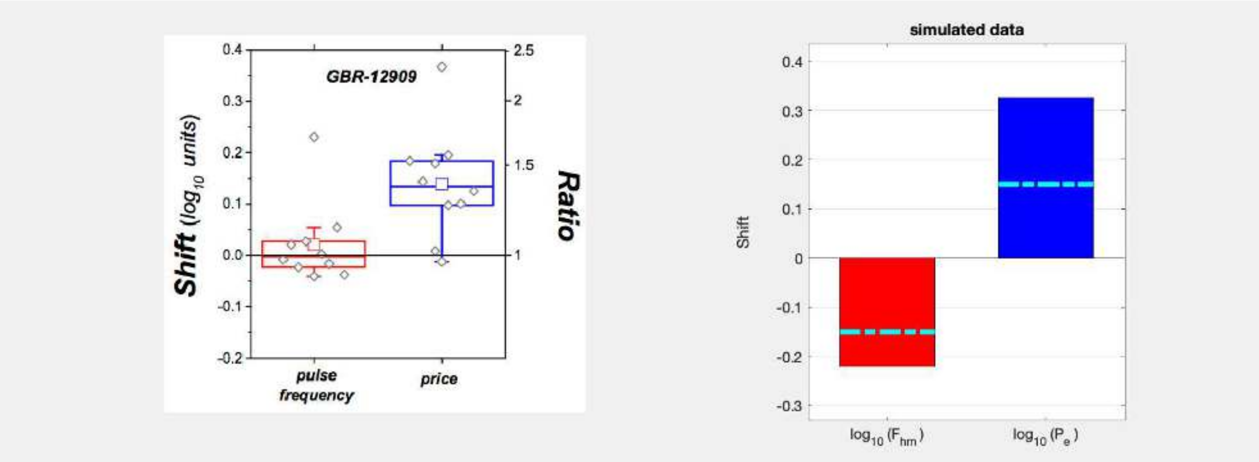

The predicted shift along the price axis is consistent qualitatively with the empirical results of the experiment in which eICSS was challenged with GBR-12909: the mountain shifts rightwards along the price axis. However, the predicted shift along the pulse-frequency axis is inconsistent with the empirical results. Whereas the series-circuit model predicts a leftward shift along the pulse-frequency axis, the mountain was not displaced systematically along the pulse-frequency axis in the empirical study. This discrepancy reveals a serious flaw in the series-circuit model.

The reason for the failure of the series-circuit model becomes clear upon inspection of

```
disp(string({strcat({'Figure '},num2str(fig_tab.Number(fig_tab.Name=='SeriesCircuit_eIC
```

```
Figure 42
```

The change in dopaminergic neurotransmission produced by GBR-12909 rescales the input to the downstream reward-growth function (to the right of the dopamine neurons), changing its position parameter. In contrast to

what is observed empirically in the eICSS studies, this causes the simulated mountain to shift along the pulse-frequency axis, regardless of whether the dopamine neurons are activated directly by optical stimulation or indirectly by synaptic input from the medial forebrain bundle neurons stimulated by an electrode.

An observer positioned at the input to the downstream reward-growth function cannot know whether optical or electrical stimulation was responsible for the phasic dopamine signal that constitutes the input to this function. Thus, the series-circuit model cannot readily generate differential predictions in response to optical and electrical inputs.

```
toc
```

```
Elapsed time is 1.282094 seconds.
```

The qualitative predictions don't depend meaningfully on the value of the parameter than scales the medial forebrain bundle drive on the dopamine neurons.

```
tic;
clear -regexp ^GBR;
if show_graphics
    close all;
end
```

The next set of graphs shows that the predictions don't change qualitatively when the maximum medial forebrain bundle drive on the dopamine neurons is reduced: The mountain continues to shift along both the price and pulse-frequency axes

```
KrgEqLo = 41; % Maximum MFB drive is reduced
% n.b. KrgEqLo must be sufficiently exceed FdaHMkDA so as to keep FscHMlo below the max
% Otherwise the back-solution function will generate an error message and return.

% Re-calculate the location-parameter values along the pulse-frequency axis for
% the series-circuit model as a whole.
FFmax = FilterFun(FPmax, FmfbBend, FmfbRO);
if FFmax > LogistNormBsFun(gMFB,FmfbHM,max(FdaHMkDA)/KrgEqLo)
    for j=1:length(FdaHMkDA)
        FscHMlo(j) = FscHMbs(FdaHMkDA(j), FmfbBend, FmfbHM, FmfbRO, FPmax, gMFB, KrgEqL
    end
else
    display("FscHM could not be calculated because KrgEqLo is too low.");
    display("Set KrgEqLo such that FFmax > FilterFun(FPmax, Fbend, Fro).");
    return
end
```

```
FscHMlo = 380.5649
FscHMlo = 1×2
  380.5649  103.3750
```

```

% Recalculate the drive on the DA neurons
FoptEquivLo = FoptEquivFun(Felec, FmfbBend, FmfbHM, FmfbRO, gMFB, KrgEqLo);
FPmaxEq = max(FoptEquivLo) % This is the highest equivalent optical pulse frequency tha
```

```
FPmaxEq = 37.7159
```

```
RnormMax = fRbsrNorm(FPmaxEq, FdaBend, FdaHMkDA, FdaRO, gDA)
```

```
RnormMax = 1×2
    0.2640    0.7290
```

```
PobjE = PobjEfun(dotPhiObj, Kaa, Kec, Krg, dotRaa, pObj, PsubBend, PsubMin, RnormMax, K
```

```
PobjE = 1×2
    2.5324   10.2974
```

```
% Compute time allocation for the vehicle and drug conditions
Tsc3 = TAfun(a, FoptEquivLo, FdaBend, FdaHMkDA(1), FdaRO, gDA, Pobj, ...
    PobjE(1), PsubBend, PsubMin, RnormMax(1));
Tsc4 = TAfun(a, FoptEquivLo, FdaBend, FdaHMkDA(2), FdaRO, gDA, Pobj, ...
    PobjE(2), PsubBend, PsubMin, RnormMax(2));
title_str1 = strcat({'Kda = '}, sprintf('%2.2f', Kda(1)));
MTNkDA1scKrgEqLo = plot_MTN(Felec, Pobj, Tsc3, 'off', 'MTNkDA1scKrgEqLo', title_str1, .
    graphs2files, FigDir);
title_str2 = strcat({'Kda = '}, sprintf('%2.2f', Kda(2)));
MTNkDA2scKrgEqLo = plot_MTN(Felec, Pobj, Tsc4, 'off', 'MTNkDA2scKrgEqLo', title_str2, .
    graphs2files, FigDir);
MTNkDA1vskDA2scKrgEqLo = dual_subplot(MTNkDA1scKrgEqLo, MTNkDA2scKrgEqLo, 'MTNkDA1vskDA
    graphs2files,FigDir);
if show_graphics
    MTNkDA1vskDA2scKrgEqLo.Visible = 'on';
end
```

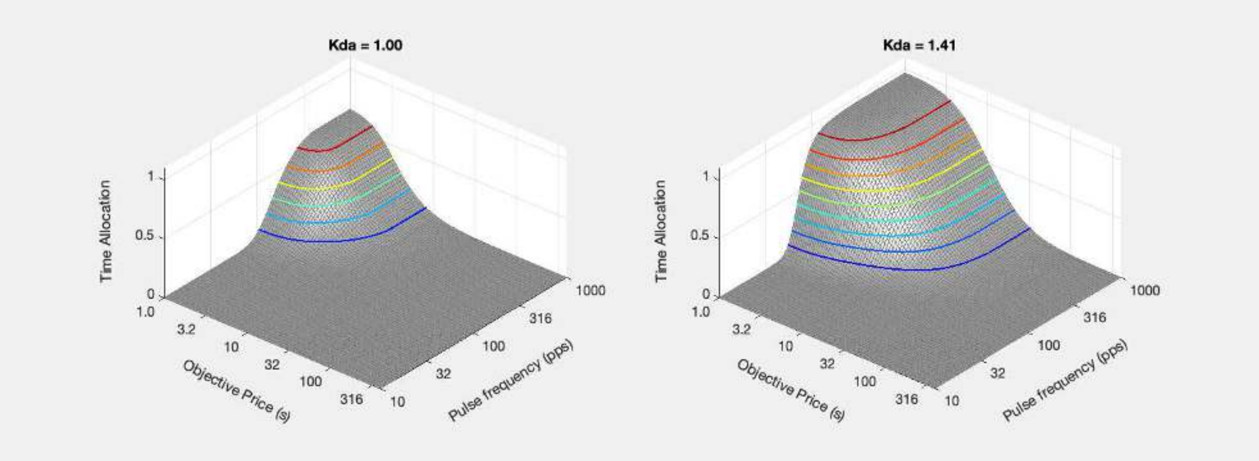


```
[fig_num, fig_tab] = add_fig(fig_num, fig_tab, "kDA1vskDA2_scKrgEqLo_mtns", ...
    "Effect of dopamine-transporter blockade on the reward mountain");
```

```
This is figure #45: (kDA1vskDA2_scKrgEqLo_mtns)
```

```
FscHMstar = FilterFun(FdaHMkDA,FdaBend,FdaRO);
% Computed on the basis of frequency following in the dopamine neurons

PsubE = PsubFun(PobjE,PsubBend,PsubMin);
```

```
PsubEstar = PsubE ./ RnormMax;
PobjEstar = PsubBsFun(PsubEstar,PsubBend,PsubMin);

ContkDA1scKrgEqLo = plot_contour(Felec, Pobj, Tsc3, PobjE(1), FscHMlo(1), 'off', 'Contk
    strcat({'Kda = '}, num2str(Kda(1))), graphs2files, FigDir);
ContkDA2scKrgEqLo = plot_contour(Felec, Pobj, Tsc4, PobjE(2), FscHMlo(2), 'off', 'Contk
    strcat({'Kda = '}, num2str(Kda(2))), graphs2files, FigDir);
bg_kDA1vskDA2scKrgEqLo = plot_bgStar(FscHM(1), FscHM(2), FscHMstar(1), FscHMstar(2),...
    PobjE(1), PobjE(2), PobjEstar(1), PobjEstar(2),...
    'off', 'kDA1vskDA2scKrgEqLo_bg', graphs2files, FigDir);
bg_root = 'bg_kDA1vskDA2scKrgEqLo';
quad_kDA1vskDA2scKrgEqLo = quad_subplot(ContkDA1scKrgEqLo, ContkDA2scKrgEqLo, bg_kDA1vs
    'quad_kDA1vskDA2scKrgEqLo', bg_root, graphs2files, FigDir);
if show_graphics
    quad_kDA1vskDA2scKrgEqLo.Visible = 'on';
end
```

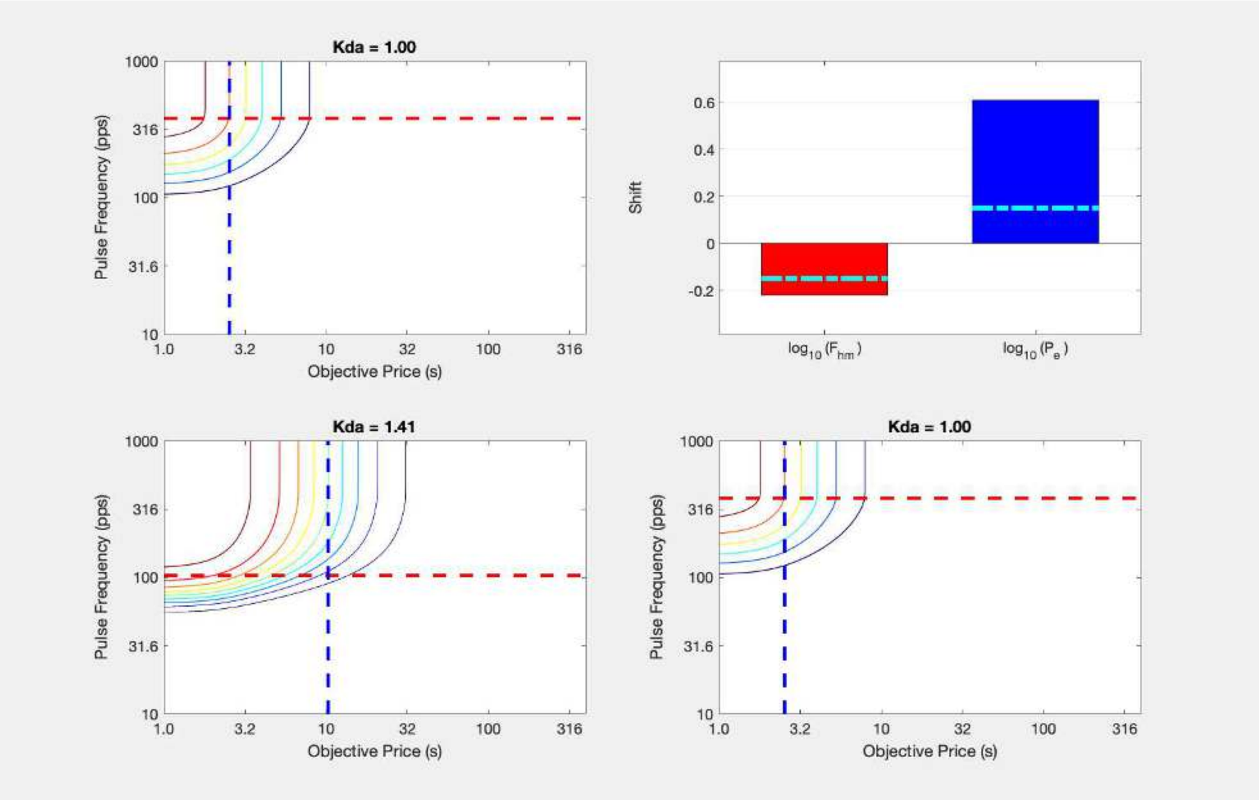


```
[fig_num, fig_tab] = add_fig(fig_num, fig_tab, "kDA1vskDA2_scKrgEqLo_quad", ...
    "Effect of dopamine-transporter blockade on the reward mountain");
```

```
This is figure #46: (kDA1vskDA2_scKrgEqLo_quad)
```

```
toc
```

```
Elapsed time is 9.818036 seconds.
```

```
tic;
clear -regexp ^bg ^Cont ^dual ^MTN ^quad;
close all;
```

Next, we increase the medial forebrain bundle drive so that some of it is wasted. The firing rate of the dopamine neurons now asymptotes at sub-maximal levels of medial forebrain bundle drive. Again, the predictions of the series-circuit model do not change qualitatively: The mountain continues to shift along both the price and pulse-frequency axes.

```
KrgEqHi = 126; % Maximum MFB drive exceeds the DA frequency-following range

% Re-calculate the location-parameter values along the pulse-frequency axis for
% the series-circuit model as a whole.
FFmax = FilterFun(FPmax, FmfbBend, FmfbRO);
if FFmax > LogistNormBsFun(gMFB,FmfbHM,max(FdaHMkDA)/KrgEqHi)
    for j=1:length(FdaHMkDA)
        FscHMhi(j) = FscHMbs(FdaHMkDA(j), FmfbBend, FmfbHM, FmfbRO, FPmax, gMFB, KrgEqH
    end
else
    display("FscHM could not be calculated because KrgEqHi is too low.");
    display("Set KrgEqHi such that FFmax > FilterFun(FPmax, Fbend, Fro).");
    return
end
```

```
FscHMhi = 44.9730
FscHMhi = 1×2
   44.9730   32.0551
```

```
% Recalculate the drive on the DA neurons
FoptEquivHi = FoptEquivFun(Felec, FmfbBend, FmfbHM, FmfbRO, gMFB, KrgEqHi);
FPmaxEq = max(FoptEquivHi) % This is the highest equivalent optical pulse frequency tha
```

```
FPmaxEq = 115.9075
```

```
RnormMax = fRbsrNorm(FPmaxEq, FdaBend, FdaHMkDA, FdaRO, gDA)
```

```
RnormMax = 1×2
    0.8186    0.9713
```

```
PobjE = PobjEfun(dotPhiObj, Kaa, Kec, Krg, dotRaa, pObj, PsubBend, PsubMin, RnormMax, K
```

```
PobjE = 1×2
    8.1862   13.7200
```

```

% Compute time allocation for the vehicle and drug conditions
Tsc5 = TAfun(a, FoptEquivHi, FdaBend, FdaHMkDA(1), FdaRO, gDA, Pobj, ...
    PobjE(1), PsubBend, PsubMin, RnormMax(1));
Tsc6 = TAfun(a, FoptEquivHi, FdaBend, FdaHMkDA(2), FdaRO, gDA, Pobj, ...
    PobjE(2), PsubBend, PsubMin, RnormMax(2));
title_str1 = strcat({'Kda = '}, sprintf('%2.2f', Kda(1)));
MTNkDA1scKrgEqHi = plot_MTN(Felec, Pobj, Tsc5, 'off', 'MTNkDA1scKrgEqHi', title_str1, .
    graphs2files, FigDir);
title_str2 = strcat({'Kda = '}, sprintf('%2.2f', Kda(2)));
MTNkDA2scKrgEqHi = plot_MTN(Felec, Pobj, Tsc6, 'off', 'MTNkDA2scKrgEqHi', title_str2, .
    graphs2files, FigDir);
MTNkDA1vskDA2scKrgEqHi = dual_subplot(MTNkDA1scKrgEqHi, MTNkDA2scKrgEqHi, 'MTNkDA1vskDA
    graphs2files,FigDir);
```

```
if show_graphics
    MTNkDA1vskDA2scKrgEqHi.Visible = 'on';
end
```

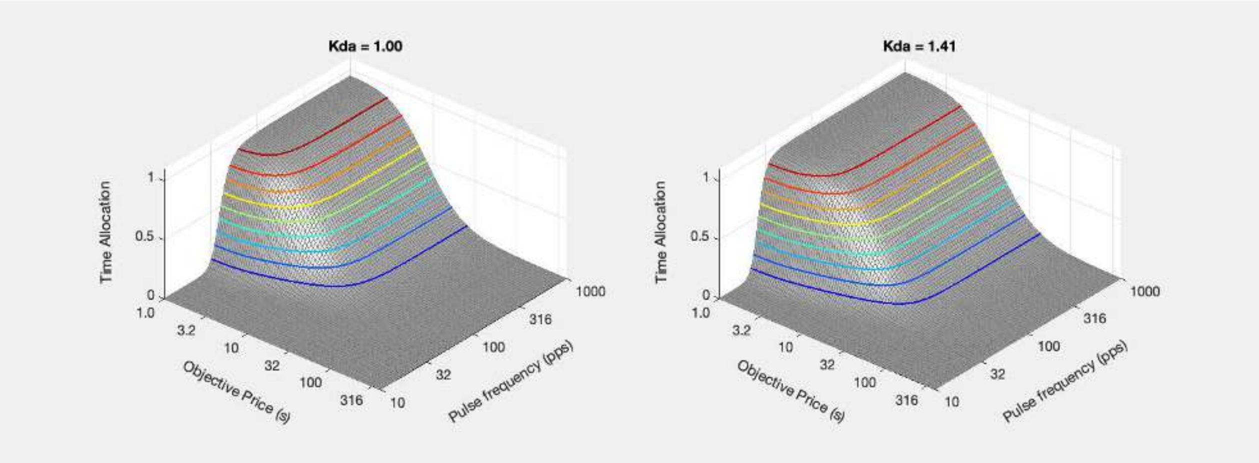


```
FscHMstar = FilterFun(FdaHMkDA,FdaBend,FdaRO);
% Computed on the basis of frequency following in the dopamine neurons

PsubE = PsubFun(PobjE,PsubBend,PsubMin);
PsubEstar = PsubE ./ RnormMax;
PobjEstar = PsubBsFun(PsubEstar,PsubBend,PsubMin);

ContkDA1scKrgEqHi = plot_contour(Felec, Pobj, Tsc5, PobjE(1), FscHMhi(1), 'off', 'Contk
    strcat({'Kda = '}, num2str(Kda(1))), graphs2files, FigDir);
ContkDA2scKrgEqHi = plot_contour(Felec, Pobj, Tsc6, PobjE(2), FscHMhi(2), 'off', 'Contk
    strcat({'Kda = '}, num2str(Kda(2))), graphs2files, FigDir);
bg_kDA1vskDA2scKrgEqHi = plot_bgStar(FscHM(1), FscHM(2), FscHMstar(1), FscHMstar(2),...
    PobjE(1), PobjE(2), PobjEstar(1), PobjEstar(2),...
    'off', 'kDA1vskDA2scKrgEqHi_bg', graphs2files, FigDir);
bg_root = 'bg_kDA1vskDA2scKrgEqHi';
quad_kDA1vskDA2scKrgEqHi = quad_subplot(ContkDA1scKrgEqHi, ContkDA2scKrgEqHi, bg_kDA1vs
    'quad_kDA1vskDA2scKrgEqHi', bg_root, graphs2files, FigDir);
if show_graphics
    quad_kDA1vskDA2scKrgEqHi.Visible = 'on';
end
```

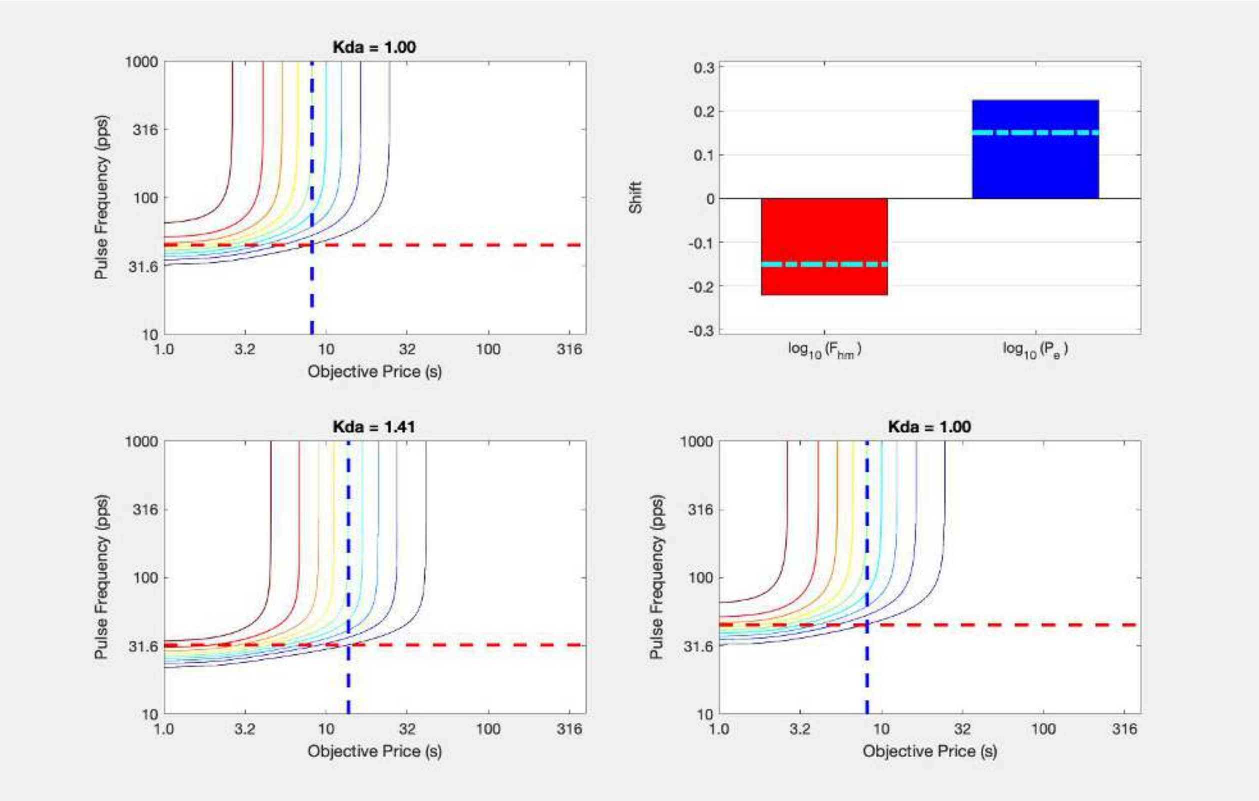


```
[fig_num, fig_tab] = add_fig(fig_num, fig_tab, "kDA1vskDA2_scKrgEqHi_quad", ...
    "Effect of dopamine-transporter blockade on the reward mountain");
```

```
This is figure #47: (kDA1vskDA2_scKrgEqHi_quad)
```

This section demontrates that the inconsistency between the simulated and observed results is seen over a broad range of values of the parameter that scales the MFB drive on the dopamine neurons.

The problem with the series-circuit model is a fundamental one. It predicts shifts of the mountain along the pulse-frequency axis in response to perturbation of dopaminergic neurotransmission. In contrast, systematic, consistent shifts along the pulse-frequency axis are not seen in eICSS studies under the influence of the dopamine transporter blocker, GBR-12909 (Hernandez et al. 2012); the dopamine, norepinephrine, and serotonin blocker, cocaine (Hernandez et al., 2010); the D2, D3 and 5HT7 receptor blocker, pimozide (Trujillo-Pisanty et al., 2014); or the cannabinoid CB-1 blocker, AM-251 (Trujillo-Pisanty et al., 2011). (AM-251 inhibits dopamine release and attenuates the stimulation-induced increase in dopamine tone (Trujillo-Pisanty et al., 2011)) . Thus, the series-circuit model fails to account for the eICSS data.

```
clearvars('-except',keepVars{:})
keepVars = who; % Restore cell array containing names of variables to be retained
toc
```

```
Elapsed time is 8.968485 seconds.
```

The convergence model

```
tic;
close all;
```

The failure of the series-circuit model to account readily for the differential movement of the reward mountain in the eICSS and oICSS studies motivates the search for an alternative. We investigate a new model here. The challenge is to account for the stability of the reward mountain along the pulse-frequency axis under dopaminergic challenge in the eICSS data, the observed displacement along the pulse-frequency axis in the oICSS data, and the observed displacement along the price axis in both datasets.

```
if show_graphics
    show_imported_graphic('eICSS_oICSS_logistic+3Pe_noMasks_v1.png',30,ImpFigDir)
end
```

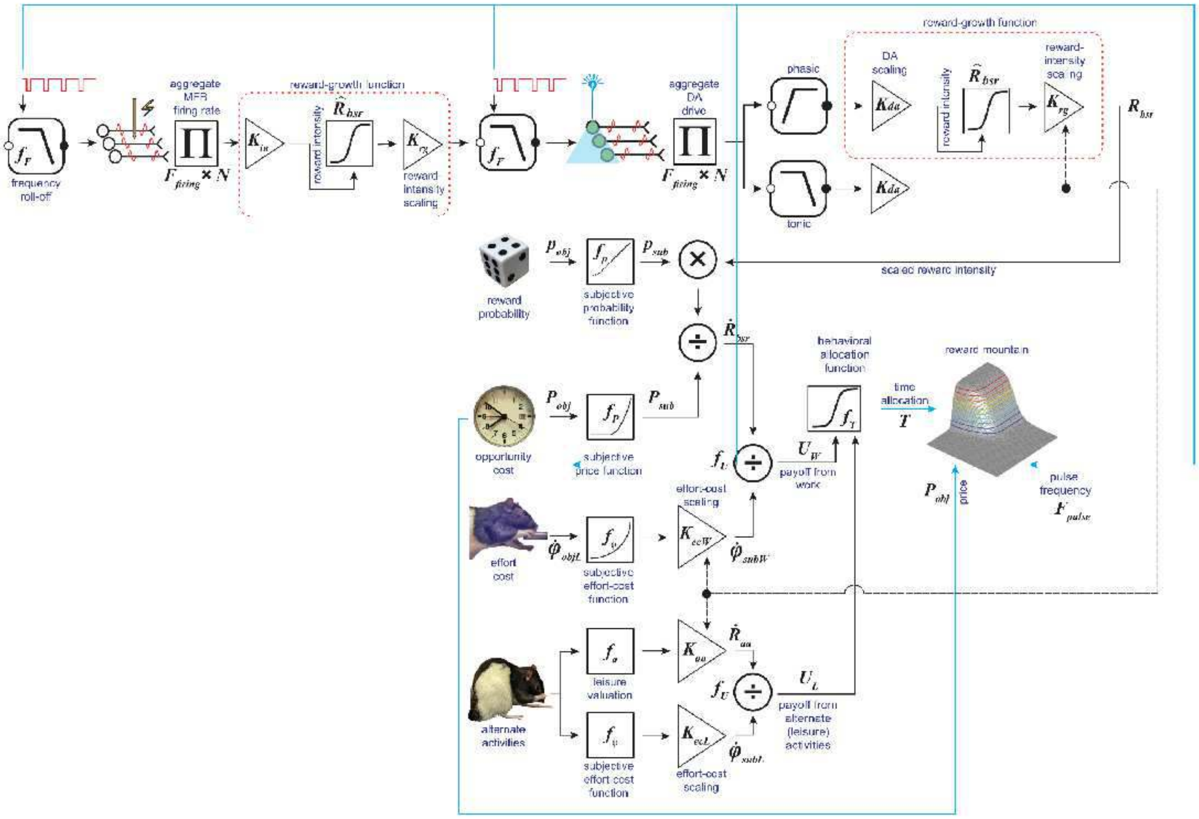


```
[fig_num, fig_tab] = add_fig(fig_num, fig_tab, "Convergence_model", ...
    "A model implementing converging pathways subserving oICSS and eICSS");
```

This is figure #48: (Convergence_model)

In this model, the circuitry underlying eICSS of the medial forebrain bundle and oICSS of midbrain dopamine neurons includes parallel stages that are linked at two levels. The parallel limbs subserve oICSS and eICSS between the directly stimulated neurons and the scaled output of the reward-growth functions. The upstream link between the two limbs relays to midbrain dopamine neurons input from neurons activated by electrical stimulation of the medial forebrain bundle. The downsteam link combines the outputs of the two limbs so as to produce the signal representing the benefit of the experimenter-controlled reward.

The input from the MFB to the midbrain dopamine neurons

It has long been known that electrical stimulation of the medial forebrain bundle provides trans-synaptic input to midbrain dopamine neurons (Maeda & Mogenson, 1980) and drives phasic dopamine release from their ventral striatal terminals (Gratton, Hoffer & Gerhardt, 1988; Yavich & Tiihonen, 2000a, 2000b; Wightman & Robinson, 2002; Yavitch & Tanila, 2007). Recently, Cossette, Conover & Shizgal (2016) demonstrated differences between the frequency-following characteristics of the MFB input to midbrain dopamine neurons that project to the medial shell of the nucleus accumbens and the frequency-following characteristics of the neurons subserving eICSS of the MFB. Whereas the neurons subserving the rewarding effect maintain high-fidelity frequency following up to ~360 pulses s^{-1} (Solomon et al., 2015), the input to the nucleus-accumbens projecting dopamine neurons fails to follow pulse frequencies greater than 130 pulses s^{-1}. To accommodate this difference, a second low-pass filter is inserted below the link between the medial forebrain bundle fibers and the spike counter to the left of the dopamine neurons in

```
disp(string({strcat({'Figure '},num2str(fig_tab.Number(fig_tab.Name=="Convergence_model
```

```
Figure 48
```

At pulse frequencies below 130 pulses s^{-1}, the amplitude of dopamine transients recorded in the nucleus accumbens in response to electrical stimulation of the medial forebrain bundle grows as function of both current and pulse frequency (Cossette, Conover & Shizgal, 2016). Increases in current can compensate for decreases in pulse frequency so as to hold constant the amplitude of the transient (and vice-versa). This suggests that with train duration held constant, the amplitude of the transient depends on the aggregate rate of firing in the medial forebrain bundle fibers that drive the activation of the dopamine neurons. That is the reason for the second spike counter.

As in the case of the series-circuit model, the trans-synaptic drive on the dopamine neurons is represented in terms of the optical pulse frequency that provides equivalent dopaminergic activation.

Summation between the outputs of the two parallel limbs

The convergence model retains the idea that there is a final common path for neural signals that encode the predicted benefits of reward procurement. The two parallel circuit limbs, one subserving eICSS and the other oICSS, converge on this final common path. A simple way to implement this convergence is to add the outputs of the two limbs. This proposal faces a seemingly daunting challenge: electrical stimulation of the MFB activates midbrain dopamine neurons. If so, one would expect such stimulation to drive signaling in both of the hypothesized converging pathways. Wouldn't this produce at least some displacement of the reward mountain along the pulse-frequency axis in response to dopamine-transporter blockade? We show below that this is not necessarily the case. Indeed, the simulations show that given reasonable assumptions and values drawn from the current data, a convergence model can replicate the eICSS findings.

The reward-intensity signal at the output of the eICSS limb of the circuit is computed as above, by applying

```
disp(string({strcat({'Function '},num2str(fun_tab.Number(fun_tab.Name=='fRbsrFull')))}))
```

```
Function 34
```

The reward-intensity signal at the output of the oICSS limb of the circuit is computed in the same manner as when the input consists of optical stimulation pulses. However, additional steps are required to compute the output of this limb during eICSS, when electrically excited medial forebrain bundle neurons provide the input

to the dopamine neurons. The electrical pulse frequency is translated in the input to the dopamine neurons (expressed in terms of the equivalent optical pulse frequency) by the function, *FmfbDAdriveFun.*

```
% [fun_num, fun_tab] = add_fun(fun_num, fun_tab, ...
%     "FmfbDAdriveFun", "FbendMFBda, Felec, FroMFBda, Kmfb, NnMFBs",...
%     "Function that translates MFB drive on the DA neurons into an equivalent optical
[fun_num, fun_tab] = add_fun(fun_num, fun_tab, ...
    "FFmfbDAbend", "Felec, FFmfbDAmax, FoptEquivVec, FmfbDAro",...
    "Function that translates MFB drive on the DA neurons into an equivalent optical pu
```

```
This is function #41: (FFmfbDAbend)
```

We now simulate the output of the model in response to electrical stimulation of the medial forebrain bundle.

```
% Reward intensity produced by the upper limb
C = 0.473; % median from Sonnenschein et al., 2003
Delec = 0.5;
logFelecBend = 1.3222; % from Solomon et al., 2015
FelecBend = 10^logFelecBend;
logFelecRO = 2.5587; % from Solomon et al., 2015
FelecRO = 10^logFelecRO;
NnMFB = 126;
RhoPi = 5000;
FPmax = 1000;
FhmUpper = FpulseHMfun(C, Delec, FelecBend, FPmax, FelecRO, NnMFB, RhoPi)
```

```
FhmUpper = 77.2222
```

```
numF = 121;
numParamVals = 6;
Felec = logspace(0,3,numF);
FelecMat = repmat(Felec,numParamVals,1);

gElec = 5;
KrgUpper = 1;
Rupper = fRbsr(Felec, FelecBend, FhmUpper, FelecRO, gElec, KrgUpper);
```

The position-parameter value for the upper limb (77 pulses s^{-1}) is typical for the eICSS studies entailing measurement of the reward mountain.

```
disp(string({strcat({'Figure '},num2str(fig_tab.Number(fig_tab.Name=='RGfunsElecFhm')))
```

```
Figure 3
```

```
TitleStrSemi = 'Upper-limb reward intensity';
pnam = "Fhm";
fnam = "Fhm";
% The data to be plotted must be in columns. Thus, FelecMat and RelecMat are transposed
RG_MFBda_upper_semilog = plot_RG(Felec',Rupper',pnam,FhmUpper,fnam,TitleStrSemi,'lin',
axh = findall(RG_MFBda_upper_semilog,'Type','Axes');
axh.XLabel.String = "Electrical pulse frequency";
axh.YLabel.String = "Upper-limb reward intensity";
```

```
if show_graphics
    RG_MFBda_upper_semilog.Visible = 'on';
end
```

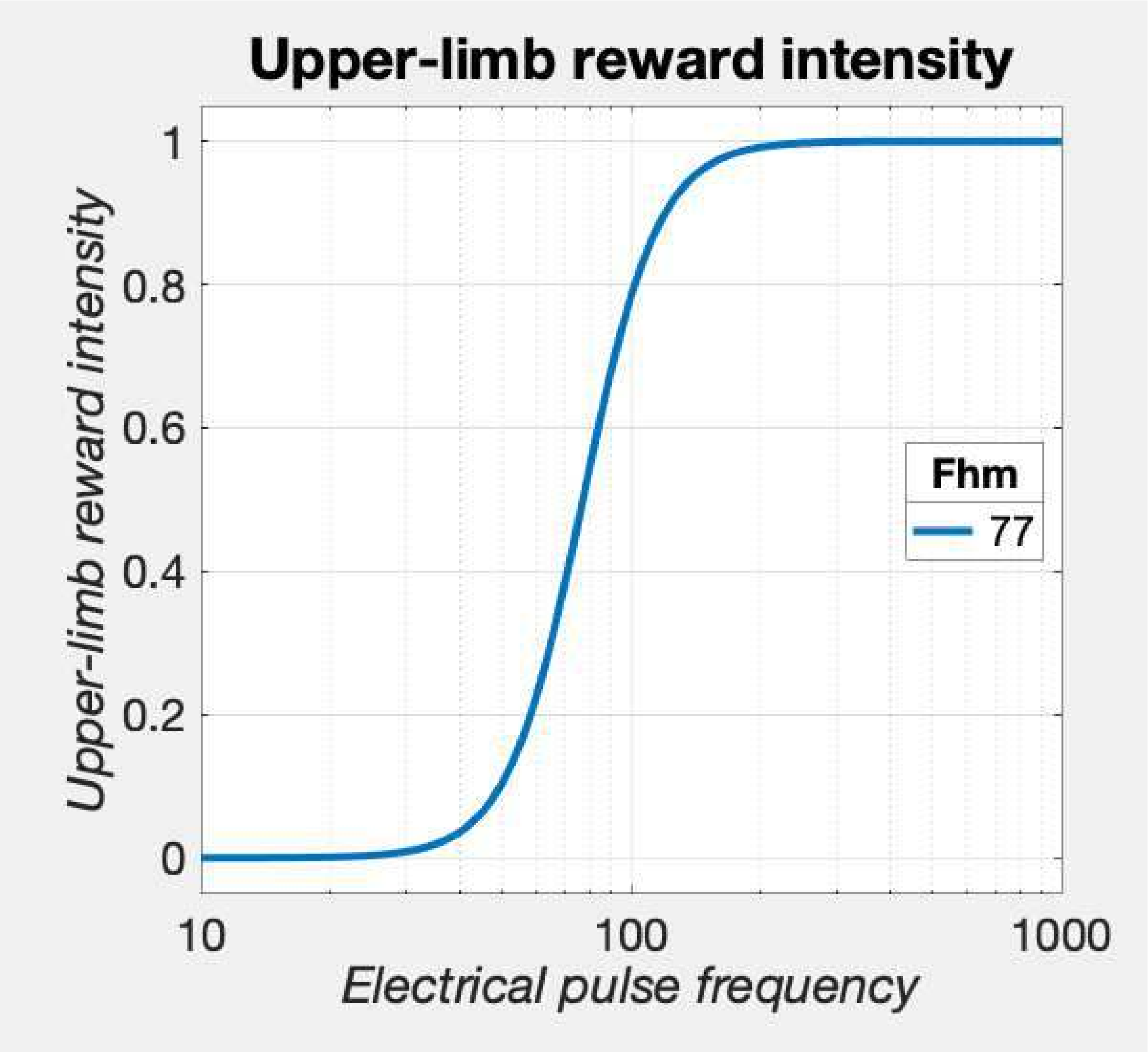


```
[fig_num, fig_tab] = add_fig(fig_num, fig_tab, "RG_upper_semilog", ...
    "Growth of upper-limb reward intensity");
```

```
This is figure #49: (RG_upper_semilog)
```

Reward-intensity growth in the lower limb depends on the strength of the MFB drive,

```
% Reward intensity produced by the lower limb
% First compute the drive on the DA neurons in terms of the equivalent optical pulse fr
FmfbDAbend = 40; % Make this gradual to match data in Cossette et al., 2016
FmfbDAro = 125; % Frequency following as described by Cossette et al., 2016
FFmfbDAmax = FilterFun(FPmax,FmfbDAbend, FmfbDAro);
FoptEquivVec = [5;10;20;40;80;160];
FoptEquivMat = repmat(FoptEquivVec, 1, numF);

FmfbDAdriveMat = FmfbDAdriveFun(FmfbDAbend, Felec, FFmfbDAmax, FoptEquivVec, FmfbDAro);
```

which is shown here:

```
% Columnar data are required to plot multiple lines. Thus the input matrices have been
FF_graphMFBda = plot_freqFoll(FelecMat',FmfbDAdriveMat','MFB_DA',1,1000,1,250);
% modify graph
lgnd = legend(num2str(FoptEquivVec),'Location','best');
lgnd.Title.String = "FoptEquiv";
axh = findall(FF_graphMFBda,'Type','Axes');
axh.XLabel.String = "Electrical pulse frequency";
axh.YLabel.String = "Equivalent optical pulse frequency";
if show_graphics
    FF_graphMFBda.Visible = 'on';
end
```

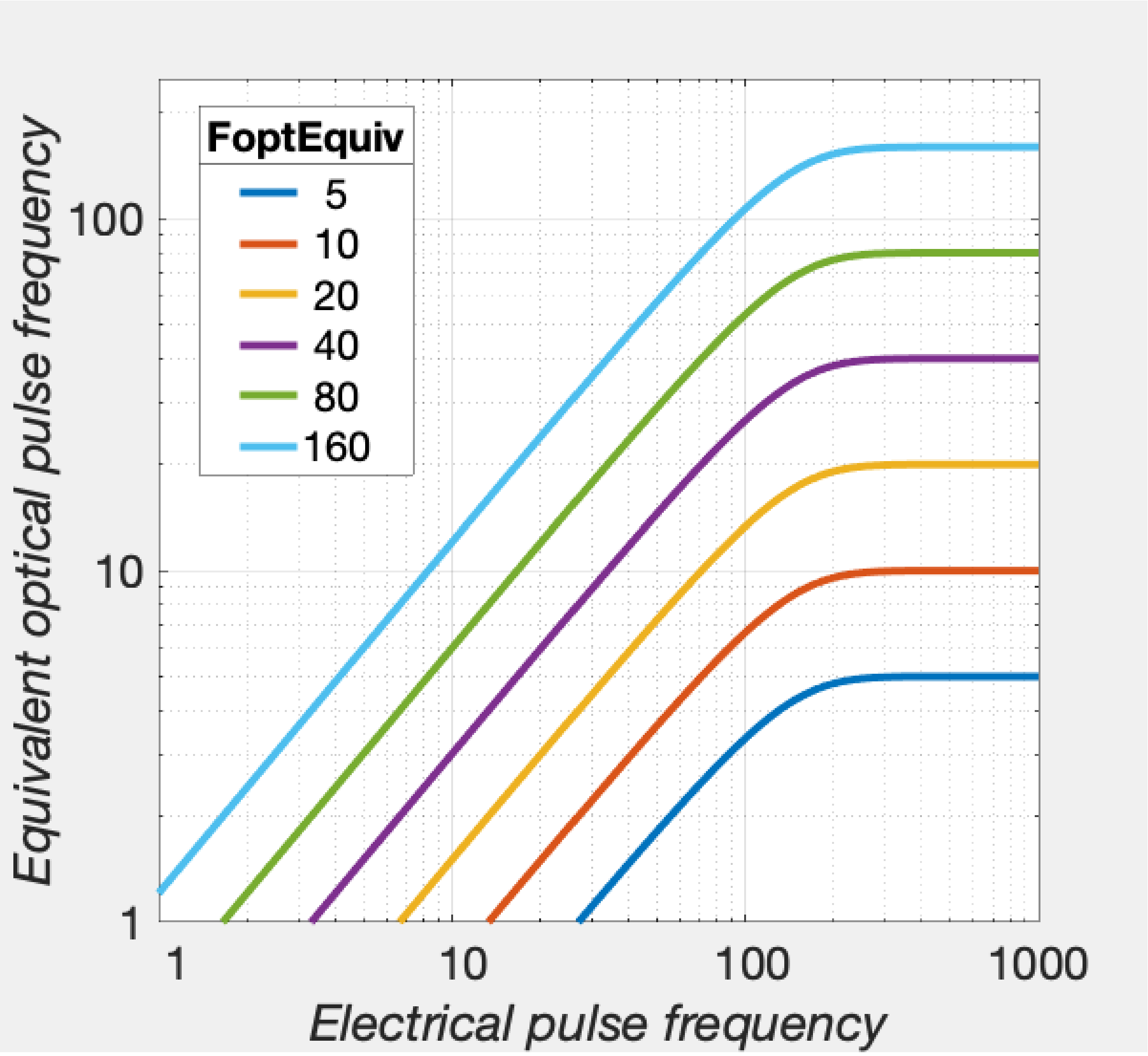


```
[fig_num, fig_tab] = add_fig(fig_num, fig_tab, "FreqFolMFBda", ...
    "Electrically induced firing frequency in MFB input to the dopamine neurons");
```

```
This is figure #50: (FreqFolMFBda)
```

```
D = Delec;
```

```
FdaBend = 20;
FdaRO = 50;
gDA = 5;
KrgLower = 1;
NnDA = 100; % Same value as in the series-circuit section
RhoPiDA = 1572; % Same value as in the series-circuit section for D = 0.5
FPmax = 1000;
FdaHM = FpulseHMfun(C, D, FdaBend, FPmax, FdaRO, NnDA, RhoPiDA)
```

```
FdaHM = 37.6342
```

The FdaHM parameter positions the reward-growth curve for the dopamine neurons along the axis representing the dopamine firing frequency:

```
logFopt = 0:0.025:2.4;
Fopt = 10.^logFopt;
Rlower = fRbsr(Fopt, FdaBend, FdaHM, FdaRO, gDA, KrgLower);

TitleStrSemi = 'Lower limb reward-intensity';
pnam = "FdaHM";
fnam = "FdaHM";
% The data to be plotted must be in columns. Thus, FelecMat and RelecMat are transposec
RG_DA_LowerEquiv_semilog = plot_RG(Fopt',Rlower',pnam,FdaHM,fnam,TitleStrSemi,'lin', 'I
RG_DA_LowerEquiv_semilog = modify_2D_graph(RG_DA_LowerEquiv_semilog, 'Axes', 'XLim', [1
    'RG_FdaHM_LowerEquiv_semilog', graphs2files, FigDir);
axh = findall(RG_DA_LowerEquiv_semilog,'Type','Axes');
axh.XLabel.String = "Equivalent optical pulse frequency";
axh.YLabel.String = "Lower-limb reward intensity";
if show_graphics
    RG_DA_LowerEquiv_semilog.Visible = 'on';
end
```

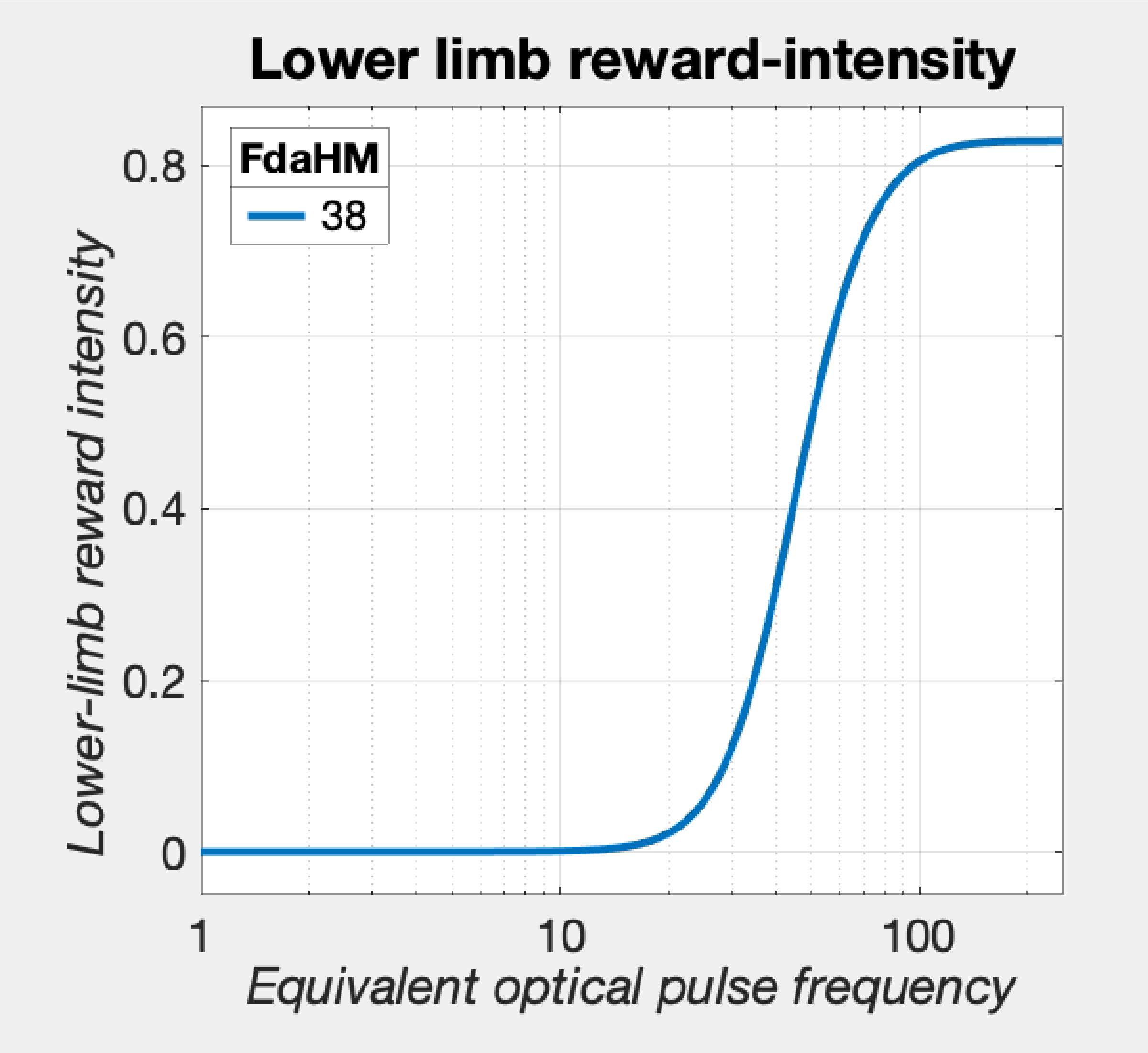


```
[fig_num, fig_tab] = add_fig(fig_num, fig_tab, "RG_DA_LowerEquiv_semilog", ...
    "Lower-limb reward intensity as a function of equivalent optical pulse frequencies'
```

```
This is figure #51: (RG_DA_LowerEquiv_semilog)
```

In the following graph, we see how reward-intensity grows in the lower limb as a function of the ***electrical*** pulse frequency.

```
Rlower = fRbsr(FmfbDAdriveMat, FdaBend, FdaHM, FdaRO, gDA, KrgLower);

TitleStrSemi = 'Lower-limb reward intensity';
pnam = "optDAeq";
fnam = "optDAeq";
% The data to be plotted must be in columns. Thus, FelecMat and RelecMat are transposec
RG_MFBda_lower_semilog = plot_RG(FelecMat',Rlower',pnam,FoptEquivVec,fnam,TitleStrSemi,
RG_MFBda_lower_semilog = modify_2D_graph(RG_MFBda_lower_semilog, 'Axes', 'XLim', [5,10(
    'RG_optDAeq_lower_semilog', graphs2files, FigDir);
axh = findall(RG_MFBda_lower_semilog,'Type','Axes');
axh.XLabel.String = "Electrical pulse frequency";
axh.YLabel.String = "Lower-limb reward intensity";
```

```
if show_graphics
    RG_MFBda_lower_semilog.Visible = 'on';
end
```

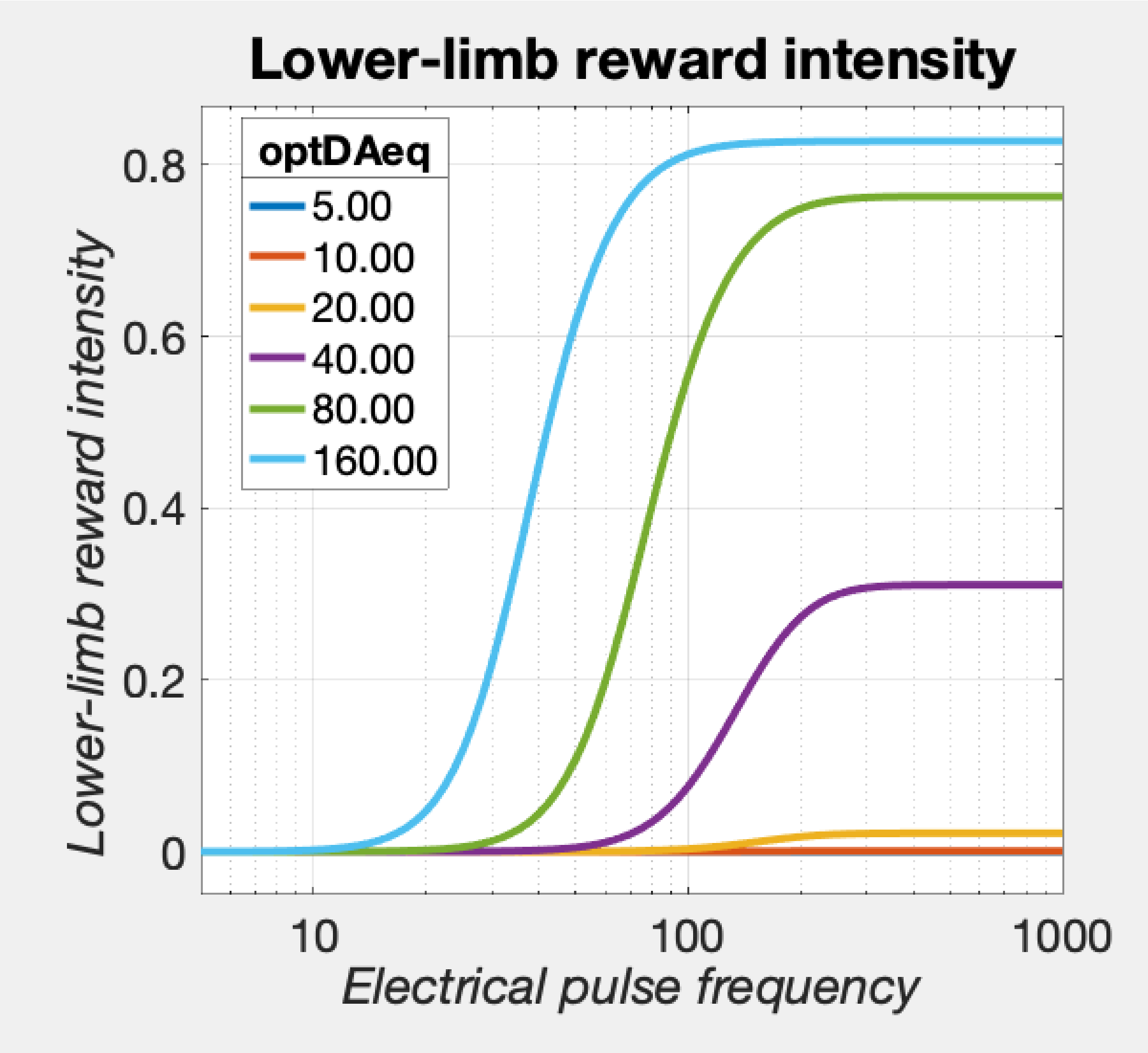


```
[fig_num, fig_tab] = add_fig(fig_num, fig_tab, "RG_lower_semilog", ...
    "Growth of lower-limb reward intensity at multiple values of Kmfb");
```

```
This is figure #52: (RG_lower_semilog)
```

The *FmfbDAdrive* function expresses the MFB drive on the dopamine neurons in units of equivalent optical pulses, i.e., an FmfbDAdrive of 25 means that the effect of the trans-synaptic input from the MFB is equivalent to that produced by 25 pulses s^{-1} of direct optical stimulation.

The graph above shows that strong input is required to generate a substantial response from the lower limb. The reason for this is that the $F_{pulse_{hm}}$ value is fairly close to the assumed frequency-following limit of the dopamine cells. The parameter values used in this simulation yield a $F_{pulse_{hm}}$ value close to the observed median in the current study when the train duration was 1 s. An adjustment is then made to predict model

output for a train duration of 0.5 s, which was used in the eICSS work. This calculation are described above (see: *The location parameter of the reward-growth function for BSR*).

Growth curves for the summated reward intensity are shown here, at different values of the strength of the MFB drive on the dopamine neurons. The equivalent optical pulse frequencies (*FoptEquiv*) are the maximum values that the simulated MFB drive can achieve.

```
Rupper = repmat(Rupper,numParamVals,1); % add rows to Rupper so that it is the same siz
Rsum = Rupper + Rlower;

TitleStrSemi = 'Summated reward intensity';
pnam = "FoptEquiv";
fnam = "MFBsum";
% The data to be plotted must be in columns. Thus, FelecMat and RelecMat are transposec
RG_MFBda_semilog = plot_RG(FelecMat',Rsum',pnam,FoptEquivVec,fnam,TitleStrSemi,'lin');
RG_MFBda_semilog = modify_2D_graph(RG_MFBda_semilog, 'Axes', 'XLim', [5,1000], ...
    'RG_MFBda_semilog', graphs2files, FigDir);
axh = findall(RG_MFBda_semilog, 'Type', 'Axes');
axh.XLabel.String = "Electrical pulse frequency";
axh.YLabel.String = "Upper + lower reward intensity";
if show_graphics
    RG_MFBda_semilog.Visible = 'on';
end
```

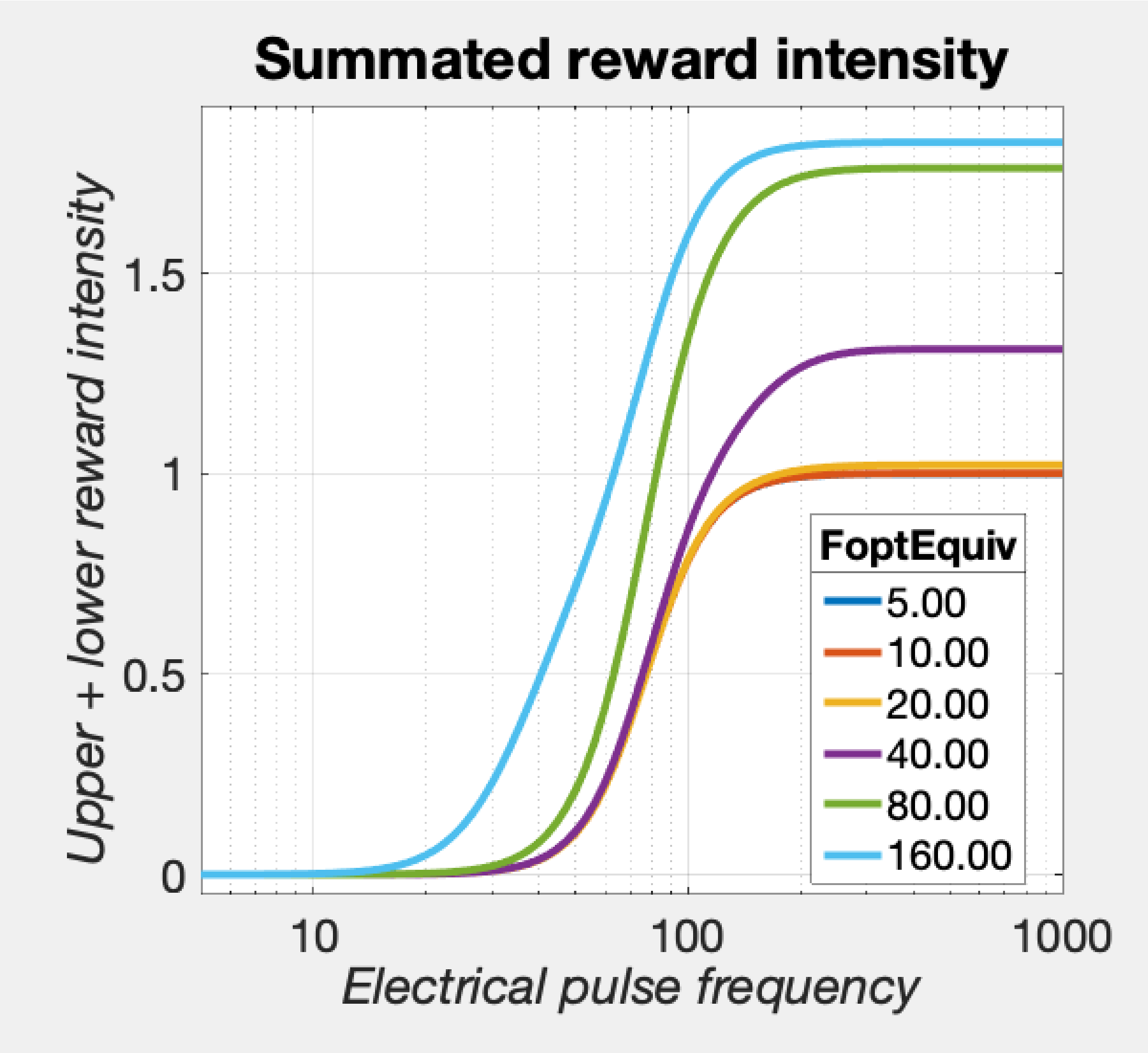

The reduced slope of the early portion of the leftmost curve is due to the fact that reward intensity in the lower limb begins rising at lower MFB pulse frequencies than reward intensity in the upper limb when the drive on the dopamine neurons is highest.

```
% Section break here to force display of RG_MFBda_semilog
```

```
close all;
[fig_num, fig_tab] = add_fig(fig_num, fig_tab, "RG_Kmfb_semilog", ...
    "Growth of summated reward intensity at at multiple values of Kmfb");
```

```
This is figure #53: (RG_Kmfb_semilog)
```

To prepare for simulation of the mountain generated by the convergence model, we first derive the electrical pulse frequencies that produce half-maximal, summated reward intensity for each value of *FoptEquiv*

```
% Find FsumHM by means of interpolation
numF = 121;
Felec = logspace(0,3,numF);
[FsumHM, RsumMax] = find_FhmSum(Felec, Rsum);
```

We generate a mountain surface, scaling the maximum MFB drive on the dopamine neurons to be equivalent to an optical pulse frequency of 80 pulses s^{-1}, a maximal or near-maximal value in the current study.

```
disp(string({strcat({'Figure '},num2str(fig_tab.Number(fig_tab.Name=='RG_Kmfb_semilog')
```

```
Figure 53
```

```
FoptEquiv = 80;
RupperFoptEquiv = Rupper(FoptEquivVec==FoptEquiv,:); % Selects the appropriate row from
RupperMax = max(RupperFoptEquiv);
RlowerFoptEquiv = Rlower(FoptEquivVec==FoptEquiv,:); % Selects the appropriate row from
RlowerMax = max(RlowerFoptEquiv);

Rsum = Rsum(FoptEquivVec==FoptEquiv,:);
FsumHM = FsumHM(FoptEquivVec==FoptEquiv);
RsumMax = RsumMax(FoptEquivVec==FoptEquiv);

FPmax = 1000;
RupperNormMax = fRbsrNorm(FPmax, FelecBend, FhmUpper, FelecRO, gElec);
RlowerNormMax = fRbsrNorm(FoptEquiv, FdaBend, FdaHM, FdaRO, gDA);
RupperRatio =  RupperMax / (RlowerMax + RupperMax)
```

```
RupperRatio = 0.5671
```

```
RlowerRatio =  RlowerMax / (RlowerMax + RupperMax)
```

```
RlowerRatio = 0.4329
```

```
RsumNormMax = (RupperRatio * RupperNormMax) + (RlowerRatio * RlowerNormMax); % Weighted

dotPhiObj = 1;
Kaa = 1;
Kec = 1;
Krg = 1;
dotRaa = 0.1;
pObj = 1;
PsubBend = 0.5;
PsubMin = 1.82;

PobjE = PobjEfun(dotPhiObj, Kaa, Kec, Krg, dotRaa, pObj, PsubBend, PsubMin, RsumNormMax
```

```
PobjE = 8.9716
```

```
a = 3;
numP = numF;
Pobj = logspace(0,3,numP); % row variable
Tsum = TAsumFun(a, Pobj, PobjE, PsubBend, PsubMin, Rsum', RsumNormMax);
% n.b., numel(Rsumxx) = numel(Pobj). Rsumxx has been transposed. Thus, Tsumxx is a squa

MTNsum = plot_MTN(Felec, Pobj, Tsum, 'off', strcat('MTNsum',num2str(FoptEquiv)), 'rewar
    graphs2files, FigDir);
if show_graphics
    MTN.Visible = 'on';
end
```

We now simulate the effect of dopamine-transporter blockade.

```
clearvars('-except',keepVars{:})
keepVars = who; % Restore cell array containing names of variables to be retained
toc
```

```
Elapsed time is 11.751399 seconds.
```

```
tic;
close all;
```

<u>The effect of dopamine transporter blockade in the convergence model</u>

The reward-intensity signal produced by the upper and lower limbs of the circuit shown in

```
disp(string({strcat({'Figure '},num2str(fig_tab.Number(fig_tab.Name=="Convergence_model
```

```
Figure 48
```

will first be computed separately and then combined additively, with two different values of K_{da}, one representing the vehicle condition, and the second representing the condition in which the dopamine-transporter blocker has been administered .

```
% Reward intensity produced by the upper limb
C = 0.473; % median from Sonnenschein et al., 2003
Delec = 0.5;
logFelecBend = 1.3222; % from Solomon et al., 2015
FelecBend = 10^logFelecBend;
logFelecRO = 2.5587; % from Solomon et al., 2015
FelecRO = 10^logFelecRO;
NnMFB = 126;
RhoPi = 5000;
FPmax = 1000;
FhmUpper = FpulseHMfun(C, Delec, FelecBend, FPmax, FelecRO, NnMFB, RhoPi)
```

```
FhmUpper = 77.2222
```

```
numF = 121;
numParamVals = 2; % for two values of Kda
Felec = logspace(0,3,numF);
FelecMat = repmat(Felec,numParamVals,1);
gElec = 5;
KrgUpper = 1;
Rupper = fRbsr(Felec, FelecBend, FhmUpper, FelecRO, gElec, KrgUpper);

% Reward intensity produced by the lower limb
% First compute the drive on the DA neurons in terms of the equivalent optical pulse fr
FmfbDAbend = 40;
FmfbDAro = 125;
FFmfbDAmax = FilterFun(FPmax,FmfbDAbend, FmfbDAro);
FoptEquiv = 80; % maximal or near-maximal value in the current study
FmfbDAdrive = FmfbDAdriveFun(FmfbDAbend, Felec, FFmfbDAmax, FoptEquiv, FmfbDAro);
```

```
% Calculate FdaHM for the vehicle and drug conditions
C = 0.473; % median from Sonnenschein et al., 2003
D = Delec;
FdaBend = 20;
FdaRO = 50;
Kda = [1,10^0.15]; % Median for current oICSS study: 10^0.1446
gDA = 5; % Median in vehicle condition of current oICSS study: 4.61
KrgLower = 1;
NnDA = 100; % Make sure to use the same value as in the series-circuit section
RhoPiDA = 1572; % Same value as in the series-circuit section for D=0.5
FPmax = 1000;
FdaHMkDA = FpulseHMfun(C, D, FdaBend, FPmax, FdaRO, NnDA, RhoPiDA, Kda) % result is a t
```

```
FdaHMkDA = 1×2
   37.6342   25.1515
```

```
% Compute reward growth in the lower limb as a function of the equivalent optical pulse
numFOpt = 97; % 0.025 log10 spacing from 0 to 2.4
numParamVals = 2; % 2 values of Kda
Fopt = logspace(0,2.4,numFOpt);
FoptMat = repmat(Fopt,numParamVals,1); % number of rows = number of Kda vals
KdaMat = repmat(Kda',1,numFOpt); % make matrix the same size as FoptMat
FdaHMmat = repmat(FdaHMkDA',1,numFOpt); % make matrix the same size as FoptMat
Rlower = fRbsr(FoptMat, FdaBend, FdaHMmat, FdaRO, gDA, KrgLower);

TitleStrSemi = 'Lower limb vs Fopt equivalent';
pnam = "Kda";
fnam = "Kda";
% The data to be plotted must be in columns. Thus, FoptMat and Rlower are transposed.
RG_MFB_DA_FoptEquiv_Kda = plot_RG(FoptMat',Rlower',pnam,Kda,fnam,TitleStrSemi,'lin', 'F
RG_MFB_DA_FoptEquiv_Kda = modify_2D_graph(RG_MFB_DA_FoptEquiv_Kda, 'Axes', 'XLim', [1,2
    'RG_Kda_FoptEquiv_semilog', graphs2files, FigDir);
axh = findall(RG_MFB_DA_FoptEquiv_Kda, 'Type','Axes');
axh.XLabel.String = "Equivalent optical pulse frequency";
axh.YLabel.String = "Lower-limb reward intensity";
if show_graphics
    RG_MFB_DA_FoptEquiv_Kda.Visible = 'on';
end
```

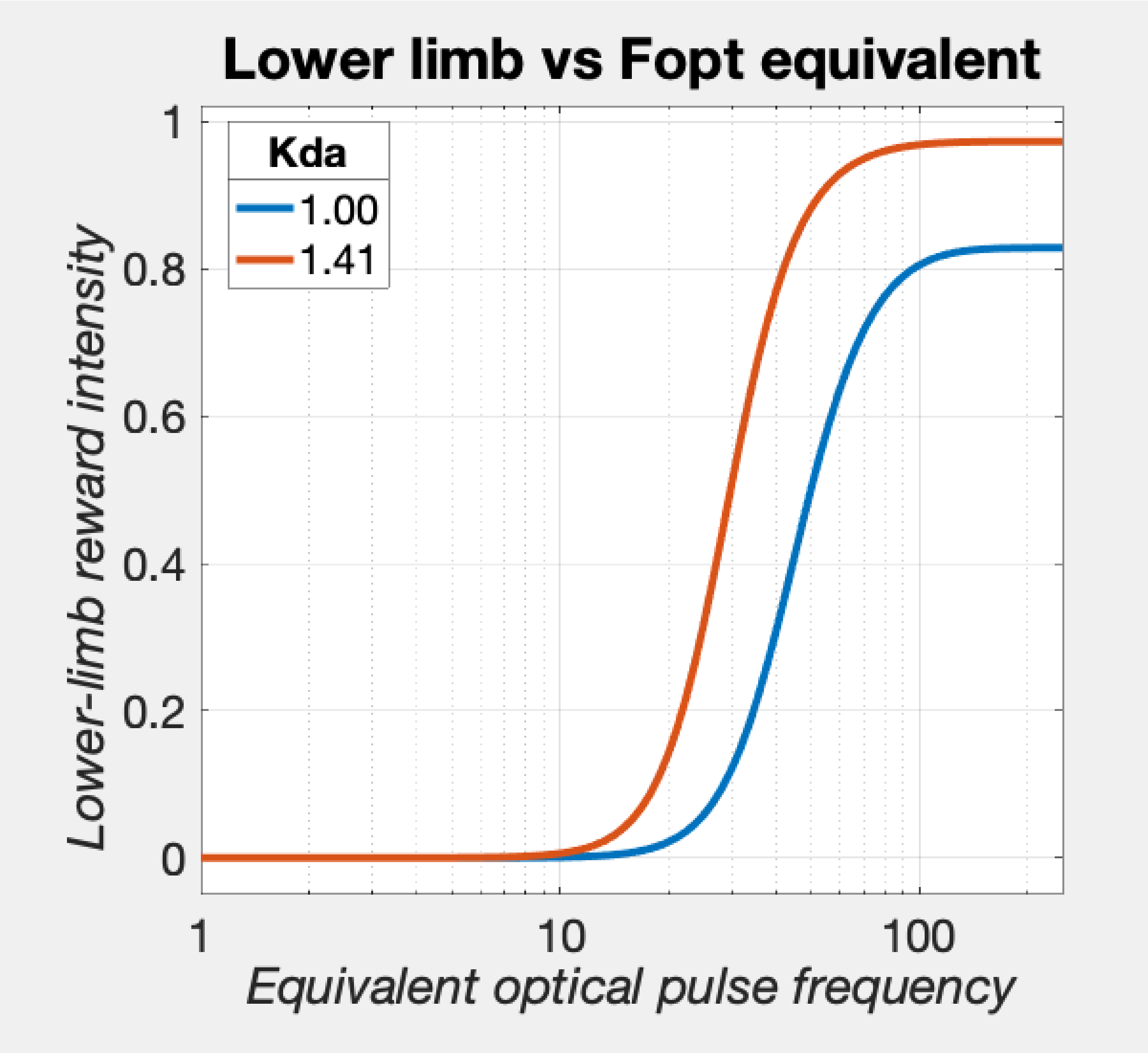


```
[fig_num, fig_tab] = add_fig(fig_num, fig_tab, "RG_MFB_DA_FoptEquiv_Kda", ...
    "Reward growth in lower limb as a function of equivalent optical pulse frequency");
```

```
This is figure #54: (RG_MFB_DA_FoptEquiv_Kda)
```

The previous graph shows lower-limb reward intensity as a function of the **equivalent optical pulse frequency** in the presence and absence of dopamine-transporter blockade. The next graph will plot lower-limb reward intensity as a function of the **electrical MFB pulse frequency**, again in the presence and absence of dopamine transporter blockade.

```
KdaMat = repmat(Kda',1,numF); % make matrix the same size as FelecMat
FdaHMmat = repmat(FdaHMkDA',1,numF); % make matrix the same size as FelecMat
Rlower = fRbsr(FmfbDAdrive, FdaBend, FdaHMmat, FdaRO, gDA, KrgLower);

TitleStrSemi = 'Lower limb vs Felec';
pnam = "Kda";
fnam = "Kda";
% The data to be plotted must be in columns. Thus, FelecMat and RelecMat are transposed
RG_MFB_DA_Felec_Kda = plot_RG(FelecMat',Rlower',pnam,Kda,fnam,TitleStrSemi,'lin', 'Fele
RG_MFB_DA_Felec_Kda = modify_2D_graph(RG_MFB_DA_Felec_Kda, 'Axes', 'XLim', [5,500], ...
```

```
    'RG_Kda_Felec_semilog', graphs2files, FigDir);
axh = findall(RG_MFB_DA_Felec_Kda, 'Type','Axes');
axh.XLabel.String = "Electrical pulse frequency";
axh.YLabel.String = "Lower-limb reward intensity";
if show_graphics
    RG_MFB_DA_Felec_Kda.Visible = 'on';
end
```

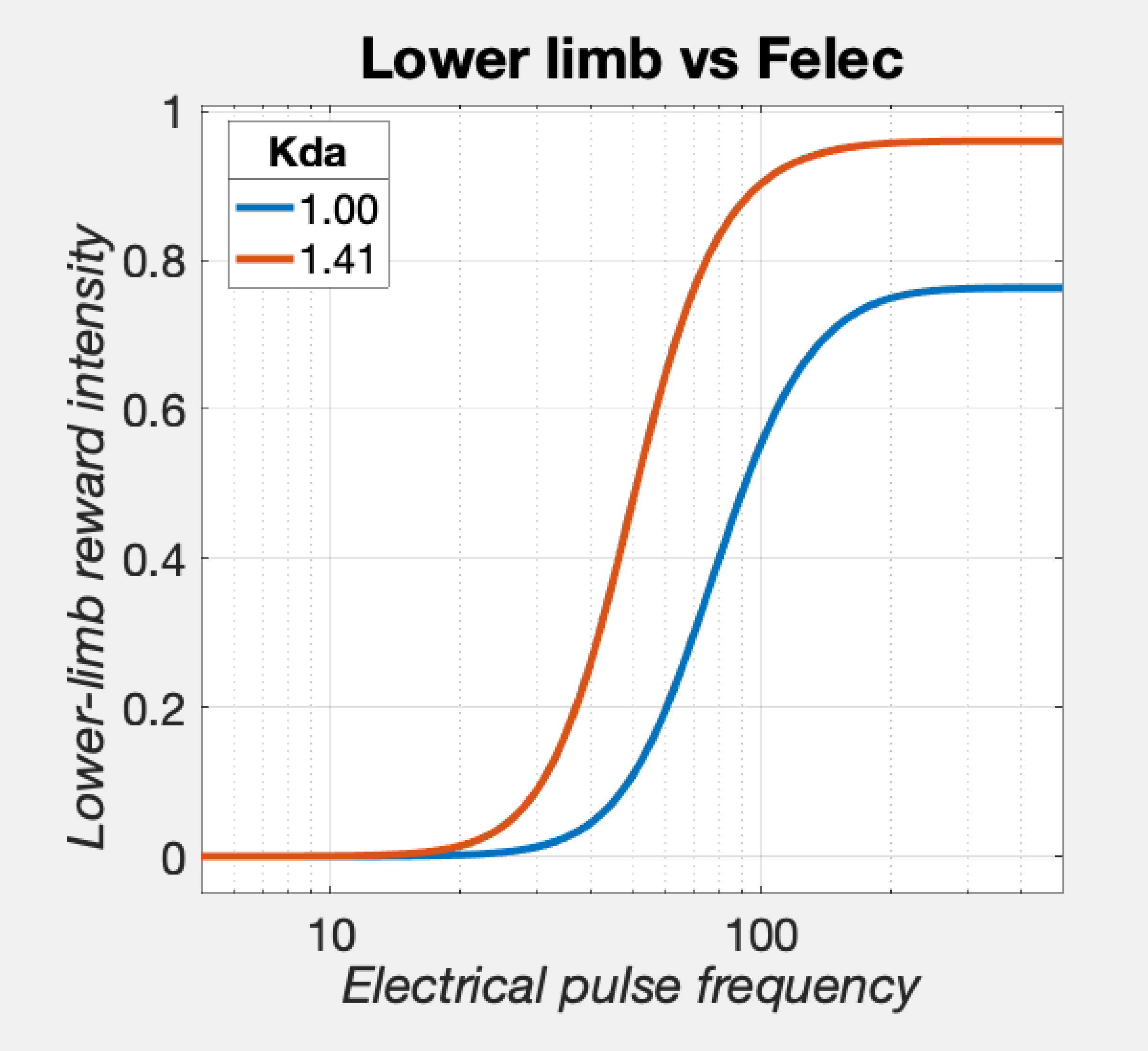


```
[fig_num, fig_tab] = add_fig(fig_num, fig_tab, "RG_MFB_DA_Felec_Kda", ...
    "Reward growth in lower limb as a function of electrical pulse frequency");
```

```
This is figure #55: (RG_MFB_DA_Felec_Kda)
```

In the convergence model, the reward-intensity signal in the upper limb is not changed by dopamine-transporter blockade. Thus, we can simply add the reward-intensity signal already computed to the results for the lower-limb reward-intensity signal to obtain the summated output of the two limbs. This corresponds to the 'benefit' signal at the right of

```
disp(string({strcat({'Figure '},num2str(fig_tab.Number(fig_tab.Name=="Convergence_model
```

```
Figure 48
```

```
Rupper = repmat(Rupper,numParamVals,1); % add rows to Rupper so that it is the same siz
Rsum = Rupper + Rlower;
```

The following graph shows the summated reward-intensity signal in response to MFB drive equivalent to an optical pulse frequency of

```
FoptEquiv
```

```
FoptEquiv = 80
```

pulses s^{-1}

```
[FsumHM, RsumMax] = find_FhmSum(FelecMat, Rsum)
```

```
FsumHM = 2×1
   75.2675
   63.0957
RsumMax = 2×1
    1.7626
    1.9598
```

```
% plot Rupper, Rlower, and Rxor for the vehicle and drug conditions
% Three curves will be plotted per graph; the matrices must be dimensioned accordingly
FelecMatPlot = repmat(Felec, 3, 1);
RmatVeh = [Rupper(1,:);Rlower(1,:);Rsum(1,:)];
RmatDrg = [Rupper(2,:);Rlower(2,:);Rsum(2,:)];

pnam = "1:up 2:lo 3:sum";
pVec = [1,2,3];
fnam = strcat("Sum",num2str(FoptEquiv),"Veh");
TitleStr = strcat("FoptEquiv=",num2str(FoptEquiv),"; vehicle");
RGsum_Rveh = plot_RG(FelecMatPlot',RmatVeh',pnam,pVec,fnam,TitleStr,'lin');
axh = findall(RGsum_Rveh, 'Type', 'Axes');
axh.XLabel.String = 'Electrical pulse frequency';
lh = findall(RGsum_Rveh, 'Type', 'Line');
lcolor = {'m','g','c'};
for j=1:3
    lh(j).Color = lcolor{j};
end
if show_graphics
    RGsum_Rveh.Visible = 'on';
end
```

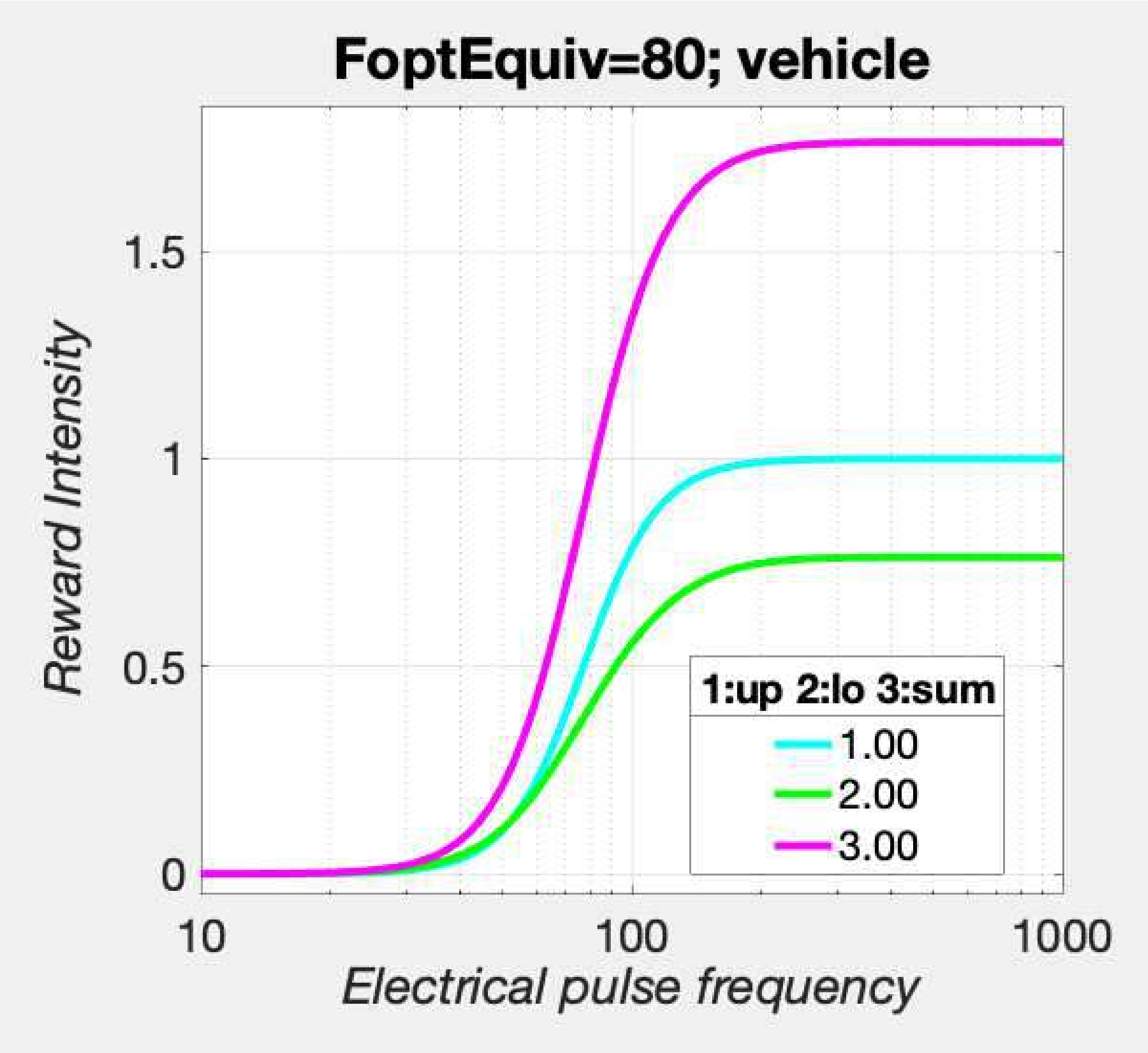


```
fig_nam = strcat("RGsum_Veh_FoptEquiv",num2str(FoptEquiv));
[fig_num, fig_tab] = add_fig(fig_num, fig_tab, fig_nam, "Reward summation in the conve
```

```
This is figure #56: (RGsum_Veh_FoptEquiv80)
```

```
fnam = strcat("sum",num2str(FoptEquiv),"Drg");
TitleStr = strcat("FoptEquiv=",num2str(FoptEquiv),"; drug");
RGsum_Rdrg = plot_RG(FelecMatPlot',RmatDrg',pnam,pVec,fnam,TitleStr,'lin');
axh = findall(RGsum_Rdrg, 'Type', 'Axes');
axh.XLabel.String = 'Electrical pulse frequency';
lh = findall(RGsum_Rdrg, 'Type', 'Line');
lcolor = {'m','g','c'};
for j=1:3
    lh(j).Color = lcolor{j};
    lh(j).LineStyle = '--';
end
if show_graphics
    RG_RDrg.Visible = 'on';
end
fig_nam = strcat("RGsum_Drg_FoptEquiv",num2str(FoptEquiv));
```

```
[fig_num, fig_tab] = add_fig(fig_num, fig_tab, fig_nam, "Reward summation in the conver
```

```
This is figure #57: (RGsum_Drg_FoptEquiv80)
```

```
shg
```

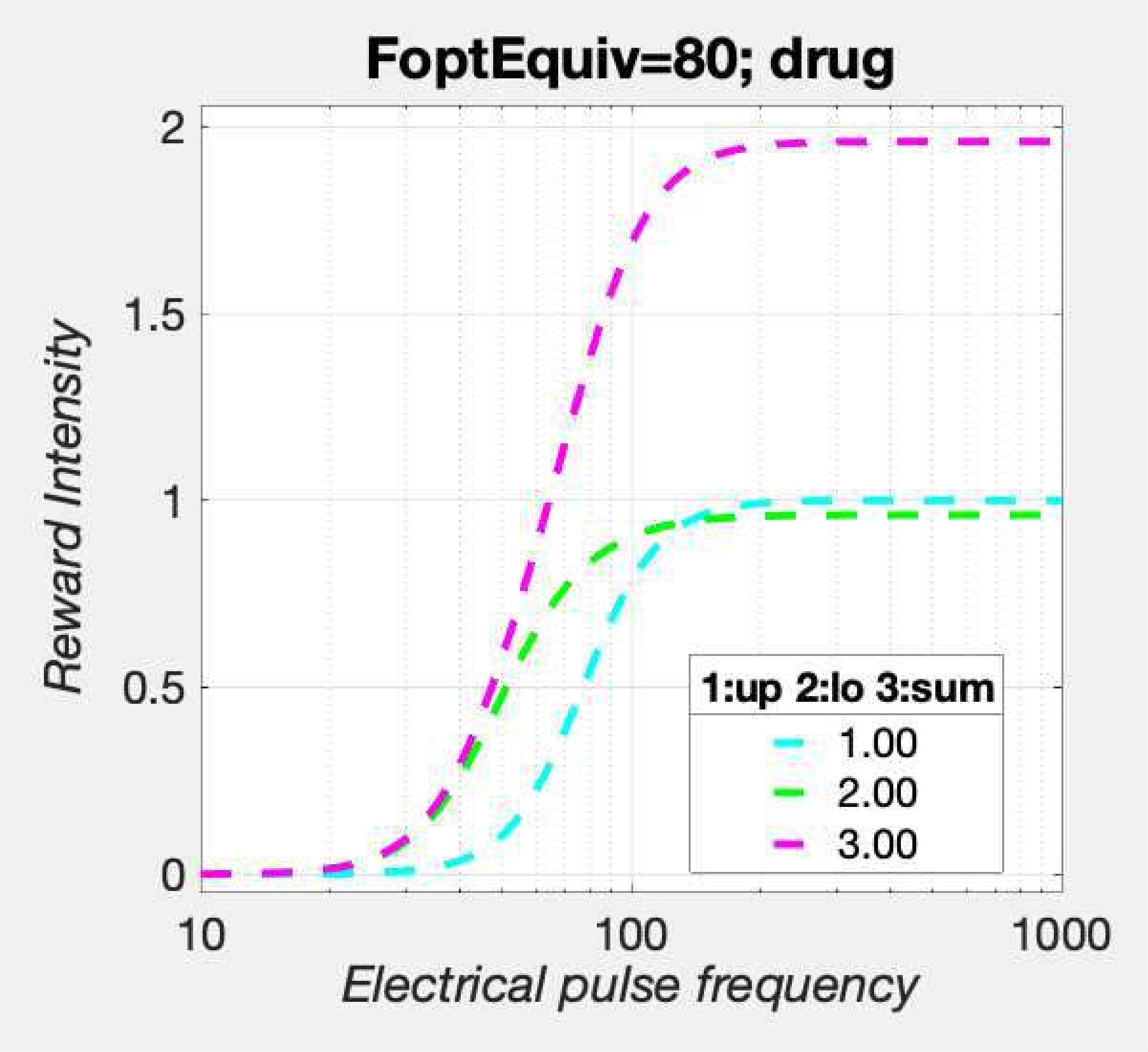


```
pnam = "Kda";
pVec = [Kda(1),Kda(2)];
fnam = strcat("Sum",num2str(FoptEquiv),"VehDrg");
TitleStr = strcat("FoptEquiv=",num2str(FoptEquiv),"; sum");
RGsum_Rvehdrg = plot_RG(FelecMat',Rsum',pnam,pVec,fnam,TitleStr,'lin');
axh = findall(RGsum_Rvehdrg, 'Type', 'Axes');
axh.XLabel.String = 'Electrical pulse frequency';
lh = findall(RGsum_Rvehdrg, 'Type', 'Line');
for j=1:2
    lh(j).Color = 'm';
end
lh(1).LineStyle = '--';
lgndh = findall(RGsum_Rvehdrg,'Type','Legend');
lgndh.Title.String = 'condition';
lgndh.String = {'vehicle','drug'};
```

```
if show_graphics
    RGsum_Rvehdrg.Visible = 'on';
end
```

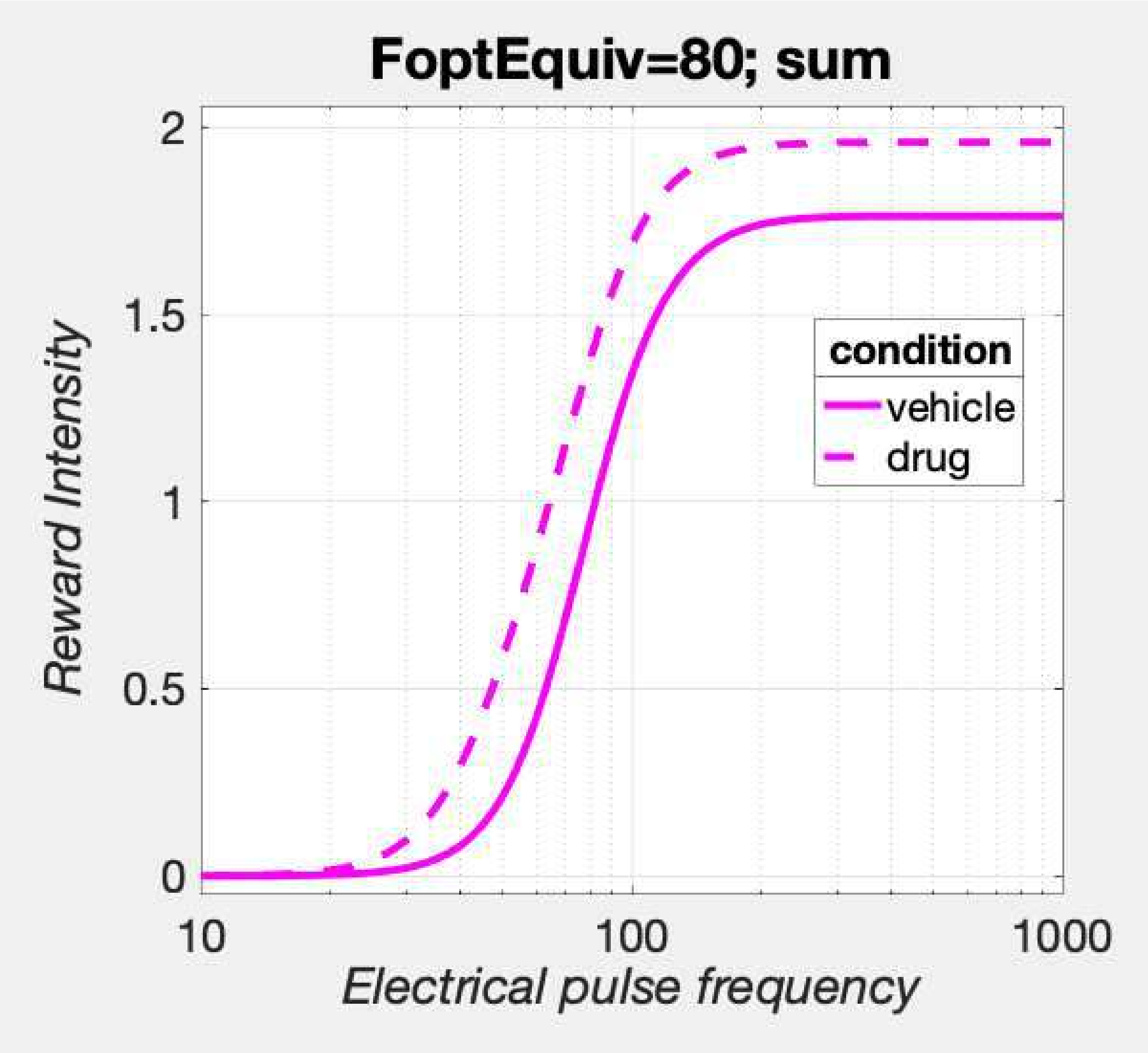


```
fig_nam = strcat("RGsum_VehDrg_FoptEquiv",num2str(FoptEquiv));
[fig_num, fig_tab] = add_fig(fig_num, fig_tab, fig_nam, "Drug modulation of reward summ
```

```
This is figure #58: (RGsum_VehDrg_FoptEquiv80)
```

At this high value of MFB drive, a small leftward curve shift is produced.

With the reward-intensity signal in hand, we can proceed to generate the mountains predicted in the presence and absence of dopamine-transporter blockade.

```
close all;

FPmax = 1000;
FhmUpperVec = repmat(FhmUpper,1,2); % Upper limb output is independent of Kda
RupperNormMax = fRbsrNorm(FPmax, FelecBend, FhmUpperVec, FelecRO, gElec)';
RlowerNormMax = fRbsrNorm(FoptEquiv, FdaBend, FdaHMkDA, FdaRO, gDA)';
```

```
RupperMax = max(Rupper, [], 2);
RlowerMax = max(Rlower, [], 2);
RupperRatio =  RupperMax ./ (RlowerMax + RupperMax)
```

```
RupperRatio = 2×1
    0.5671
    0.5100
```

```
RlowerRatio =  RlowerMax ./ (RlowerMax + RupperMax)
```

```
RlowerRatio = 2×1
    0.4329
    0.4900
```

```
RsumNormMax = (RupperRatio .* RupperNormMax) + (RlowerRatio .* RlowerNormMax); % Weight

dotPhiObj = 1;
Kaa = 1;
Kec = 1;
Krg = 1;
dotRaa = 0.1;
pObj = 1;
PsubBend = 0.5;
PsubMin = 1.82;
PobjE = PobjEfun(dotPhiObj, Kaa, Kec, Krg, dotRaa, pObj, PsubBend, PsubMin, RsumNormMax
```

```
PobjE = 2×1
    8.9716
    9.8029
```

```
a = 3;
numP = numF;
Pobj = logspace(0,3,numP); % row variable
TsumKda1 = TAsumFun(a, Pobj, PobjE(1), PsubBend, PsubMin, Rsum(1,:)', RsumNormMax(1));
TsumKda2 = TAsumFun(a, Pobj, PobjE(2), PsubBend, PsubMin, Rsum(2,:)', RsumNormMax(2));
% n.b., numel(Rsum(x)) = numel(Pobj). Rsum(x) has been transposed. Thus, TsumKda(x) is

% Plot the individual mountains for the two values of Kda
title_str1 = strcat({'Kda = '}, sprintf('%2.2f', Kda(1)));
MTNsumKda1 = plot_MTN(Felec, Pobj, TsumKda1, 'off', 'MTNsumKda1', title_str1, ...
    graphs2files, FigDir);
title_str2 = strcat({'Kda = '}, sprintf('%2.2f', Kda(2)));
MTNsumKda2 = plot_MTN(Felec, Pobj, TsumKda2, 'off', 'MTNsumKda1', title_str2, ...
    graphs2files, FigDir);

% Plot the pair of mountains for the two values of Kda
dual_sum_plot = dual_subplot(MTNsumKda1, MTNsumKda2, 'MTNsum_Kda1_Kda2',...
    graphs2files,FigDir);
if show_graphics
    dual_sum_plot.Visible = 'on';
end
```

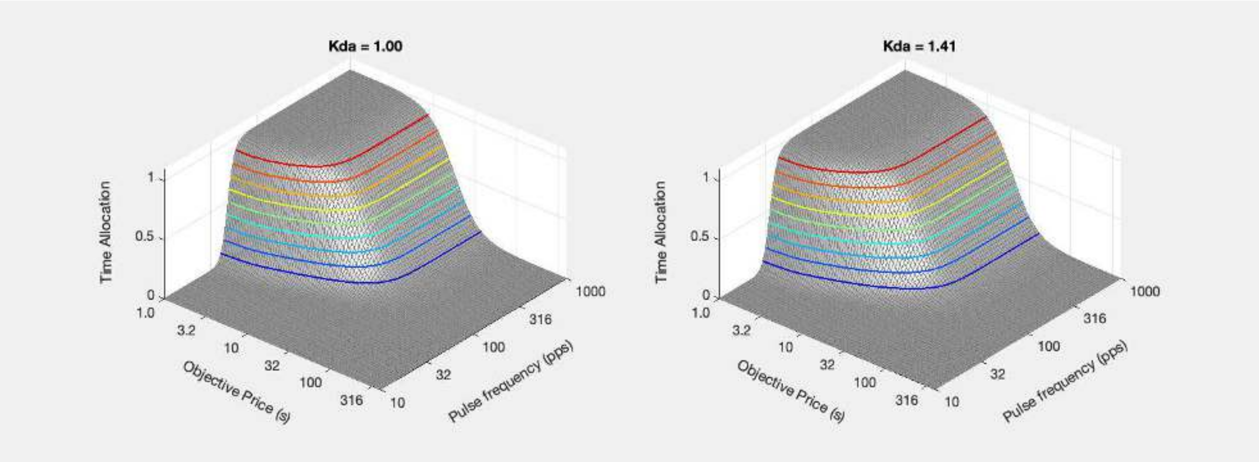


```
% Estimate the weighted average of the firing frequencies that produce half-maximal rew
% upper and lower limbs
FhmUpperStar = FilterFun(FhmUpper,FelecBend,FelecRO);

FhmLowerDrive = FmfbDAdriveFun(FmfbDAbend, FsumHM, FFmfbDAmax, FoptEquiv, FmfbDAro);
FFhmLower = FilterFun(FhmLowerDrive,FdaBend, FdaRO);

FsumHMstar = FsumHM .* ...
    ((RupperRatio .* (FhmUpperStar ./ FsumHM)) + (RlowerRatio .* (FFhmLower ./ FhmLower
% Weighted average of the frequency-following fidelity in the upper and lower limbs
PsubE = PsubFun(PobjE,PsubBend,PsubMin);
PsubEstar = PsubE ./ RsumNormMax;
PobjEstar = PsubBsFun(PsubEstar,PsubBend,PsubMin);

ContkDA1sum = plot_contour(Felec, Pobj, TsumKda1, PobjE(1), FsumHM(1), 'off', 'ContkDA1
    strcat({'Kda = '}, num2str(Kda(1))), graphs2files, FigDir);
ContkDA2sum = plot_contour(Felec, Pobj, TsumKda2, PobjE(2), FsumHM(2), 'off', 'ContkDA2
    strcat({'Kda = '}, num2str(Kda(2))), graphs2files, FigDir);

bg_kDA1vskDA2sum = plot_bgStar(FsumHM(1), FsumHM(2), FsumHMstar(1), FsumHMstar(2),...
    PobjE(1), PobjE(2), PobjEstar(1), PobjEstar(2),...
    'off', 'kDA1vskDA2sum_bg', graphs2files, FigDir, -0.3, 0.3);
% Set a scale for the y-axis of this bar graph that ressembles the scales for the other
bg_root = 'bg_kDA1vskDA2sum';

quad_kDA1vskDA2sum = quad_subplot(ContkDA1sum, ContkDA2sum, bg_kDA1vskDA2sum, 'quad_kDA
    graphs2files, FigDir);
if show_graphics
    quad_kDA1vskDA2sum.Visible = 'on';
end
```

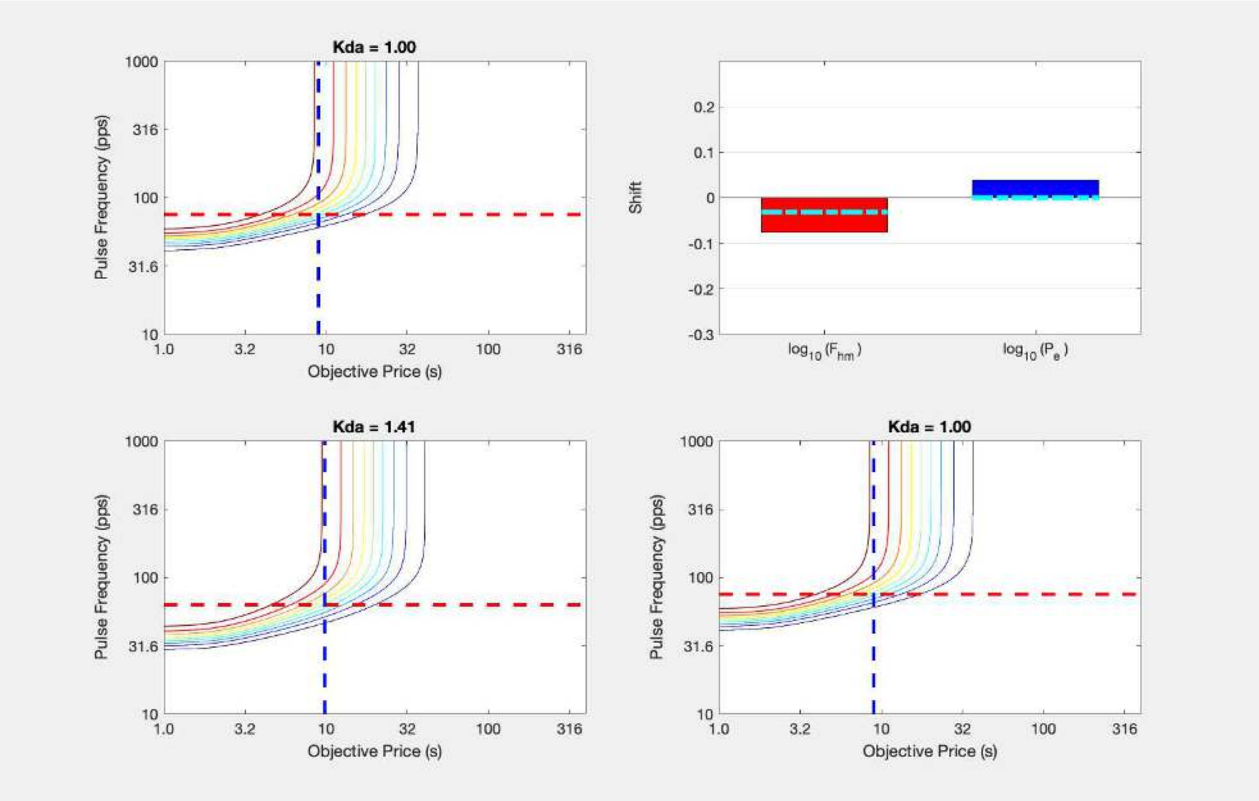


```
fig_nam = strcat("quad_kDA1vskDA2sum",num2str(FoptEquiv));
[fig_num, fig_tab] = add_fig(fig_num, fig_tab, ...
    fig_nam, "Modest downward shift of the reward mountain induced by dopamine-transpor
```

```
This is figure #59: (quad_kDA1vskDA2sum80)
```

```
% Insert section break to force display of the last graph
```

```
close all;
```

As expected from the small leftward displacement of the reward-intensity growth function by dopamine transporter blockade shown in

```
disp(string({strcat({'Figure '},num2str(fig_tab.Number(fig_tab.Name=="RGsum_VehDrg_Fopt
```

```
Figure 58
```

the mountain is shifted modestly along the pulse-frequency axis. None of the eight subjects in the Hernandez et al. (2012) eICSS study showed such a shift.

Adding dopaminergic modulation of effort cost (i.e., a drug-induced reduction in these costs) boosts the shift along the price axis. The same effect could be produced by boosting the output of the reward-growth function(s) $(\uparrow K_{rg})$ or decreasing the value of alternate activities.

With dopaminergic modulation of effort cost:

```
KeffMod = [1;10^0.15]; % Median shift in Pe in the current oICSS study: 10^0.1525
PobjE = PobjEfun(dotPhiObj, Kaa, Kec, Krg, dotRaa, pObj, PsubBend, PsubMin, RsumNormMax
```

```
PobjE = 2×1
    8.9716
   13.8470
```

```
TsumEffModKda1 = TAsumFun(a, Pobj, PobjE(1), PsubBend, PsubMin, Rsum(1,:)', RsumNormMax
TsumEffModKda2 = TAsumFun(a, Pobj, PobjE(2), PsubBend, PsubMin, Rsum(2,:)', RsumNormMax
% n.b., numel(Rsum(x)) = numel(Pobj). Rsum(x) has been transposed. Thus, TsumKda(x) is

% Plot the individual mountains for the two values of Kda
title_str1 = strcat({'Kda = '}, sprintf('%2.2f', Kda(1)));
MTNsumEffModKda1 = plot_MTN(Felec, Pobj, TsumEffModKda1, 'off', 'MTNsumEffModKda1', tit
    graphs2files, FigDir);
title_str2 = strcat({'Kda = '}, sprintf('%2.2f', Kda(2)));
MTNsumEffModKda2 = plot_MTN(Felec, Pobj, TsumEffModKda2, 'off', 'MTNsumEffModKda2', tit
    graphs2files, FigDir);

% Plot the pair of mountains for the two values of Kda
dual_sum_plot = dual_subplot(MTNsumEffModKda1, MTNsumEffModKda2, 'MTNsumEffMod_Kda1_Kda
    graphs2files,FigDir);
if show_graphics
    dual_sum_plot.Visible = 'on';
end
```

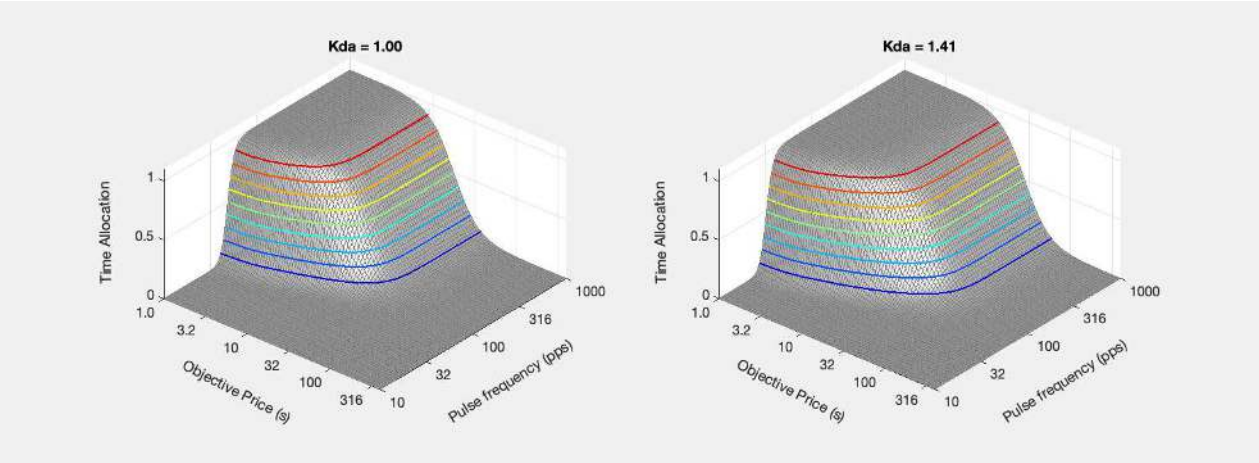


```
% Use value of FsumHMstar calculated above;
PsubE = PsubFun(PobjE,PsubBend,PsubMin);
PsubEstar = PsubE ./ RsumNormMax;
PobjEstar = PsubBsFun(PsubEstar,PsubBend,PsubMin);

ContkDA1sumEffMod = plot_contour(Felec, Pobj, TsumEffModKda1, PobjE(1), FsumHM(1), 'off
    'ContkDA1sumEffMod', title_str1, ...
    strcat({'Kda = '}, num2str(Kda(1))), graphs2files, FigDir);
ContkDA2sumEffMod = plot_contour(Felec, Pobj, TsumEffModKda2, PobjE(2), FsumHM(2), 'off
    'ContkDA2sumEffMod', title_str2, ...
    strcat({'Kda = '}, num2str(Kda(2))), graphs2files, FigDir);

bg_kDA1vskDA2sumEffMod = plot_bgStar(FsumHM(1), FsumHM(2), FsumHMstar(1), FsumHMstar(2)
    PobjE(1), PobjE(2), PobjEstar(1), PobjEstar(2),...
```

```
    'off', 'kDA1vskDA2sumEffMod_bg', graphs2files, FigDir, -0.3, 0.3);
% Set a scale for the y-axis of this bar graph that ressembles the scales for the other
bg_root = 'bg_kDA1vskDA2sumEffMod';

quad_kDA1vskDA2sumEffMod = quad_subplot(ContkDA1sumEffMod, ContkDA2sumEffMod, bg_kDA1vs
    'quad_kDA1vskDA2sumEffMod', bg_root, graphs2files, FigDir);
if show_graphics
    quad_kDA1vskDA2sumEffMod.Visible = 'on';
end
```

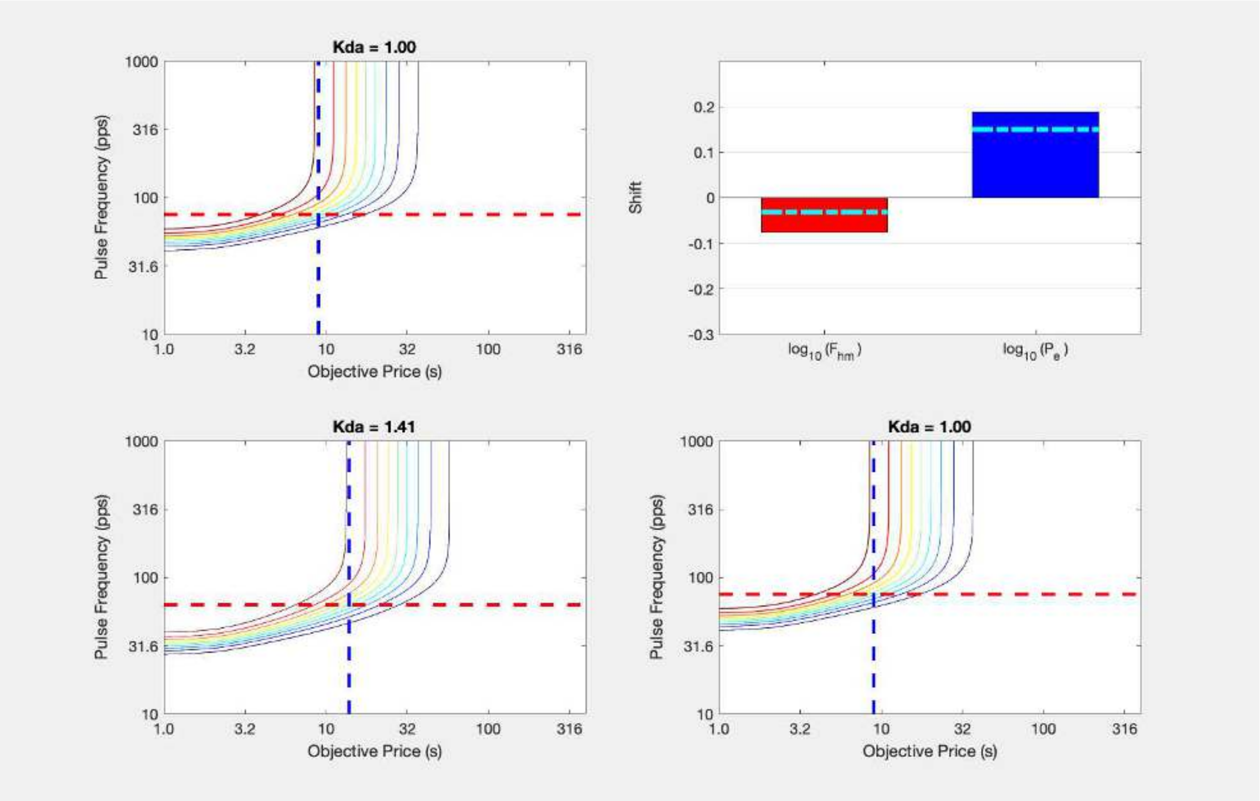


```
fig_nam = strcat("quad_kDA1vskDA2sumEffMod",num2str(FoptEquiv));
[fig_num, fig_tab] = add_fig(fig_num, fig_tab, ...
    fig_nam, "Dual shifts of the reward mountain induced by dopamine-transporter blocka
```

```
This is figure #60: (quad_kDA1vskDA2sumEffMod80)
```

```
close all;
vars2save = who;
ws_name = fullfile(pwd, 'WS_convergence_vars80.mat');
save_ws;
```

```
Saving workspace to /Users/shizgal/Work/Research/papers/In_Progress/Opto_GBR2/Simulations/WS_convergence_v
Copying backup to /Users/shizgal/Work/Research/papers/In_Progress/Opto_GBR2/Simulations/WS_convergence_var
```

The small shift along the pulse-frequency axis can be eliminated by reducing the MFB drive. We will illustrate this by means of reward-growth graphs.

```
% Rupper is unchanged - no need to regenerate it
% Regenerate Rlower using weaker MFB drive
FoptEquiv = 40; % Value was 80 in above simulations
FmfbDAdrive = FmfbDAdriveFun(FmfbDAbend, Felec, FFmfbDAmax, FoptEquiv, FmfbDAro);
FdaHMkDA = FpulseHMfun(C, D, FdaBend, FPmax, FdaRO, NnDA, RhoPiDA, Kda) % result is a t
```

```
FdaHMkDA = 1×2
   37.6342   25.1515
```

```
KdaMat = repmat(Kda',1,numF);
FdaHMmat = repmat(FdaHMkDA',1,numF);
Rlower = fRbsr(FmfbDAdrive, FdaBend, FdaHMmat, FdaRO, gDA, KrgLower);
Rsum = Rupper + Rlower;
[FsumHM, RsumMax] = find_FhmSum(FelecMat, Rsum)
```

```
FsumHM = 2×1
   84.1920
   84.2963
RsumMax = 2×1
    1.3105
    1.7715
```

```


RmatVeh = [Rupper(1,:);Rlower(1,:);Rsum(1,:)];
RmatDrg = [Rupper(2,:);Rlower(2,:);Rsum(2,:)];

pnam = "1:up 2:lo 3:sum";
pVec = [1,2,3];
fnam = strcat("Sum",num2str(FoptEquiv),"Veh");
TitleStr = strcat("FoptEquiv=",num2str(FoptEquiv),"; vehicle");
RGsum_Rveh = plot_RG(FelecMatPlot',RmatVeh',pnam,pVec,fnam,TitleStr,'lin');
axh = findall(RGsum_Rveh, 'Type', 'Axes');
axh.XLabel.String = 'Electrical pulse frequency';
lh = findall(RGsum_Rveh, 'Type', 'Line');
lcolor = {'m','g','c'};
for j=1:3
    lh(j).Color = lcolor{j};
end
if show_graphics
    RGsum_Rveh.Visible = 'on';
end
```

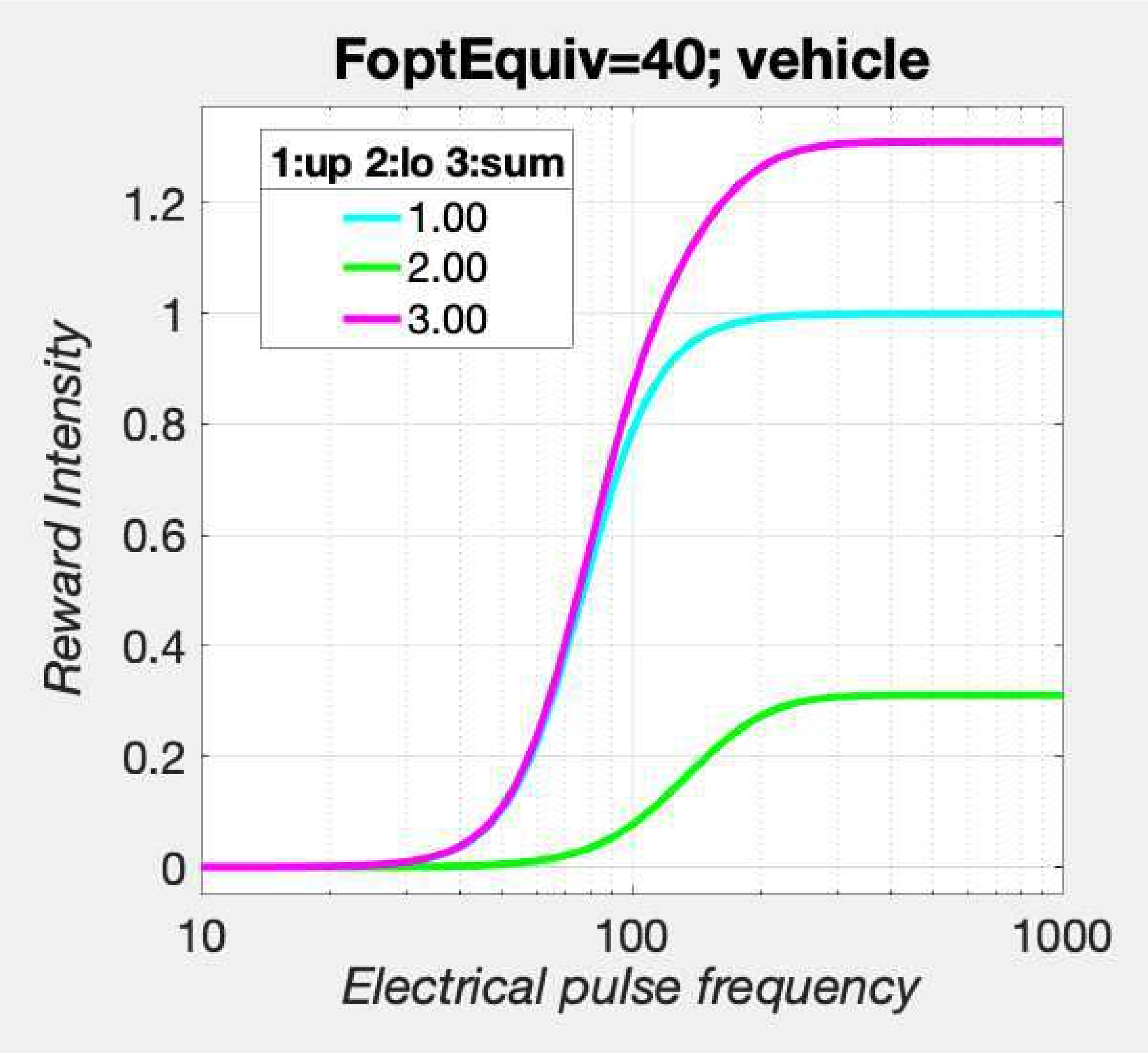


```
fig_nam = strcat("RGsum_Veh_FoptEquiv",num2str(FoptEquiv));
[fig_num, fig_tab] = add_fig(fig_num, fig_tab, fig_nam, "Reward summation in the conver
```

```
This is figure #61: (RGsum_Veh_FoptEquiv40)
```

```
pnam = "1:up 2:lo 3:sum";
pVec = [1,2,3];
fnam = strcat("Sum",num2str(FoptEquiv),"Drg");
TitleStr = strcat("FoptEquiv=",num2str(FoptEquiv),"; drug");
RGsum_Rdrg = plot_RG(FelecMatPlot',RmatDrg',pnam,pVec,fnam,TitleStr,'lin');
axh = findall(RGsum_Rdrg, 'Type', 'Axes');
axh.XLabel.String = 'Electrical pulse frequency';
lh = findall(RGsum_Rdrg, 'Type', 'Line');
lcolor = {'m','g','c'};
for j=1:3
    lh(j).Color = lcolor{j};
    lh(j).LineStyle = '--';
end
if show_graphics
    RGsum_Rdrg.Visible = 'on';
```

```
end
```

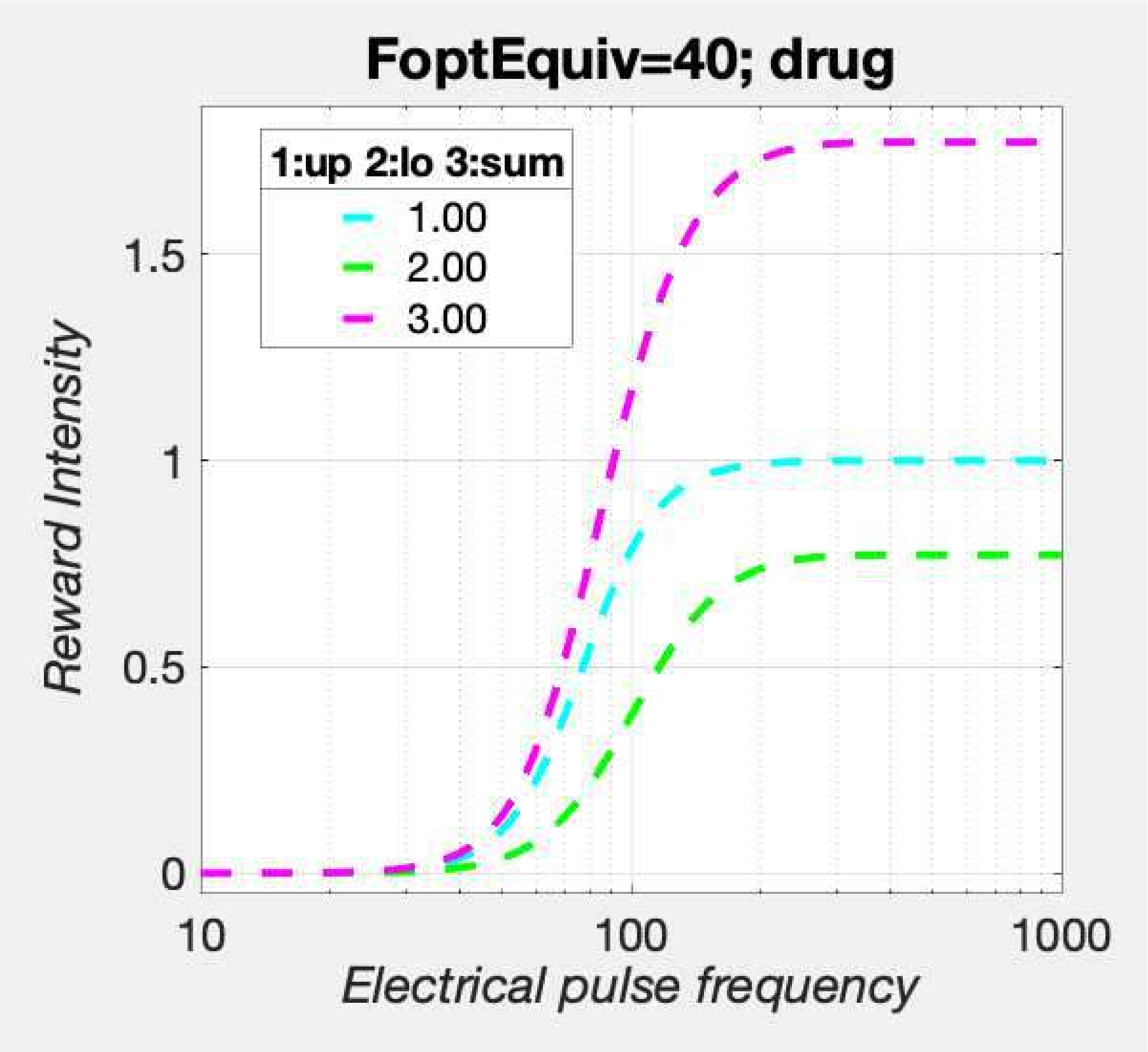


```
fig_nam = strcat("RGsum_Drg_FoptEquiv",num2str(FoptEquiv));
[fig_num, fig_tab] = add_fig(fig_num, fig_tab, fig_nam, "Reward summation in the conver
```

```
This is figure #62: (RGsum_Drg_FoptEquiv40)
```

```
pnam = "Kda";
pVec = [Kda(1),Kda(2)];
fnam = strcat("Sum",num2str(FoptEquiv),"VehDrg");
TitleStr = strcat("FoptEquiv=",num2str(FoptEquiv),"; sum");
RGsum_Rvehdrg = plot_RG(FelecMat',Rsum',pnam,pVec,fnam,TitleStr,'lin');
axh = findall(RGsum_Rvehdrg, 'Type', 'Axes');
axh.XLabel.String = 'Electrical pulse frequency';
lh = findall(RGsum_Rvehdrg, 'Type', 'Line');
lh(1).LineStyle = '--';
for j=1:2
    lh(j).Color = 'm';
end
lgndh = findall(RGsum_Rvehdrg, 'Type', 'Legend');
```

```
lgndh.Title.String = "condition";
lgndh.String = {'vehicle','drug'};
if show_graphics
    RGsum_Rvehdrg.Visible = 'on';
end
```

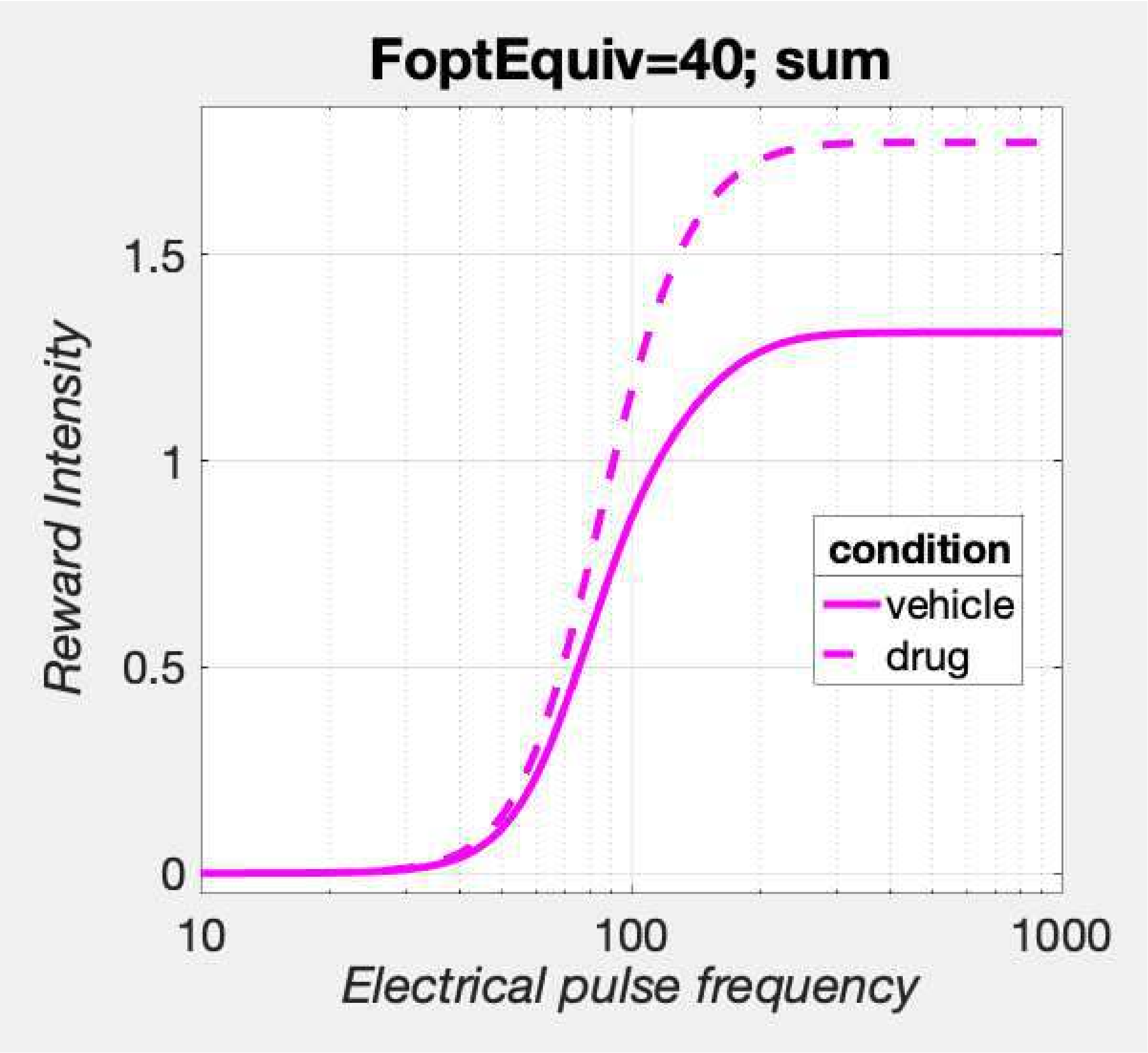


```
fig_nam = strcat("RGsum_VehDrg_FoptEquiv",num2str(FoptEquiv));
[fig_num, fig_tab] = add_fig(fig_num, fig_tab, fig_nam, "Drug modulation of reward summ
```

```
This is figure #63: (RGsum_VehDrg_FoptEquiv40)
```

At this weaker value of MFB drive, the reward-growth curve is shifted to the right. At this value of K_{da} (~median for the current study), the drug cannot shift the reward-growth curve far enough to the left to displace the summated curve leftwards. Thus, we learn from this that shifts along the pulse-frequency axis in the convergence model depend on the strength of the MFB drive.

```
close all;

FPmax = 1000;
```

```
FhmUpperVec = repmat(FhmUpper,1,2); % Upper limb output is independent of Kda
RupperNormMax = fRbsrNorm(FPmax, FelecBend, FhmUpperVec, FelecRO, gElec)';
RlowerNormMax = fRbsrNorm(FoptEquiv, FdaBend, FdaHMkDA, FdaRO, gDA)';
RupperMax = max(Rupper, [], 2);
RlowerMax = max(Rlower, [], 2);
RupperRatio =  RupperMax ./ (RlowerMax + RupperMax)
```

```
RupperRatio = 2×1
    0.7627
    0.5643
```

```
RlowerRatio =  RlowerMax ./ (RlowerMax + RupperMax)
```

```
RlowerRatio = 2×1
    0.2373
    0.4357
```

```
RsumNormMax = (RupperRatio .* RupperNormMax) + (RlowerRatio .* RlowerNormMax); % Weight

dotPhiObj = 1;
Kaa = 1;
Kec = 1;
Krg = 1;
dotRaa = 0.1;
pObj = 1;
PsubBend = 0.5;
PsubMin = 1.82;
PobjE = PobjEfun(dotPhiObj, Kaa, Kec, Krg, dotRaa, pObj, PsubBend, PsubMin, RsumNormMax
```

```
PobjE = 2×1
    8.3618
   12.7179
```

```


a = 3;
numP = numF;
Pobj = logspace(0,3,numP); % row variable
TsumEffModLoFoeqKda1 = TAsumFun(a, Pobj, PobjE(1), PsubBend, PsubMin, Rsum(1,:)', RsumN
TsumEffModLoFoeqKda2 = TAsumFun(a, Pobj, PobjE(2), PsubBend, PsubMin, Rsum(2,:)', RsumN
% n.b., numel(Rsum(x)) = numel(Pobj). Rsum(x) has been transposed. Thus, TsumKda(x) is

% Plot the individual mountains for the two values of Kda
title_str1 = strcat({'Kda = '}, sprintf('%2.2f', Kda(1)));
MTNsumEffModLoFoeqKda1 = plot_MTN(Felec, Pobj, TsumEffModLoFoeqKda1, 'off', 'MTNsumEffM
    graphs2files, FigDir);
title_str2 = strcat({'Kda = '}, sprintf('%2.2f', Kda(2)));
MTNsumEffModKLoFoeqda2 = plot_MTN(Felec, Pobj, TsumEffModLoFoeqKda2, 'off', 'MTNsumEffM
    graphs2files, FigDir);

% Plot the pair of mountains for the two values of Kda
dual_sum_plot = dual_subplot(MTNsumEffModLoFoeqKda1, MTNsumEffModKLoFoeqda2, 'MTNsumEff
    graphs2files,FigDir);
if show_graphics
    dual_sum_plot.Visible = 'on';
end
```

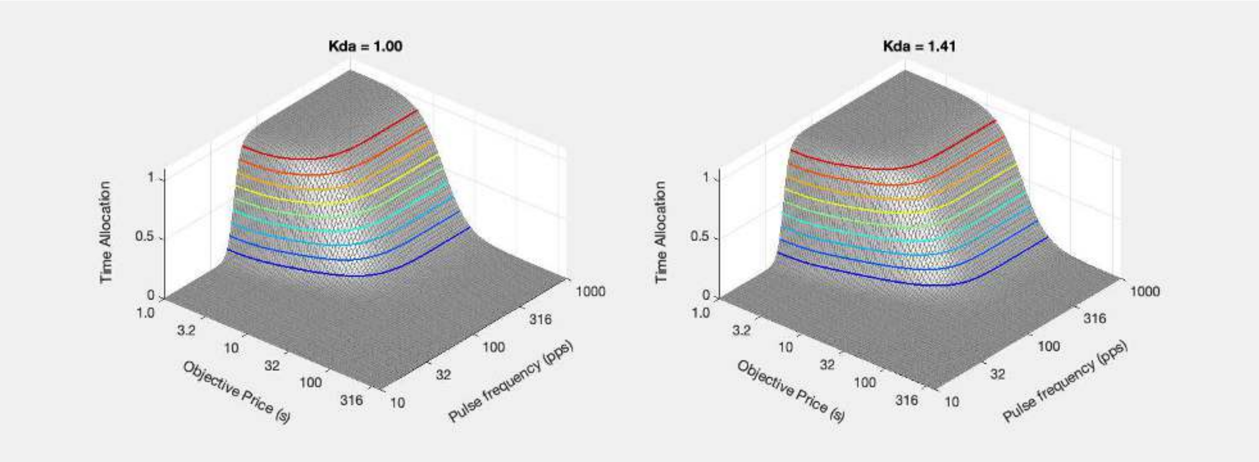


```
% Estimate the weighted average of the firing frequencies that produce half-maximal rew
% upper and lower limbs
FhmUpperStar = FilterFun(FhmUpper,FelecBend,FelecRO);

FhmLowerDrive = FmfbDAdriveFun(FmfbDAbend, FsumHM, FFmfbDAmax, FoptEquiv, FmfbDAro);
FFhmLowerStar = FilterFun(FhmLowerDrive,FdaBend, FdaRO);

FsumHMstar = FsumHM .* ...
    ((RupperRatio .* (FhmUpperStar ./ FsumHM)) + (RlowerRatio .* (FFhmLowerStar ./ FhmI

PsubE = PsubFun(PobjE,PsubBend,PsubMin);
PsubEstar = PsubE ./ RsumNormMax;
PobjEstar = PsubBsFun(PsubEstar,PsubBend,PsubMin);

ContkDA1sumEffMod = plot_contour(Felec, Pobj, TsumEffModLoFoeqKda1, PobjE(1), FsumHM(1)
    'ContkDA1sumEffModLoFoeq', title_str1, ...
    strcat({'Kda = '}, num2str(Kda(1))), graphs2files, FigDir);
ContkDA2sumEffMod = plot_contour(Felec, Pobj, TsumEffModLoFoeqKda2, PobjE(2), FsumHM(2)
    'ContkDA2sumEffModLoFoeq', title_str2, ...
    strcat({'Kda = '}, num2str(Kda(2))), graphs2files, FigDir);

bg_kDA1vskDA2sumEffMod = plot_bgStar(FsumHM(1), FsumHM(2), FsumHMstar(1), FsumHMstar(2)
    PobjE(1), PobjE(2), PobjEstar(1), PobjEstar(2),...
    'off', 'kDA1vskDA2sumEffMod_bg', graphs2files, FigDir, -0.3, 0.3);
% Set a scale for the y-axis of this bar graph that ressembles the scales for the other
bg_root = 'bg_kDA1vskDA2sumEffMod';

quad_kDA1vskDA2sumEffMod = quad_subplot(ContkDA1sumEffMod, ContkDA2sumEffMod, bg_kDA1vs
    'quad_kDA1vskDA2sumEffMod', bg_root, graphs2files, FigDir);
if show_graphics
    quad_kDA1vskDA2sumEffMod.Visible = 'on';
end
```

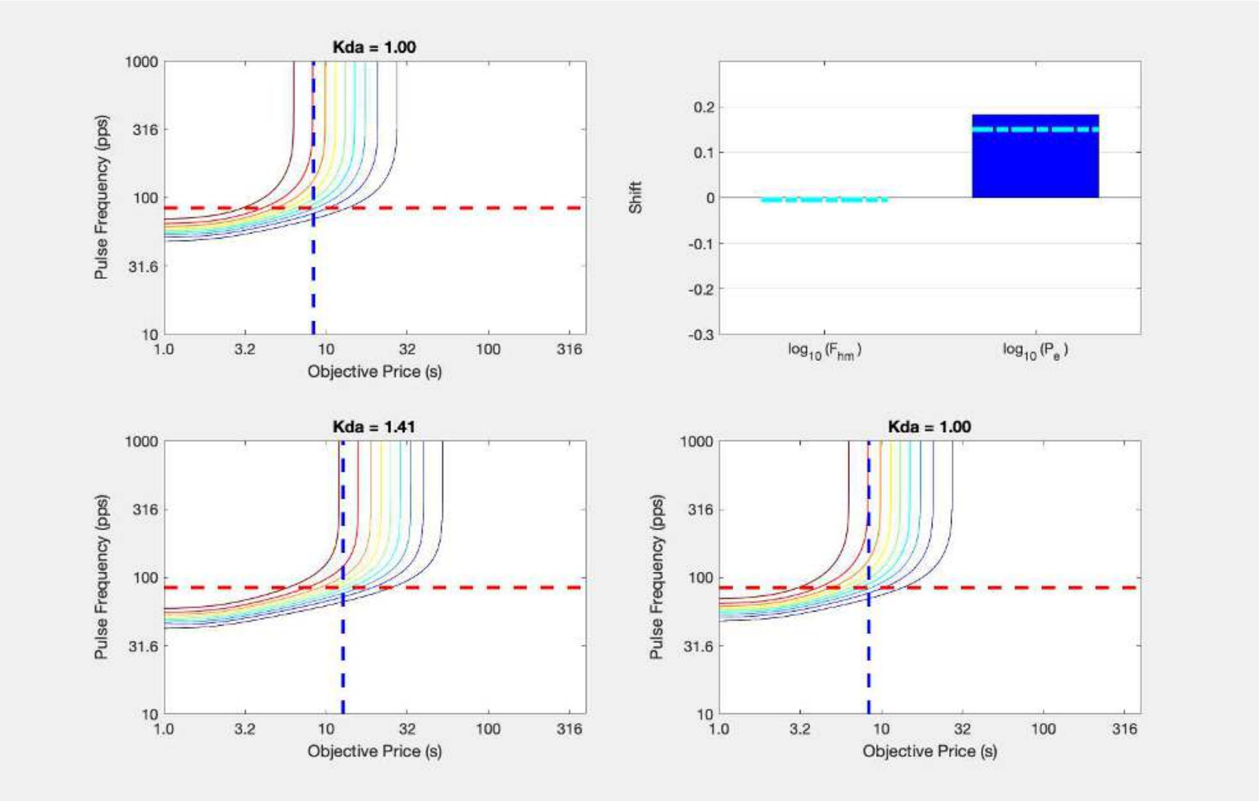


```
fig_nam = strcat("quad_kDA1vskDA2sumEffMod",num2str(FoptEquiv));
[fig_num, fig_tab] = add_fig(fig_num, fig_tab, ...
    fig_nam, "Dual shifts of the reward mountain induced by dopamine-transporter blocka
```

```
This is figure #64: (quad_kDA1vskDA2sumEffMod40)
```

```
close all;
vars2save = who;
ws_name = fullfile(pwd, 'WS_convergence_vars.mat');
save_ws;
```

```
Saving workspace to /Users/shizgal/Work/Research/papers/In_Progress/Opto_GBR2/Simulations/WS_convergence_v
Copying backup to /Users/shizgal/Work/Research/papers/In_Progress/Opto_GBR2/Simulations/WS_convergence_var
```

```
clearvars('-except',keepVars{:})
keepVars = who; % Restore cell array containing names of variables to be retained
toc
```

```
Elapsed time is 42.313713 seconds.
```

```
tic;
```

Remarks concerning the convergence model

The results in

```
disp(string({strcat({'Figure '},num2str(fig_tab.Number(fig_tab.Name=='quad_kDA1vskDA2su
```

```
Figure
```

above resemble those obtained in the eICSS study in which the reward mountain was measured under the influence of GBR-12901 (Hernandez et al., 2012). Thus, given moderate MFB drive and $F_{pulse_{hm}}$ values compatible with those obtained in the present study (adjusted for a train duration of 0.5 s), the convergence model can generate outputs that match our earlier eICSS results.

The dependence of the output of the convergence model on experimental parameters is of interest. Although failure to observe shifts along the pulse-frequency axis was the most common result of the experiments in which the eICSS reward-mountain was measured under the influence of drugs that alter dopaminergic neurotransmission (27 of 32 cases reported by Hernandez et al., 2010, Trujillo-Pisanty et al., 2011, Hernandez et al., 2012, and Trujillo-Pisanty et al., 2014), it is not the only result. Reward mountains obtained from three subjects in the cocaine study (Hernandez et al., 2010) showed fairly substantial, reliable, shifts along the pulse-frequency axis. (The shift was marginally reliable in a fourth subject when tested initially but disappeared upon re-test.) Although no subject in the GBR-12909 (Hernandez et al., 2012) or pimozide (Trujillo-Pisanty et al., 2014) studies showed such shifts, one subject in the AM-251 study (Trujillo-Pisanty et al., 2011) did. Could variation in the strength of MFB drive on the dopamine neurons and in the individual $F_{pulse_{hm}}$ values for the dopamine pathway explain these findings?

Please see the main body of the manuscript for more detailed discussion.

Sort tables and write to files

Formatted equations

```
if tabdir
    writetable(sortrows(eqn_tab,2),...
        fullfile(tabdir,strcat('eqn_tab_v',num2str(version),'.csv')),...
        'WriteVariableNames', true);
end
```

Figures

```
if tabdir
    writetable(sortrows(fig_tab,2),...
    fullfile(tabdir,strcat('fig_tab_v',num2str(version),'.csv')),...
    'WriteVariableNames', true);
end
```

Functions

```
if tabdir
    writetable(sortrows(fun_tab,2),...
    fullfile(tabdir,strcat('fun_tab_v',num2str(version),'.csv')),...
    'WriteVariableNames', true);
end
```

Symbols

```
if tabdir
    writetable(sortrows(sym_tab,2),...
    fullfile(tabdir,strcat('sym_tab_v',num2str(version),'.csv')),...
    'WriteVariableNames', true);
end
```

Save workspace

```
if saveWS
    ws_name = fullfile(pwd, strcat('WS_GBR_eICSS_oICSS_v',num2str(version),'.mat'));
    save_ws;
end
```

```
Saving workspace to /Users/shizgal/Work/Research/papers/In_Progress/Opto_GBR2/Simulations/WS_GBR_eICSS_oIC
Copying backup to /Users/shizgal/Work/Research/papers/In_Progress/Opto_GBR2/Simulations/WS_GBR_eICSS_oICSS
```

Last revised on:

```
datetime('now')
```

```
ans = datetime
   01-Dec-2019 18:52:43
```

```
toc % elapsed time for this section
```

```
Elapsed time is 1.230854 seconds.
```

```
toc(t_start) % elapsed time for the entire script
```

```
Elapsed time is 226.200086 seconds.
```

Tools for this live script

```
function init_all
    global eqn_num eqn_tab fig_num fig_tab fun_num fun_tab sym_num sym_tab
    eqn_num = 0; % initialize counter
    eqn_nam = ""; % initialize equation-name string array
    eqn_desc = ""; % initialize equation-description string array;
    eqn_tab = table(eqn_num, eqn_nam, eqn_desc,'VariableNames', {'Number'; 'Name'; 'Des
    eqn_tab(1,:)=[]; % clear the table
    disp("The equation table has been initialized.");

    fig_num = 0; % initialize counter
    fig_nam = ""; % initialize figure-name string array
    fig_desc = ""; % initialize figure-description string array;
    fig_tab = table(fig_num, fig_nam, fig_desc,'VariableNames', {'Number'; 'Name'; 'Des
    fig_tab(1,:)=[]; % clear the table
    disp("The figure table has been initialized.");

    fun_num = 0; % initialize counter
    fun_nam = ""; % initialize function-name string array
    fun_args = ""; %initialize function-argument string array
```

```
    fun_desc = ""; % initialize function-description string array;
    fun_tab = table(fun_num, fun_nam, fun_args, fun_desc, ...
        'VariableNames', {'Number'; 'Name'; 'Arguments'; 'Description'});
    fun_tab(1,:)=[]; % clear the table
    disp("The function table has been initialized.");

    sym_num = 0; % initialize counter
    sym_nam = ""; % initialize symbol-name string array
    sym_desc = ""; % initialize symbol-description string array;
    sym_tab = table(sym_num, sym_nam, sym_desc,'VariableNames', {'Number'; 'Name'; 'Des
    sym_tab(1,:)=[]; % clear the table
    disp("The symbol table has been initialized.");

    [fun_num, fun_tab] = add_fun(fun_num, fun_tab, "init_all", "", "initialize tables")
    [fun_num, fun_tab] = add_fun(fun_num, fun_tab, "add_eqn", "eqn_num, eqn_tab, EqnNam
        "add equation to table");
    [fun_num, fun_tab] = add_fun(fun_num, fun_tab, "add_fig", "fig_num, fig_tab, FigNam
        "add figure to table");
    [fun_num, fun_tab] = add_fun(fun_num, fun_tab, "add_fun", "fun_num, fun_tab, FunNam
        "add local function to table");
    [fun_num, fun_tab] = add_fun(fun_num, fun_tab, "add_sym", "sym_num, sym_tab, SymNam
        "add symbol to table");
    [fun_num, fun_tab] = add_fun(fun_num, fun_tab, "FilterFun", "F, Fbend, Fro",...
        "Frequency-following function");
    [fun_num, fun_tab] = add_fun(fun_num, fun_tab, "FilterFunBS", "Fbend, Fro, Frate, F
        "Back-solution of frequency-following function");
    [fun_num, fun_tab] = add_fun(fun_num, fun_tab, "LogistNormFun", "exponent, input, l
        "Rising logistic function");
    [fun_num, fun_tab] = add_fun(fun_num, fun_tab, "LogistNormBsFun", "exponent, locati
        "Back-solution of logistic function to return the input");
    [fun_num, fun_tab] = add_fun(fun_num, fun_tab, "LogistNormBsLocFun", "exponent, inp
        "Back-solution of logistic function to return the location parameter");
    [fun_num, fun_tab] = add_fun(fun_num, fun_tab, "PsubFun", "Pobj, PsubBend, PsubMin'
        "Subjective-price function");
    [fun_num, fun_tab] = add_fun(fun_num, fun_tab, "PsubBsFun", "Psub, PsubBend, PsubMi
        "Back-solution of subjective-price function");
    [fun_num, fun_tab] = add_fun(fun_num, fun_tab, "ScalarDivFun", "dividend, divisor",
        "Scalar-division function");
    [fun_num, fun_tab] = add_fun(fun_num, fun_tab, "ScalarDivBsFun", "divisor, quotient
        "Back-solution of scalar-division function");
    [fun_num, fun_tab] = add_fun(fun_num, fun_tab, "ScalarMultFun", "multiplicand, mult
        "Scalar-multiplication function");
    [fun_num, fun_tab] = add_fun(fun_num, fun_tab, "ScalarMultBsFun", "multiplier, prod
        "Back-solution of scalar-multiplication function");
    [fun_num, fun_tab] = add_fun(fun_num, fun_tab, "PsubEfun", "Ceff, Rmax, Ue",...
        "Function to compute subjective equivalent of the objective-price location para
    [fun_num, fun_tab] = add_fun(fun_num, fun_tab, "PobjEfun", "Ceff, PsubBend, PsubMin
        "Function to compute objective-price location parameter");
end % end of init_all function

function [eqn_num, eqn_tab] = add_eqn(eqn_num, eqn_tab, EqnNam, EqnDesc)
    if any(strcmp(EqnNam,eqn_tab.Name))
       disp(strcat({'Equation '}, EqnNam, {' has already been entered.'}));
       return;
```

```
    else
        eqn_num = eqn_num+1;
        eqn_tab(eqn_num,:) = {eqn_num, ...
            string(EqnNam), ...
            string(EqnDesc)};
        disp(strcat({'This is equation #'} , num2str(eqn_tab.Number(height(eqn_tab))),
            {': ('}, eqn_tab.Name(height(eqn_tab)), {')'}));
    end
end

function [fig_num, fig_tab] = add_fig(fig_num, fig_tab, FigNam, FigDesc)
    if any(strcmp(FigNam,fig_tab.Name))
       disp(strcat({'Figure '}, FigNam, {' has already been entered.'}));
       return;
    else
        fig_num = fig_num+1;
        fig_tab(fig_num,:) = {fig_num, ...
            string(FigNam), ...
            string(FigDesc)};
        disp(strcat({'This is figure #'} , num2str(fig_tab.Number(height(fig_tab))), ..
            {': ('}, fig_tab.Name(height(fig_tab)), {')'}));
    end
end

function [fun_num, fun_tab] = add_fun(fun_num, fun_tab, FunNam, FunArgs, FunDesc)
    if any(strcmp(FunNam,fun_tab.Name))
       disp(strcat({'Function '}, FunNam, {' has already been entered.'}));
       return;
    else
        fun_num = fun_num+1;
        fun_tab(fun_num,:) = {fun_num, ...
            string(FunNam), ...
            string(FunArgs), ...
            string(FunDesc)};
        disp(strcat({'This is function #'} , num2str(fun_tab.Number(height(fun_tab))),
            {': ('}, fun_tab.Name(height(fun_tab)), {')'}));
    end
end

function [sym_num, sym_tab] = add_sym(sym_num, sym_tab, SymNam, SymDesc)
    if any(strcmp(SymNam,sym_tab.Name))
       disp(strcat({'Symbol '}, SymNam, {' has already been entered.'}));
       return;
    else
        sym_num = sym_num+1;
        sym_tab(sym_num,:) = {sym_num, ...
            string(SymNam), ...
            string(SymDesc)};
%         disp(strcat({'This is symbol #'} , num2str(sym_tab.Number(height(sym_tab))),
%             {': ('}, sym_tab.Name(height(sym_tab)), {')'}));
    end
end

function FigDir = set_figdir(figdirnam, graphs2files)
```

```
    global Figdir
    FigDir = fullfile(pwd,figdirnam);
    if graphs2files
        if ~exist(FigDir, 'dir')
           mkdir(FigDir);
        end
    end
end

function ImpFigDir = set_impfigdir(impfigdirnam)
    ImpFigDir = fullfile(pwd,impfigdirnam);
    if ~exist(ImpFigDir, 'dir')
        disp(strcat({'Exiting because Imported-files directory '}, impfigdirnam, {' doe
        return
    end
end

function tabdir = set_tabdir(tabdirnam, tabs2files)
    tabdir = fullfile(pwd,tabdirnam);

    if tabs2files
        if ~exist(tabdir, 'dir')
           mkdir(tabdir);
        end
    end
end
```

Functional building blocks for the simulations

```
function Frate = FilterFun(F, Fbend, Fro) % Frequency-following function
    Frate = Fbend.*(log(1+exp(Fro./Fbend))-log(1+exp((Fro-F)./Fbend)));
end
% see:
% Breton, Y.-A., Mullett, A., Conover, K., & Shizgal, P. (2013).
% Validation and extension of the reward-mountain model.
% Frontiers in Behavioral Neuroscience, 7, 125. https://doi.org/10.3389/fnbeh.2013.0012
% Solomon, R. B., Trujillo-Pisanty, I., Conover, K., & Shizgal, P. (2015).
% Psychophysical inference of frequency-following fidelity in the neural substrate for
% Brain Research, 292, 327-341. https://doi.org/10.1016/j.bbr.2015.06.008

% Last revised by Peter Shizgal on 2019-09-05 09:57
function Fpulse = FilterFunBS(Fbend, Fro, FF, FPmax) % Back-solution of frequency-follo
    % Estimate the maximum achievable firing rate, given FPmax, Fbend, and Fro
    FFmax = FilterFun(FPmax, Fbend, Fro);
    FpulseRI = Fro - Fbend.*log(exp(-FF/Fbend).*(exp(Fro/Fbend) + 1) - 1);
    % FpulseRI is a complex number. When FF < FFmax, the imaginary part = zero. When Fp
    % We retain the real component when FF < FFmax & >= 0. returning NaN when FF > FFma
    sz = size(FF);
    Fpulse = zeros(sz); % pre-allocate

    neg = FF < 0;
    below = (FF < FFmax & FF >= 0);
    eq = FF == FFmax;
    above = FF > FFmax;
```

```
    Fpulse(neg) = NaN;
    Fpulse(below) = real(FpulseRI(below)); % Complex portion, if it exists, equals zero
    Fpulse(eq) = Inf;
    Fpulse(above) = NaN;
end
% This back-solution can be verified from the following code snippet, which requires th
% Matlab Symbolic-Math Toolbox. It is commented out here to allow this Live Script to r
% on systems that lack the Symbolic-Math toolbox.
% syms FdaRate FdaBend FdaRO Fopt positive;
% simplify(solve(FdaRate == FdaBend * (log(1+exp(FdaRO/FdaBend)) - (log(1+exp((FdaRO-Fo
% Fopt = FdaRO - FdaBend*log(exp(-FdaRate/FdaBend)*(exp(FdaRO/FdaBend) + 1) - 1)
%%
% This function returns imaginary numbers. The imaginary portion equals zero provided t
% is less than its maximum achievable value. That maximum is estimated from the forward
% as the input.
%%
% see:
% Breton, Y.-A., Mullett, A., Conover, K., & Shizgal, P. (2013).
% Validation and extension of the reward-mountain model.
% Frontiers in Behavioral Neuroscience, 7, 125. https://doi.org/10.3389/fnbeh.2013.0012
% Solomon, R. B., Trujillo-Pisanty, I., Conover, K., & Shizgal, P. (2015).
% Psychophysical inference of frequency-following fidelity in the neural substrate for
% Behavioural Brain Research, 292, 327-341. https://doi.org/10.1016/j.bbr.2015.06.008

function output = LogistNormFun(exponent, input, location)
    output = ...
            ((input .^ exponent) ./...
            ((input .^ exponent) + (location .^ exponent)));
end
% The symbol for the logistic function in the flow diagrams is a rising S-shaped curve
%

% Revised by Peter Shizgal on 2019-10-29 21:25
% The argument called "output" must be scalar
function input = LogistNormBsFun(exponent, location, output)
% Logistic function back-solved to return the input
    if (output >= 1) || (output <= 0)
        disp("Illegal value. Call with 0 < output < 1");
        return
    end

    input = location .* (output./(1-output)).^(1./exponent);
end

% Revised by Peter Shizgal on 2019-10-29 21:25
function location = LogistNormBsLocFun(exponent, input, output)
% The argument called "output" must be scalar
% Logistic function back-solved to return the location parameter
    if  output >= 1 || output <= 0
        disp("Illegal value. Call with 0 < output < 1");
        return
    end
```

```
    location = input .* ((1 - output) ./output).^ (1./exponent);
end

% The symbol for the subjective-price function in the flow diagrams is a
% hockey-stick-shaped curve in a box.
%
% Solomon, R. B., Conover, K., & Shizgal, P. (2017).
% Valuation of opportunity costs by rats working for rewarding electrical brain stimula
% PLOS ONE, 12(8), e0182120. https://doi.org/10.1371/journal.pone.0182120
function Psub = PsubFun(Pobj, PsubBend, PsubMin) % Subjective-price function
    Psub = PsubMin + PsubBend .* (log(1 + exp((Pobj - PsubMin)./PsubBend)));
end

% The equation for PsubBsFun will return -Inf when Psub = PsubMin and a complex number
% than PsubMin. When Psub includes values between PsubMin and Pobj_0 (the value of Pobj
% PsubBsFun returns negative values. The definition of PsubBsFun has thus been couched
% > 0 when Psub > Pobj_0, 0 when Psub = Pobj_0, and NaN otherwise. The reason that 0 is
% is to ensure consistency between the forward and backward solutions.
% Last revised by Peter Shizgal on 2019-09-08 15:06
function Pobj = PsubBsFun(Psub, PsubBend, PsubMin) % Back-solution of subjective-price
    Psub_Pobj_0 = PsubMin + PsubBend .* log(1 + exp((-PsubMin)./PsubBend));
    above = Psub > Psub_Pobj_0;
    eq = Psub == Psub_Pobj_0;
    below = Psub < Psub_Pobj_0;

    Pobj = PsubMin + PsubBend .* log(-1 + exp((Psub(above) - PsubMin)./PsubBend));
    % Output will be real because Psub(above) > the value that drives Pobj to zero
    Pobj(eq) = 0;
    Pobj(below) = NaN;
end

% The next four functions are defined for consistency in formatting when composing more
% five building blocks (FilterFun, PsubFun, LogistNormFun, ScalarDivFun, ScalarMultFun)
% six back-solutions (FilterFunBS, PsubBsFun, LogistNormBsFun, LogistNormBsLocFun, Scal
% Obviously, ScalarDiv function == "/", ScalarDivBsFun == "*", ScalarMultFun == "*", an
% ScalarMultBsFun == "/".

function quotient = ScalarDivFun(dividend, divisor)
    quotient = (dividend ./ divisor);
end

function dividend = ScalarDivBsFun(divisor, quotient)
    % Back solution of the scalar-division function (scalar multiplication)
    dividend = (quotient .* divisor);
end

function product = ScalarMultFun(multiplicand, multiplier)
    product = (multiplicand .* multiplier);
end
% Scalar multiplication is represented in the flow diagrams in three ways:
% 1) as a product operator (Pi notation) in a box (to compute the aggregate firing rate
% 2) as a right-facing triangle (representing amplification), and
% 3) as a standard multiplication sign
```

```
function multiplicand = ScalarMultBsFun(multiplier, product)
    % Back solution of the scalar-multiplication function (scalar division)
    multiplicand = (product ./ multiplier);
end
```

Functions composing the reward-mountain model

```
% Dummy function to relate the duration of the burst of increased firing in the directl
% the pulse frequency. We set the two to be equal, a reasonable assumption if frequency
% high.
function Dburst = fD(Dtrain)
    Dburst = Dtrain;
end

% Function to compute the aggregate rate of firing required to produce a reward of half
% see: Sonnenschein, B., Conover, K., & Shizgal, P. (2003).
% Growth of brain stimulation reward as a function of duration and stimulation strength
% Behavioral Neuroscience, 117(5), 978-994. http://psycnet.apa.org/journals/bne/117/5/9

function FFaggHM = fH(C, D, RhoPi)
    FFaggHM = ScalarMultFun(...
                          RhoPi,...
                          (1 + ScalarDivFun(...
                                           C,...
                                           D...
                                           )...
                           )...
                         );
end

% Function to compute the average rate of firing required to produce a reward of half-m
% see: Sonnenschein, B., Conover, K., & Shizgal, P. (2003).
% Growth of brain stimulation reward as a function of duration and stimulation strength
% Behavioral Neuroscience, 117(5), 978-994. http://psycnet.apa.org/journals/bne/117/5/9
function FFhm = fHfiring(C, D, N, RhoPi, varargin)
    if size(varargin,2) == 0
        FFhm = ScalarDivFun(...
                          fH(...
                                    C, ...
                                    D, ...
                                    RhoPi...
                                    ),...
                          N...
                          );
    else
        Kin = varargin{1};
        FFhm = ScalarDivFun(...
                          fH(...
                                    C, ...
                                    D, ...
                                    ScalarDivFun(...
                                                 RhoPi,...
                                                 Kin...
```

```
                                                  )...
                                     ),...
                           N...
                           );
    end
end

% Function to compute the average pulse frequency required to produce a reward of half-
% see: Sonnenschein, B., Conover, K., & Shizgal, P. (2003).
% Growth of brain stimulation reward as a function of duration and stimulation strength
% Behavioral Neuroscience, 117(5), 978-994. http://psycnet.apa.org/journals/bne/117/5/9
function FpulseHM = FpulseHMfun(C, D, Fbend, FPmax, Fro, N, RhoPi, varargin)
    if size(varargin,2) == 0
        FpulseHM = FilterFunBS(...
                               Fbend, ...
                               Fro, ...
                               fHfiring(C, ...
                                      D, ...
                                      N, ...
                                      RhoPi),...
                               FPmax...
                               );
    else
        Kin = varargin{1};
        FpulseHM = FilterFunBS(...
                               Fbend, ...
                               Fro, ...
                               fHfiring(C, ...
                                      D, ...
                                      N, ...
                                      RhoPi,...
                                      Kin...
                                      ),...
                               FPmax...
                               );
    end
end

% Function to find the pulse frequency that produces a reward of half-maximal intensity
% reward intensity
% FFmax, the maximal induced firing rate is obtained by setting the pulse frequency to
function FPhmPG = find_FhmPG(FPmax, Fbend, Fro, g, Kin, Kout, RmaxPG)
    FFmax = FilterFun(FPmax,Fbend,Fro);
    FFhmPG = Kin .* ((0.5 .* RmaxPG) ./ Kout).^(1./g);
    FPhmPG = FilterFunBS(Fbend, Fro, FFhmPG, FPmax);
end

% Function to translate the effect of an electrical input to the directly stimulated mf
% of the series circuit model into an equivalent optical input to the dopaminergic stag
% Both inputs produce the same level of firing in the dopamine neurons.
function FoptEquiv = FoptEquivFun(Felec, FmfbBend, FmfbHM, FmfbRO, gMFB, KrgE)
    FoptEquiv = fRbsr(...
                      Felec,...
                      FmfbBend,...
```

```
                      FmfbHM,...
                      FmfbRO,...
                      gMFB,...
                      KrgE);
end

% Function to compute the equivalent optical drive on midbrain dopamine neurons
% due to trans-synaptic input from electrically excited MFB neurons
function FmfbDAdrive = FmfbDAdriveFun(FmfbDAbend, Felec, FFmfbDAmax, FoptEquiv, FmfbDAr
    FmfbDAdrive = ScalarMultFun(...
                              ScalarDivFun(...
                                            FilterFun(...
                                                      Felec,...
                                                      FmfbDAbend,...
                                                      FmfbDAro...
                                                      ),... % Filtered firing rate in M
                                            FFmfbDAmax...
                                        ),... % Proportion of maximum firing rate achie
                              FoptEquiv...
                              ); % Maximum equivalent optical pulse frequency applied t
end

% Find the electrical pulse frequency that produces half-maximal, summated reward inter
% The solution is obtained by means of interpolation
%function [FsumHM, RsumMax] = find_FhmSum(Felec, Rsum)
function [FsumHM, RsumMax, CindAbove, CindBelow] = find_FhmSum(Felec, Rsum)
    RsumMax = max(Rsum, [], 2); % max along each row
    Rcrit = RsumMax./2;
    RsumHMlog = Rsum > Rcrit;
    RsumHMlogDiff = diff(RsumHMlog==1,1,2); % indices (offset by 1) marking transition
%     RsumHMlogDiff = diff(RsumHMlog); % indices (offset by 1) marking transition from
    siz = size(RsumHMlogDiff);
    IndDiffAbove = find(RsumHMlogDiff==1); % linear indices for first elements > Rcrit
    [Rind Cind] = ind2sub(siz,IndDiffAbove);
    % row & column indices for first elements > Rcrit offset by -1
    CindSort(Rind) = Cind; % column indices sorted by row number of Rsum matrix
    CindAbove = CindSort + 1; % row index of value just above Fh
    CindBelow = CindSort;

    % find exact Fhm by interpolation
    siz = size(Rsum); % one more column than diff matrix
    IndAbove = sub2ind(siz,1:length(Rind),CindAbove);
    IndBelow = IndAbove - siz(1);
    % The indices increment down the columns, so adjacent row indices are separated by
    RsumAbove = Rsum(IndAbove);
    RsumBelow = Rsum(IndBelow);
    slp = (RsumAbove - RsumBelow) ./ (Felec(CindAbove) - Felec(CindBelow));
    FsumHM = (((Rcrit' - RsumBelow) ./ slp) + Felec(1,CindBelow))';
    % All rows of Felec are the same
    % Rcrit is a column vector but RsumBelow and Felec are row vectors
    % Return FsumHM as a column vector so that it has the same dimensions as RsumMax
end

% Function to compute the normalized value of the reward-growth function
```

```
% F is a pulse frequency, whereas FFhm is a firing frequency
function RbsrNorm = fRbsrNorm(F, Fbend, FFhm, Fro, g)
   RbsrNorm = LogistNormFun(...
                     g,...
                     FilterFun(...
                               F,...
                               Fbend,...
                               Fro...
                               ),...
                     FFhm...
                     );
end

% Function to compute the scaled value of the reward-growth function
% F is a pulse frequency, whereas Fhm is a firing frequency
function Rbsr = fRbsr(F, Fbend, Fhm, Fro, g, Krg)
   Rbsr = ScalarMultFun(...
                     Krg,...
                     LogistNormFun(...
                                   g,...
                                   FilterFun(...
                                             F,...
                                             Fbend,...
                                             Fro...
                                             ),...
                                   Fhm...
                                   )...
                     );
end

function Rbsr = fRbsrFull(C, D, F, Fbend, Fro, g, Krg, N, RhoPi, varargin)
    if size(varargin,2) == 0
        Rbsr = ScalarMultFun(...
                          Krg,...
                          LogistNormFun(...
                                        g,...
                                        FilterFun(...
                                                  F,...
                                                  Fbend,...
                                                  Fro...
                                                  ),...
                                       fHfiring(...
                                              C, ...
                                              D, ...
                                              N, ...
                                              RhoPi...
                                              )...
                                       )...
                         );
    else
        Kin = varargin{1};
        Rbsr = ScalarMultFun(Krg,...
                          LogistNormFun(g,...
                                       FilterFun(...
```

```
                                                F,...
                                                Fbend,...
                                                Fro...
                                                ),...
                                        fHfiring(...
                                              C, ...
                                              D, ...
                                              N, ...
                                              RhoPi,...
                                              Kin...
                                              )...
                                        )...
                          );
    end
end

% Back solution of the full reward-growth function for the series-circuit (sc) model
% KrgE translates the normalized output of the upstream reward-growth function into
% the equivalent of an optical pulse frequency applied to the dopamine neurons
function FscHM = FscHMbs(FhmDA, FmfbBend, FhmMFB, FmfbRO, FPmax, gMFB, KrgEq)
    FscHM = FilterFunBS(... % Electrical pulse frequency that produces HM reward intens
                        FmfbBend,...
                        FmfbRO,...
                        LogistNormBsFun(... % MFB firing-rate that produces HM reward ir
                                       gMFB,... % exponent of MFB RG function
                                       FhmMFB,... % location parameter of MFB RG functic
                                       ScalarMultBsFun(... % normalized output of MFB RC
                                                      KrgEq,...
                                                      FhmDA...
                                                      )... % normalized output of MFB RC
                                       ),...  % MFB firing-rate that produces HM reward
                         FPmax... % pulse frequency sufficient to maximize firing
                         ); % Electrical pulse frequency that produces HM reward intensi
end

% Power function to compute reward growth
% Kin scales the firings, and the product is raised to the power of g
function R = fRpg(F, Fbend, Fro, g, Kin, Kout)
   R = ScalarMultFun(...
                     Kout,...
       ScalarDivFun(...
                                 FilterFun(F,...
                                           Fbend,...
                                           Fro...
                                           ),...
                                 Kin...
                                 )...
                                  .^g...
                     );
end
% Power function to compute reward growth, normalized to output values between 0 & 1
% A pulse frequency of 1000 is used to estimate Rmax
function Rnorm = RGpgNormFun(F, Fbend, Fro, g, Kin, Kout)
   Rnorm = ScalarDivFun(...
```

```
                             fRpg(F, Fbend, Fro, g, Kin, Kout),...
                             fRpg(1000, Fbend, Fro, g, Kin, Kout)...
                             );
end

% Dummy function to compute subjective probability (output = input)
% Breton, Y.-A., Conover, K., & Shizgal, P. (2014).
% The effect of probability discounting on reward seeking: a three-dimensional perspect[illegible]
% Frontiers in Behavioral Neuroscience, 8, 284. https://doi.org/10.3389/fnbeh.2014.0028[illegible]
% found that subjective and objective reward probabilites in an eICSS experiment
% were indistiguishable when the objective probability was 0.5 or greater.
% Thus, subjective and objective probabilities are equated here.
% We do not know at what point this equivalence breaks down, so use of
% this function is invalid for objective probabilities <0.5.
function ProbSub = ProbSubFun(ProbObj)
    ProbSub = ProbObj;
end

% Function to compute subjective effort cost (output = input)
% This function is certainly not scalar! However, we haven't measured it yet.
% We make the assumption that the rate of subjective exertion is held constant
% under the conditions of our experiment, and we thus impose a fixed value on the subje[illegible]
% Due to factors such as fatigue, this can't be entirely right. We hope
% that violations of this assumption aren't serious.
% To accommodate the hypothesis that subjective effort costs are modulated by dopamine[illegible]
% the optional KeffMod parameter scales the subjective effort cost.
% We use this function to set the subjective rates of exertion for both work and leisur[illegible]
function dotPhiSubScaled = fphiSc(dotPhiSub, Kec, varargin)
    if size(varargin,2) == 0
        dotPhiSubScaled = ScalarMultFun(...
                               dotPhiSub,...
                               Kec...
                               );
    else
        KeffMod = varargin{1};
        dotPhiSubScaled = ScalarMultFun(...
                               dotPhiSub,...
                               ScalarDivFun(...
                                            Kec,...
                                            KeffMod...
                                            )...
                               );
    end
end

% Dummy function to compute subjective value of alternate ("leisure") activities (outpu[illegible]
% Again, we impose a fixed value, faute de mieux.
function RaaScaled = fRaa(Raa,Kaa)
    RaaScaled = ScalarMultFun(...
                          Raa,...
                          Kaa...
                          );
end
```

```
% Function to compute the subjective equivalent of the objective-price location paramet[illegible]
function PsubE = PsubEfun(dotPhiObj, Kaa, Kec, Krg, Raa, pObj, RnormMax, varargin)
    if size(varargin,2) == 0
        PsubE = ScalarDivFun(...
                             ScalarMultFun(...
                                           ProbSubFun(pObj),...
                                           ScalarMultFun(... % compute Rmax
                                                         Krg,...
                                                         RnormMax...
                                                         )...
                                           ),...
                             ScalarMultFun(...
                                           fphiSc(dotPhiObj, Kec),...
                                           fRaa(Raa, Kaa)...
                                           )...
                             );
    else
        KeffMod = varargin{1};
            PsubE = ScalarDivFun(...
                                 ScalarMultFun(...
                                               ProbSubFun(pObj),...
                                               ScalarMultFun(... % compute Rmax
                                                             Krg,...
                                                             RnormMax...
                                                             )...
                                               ),...
                                 ScalarMultFun(...
                                               fphiSc(dotPhiObj, Kec, KeffMod),...
                                               fRaa(Raa, Kaa)...
                                               )...
                                 );
    end
end

% Function to compute the subjective-price location parameter for the power-growth mode[illegible]
function PsubEpg = PsubEpgFun(dotPhiObj, Kaa, Kec, Krg, ObjAA, pObj, Rmax)
    PsubEpg = ScalarDivFun(...
                           ScalarMultFun(...
                                         Rmax,...
                                         ProbSubFun(pObj)...
                                         ),...
                           ScalarMultFun(...
                                         fphiSc(dotPhiObj, Kec),...
                                         fRaa(ObjAA, Kaa)...
                                         )...
                           );
end

% Function to compute the objective-price location parameter
% This is the back-solution of the function that computes PsubE
function PobjE = PobjEfun(dotPhiObj, Kaa, Kec, Krg, ObjAA, pObj, PsubBend, PsubMin, Rno[illegible]
    if size(varargin,2) == 0
        PobjE = PsubBsFun(...
                            PsubEfun(dotPhiObj, Kaa, Kec, Krg, ObjAA, pObj, RnormMax), ..[illegible]
```

```
                            PsubBend, ...
                            PsubMin...
                            );
    else
        KeffMod = varargin{1};
        PobjE = PsubBsFun(...
                            PsubEfun(dotPhiObj, Kaa, Kec, Krg, ObjAA, pObj, RnormMax, Kef
                            PsubBend, ...
                            PsubMin...
                            );
    end
end

% Function to compute time allocation
% This function builds the mountain surface
% The values of the location parameters, FpulseHM & PobjE, are inputs
% These must be computed before this function is called
function T = TAfun(a, F, Fbend, FpulseHM, Fro, g, Pobj, PobjE, PsubBend, PsubMin, Rnorm
    T = LogistNormFun(...
                      a, ...
                      fRbsrNorm(...
                                F, ...
                                Fbend, ...
                                FpulseHM, ...
                                Fro, ...
                                g...
                                ), ...
                      ScalarDivFun(...
                                   ScalarMultFun(...
                                                 PsubFun(Pobj, ...
                                                         PsubBend, ...
                                                         PsubMin),...
                                                         RnormMax...
                                                 ),...
                                   PsubFun(PobjE, ...
                                           PsubBend, ...
                                           PsubMin)...
                                   )...
                      );
end

% Function to compute time allocation using power growth of reward intensity
% This function builds the mountain surface
% The values of the location parameter, PsubEpg, is an input and must be computed befor
function T = TApgFun(a, F, Fbend, Fro, g, Kin, Kout, Pobj, PsubBend, PsubEpg, PsubMin)
    T = LogistNormFun(...
                      a, ...
                      RGpgNormFun(F, Fbend, Fro, g, Kin, Kout),...
                      ScalarDivFun(...
                                   PsubFun(Pobj, ...
                                           PsubBend, ...
                                           PsubMin),...
                                   PsubEpg...
                                   )...
```

```
                       );
end

% Function to compute time allocation for the convergence model
% The reward-growth function must be computed beforehand. Its output, RGvec, must be a
function T = TAsumFun(a, Pobj, PobjE, PsubBend, PsubMin, RGvec, RnormMax)
    T = LogistNormFun(...
                       a, ...
                       RGvec, ...
                       ScalarDivFun(...
                                    ScalarMultFun(...
                                                  PsubFun(Pobj, ...
                                                          PsubBend, ...
                                                          PsubMin),...
                                                          RnormMax...
                                                  ),...
                                    PsubFun(PobjE, ...
                                            PsubBend, ...
                                            PsubMin)...
                                    )...
                       );
end
```

Functions that plot graphs and set attributes

```
% Plot of the frequency-following function
% function FF_graph = plot_freqFoll(F, FR, fnroot, graphs2files, figdir, varargin)
function FF_graph = plot_freqFoll(F, FR, fnroot, varargin)
    global graphs2files FigDir
    if size(varargin,2) == 0
        % To accommodate multiple plots based on input matrices, must compute {min, max
        % Revise this section to operate in the log domain and then transform back to l
        xmin = min(min(F)) - (0.1*(max(max(F))-min(min(F))));
        xmax = max(max(F)) + (0.1*(max(max(F))-min(min(F))));
        ymin = min(min(FR)) - (0.1*(max(max(FR))-min(min(FR))));
        ymax = max(max(FR)) + (0.1*(max(max(FR))-min(min(FR))));
    else
        xmin = varargin{1};
        xmax = varargin{2};
        ymin = varargin{3};
        ymax = varargin{4};
    end

    FF_graph = figure;
    FF_graph.Visible = 'off';
    FF_graph.Position = [0 0 600 600];
    gmfbfr = loglog(F,FR);
%    gmfbfr.LineWidth = 4;
    arrayfun(@(x) set(x,'LineWidth',4), gmfbfr); % Needed if plot includes multiple lin
    grid on;
    ax1 = gca;
    ax1.XLim = [xmin xmax];
    ax1.YLim = [ymin ymax];
    ax1.XLabel.String = 'Pulse Frequency_{ }';
```

```
    ax1.YLabel.String = 'Firing Frequency_{ }';
    ax1.FontSize = 24;
    ax1.XLabel.FontAngle = 'italic';
    ax1.YLabel.FontAngle = 'italic';
    arrayfun(@(x) set(ax1, 'XTickLabel', x), {num2str(ax1.XTick')});
    arrayfun(@(x) set(ax1, 'YTickLabel', x), {num2str(ax1.YTick')});

    if graphs2files
        saveas(gca,fullfile(FigDir,strcat(fnroot,'_freq_resp.fig')));
        saveas(gca,fullfile(FigDir,strcat(fnroot,'_freq_resp.png')));
    end
end

% Plot of the strength-duration function for trains
function SDT_graph = plot_StrDurTrains(Delec,FelecHM)
    global graphs2files FigDir
    SDT_graph = figure;
    SDT_graph.Visible = 'off';
    SDT_graph.Position = [0 0 600 600];
    gmfbfr = loglog(Delec,FelecHM);
    gmfbfr.LineWidth = 4;
    grid on;
    ax1 = gca;
    ymax = max(FelecHM)*10^0.1;
    ymin = min(FelecHM)*10^-0.1;
    ax1.YLim = [ymin ymax];
    ax1.XLabel.String = 'Train Duration (s)_{ }';
    ax1.YLabel.String = 'F_{hm }';
    ax1.FontSize = 24;
    ax1.XLabel.FontAngle = 'italic';
    ax1.YLabel.FontAngle = 'italic';
    ax1.XTickLabels = {'0.1', '1', '10', '100'};

    if graphs2files
        saveas(gca,fullfile(FigDir,'mfb_StrDurTrains.fig'));
        saveas(gca,fullfile(FigDir,'mfb_StrDurTrains.png'));
    end
end

% Plot one or more reward-growth functions
function out_graph = plot_RG(Fmat,RMat,pnam,pVec,fnam,TitleStr,linlog, varargin)
    global graphs2files FigDir
    fh = figure;
    fh.Position = [0 0 600 600];
    fh.Visible = 'off';

    plab = regexprep(regexprep(pnam,'\W',''),'_','');

    switch linlog
        case 'lin' % linear y-axis, logarithmic x-axis
            gRG = semilogx(Fmat,RMat);
            ymin = -0.05;
            ymax = max(max(RMat)) * 1.05;
            if graphs2files
```

```
                if size(varargin,2) == 0
%                      figfile = fullfile(FigDir,char(strcat('RG_',fnam','_','semilog.fi
%                      pngfile = fullfile(FigDir,char(strcat('RG_',fnam','_','semilog.pr
                    figfile = fullfile(FigDir,char(strcat('RG_',fnam,'_','semilog.fig')
                    pngfile = fullfile(FigDir,char(strcat('RG_',fnam,'_','semilog.png')
                else
                    name_suffix = varargin{1};
                    figfile = ...
                        fullfile(FigDir,char(strcat('RG_',fnam','_',name_suffix,'_','se
                    pngfile = ...
                        fullfile(FigDir,char(strcat('RG_',fnam','_',name_suffix,'_','se
                end
            end
        case 'log' % logarithmic y-axis, logarithmic x-axis
            gRG = loglog(Fmat,RMat);
            ymin = 0.01;
            ymax = max(max(RMat)) * 10^0.1;
            if graphs2files
                if size(varargin,2) == 0
                    figfile = fullfile(FigDir,char(strcat('RG_',fnam','_','loglog.fig')
                    pngfile = fullfile(FigDir,char(strcat('RG_',fnam','_','loglog.png')
                else
                    name_suffix = varargin{1};
                    figfile = ...
                        fullfile(FigDir,char(strcat('RG_',fnam','_',name_suffix,'_','lo
                    pngfile = ...
                        fullfile(FigDir,char(strcat('RG_',fnam','_',name_suffix,'_','lo
                end
            end
    end

    for j = 1:length(gRG)
        gRG(j).LineWidth = 4;
    end
    grid on;
    ax1 = gca;
    ax1.Position = [0.175 0.15 0.75 0.75];
    ax1.XLim = [10 1000];
    ax1.YLim = [ymin ymax];
    ax1.XLabel.String = 'Pulse Frequency_{ }';
    ax1.YLabel.String = 'Reward Intensity_{ }';
    ax1.FontSize = 24;
    ax1.XLabel.FontAngle = 'italic';
    ax1.YLabel.FontAngle = 'italic';
    ax1.XTick = [1 10 100 1000];
    ax1.XTickLabels = {'1', '10', '100', '1000'};
    ax1.LineWidth = 1;

    switch linlog
        case 'lin'
            XlabPos = ax1.XLabel.Position;
            XlabPos(2) = ax1.YLim(1) - ((ax1.YLim(2) - ax1.YLim(1)) * 0.075); % 7.5% be
            ax1.XLabel.Position = XlabPos;
        case 'log'
```

```
            XlabPos = ax1.XLabel.Position;
            XlabPos(2) = 10^(log10(ax1.YLim(1)) - ((log10(ax1.YLim(2)) - log10(ax1.YLim
            ax1.XLabel.Position = XlabPos;
    end
    YlabPos = ax1.YLabel.Position;
    YlabPos(1) = 10^(log10(ax1.XLim(1)) - ((log10(ax1.XLim(2)) - log10(ax1.XLim(1)))*0.
    ax1.YLabel.Position = YlabPos;

%     if min(pVec) < 0.01
%         formatstring = '%3.2e';
%     else
%         formatstring = '%0.2f';
%     end
    if min(pVec) < 10
        formatstring = '%3.2f';
    else
        formatstring = '%3.0f';
    end

% Make a cell array of the legend values and pass to the legend function
    lgnd = legend(arrayfun(@(x) sprintf(formatstring,x), pVec, 'UniformOutput', false),
                  'Location', 'best');
    lgnd.Title.String = strjoin(pnam,'\n');

    title(ax1, TitleStr, 'FontSize', 30);

    out_graph = gcf;

    if graphs2files
        saveas(gca,figfile);
        saveas(gca,pngfile);
    end
end

% Plot of the subjective-price function
function SP_graph = plot_PsubFun(Pobj,Psub,graphs2files, figdir)
    SP_graph = figure;
    SP_graph.Visible = 'off';
    SP_graph.Position = [0 0 600 600];

    gPsub = loglog(Pobj,Psub);
    gPsub.LineWidth = 4;
    grid on;
    ax1 = gca;
    ax1.XLim = [0.1 100];
    ax1.YLim = [0.5 100];
    ax1.XTickLabels = {'0.1', '1', '10', '100'};
    ax1.YTickLabels = {'1', '10', '100'};
    ax1.LineWidth = 1;
    ax1.XLabel.String = 'Objective Price (s)_{ }';
    ax1.YLabel.String = 'Subjective Price (s)_{ }';
    ax1.FontSize = 24;
    ax1.XLabel.FontAngle = 'italic';
    ax1.YLabel.FontAngle = 'italic';
```

```
    if graphs2files
        saveas(gca,fullfile(figdir,'Subj_price.fig'));
        saveas(gca,fullfile(figdir,'Subj_price.png'));
    end
end

% Function to plot a single mountain
% F is a logarithmically spaced vector of pulse-frequencies
% Pobj is a logarithmically spaced vector of prices
% T is a square matrix of time-allocation values computed with price as the row variabl
% and pulse frequency as the column variable.
% Visible is a character vector ('on', or 'off') that determines whether or not the fig
% The optional arguments are xmin, xmax, ymin, ymax.
function gh3d2 = plot_MTN(F, Pobj, T, Visible, mtn_root, title_str, ...
    graphs2files, figdir, varargin)
    logF = log10(F);
    logP = log10(Pobj);
    [X,Y] = meshgrid(logP, logF);
    Z = T;

    if size(varargin,2) == 0
        xmin = 0;
        xmax = 2.6;
        ymin = 1;
        ymax = 3;
    else
        xmin = varargin{1};
        xmax = varargin{2};
        ymin = varargin{3};
        ymax = varargin{4};
    end

    gh3d = figure;
    gh3d.Visible = Visible;
    gh3d.Position = [0 0 600 600];
    surface(X, Y, Z, 'CData', Z,...
        'FaceLighting','gouraud',...
        'FaceColor',[0.5 0.5 0.5], 'FaceAlpha', 0.5,...
        'EdgeColor', [0.5 0.5 0.5],...
        'SpecularColorReflectance',0.5, 'SpecularExponent',4, 'SpecularStrength',1);
    light('Parent',gca,'Position',[10 -5 2]);
    view([40 30]);

    ax3d = findall(gca,'Type','Axes');
    title(ax3d, title_str, 'FontSize', 36);
    ax3d.Position = [0.15, 0.15, 0.75, 0.75];
    ax3d.FontSize = 16;
    ax3d.XLabel.String = 'Objective Price (s)';
    ax3d.XLabel.FontSize = 22;
    ax3d.XLabel.Rotation = -27;
    ax3d.XLabel.Units = 'Normalized';
    ax3d.XLabel.Position = [0.325 -0.0625 0];
    ax3d.XAxis.Scale = 'linear';
```

```
    ax3d.XLim = [xmin,xmax];
    ax3d.XTick = [0, 0.5, 1, 1.5, 2, 2.5];
    ax3d.XTickLabel = {'1.0','3.2','10','32','100','316'};
    ax3d.YLabel.String = 'Pulse frequency (pps)';
    ax3d.YLabel.FontSize = 22;
    ax3d.YLabel.Rotation = 32;
    ax3d.YLabel.Units = 'Normalized';
    ax3d.YLabel.Position = [0.85 0.05 0];
    ax3d.YAxis.Scale = 'linear';
    ax3d.YLim = [ymin,ymax];
    ax3d.YTick = [0, 0.5, 1, 1.5, 2, 2.5, 3];
    ax3d.YTickLabel = {'1','3.2','10','32', '100', '316', '1000'};
    ax3d.ZLim = [0,1.1];
    ax3d.ZLabel.String = 'Time Allocation';
    ax3d.ZLabel.FontSize = 22;
    ax3d.ZLabel.Rotation = 91;
    ax3d.ZLabel.Units = 'Normalized';
    ax3d.ZLabel.Position = [-0.125 0.475 0];
    ax3d.DataAspectRatio = [1 0.75 0.8];

    hold on;
    z_shim = 0.01;
    z_shim = Z + z_shim;
    contvec = 0.1:0.1:0.9;
    [~, ch] = contour3( X, Y, z_shim, contvec);
    colormap('jet');
    set(ch, 'LineWidth', 2);
    gh3d2 = figure(gcf);
    gh3d2.Visible = Visible;
    grid on;
    hold off;

    if graphs2files
        saveas(gca,fullfile(figdir,strcat(mtn_root,'.fig')));
        saveas(gca,fullfile(figdir,strcat(mtn_root,'.png')));
    end
end

% Function to plot a single contour graph
% F is a logarithmically spaced vector of pulse-frequencies.
% Pobj is a logarithmically spaced vector of prices.
% T is square matrix of time-allocation values computed with price as the row variable
% and pulse frequency as the column variable.
% Visible is a character vector ('on', or 'off') that determines whether or not the fig
% The optional arguments are xmin, xmax, ymin, ymax.
function cont1 = plot_contour(F, Pobj, T, Pobj_e, Fhm, Visible, mtn_root, ...
        title_str, annot_str, graphs2files, figdir, varargin)
    logF = log10(F);
    logP = log10(Pobj);
    [X,Y] = meshgrid(logP, logF);
    Z = T;

    if size(varargin,2) == 0
        xmin = 0;
```

```
        xmax = 2.6;
        ymin = 1;
        ymax = 3;
    else
        xmin = varargin{1};
        xmax = varargin{2};
        ymin = varargin{3};
        ymax = varargin{4};
    end

    contvec = 0.1:0.1:0.9;

    cont1 = figure;
    cont1.Visible = Visible;
    cont1.Position = [0 0 600 600];
    contour(X,Y,Z,contvec,'LineWidth',1.25);
    colormap('jet');
    hold on;

    axc1 = findall(gca,'Type','Axes');
    title(axc1, title_str, 'FontSize', 36);
    axc1.Position = [0.15, 0.175, 0.825, 0.75];
    axc1.FontSize = 18;

    axc1.XLim = [xmin,xmax];

    axc1.XLabel.String = 'Objective Price (s)';
    axc1.XLabel.FontSize = 24;
    % Adjust position of X-axis label
    xlpos = axc1.XLabel.Position;
    xlpos(2) = ymin - ((ymax - ymin) * 0.1);
    axc1.XLabel.Position = xlpos;

    axc1.XTick = [-2, -1.5, -1, -0.5, 0, 0.5, 1, 1.5, 2, 2.5];
    axc1.XTickLabel = {'','','0.1','0.32','1.0','3.2','10','32','100', '316'};

    axc1.YLim = [ymin,ymax];

    axc1.YLabel.String = 'Pulse Frequency (pps)';
    axc1.YLabel.FontSize = 24;
    % Adjust position of Y-axis label
    ylpos = axc1.YLabel.Position;
    ylpos(1) = xmin - ((xmax - xmin) * 0.125);
    axc1.YLabel.Position = ylpos;

    axc1.YTick = [0. 0.5, 1, 1.5, 2, 2.5, 3];
    axc1.YTickLabel = {'1', '3.2', '10', '31.6', '100', '316', '1000'};

    FhmLine1 = line('XData', axc1.XLim,'YData', [log10(Fhm), log10(Fhm)]);
    FhmLine1.Color = 'r';
    FhmLine1.LineWidth = 4;
    FhmLine1.LineStyle = '--';
    PeLine1 = line('XData', [log10(Pobj_e), log10(Pobj_e)],'YData', axc1.YLim);
    PeLine1.Color = 'b';
```

```
    PeLine1.LineWidth = 4;
    PeLine1.LineStyle = '--';

    tb2 = annotation('textbox');
    tb2.String = annot_str;
    tb2.FontSize = 18;
    tb2.Position = [0.7, 0.25, 0.15, 0.075];
    hold off;

    legend([FhmLine1, PeLine1], [{'F_{hm }'}, {'P_e '}], 'Location','northwest');

    if graphs2files
        saveas(gca,fullfile(figdir,strcat(mtn_root,'.fig')));
        saveas(gca,fullfile(figdir,strcat(mtn_root,'.png')));
    end
end

% Function to produce a bargraph showing changes in the location parameters
function bg = plot_bg(Fhm1, Fhm2, Pobj_e1, Pobj_e2, Visible, bg_root,...
        graphs2files, figdir, varargin)
    logFhmShift = log10(Fhm2) - log10(Fhm1);
    logPobjShift = log10(Pobj_e2) - log10(Pobj_e1);

    if size(varargin,2) == 0
        span = max(logFhmShift, logPobjShift) - min(logFhmShift, logPobjShift);
        ymax = max(logFhmShift, logPobjShift) + 0.2 * span;
        ymin = min(logFhmShift, logPobjShift) - 0.2 * span;
    else
        ymin = varargin{1};
        ymax = varargin{2};
    end

    bg = figure;
    bg.Visible = Visible;
    bg.Position = [0 0 600 800];
    bar(1,logFhmShift, 'r');
    hold on;
    bar(2,logPobjShift, 'b');
    hold off;
    ax = findall(gca,'Type','Axes');
    ax.Position = [0.2 0.15 0.6 0.8]; %[left bottom width height]
    ax.XLim = [0.5,2.5];
    ax.YLim = [ymin, ymax];
    ax.YLabel.String = 'Shift';
    ylabpos = ax.YLabel.Position;
    ylabpos(1) = 0.15;
    ax.YLabel.Position = ylabpos;
    ax.XAxis.FontSize = 22;
    ax.YAxis.FontSize = 22;
    ax.YGrid = 'on';
    ax.XTick = [1,2];
    ax.XTickLabel = {'log_{10 }(F_{hm })', 'log_{10 }(P_e )'};
    bh = findall(gcf,'Type','Bar');
    bh(1).BarWidth = 0.6;
```

```
    bh(2).BarWidth = 0.6;

    if graphs2files
        saveas(gca,fullfile(figdir,strcat(bg_root,'.fig')));
        saveas(gca,fullfile(figdir,strcat(bg_root,'.png')));
    end
end

% Function to produce a bargraph showing changes in the location parameters
function bg = plot_bgStar(Fhm1, Fhm2, FhmStar1, FhmStar2, Pobj_e1, Pobj_e2, PobjEstar1,
    Visible, bg_root, graphs2files, figdir, varargin)
    logFhmShift = log10(Fhm2) - log10(Fhm1);
    logPobjShift = log10(Pobj_e2) - log10(Pobj_e1);
    logFhmStarShift = log10(FhmStar2) - log10(FhmStar1); % These are the corrected valu
    logPobjStarShift = log10(PobjEstar2) - log10(PobjEstar1); % These are the correcte

    if size(varargin,2) == 0
        span = max(logFhmShift, logPobjShift) - min(logFhmShift, logPobjShift);
        ymax = max(logFhmShift, logPobjShift) + 0.2 * span;
        ymin = min(logFhmShift, logPobjShift) - 0.2 * span;
    else
        ymin = varargin{1};
        ymax = varargin{2};
    end

    bwidth = 0.6; % width of bars
    FhmStarX = [1-(bwidth/2), 1+(bwidth/2)];
    FhmStarY = [logFhmStarShift, logFhmStarShift];
    PeStarX = [2-(bwidth/2), 2+(bwidth/2)];
    PeStarY = [logPobjStarShift, logPobjStarShift];

    bg = figure;
    bg.Visible = Visible;
    bg.Position = [0 0 600 800];

    bar(1,logFhmShift, 'r');
    hold on;
    bar(2,logPobjShift, 'b');

    bh = findall(gcf,'Type','Bar');
    bh(1).BarWidth = bwidth;
    bh(2).BarWidth = bwidth;

    % Add lines showing corrected location parameters
    plot(FhmStarX, FhmStarY, 'Color','c', 'LineStyle', '-.', 'LineWidth',6);
    plot(PeStarX, PeStarY, 'Color','c', 'LineStyle', '-.', 'LineWidth',6);
    hold off;

    ax = findall(gca,'Type','Axes');
    ax.Position = [0.2 0.15 0.6 0.8]; %[left bottom width height]
    ax.XLim = [0.5,2.5];
    ax.YLim = [ymin, ymax];
    ax.YLabel.String = 'Shift';
    ylabpos = ax.YLabel.Position;
```

```
    ylabpos(1) = 0.15;
    ax.YLabel.Position = ylabpos;
    ax.XAxis.FontSize = 22;
    ax.YAxis.FontSize = 22;
    ax.YGrid = 'on';
    ax.XTick = [1,2];
    ax.XTickLabel = {'log_{10 }(F_{hm })', 'log_{10 }(P_e )'};
    bh = findall(gcf,'Type','Bar');
    bh(1).BarWidth = 0.6;
    bh(2).BarWidth = 0.6;

    if graphs2files
        saveas(gca,fullfile(figdir,strcat(bg_root,'.fig')));
        saveas(gca,fullfile(figdir,strcat(bg_root,'.png')));
    end
end

% Modify attributes of a 2D graph. handle_attribute and attribute_value can be cell arr
% in which case attribute value must have the same number of rows as the length of the
% and the same number of columns as the number on handle_attributes.
% The restrictions of the set command apply. For example, this function cannot modify l
function out_graph = modify_2D_graph(in_graph, handle_type, handle_attribute, attribute
    gFileNam, graphs2files, figdir)
    gh = findall(in_graph, 'Type', handle_type);
    set(gh, handle_attribute, attribute_value);

    if strcmp(handle_attribute,'XLim') || strcmp(handle_attribute,'YLim')
        ax = findall(gcf, 'Type', 'Axes');
        XlabPos = ax.XLabel.Position;
        YlabPos = ax.YLabel.Position;

        LinLogX = ax.XAxis.Scale;
        switch LinLogX
            case 'linear'
                XlabPos(1) = ax.XLim(1) + ((ax.XLim(2) - ax.XLim(1)) / 2);
                YlabPos(1) = ax.Xlim(1) - ((ax.XLim(2) - ax.XLim(1)) * 0.1); % 10% belo
            case 'log'
                XlabPos(1) = 10^(log10(ax.XLim(1)) + ((log10(ax.XLim(2)) - log10(ax.XLi
                YlabPos(1) = 10^(log10(ax.XLim(1)) - ((log10(ax.XLim(2)) - log10(ax.XLi
        end
        ax.XLabel.Position = XlabPos;

        LinLogY = ax.YAxis.Scale;
        switch LinLogY
            case 'linear'
                XlabPos(2) = ax.YLim(1) - ((ax.YLim(2) - ax.YLim(1)) * 0.0/075); % 7.5%
                YlabPos(2) = ax.YLim(1) + (ax.YLim(1) + ((ax.YLim(2) - ax.YLim(1)) / 2)
            case 'log'
                XlabPos(2) = 10^(log10(ax.YLim(1)) - ((log10(ax.YLim(2)) - log10(ax.YLi
                YlabPos(2) = 10^(log10(ax.YLim(1)) + ((log10(ax.YLim(2)) - log10(ax.YLi
        end
        ax.YLabel.Position = YlabPos;
    end
```

```
    out_graph = gcf;

    if graphs2files
        figfile = fullfile(figdir, strcat(gFileNam, '.fig'));
        saveas(gcf,figfile);
        pngfile = regexprep(figfile, '.fig','.png');
        saveas(gcf,pngfile);
    end
end
```

Functions that display multi-panel Matlab figures

```
function dual_sub = dual_subplot(g1, g2, dual_sub_out, graphs2files, figdir)
    dual_sub = figure;
    dual_sub.Visible = 'off';
    dual_sub.Units = 'pixels';
    dual_sub.Position = [0 0 1500 600];
    colormap 'jet';

    ax(1) = findall(g1, 'Type', 'Axes');
    ax(2) = findall(g2, 'Type', 'Axes');

    % Collect legend properties if a legend exists in the input figures
    for j = 1:2
        lg(j) = false;
        if ~isempty(ax(j).Legend)
            lg(j) = true;
            lgh = ax(j).Legend;
            lgstr(j).str = lgh.String;
            lgstr(j).tstr = lgh.Title.String;
        end
    end

    for j = 1:2
        ax_copy(j) = copyobj(ax(j), dual_sub);
        sph(j) = subplot(1,2,j,ax_copy(j));

%          if ~isempty(ax(j).Legend)
        if lg(j)
%              splg(j) = legend(sph(j),lgstr(j).str,'Location','southeast');
            % This position is rigid, but it should work in this specific case.
            % The reward-growth graphs have legends; the mountain plots do not.
            splg(j) = legend(sph(j),lgstr(j).str,'Location','best');
            splg(j).Title.String = lgstr(j).tstr;
        end
    end

    if graphs2files
        saveas(dual_sub,fullfile(figdir,strcat(dual_sub_out,'.fig')));
        saveas(dual_sub,fullfile(figdir,strcat(dual_sub_out,'.png')));
    end
end

% function to plot the quad-panel display of the contour and bar graphs
```

```
% cont1 is plotted twice, once in the upper left and once in the lower right
function quad_sub = quad_subplot(cont1, cont2, bg, quad_sub_out, bg_root, ...
        graphs2files, figdir)
    quad_sub = figure;
    quad_sub.Visible = 'off';
    quad_sub.Units = 'pixels';
    quad_sub.Position = [0 0 1500 1500];
    colormap 'jet';

    fh(1) = cont1;
    fh(2) = bg;
    fh(3) = cont2;
    fh(4) = cont1;

    for j = 1:4
        ax(j) = findall(fh(j), 'Type', 'Axes');
    end

    % Collect textbox properties if a textbox exists in the input figure
    for j = 1:4
    tbh = findall(fh(j),'Type','TextBox');
        tb(j) = false;
        if ~isempty(tbh)
            tb(j) = true;
            tbstr(j) = tbh.String;
        end
    end

    bg_str = findall(ax(2), '-Property', 'FontSize');
    bg_str = findall(bg_str(:),'-Property','FontSize');

    for j = 1:length(bg_str)
        bg_str(j).FontSize = bg_str(j).FontSize * 1.75;
    end

    for j = 1:4
        ax_copy(j) = copyobj(ax(j), quad_sub);
        sph(j) = subplot(2,2,j,ax_copy(j));

        if tb(j)
            axpos = sph(j).Position;
            x_offset = axpos(3) * 0.7;
            y_offset = axpos(4) * -0.1;
            tbdim = [(axpos(1) + x_offset) (axpos(2) + y_offset) 0.1 0.1];
            ann(j) = annotation('textbox', tbdim, 'String', tbstr(j),'FitBoxToText','or
            ann(j).FontSize = 16;
        end
    end
    if graphs2files
        saveas(quad_sub,fullfile(figdir,strcat(quad_sub_out,'.fig')));
        saveas(quad_sub,fullfile(figdir,strcat(quad_sub_out,'.png')));
    end
end
```

```
function out_fig = adjust_right_panel(dual_sub, h, v)
    out_fig = dual_sub;
    out_fig.Visible = 'off';
    ax = findall(out_fig,'Type','Axes');

    ax1_pos = ax(1).Position;
    ax1_ip = ax(1).InnerPosition;
    ax1_op = ax(1).OuterPosition;
    ax(1).InnerPosition = ax(1).OuterPosition; % tight borders

    lgutter = ax1_op(1) - 0.5;
    panel_width = 0.5 - lgutter;
    panel_height = 1;

    new_ax_width = ax1_pos(3) * h; % rescale height
    new_ax_height = ax1_pos(4) * v; % rescale width

    new_hmargin = (panel_width - new_ax_width) / 2;
    new_vmargin = (panel_height - new_ax_height) / 2;

    ax1_newxpos = 0.5 + lgutter + new_hmargin;
    ax1_newpos(1)= ax1_newxpos;

    ax1_newvpos = new_vmargin; % unnecessary, but included for consistency
    ax1_newpos(2) = ax1_newvpos;

    ax1_newpos(3) = new_ax_width;
    ax1_newpos(4) = new_ax_height;

    ax(1).Title.String = 'simulated data';

    ax(1).Position = ax1_newpos;
end
```

Functions that create & display images from stored files

```
function show_imported_graphic(gnam, mag, ImpFigDir)
    imshow(fullfile(ImpFigDir,gnam),'Border','tight','InitialMagnification',mag);
end

function F = make_fig_from_png(png_file, mag)
    img = imread(png_file, 'png');
    sz = size(img);
    F = figure;
    image(img);
    pos = F.Position; % the conventional x and y dimensions are reversed
    pos(3) = sz(2) * mag;
    pos(4) = sz(1) * mag;
    F.Position = pos;
    axis tight;
    ah = findall(F,'Type','Axes');
    ah.Visible = 'off';
    F.Visible = 'off';
end
```
