# Supplementary material for: Dopamine neurons do not constitute an obligatory stage in the final common path for the evaluation and pursuit of brain stimulation reward
Source: PLoS One. 2020 Jun 5;15(6):e0226722. doi: 10.1371/journal.pone.0226722 (PMC7274413; doi:10.1371/journal.pone.0226722)
Supplement: Attachment — Submitted filename: PLOS_ONE_rebuttal_letter_v3.pdf [file pone.0226722.s002.pdf]

Center for Studies in Behavioral Neurobiology
FRQS Groupe de recherche en neurobiologie comportementale

April 14, 2020

Dr. Juan M. Dominguez
Professor
Alma Idell Carlson Fellow
Department of Psychology and
Department of Pharmacology & Toxicology
The University of Texas at Austin

Dear Dr. Dominguez,

Thank for your message of February 13, 2020 and for the exceptionally helpful comments of the reviewers on our manuscript, **PONE-D-19-33493**. Our responses are detailed below along with a description of the revisions. Before delving into the specific critiques and suggestions, we wish to offer some overarching comments concerning the reviews and the changes they have inspired.

Both reviewers view the paper as important:

> Reviewer 1: "Bottom line: This is an unusually important paper, strongly challenging a widely and strongly held belief using state-of-the-art methods and exceptionally punctilious quantitative modeling of hypotheses specified in unusual detail."

> Reviewer 2: "This result is very important because it addresses the long-standing controversies surrounding dopamine's behavioral function (i.e., whether it plays role in motivation, effort exertion, reinforcement, learning, or some combination of these). The result forces us to re-think the interpretation of 50 years of experimentation with electrical MFB stimulation with respect to dopamine, and additionally forces us to broaden our thinking about what optogenetic stimulation of dopamine neurons actually does at behavioral, circuit and computational levels."

We are very glad that the reviewers reached these conclusions, and we understand that their comments are aimed at helping us communicate the ideas and findings more effectively than we did in the initial submission.

UNIVERSITÉ Concordia UNIVERSITY

Phone: +1 514.848.2424 Ext. 2191; E-mail: peter.shizgal@concordia.ca; Web: csbn.concordia.ca/Faculty/Shizgal
Dept. Psychology, CSBN, SP244, Concordia University, 7141 Sherbrooke St. W., Montréal, QC, Canada H4B 1R6

Reviewer 1 found the main argument of the paper to be buried under technical detail and to appear far too late in the exposition. He advocates making that argument earlier, more clearly, and more simply. We agree and have done our best to do that in the revised manuscript, as we detail below. However, we fear that the reviewer's view of how much simplicity can be attained may rest, at least in part, on a misunderstanding that we inadvertently encouraged. The reviewer states:

> "In short, we now know that blockading dopaminergic transmission ***does not diminish the reinforcing effect*** of brain stimulation reinforcement; it ***increases some cost variable*** to the point where the rats will no longer work for that wonderful reinforcement" and
>
> "dopamine transporter blockade … ***increases the reinforcing intensity*** of oICCS but it does not increase the reinforcing intensity of eICSS"

Here, the reviewer is imbuing the mountain model with a superpower that it does not possess, at least in its current form. It does not support unambiguous inference of reinforcing intensity from behavioral data. However, it ***does*** support an unambigous inference about a particularly revealing change in the function that translates the pulse frequency into the reinforcing intensity. That inference, when applied to the current data and the results of our prior eICSS study of the effect of specific dopamine-transporter blockade is sufficient to sink the series-circuit model and motivate the replacement we propose.

Reviewer 1 has kindly identified himself as Charles R. Gallistel. It is he who first conceived the core idea of the paper, the reward-growth function, and it was he and his team who first marshalled empirical data to describe the form of this function. We speculate that some combination of the simplified diagram we used to introduce the mountain model (previously Fig 1, now Fig 2) and the label on the X axis of the mountain ("price") encouraged reviewer 1, a most distinguished scholar of brain stimulation reward, to form an exaggerated view of what the mountain model achieves. That tells us that something was wrong with the way in which we introduced the model. We have done our best to correct this.

First, we provide a figure up front (new Fig 1) to show the reward-growth function, illustrate the orthogonal shifts produced by changes in input sensitivity and output amplification, and reveal how these produce correspondingly orthogonal changes in the position of the mountain.

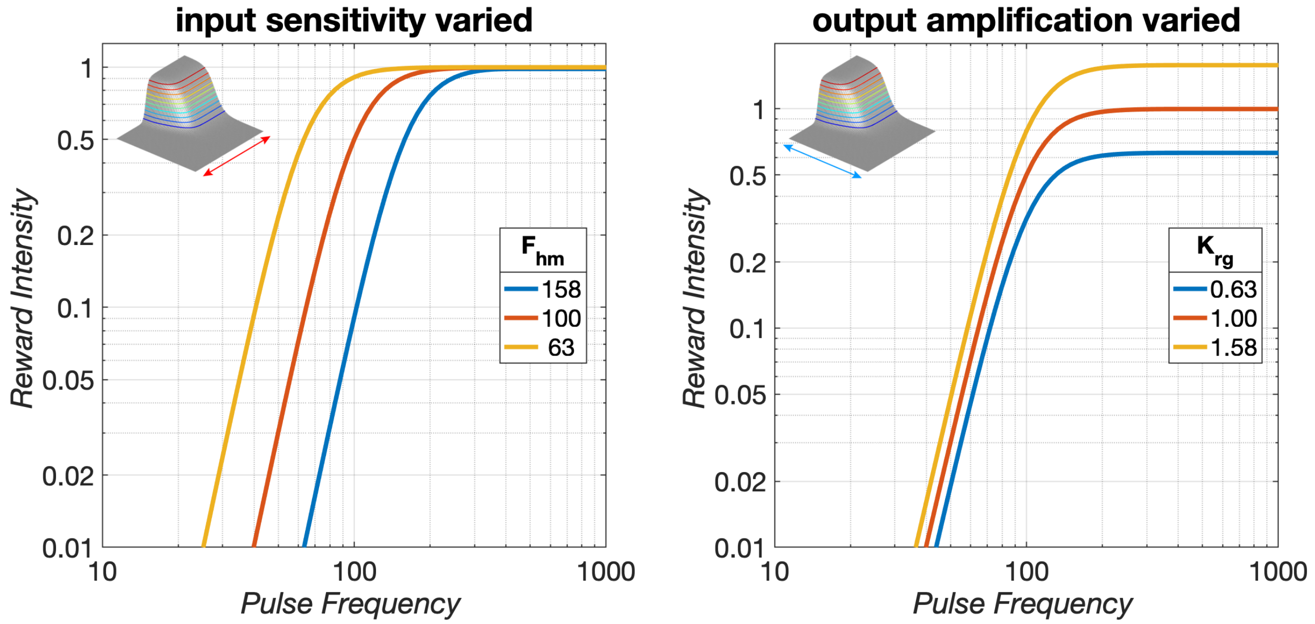

The revised paragraph that introduces this figure (lines 102-114) and the caption are as follows:

> "Simmons and Gallistel [23] brought out a key feature of the reward-growth function: given a sufficiently high current, reward intensity saturates at pulse frequencies well within the frequency-following capabilities [8] of the directly stimulated substrate. In other words, reward intensity levels off as pulse frequency increases even though the output of the directly stimulated neurons continues to grow. The reward-growth function they described is well fit by a logistic [33], a function that is shaped like an inverted hockey stick (Fig 1) when plotted on double logarithmic coordinates. As we will show, it is the non-linear form of this growth function that prevents the series-circuit model from explaining the differential effects of dopamine-transporter blockade on eICSS and oICSS. To account for the oICSS data, the contribution of dopamine neurons must be brought to bear on the input side of the reward-growth function as well as on the output side. To account for the eICSS, dopamine neurons must intervene on the output side but not on the input side."

> Caption: "Gallistel and colleagues have shown that the pulse frequency is translated into the intensity of the rewarding effect by a saturating function [21-23,28], such as a logistic (Eq S11). We assume that in those experiments, the induced firing frequency can be equated to the pulse frequency [8]. The position of the growth curve along the X axis is determined by the pulse frequency that drives reward intensity to half its maximal value (F_{hm}, also

called $F_{pulse_{hm}}$ below), whereas the position along the Y axis (e.g., the maximal reward intensity attained) is determined by a parameter we call K_{rg}. In the left panel, F_{hm}, is varied while K_{rg} is held constant, whereas in the right panel, K_{rg} is varied while F_{hm} is held constant. The inserts are described below, after the reward mountain has been introduced. The pulse frequencies shown here are typical of eICSS experiments."

Later in the introduction (lines 159-179), we flesh out the argument:

"Fig 2 shows the content of the green box in Fig 2 labeled "benefit:" the reward-growth function. Shifts of this function along the pulse-frequency axis (left panel) reflect changes in ***input*** sensitivity. These changes alter the pulse frequency required to drive reward intensity to a given proportion of its maximum. This is tantamount to rescaling the ***input***. When input sensitivity changes, the reward-growth function shifts laterally, dragging the reward mountain with it along the pulse-frequency axis (see insert). In contrast, one of the factors that can shift the reward mountain along the cost axis (right panel) is a change in ***output*** amplification (called "gain" in our previous papers [34,39,46,47]). This alters the maximal reward intensity without changing the pulse frequency required to achieve this maximum (or any other proportion of the maximal intensity); the reward-growth function is shifted vertically along the logarithmic axis representing reward intensity. Because all non-zero reward intensities have now been boosted or cut, willingness to pay for them changes accordingly, and the mountain shifts along the cost axis (see insert). However, that shift is not unique. For example, due to the scalar combination of benefits and costs (as represented by the circle containing an X that combines benefits and costs in Fig 2), indistinguishable shifts along the cost axis result from multiplying the benefits by a constant or from dividing the costs by the same constant. Thus, the reward-mountain method does not unambiguously distinguish changes in benefits and costs. What it does instead is to distinguish changes in input sensitivity (left panel of Fig 1) from all the other determinants of reward valuation."

We delve into the reward-growth function formally and in greater depth in the discussion [lines 612-700; Figs 11, S33, S34].

For reference, here is Fig 2 (formerly Fig 1):

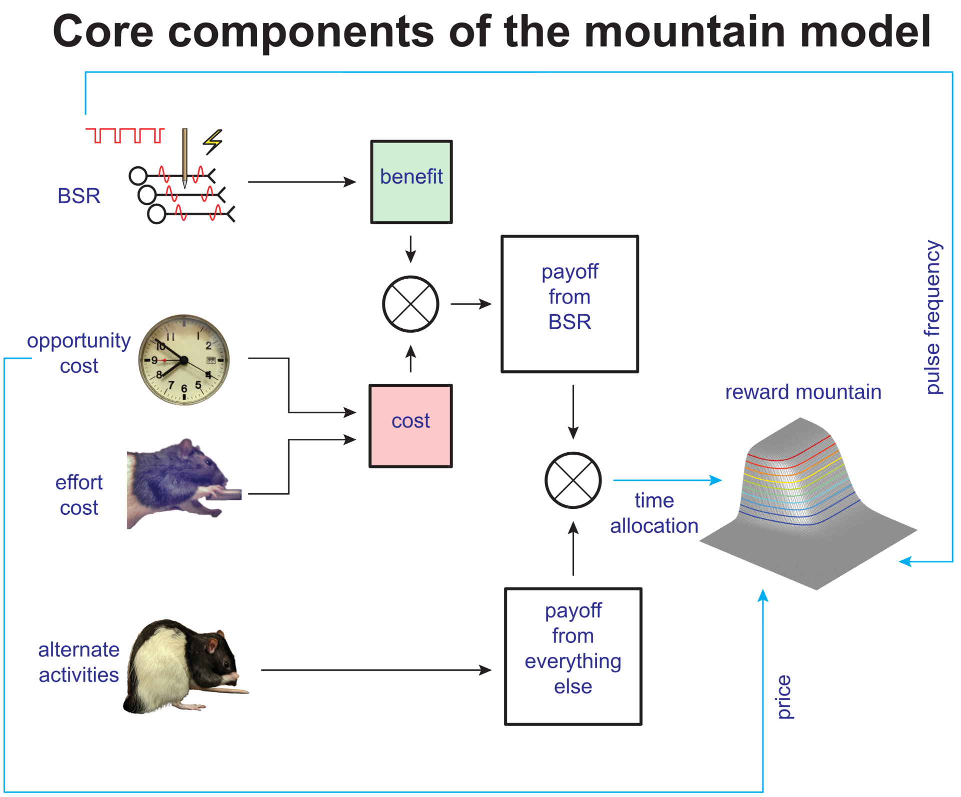

In the following paragraphs, we list revisions made in order to state the principal finding simply and early, as reviewer 1 advises. We also include some phrases retained from the original version that already made such statements.

> [end of abstract, unchanged]: "… we propose that midbrain dopamine neurons and neurons with non-dopaminergic, MFB axons constitute parallel limbs of brain-reward circuitry that ultimately converge on the final-common path for the evaluation and pursuit of rewards."

> [end of authors' summary, lightly revised]: "Mathematical modeling of the results argues for a new view of the structure of brain reward circuitry. On this view, the pathway in which the dopamine neurons is embedded is only one of multiple parallel channels that process reward signals in the brain. To achieve a full understanding of how goals are evaluated, selected and pursued, all these channels must be identified and investigated."

> [lines 34-36, added]: "The results challenge a long-dominant model in which dopaminergic neurons constitute an obligatory stage in the final common path for the rewarding effect manifested in ICSS."

[lines 57-59, revised]: “We thus propose a fundamental revision in which the phasic firing of dopaminergic neurons constitutes only one of the multiple signals that converge on the final common path subserving the evaluation and pursuit of rewards.”

Addition of Fig 1 and its caption, as described in lines 102-114 and lines 159-179, provided above.

[lines 190-195]: “We show here that one of the contributions of dopaminergic neurons to ***oICSS*** is brought to bear at, or prior to, the input to the reward-growth function; it alters the input sensitivity of the reward-growth function. This contrasts sharply with the case of ***eICSS*** in which such changes in input sensitivity have rarely been seen following perturbation of dopaminergic neurotransmission in reward-mountain experiments [34,39,47]. ”

Additional comments and suggestions by reviewer 1

Reviewer 1 asks: “Could not the locus of convergence be memory itself, not the integration of cost and intensity? Might not the trade-off between cost and reinforcing intensity be computed post memory at the time of deciding on an action?” Indeed. In prior papers [39,46,47], we included a figure that shows storage of reward intensity, reward probability, opportunity cost, and effort cost in memory and the computation of time allocation performed upon the retrieval of this information. By so doing, we were likely getting out too far ahead of our skis; it strikes me now that we don’t really have the empirical goods on that issue. We now admit as much in the following (lines 1015-1023):

“Indeed, we see the models discussed here as over-simplified rather than over-complicated. For example, they say nothing about fundamental matters such as the functional specialization of dopamine subpopulations, how signals from the *milieu interne* modulate the decision variables, which operations are performed prior to the recording of key quantities in memory or after their retrieval, or how the subjects learn and update the reward intensities and costs that determine their behavioral allocation. Nonetheless, we argue that the combination of the modeling, simulation and empirical work provides a new perspective on the structure of brain-reward circuitry while challenging a long-established view.”

Reviewer 1: “In addition to making the data available at https://spectrum.library.concordia.ca/, I suggest that they and the (sic) code used to obtain the results and in the modeling be uploaded to a public GitHub repository. Obviously, the simulation script should also go there. I have had more than one bad experience with supplementary materials supposedly preserved by journal on line. More than one of my own such depositions have become inaccessible. GitHub is much more trustworthy.”

Good idea. We promise to look into that as soon as the revision has been submitted.

Reviewer 1: “This dependence is described by a surface in a three-dimensional space (reward-seeking performance versus pulse frequency and response cost (old Fig 1))." I suggest adding, closely analogous in its construction and interpretive function to the copula (the bivariate cumulative distribution function) in bivariate statistic.” He adds: “The reward-mountain figure looks exactly like a copula. This is no accident, because at the plateau at the top of the reward mountain represents describes the stimulation- and task-parameter space such that the probability of working for reinforcement is 1. The plateau of a copula describes the parameter space within which both cumulative probabilities are 1. Moreover, the function of copulas is to remove ambiguities inherent in the univariate cumulative probability functions. (A copula is a joint cumulative distribution function.)”

With all due respect, we prefer to pass on this one. Any teacher who has attempted to explain a difficult concept or mechanism by means of an analogy has learned the hard lesson that to be effective, the analogy has to be simpler and more intuitive than what it is intended to explain. For most readers, we doubt that the copula will pass that test, familiar as it may be to the very small number of statisticians likely to read our paper in depth. Is the manuscript not complicated enough already?

We appreciate that anyone who looks at a plot of the joint cumulative normal (or similar) distribution (e.g., https://www.mathworks.com/help/stats/mvncdf.html) will see a striking similarity to the mountain surface. We also appreciate that time allocation might be portrayed as the average instantaneous probability that the rat is holding down the lever within a trial. That said, we wonder (perhaps due to our ignorance) whether the analogy is really apt. Isn’t the argument of the cumulative distribution function supposed to be a random variable? In our understanding, neither the pulse frequency nor the price are random variables. We chose the specific tested values of these independent variables very deliberately and carefully! That seems quite different than the case of hydrological studies, for example, that have made use of copulas. In those studies (e.g., Salvadori & De Michele, 2007), the arguments of the marginal distributions are indeed random variables (e.g., flood peak, and flood volume), which are observed rather than manipulated by an experimenter. Even if one wishes to portray time allocation in probabilistic terms, isn’t it a probability density rather than a cumulative probability?

What bothers us most about the cupola anology is that it appears to obscure the multiple embedding of non-linear functions that must be kept in mind to understand how the mountain model works and what it adds to prior methods.

In the hydrological example, the researcher has indeed collected probability distributions which can be cumulated directly. In the case of the mountain, we wish to emphasize for the reader that the typical psychometric functions used in studies of reward pursuit (e.g., the rate-frequency and progressive-ratio curves) are actually the result of multiple embeddings (e.g., the frequency-following function within the counter, within the reward-growth function, within the payoff-generating function, within the behavioral-allocation function; the effort-cost function within the payoff-generating function, within the behavioral-allocation function etc.). It is very difficult to form accurate intuitions about the results of such embeddings, particularly when so many of the functions are non-linear. That is one of the principal reasons why the modeling and simulation work is such an important complement to the emprical results.

Reviewer 1: “Whereas the crude response counts used initially to measure behavioral output are confounded by inherent non-linearity..." [should read ..."the inherent non-linearity of the performance function linking rate of response to reward magnitude, as well as ... because it is not clear to what function the mentioned nonlinearity applies.”

Our revision, guided by this suggestion [lines 23-28]: “Whereas the performance function linking response vigor to reward magnitude is inherently non-linear and is subject to distortion by disruptive side-effects of drugs and motoric activation, contemporary psychophysical methods "see through" this function so as to support inferences about the value of the induced reward as well as the form and parameters of the functions that map observable inputs and outputs into the variables that determine behavioral-allocation decisions.”

Reviewer 1: “p. 9: I suggest that in the paragraph explaining why the results in Figure 4 are ambiguous, … you reveal the result of the disambiguation in the plainest possible language. “

Our attempt to address this:

[lines 305-315]: “This ambiguity is removed by fitting the reward-mountain model, which expresses time allocation as a function of both the price and strength of the rewarding stimulation. In the three-dimensional space of the reward-mountain model, we can determine unambiguously the degree to which dopamine-transporter blockade displaces the mountain along the price and pulse-frequency axes. **By so doing, we distinguish the effect of dopamine-transporter blockade at, or prior to, the input to the reward-growth function (Fig 1) from the effect at, or beyond, the output of this function. As we will show, the fate of the series-circuit model hangs on that distinction and founders on the fact that specific dopamine-transporter**

blockade shifts the reward mountain for oICSS (present data), but not eICSS [34], along the pulse-frequency axis."

Reviewer 1: "I also suggest putting the discussion of other parametric work consistent with these results and conclusions be put after these conclusions from the present experiment in the Discussion rather than before, because the methods used here are so much more powerful than the methods used heretofore. There is altogether too much burying of the lead in this exposition. Those without a taste for technical detail may never get to the astonishing conclusions."

> [lines 448-457, at end of the second paragraph of the discussion]: "We show here that the reward mountain is shifted along the pulse-frequency axis by the specific dopamine-transporter blocker, GBR-12909. Thus, the transporter blocker acted as if to rescale the input to the reward-growth function for oICSS (i.e., via an action at, or prior to, the input). According to the series-circuit model, the directly activated neurons subserving eICSS of the medial forebrain bundle produce their rewarding effect by transsynaptic activation of the same midbrain dopamine neurons that were activated directly in the present study by optogenetic means. If so, specific dopamine transporter blockade must also shift the reward-growth function for eICSS along the pulse-frequency axis. However, it failed to do so in all 10 subjects of the study in which eICSS was challenged by the administration of GBR-12909 [34]."

Suggestions by reviewer 2

Reviewer 2: "The argument developed to explain why the parallel model accommodates the GBR12909-induced shift along the pulse frequency axis refers to "tonic dopamine" (in the Fig. 11 legend). But the authors nowhere explicitly state what they think the roles of tonic and phasic dopamine are with respect to the two mountain axes. Such an explanation would be helpful."

> [lines 57-59]: "We thus propose a fundamental revision in which the phasic firing of dopaminergic neurons constitutes only one of the multiple signals that converge on the final common path subserving the evaluation and pursuit of rewards."
>
> The reward-growth function for oICSS must take as its input phasic changes in dopamine release. We know this because reward-seeking behavior can adjust to changes in pulse frequency after a delivery of a single pulse train (Shizgal, P., Trujillo-Pisanty, I., Cossette, M., Conover, K., Carter, F., Pallikaras, V., Breton, Y.-A., & Solomon, R.B. Does phasic dopamine signalling play a causal role in reinforcement learning? Reinforcement Learning and Decision Making, July 9, 2019. http://rldm.org/papers/extendedabstracts.pdf)

[lines 717-724]: “In addition to increasing the amplitude of stimulation-induced dopamine transients, blockade of the dopamine transporter by GBR-12909 also increases the baseline ("tonic") level of dopamine [34, 70, 71]. The increase in dopamine tone could rescale the output of the reward-growth function upwards, reduce subjective effort costs and/or diminish the value of activities that compete with pursuit of the optical reward. Such effects could arise from increases in Krg and/or decreases in Kec or Kaa (Fig S16, Eqs S10, S11, S19,). Any combination of these effects could account for the observed rightward shifts of the reward mountain along the price axis (Figs 7,S23-S28).”

Reviewer 2: “On line 483, the authors write, “the rat is willing to sacrifice more leisure in order to obtain strong stimulation of a "good" eICSS site than a poorer one.” As the price gets higher, could it be that the animal is not so much interrupting effort to indulge in leisure, but rather he becomes fatigued, and a higher reward intensity makes it worth his while to work despite his fatigue? One could argue that taking a break from work is simply leisure, but the point here is that higher work requirements could induce fatigue and lead to more leisure-seeking.”

The reviewer is offering an alternative explanation for the form of the behavioral allocation function. Please keep in mind that both the frequency-sweep and price-sweep data are passed through the same behavioral-allocation function. If fatigue were to have set in at higher prices, wouldn’t one expect the price-sweep data to deviate more from the fitted surface that the frequency-sweep data, which were all collected at the same low price? I don’t see signs of this in Figs 5 and S17-S22. Please keep in mind that the lever is not mounted very high above the floor. The rats can keep the lever depressed by simply standing with one paw on the lever. They are big bruisers, typically weighing between 600-800 g at the time that the mountain data were collected. The force required to depress the lever is small in comparison to the body mass of the rats.

Reviewer 2: “Somewhat related to point #2, a growing literature implicates VTA dopamine neurons in arousal. To what extent could shifts along the price axis, such as that induced by GBR12909, be due to changes in arousal?”

Wouldn’t you expect increases in arousal to boost time allocation non-specifically? That would generate correlated decreases in F_{hm} and increases in P_e. However, the changes in these two location parameters are uncorrelated (lines 415-417 and Fig S29).

Reviewer 2: “In 1905, a paper was published in Annalen der Physik entitled “On the Electrodynamics of Moving Bodies.” Despite its nondescript (and uninformative) title, the

paper was widely read by physicists, who grew to appreciate the equations and ideas in the paper that described the Special Theory of Relativity. Alas, neuroscience is not like physics. A catchy title makes a big difference (e.g., "Accumbal D1R Neurons Projecting to Lateral Hypothalamus Authorize Feeding"). Fortunately, the authors have already supplied the beginnings of a title that actually describes their conclusions (unlike their present one) on line 980: "Dopamine neurons do not constitute an obligatory stage in the final common path for [reward] evaluation and pursuit."

We have adopted this very helpful suggestion.

Some speculation about the identity of the MFB component that contributes to reward intensity in a dopamine-independent way would be helpful.

By the early 1980s, over 50 distinguishable components of the MFB had been identified. No compendium has since been built associating origin, termination, trajectory, myelination, and neurochemical coding. That said, there is hope that we will not have all that long to wait, e.g., https://www.janelia.org/project-team/mouselight.

[lines 1040-1051]: "The convergence model elevates the status of the directly activated neurons subserving the rewarding effect of MFB stimulation. This model asserts that multiple, partially parallel, neural circuits can generate reward and that the dopamine neurons do not constitute an obligatory stage in the final common path for their evaluation and pursuit. From that perspective, it is important to intensify the search for the limb(s) of brain reward circuitry that may parallel the much better characterized dopaminergic pathways. Application of modern tracing methods that integrate approaches from neuroanatomy, physiology, optics, cell biology and molecular biology (e.g., reference) may well achieve what application of the cruder, older tools failed to accomplish. The detailed psychophysical characterization of the quarry that has already been achieved, particularly the evidence for myelination and axonal trajectory (references), can guide the application of such methods."

In the sentences quoted above, we suggest how promising MFB components could be identified and characterized. We aren't comfortable going beyond this. On what basis are we to select a subset of MFB components to highlight before the necessary experimental work has been conducted?

Reviewer 2: It might also be useful to include a simplified version of figure 10 that explains in intuitive terms why the GBR12909 causes shifts along the reward intensity axis for optical stimulation but not electrical stimulation reward.

That is the point of the upper row of panels in Figs 12 (formerly Fig 11) and S35, as explained in some detail on lines 812-861. At least in our hands, the message disappears once we try to simplify further.

Comments from the editor

A version of the manuscript with marked changes is requested. The manuscript was prepared using a LaTeX editor that doesn't provide tracked changes. Thus, we used a separate application to compare the pdf output files. Two outputs, both in html and pdf format, are included. One interleaves sections of the originally submitted 2019-12-03 and current 2020-04-14 versions. The second places the two versions side-by-side. That version might be particularly useful on a system equipped with an ultra-widescreen display or a means of spanning a window across two displays.

Daniel Palacios was a full-time student at Concordia University at the time that he contributed to this project. He was hired by OMMAX Digital Strategy only afterwards. That firm had no role whatsoever in the project. Therefore, we have deleted Daniel's affiliation with that firm.

Editor: "Please ensure that you refer to Figure 12 in your text." That reference was in error. The reference should have been to Fig S35. We have corrected that error in the revised manuscript. (The former Fig 11 is now Fig 12. The numbering of the figures in the supplementary-information file has not changed.)

Closing remarks

We are deeply grateful to the reviewers for their very constructive and thoughtful feedback, for working through a long, complex manuscript so carefully and now working through a long letter. In our view, the manuscript has been much improved due to their efforts. We thank the editor for his stewardship of the review process and for the consideration he has shown regarding our timing constraints. Merci beaucoup!

We hope that our responses to the input from the reviewers will prove satisfactory.

Sincerely,

Peter Shizgal

Peter Shizgal, PhD
Distinguished Professor Emeritus and
Honorary Concordia University Research Chair
